# NRIP1 is activated by C-JUN/C-FOS and activates the expression of *PGR*, *ESR1* and *CCND1* in luminal A breast cancer

**DOI:** 10.1038/s41598-021-00291-w

**Published:** 2021-10-27

**Authors:** Renata Binato, Stephany Corrêa, Carolina Panis, Gerson Ferreira, Igor Petrone, Igor Rodrigues da Costa, Eliana Abdelhay

**Affiliations:** 1 Stem Cell Laboratory, Divisão de Laboratórios do CEMO, Bone Marrow Transplantation Unit, National Cancer Institute (INCA), Praça da Cruz Vermelha 23, 6° andar ALA C, Rio de Janeiro, RJ CEP 20.230-130 Brazil; 2 Instituto Nacional de Ciência e Tecnologia Para o Controle do Câncer (INCT), Rio de Janeiro, RJ Brazil; 3 Laboratório de Mediadores Inflamatórios, Universidade Estadual do Oeste do Paraná, UNIOESTE, Campus Francisco Beltrão, Paraná, Brazil; 4 LADETEC, Departamento de Bioquímica, Instituto de Química, Universidade Federal do Rio de Janeiro (UFRJ), Rio de Janeiro, RJ Brazil

**Keywords:** Cancer, RNAi, Transcriptomics

## Abstract

Using chip array assays, we identified differentially expressed genes via a comparison between luminal A breast cancer subtype and normal mammary ductal cells from healthy donors. In silico analysis confirmed by western blot and immunohistochemistry revealed that C-JUN and C-FOS transcription factors are activated in luminal A patients as potential upstream regulators of these differentially expressed genes. Using a chip-on-chip assay, we identified potential C-JUN and C-FOS targets. Among these genes, the *NRIP1* gene was revealed to be targeted by C-JUN and C-FOS. This was confirmed after identification and validation with transfection assays specific binding of C-JUN and C-FOS at consensus binding sites. *NRIP1* is not only upregulated in luminal A patients and cell lines but also regulates breast cancer-related genes, including *PR*, *ESR1* and *CCND1.* These results were confirmed by NRIP1 siRNA knockdown and chip array assays, thus highlighting the putative role of *NRIP1* in *PGR*, *ESR1* and *CCND1* transcriptional regulation and suggesting that *NRIP1* could play an important role in breast cancer ductal cell initiation.

## Introduction

Breast cancer (BC) is a complex disease with several presentations and thus results in various clinical implications. Twenty years ago, the use of molecular high-throughput platforms enhanced the stratification of the molecular subtypes based on gene expression profiles. This classification divides all BCs into the following five different subtypes: luminal A, luminal B, Her2 enriched, basal and normal^1,2^.

The luminal A subtype is the most frequent ductal invasive breast cancer^3^ and has the best prognosis among BC tumors. Early diagnosis usually shows a tumor grade I or II without a lymph node compromised status.

Although considerable knowledge has been accumulated in recent decades regarding BC, many gaps remain regarding tumor establishment and the mechanisms underlying tumor initiation. The exact mechanisms by which breast cancer is initiated and becomes invasive are unknown.

Estrogen, a steroid hormone important for breast cancer development, acts through estrogen receptors alpha and beta. Signaling can occur by a canonical pathway where ER activates transcription through binding the nuclear estrogen receptor^4,5^. In the noncanonical pathway, extracellular estrogen binds to its receptor in the plasma membrane and activates the PI3K or Ras signaling pathway^6^. In both cases, coactivators are important for promoter binding. Several coactivators have been identified, and the most predominant include ERAP-160, NRIP1 (RIP140), SRC-1, CBP, p300, TIF-2 and A1B1^7,8^.

In the present work, we showed that during transformation from a normal ductal cell to a luminal A BC, C-JUN and C-FOS are activated and together activate the expression of *NRIP1.* Moreover, we suggest that *NRIP1* plays a putative role in *PGR*, *ESR1* and *CCND1* transcriptional regulation.

## Results

### Gene expression comparison between luminal A ductal cells and normal ductal cells indicates that C-JUN and C-FOS are putative regulators of differential expressed genes

Using a fold change ≥ 5 to compare gene expression between Luminal A tissues and healthy tissue, we identified 133 differentially expressed genes (DEGS) (Supplementary Table S2).

**Figure 1:**
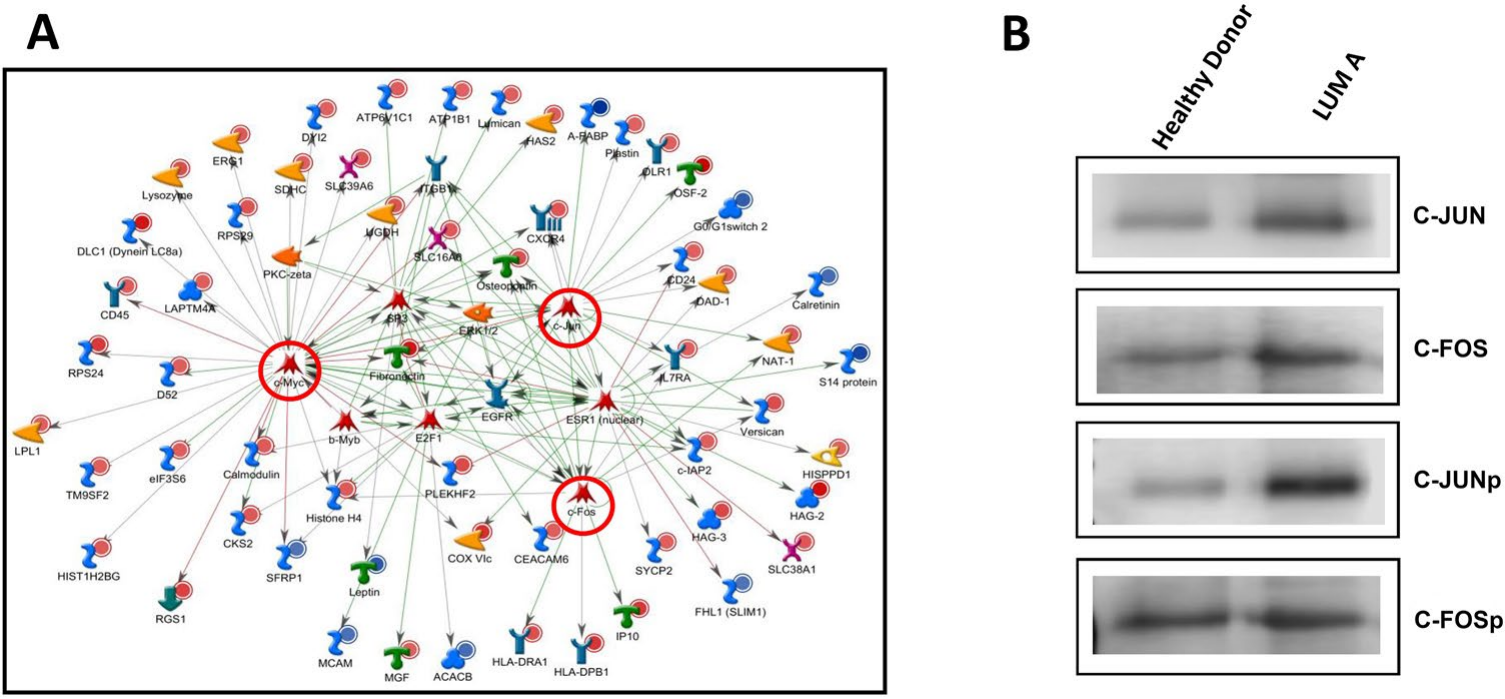
Comparison of the gene expression profiles of luminal A patients and healthy donors indicates that C-JUN and C-FOS are putative candidate regulators of the differentially expressed genes. (**A**) Network based on putative upstream regulators illustrates C-JUN, C-FOS and C-MYC as putative candidates as upstream regulators of the differentially expressed genes in luminal A × healthy donors. Overexpressed genes are marked with red circles; and downregulated genes are marked with blue circles. (**B**) Western blot analysis of C-JUN, C-FOS, C-JUN phosphorylated (C-JUNp) and C-FOS phosphorylated (C-FOSp) antibodies. Thirty micrograms of protein extracts from Healthy Donor and LUM A breast tissues were separated by SDS-PAGE and probed with the previously mentioned specific antibodies. Rouge Ponceau staining was used as the loading control.

The application of an in silico analysis with MetaCore software (Clarivate Analytics, USA) indicated that C-JUN, C-FOS and C-MYC were putative candidate regulators of our differentially expressed genes (Fig. 1A).

**Figure 2:**
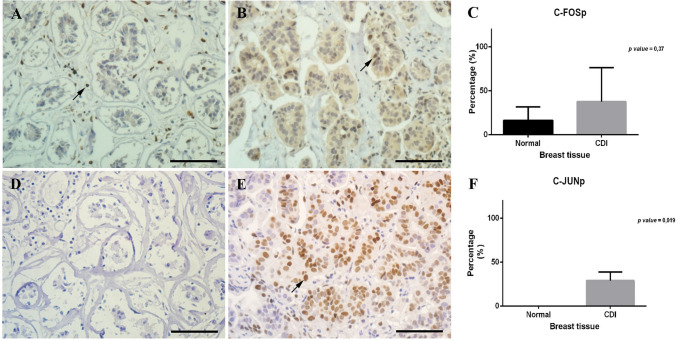
Immunohistochemistry assay confirming increased expression of C-JUN and CFOS in luminal A patients. (**A,B**) Using an antibody against phosphorylated C-FOS, we observed that C-FOS expression was slightly increased in luminal A patients (**B**) compared to mammoplasty tissue (**A**). (**C**) Graphical representation of C-FOS. Statistical analyses showed increased C-FOS expression in luminal A tissue compared to normal breast tissue (p-value = 0.37). (**D,E**) Using an antibody against phosphorylated C-JUN, we observed that C-JUN expression was slightly increased in the luminal A patients (**E**) compared to that in the mammoplasty tissue (**D**). (**F**) Graphical representation of C-JUN. Statistical analyses showed a significant increase in C-JUN expression in luminal A tissue compared to normal breast tissue (p-value = 0.019). Scale bar: 100 µm; magnification: × 400. The arrows indicate positive labeling.

As shown in Fig. 1B, both C-JUN and C-FOS were overexpressed in the luminal A samples. We also evaluated the expression of phosphorylated C-JUN and C-FOS proteins, and both were increased in luminal A tissues (Fig. 1B, Supplementary Fig. S1). We also performed immunohistochemistry assays using C-JUN and C-FOS phosphorylated antibodies (Fig. 2).

Together, our data indicates that activation of both the C-JUN and C-FOS proteins in luminal A tissues may be related to the increased expression of some differentially expressed genes.

### *NRIP1* is a target of C-JUN and C-FOS in luminal A breast cancer

To identify the targets of C-JUN and C-FOS transcription factors, a chromatin immunoprecipitation (ChiP) assay with anti-C-JUN and anti-C-FOS antibodies was carried out, followed by a promoter chip array assay using MCF7 and HMEC cell lines. We defined the C-JUN and C-FOS targets in each cell line using a p-value of < 0.01 as a cutoff.

Next, we generated a Venn diagram (http://bioinformatics.psb.ugent.be/webtools/Venn/) to identify the exclusive targets for both transcription factors.

Using a ≥ twofold change as a cutoff (Supplementary Table S2), we found 45 DEGs in the luminal A tissues that are potential genes regulated by C-JUN and/or C-FOS transcription factors (Supplementary Table S3). The expression of these genes was confirmed in 541 luminal A breast cancer patients from TCGA-BC and 178 healthy breast tissue samples from the GTEX dataset (Supplementary Fig. S2).

Five of these genes were tested for AP1 regulation (*DNAJC10, INHABA, NRIP1, YTHDF3* and *ZBTB6*). For this, 2 kb of the promoters of each chosen gene were analyzed in silico, and the predicted sites were tested for AP1 direct binding.

We identified two consensus binding sites in the *NRIP1* gene, five in the *ZBTB6* gene, two in the *DNAJC10* gene and one consensus binding site in the *INHBA* and in the *YTHDF-3* genes.

**Figure 3:**
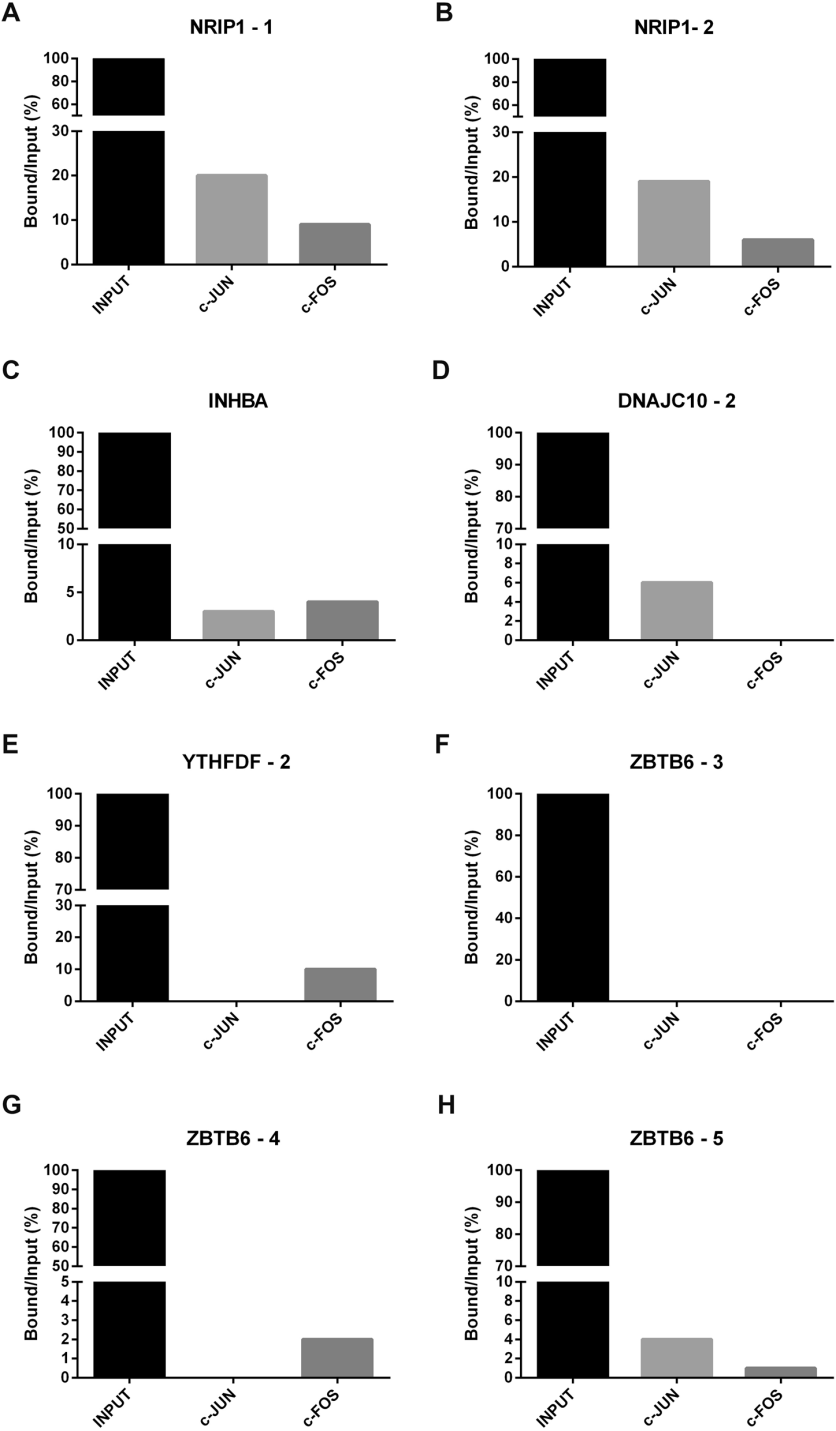
Chromatin immunoprecipitation assay (ChiP) with C-JUN and C-FOS followed by RT-qPCR of the predicted AP-1 binding sites in the *DNAJC10, INHABA, NRIP1, YTHDF-3 and ZBTB6* gene promoters. Using the C-JUN antibody, we observed specific binding to *NRIP1* at the consensus binding sites from AP-1 (**A,B**), consensus site from *INHABA* (**C**), consensus site 2 from *DNAJC10* (**D**) and consensus binding site 5 from the *ZBTB6* gene promoter (**H**). Using the C-FOS antibody, we also observed specific binding to NRIP1 at the consensus binding sites from AP-1 (**A,B**), consensus site from *INHABA* (**C**), consensus site from *YTHDF-3* (**E**) and consensus binding sites 4 and 5 from the *ZBTB6* gene promoter (**G,H**). No binding was observed at consensus binding site 3 from the *ZBTB6* gene promoter (**F**). The bar graphs show the fold-change at each site compared to the binding of the input control.

As shown in Fig. 3, our results showed several specific bindings of C-JUN and C-FOS at consensus binding sites for these genes. However, binding of both C-JUN and C-FOS observed in the two consensus binding sites was only observed for the *NRIP1* gene promoter.

**Figure 4:**
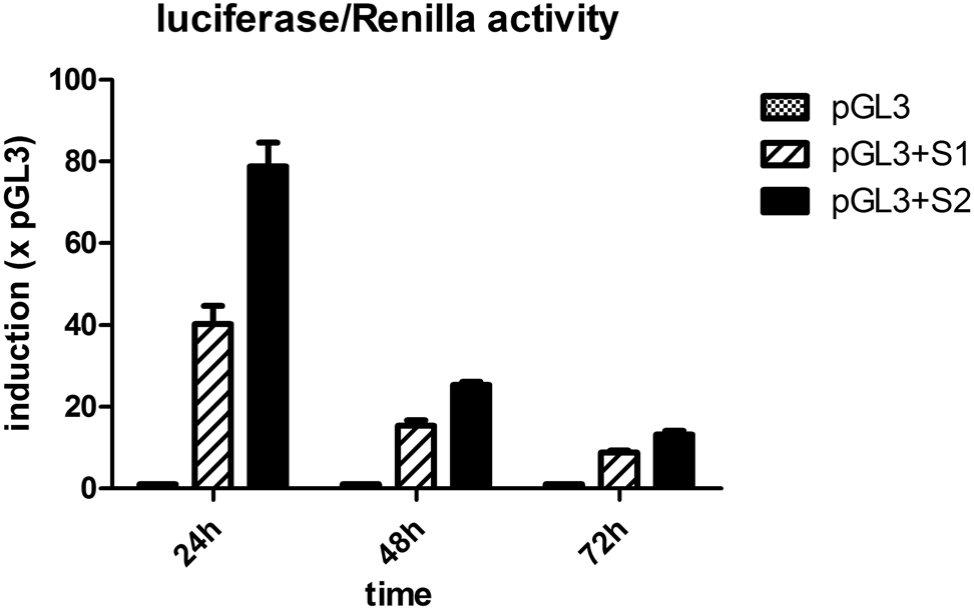
Relative luciferase activity of MCF-7 cells transfected with the pGL3-Basic plasmid containing promoter regions of the NRIP1 gene. Firefly luciferase was normalized to the Renilla vector (pRL-TK Renilla plasmid). The samples were collected 24 h, 48 h and 72 h after transfection. The values were compared to pGL3 (Mock), which was used as a negative control. pGL3-S1 contains proximal and distal sites, and pGL3-S2 contains only proximal sites. After 24 h, the S2 region showed twice the activity compared to the S1 region, which spans distal and proximal regions. After 48 h and 72 h, similar behavior was observed but with less intensity. Each bar represents the mean ± SD. *p < 0.05, **p < 0.01.

These results suggest that the *NRIP1* gene is a potential gene regulated by AP1. We next performed transient transfection assays using the MCF7 cell line with constructs containing two (S1) or one AP-1 (S2) consensus binding site, and luciferase activity was measured using a luciferase assay approach. As shown in Fig. 4, both promoter regions caused an upregulation of luciferase activity, mainly in the proximal region (S2).

### *NRIP1* gene and its target genes are altered in luminal A breast cancer patients

**Figure 5:**
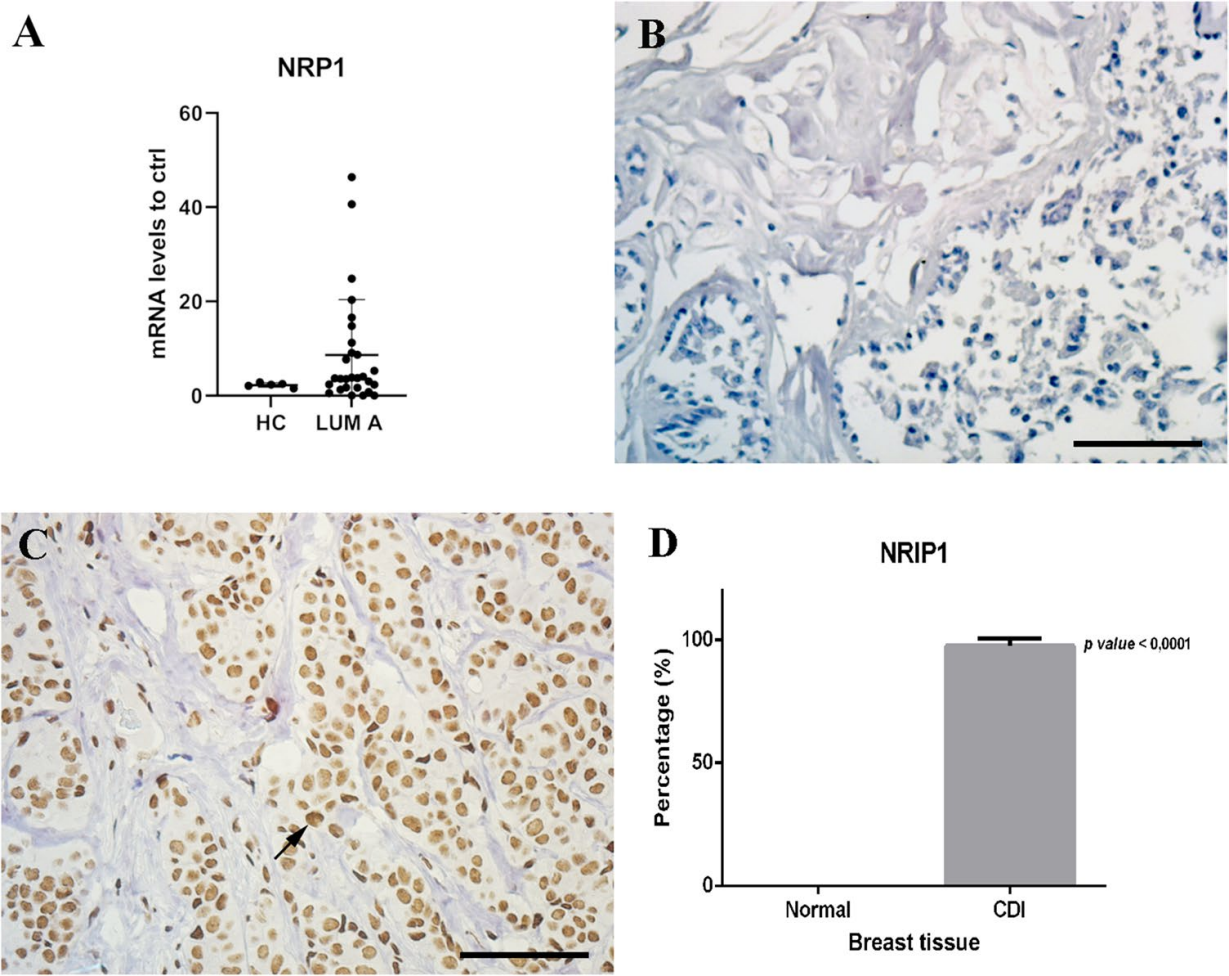
NRIP1 expression is increased in luminal A breast cancer patients. (**A**) To confirm NRIP1 expression, we used RT-qPCR to determine changes in NRIP1 mRNA expression levels in luminal A patients and healthy donors. (**B,C**) Immunohistochemistry assay showed that NRIP1 expression is increased in luminal A patients (**C**) compared to mammoplasty tissue (**B**). (**D**) Graphical representation of NRIP1. Statistical analyses showed increased NRIP1 expression in luminal A tissue compared to that in normal breast tissue (p-value < 0.0001). Scale bar: 100 µm; magnification: × 400. The arrow indicates positive labeling.

The *NRIP1* gene was overexpressed in luminal A tissues in the chip array and TGCA dataset (Supplementary Table S2, Supplementary Fig. S2, respectively). To validate this expression, we performed RT-qPCR with a larger number of patients and healthy donors (Fig. 5A). Moreover, through immunohistochemistry assays, we demonstrated an increase of the NRP1 protein in all Luminal A patients (100.0%) (Fig. 5B,C). None of the healthy donors presented NRIP1 nuclear labeling, suggesting increased nuclear protein expression (Fig. 5D).

Next, an in silico analysis showed that genes related to breast cancer, including *HES1, MYC, CCND1, FOS* and *PGR,* could be regulated by *NRIP1* (data not shown).

**Figure 6:**
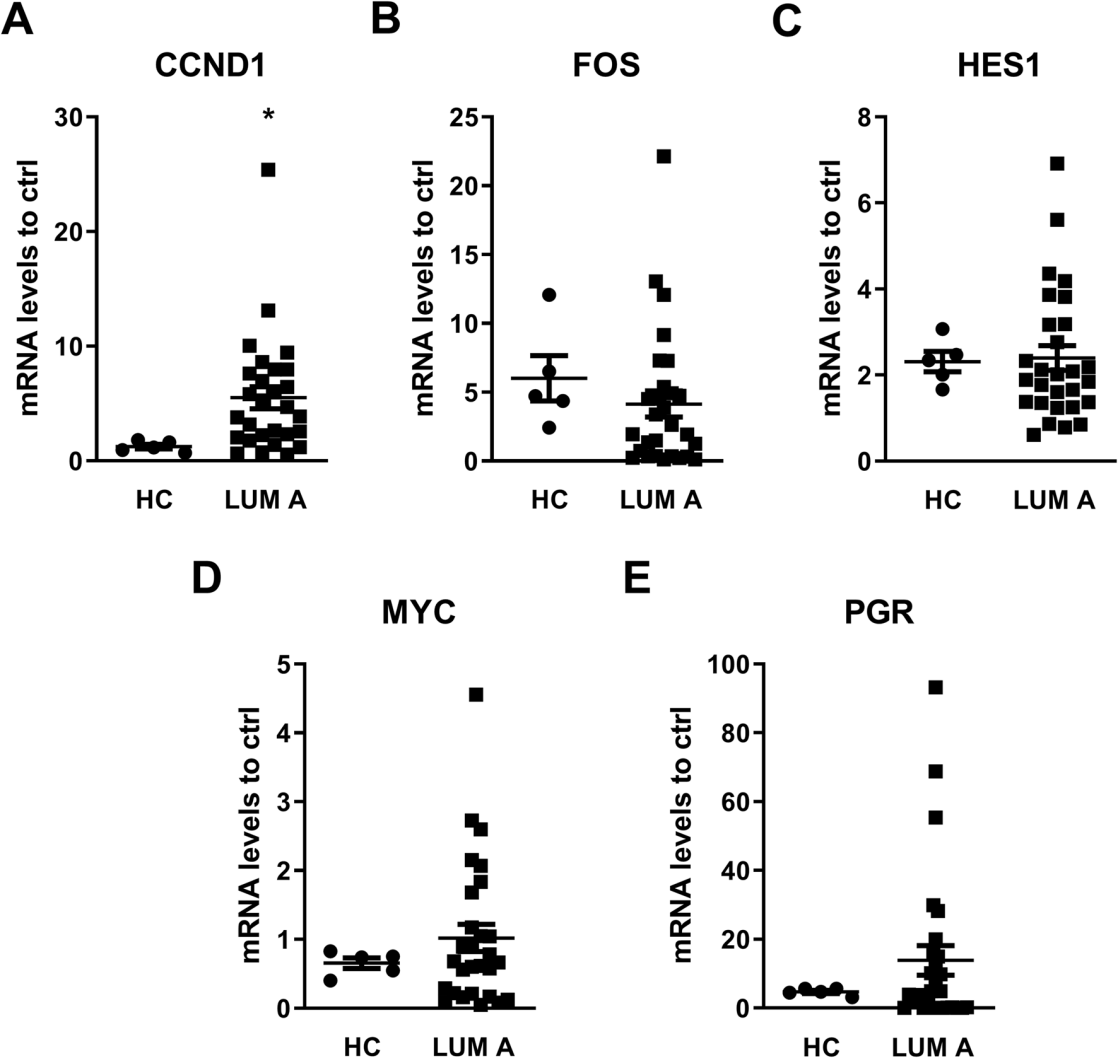
RT-qPCR of *NRIP1* target genes. To determine the changes in the mRNA levels of the *NRIP1* target genes in luminal A patients in comparison with healthy donors, RT-qPCR assays were performed. The endogenous gene *GAPDH* was used for data normalization. The mRNA levels of *CCND1* (**A**)*, FOS* (**B**), *HES1* (**C**)*, MYC* (**D**) and *PGR* (**E**) are differentially expressed in luminal A patients. The bars indicate the mean mRNA levels (± standard deviation). *p < 0.01.

As shown in Fig. 6, the mRNA levels of *HES1, MYC, CCND1,* and *PGR* were increased in the luminal A patients compared with those in the healthy donors, indicating that *NRIP1* could be important for the regulation of these genes.

**Figure 7:**
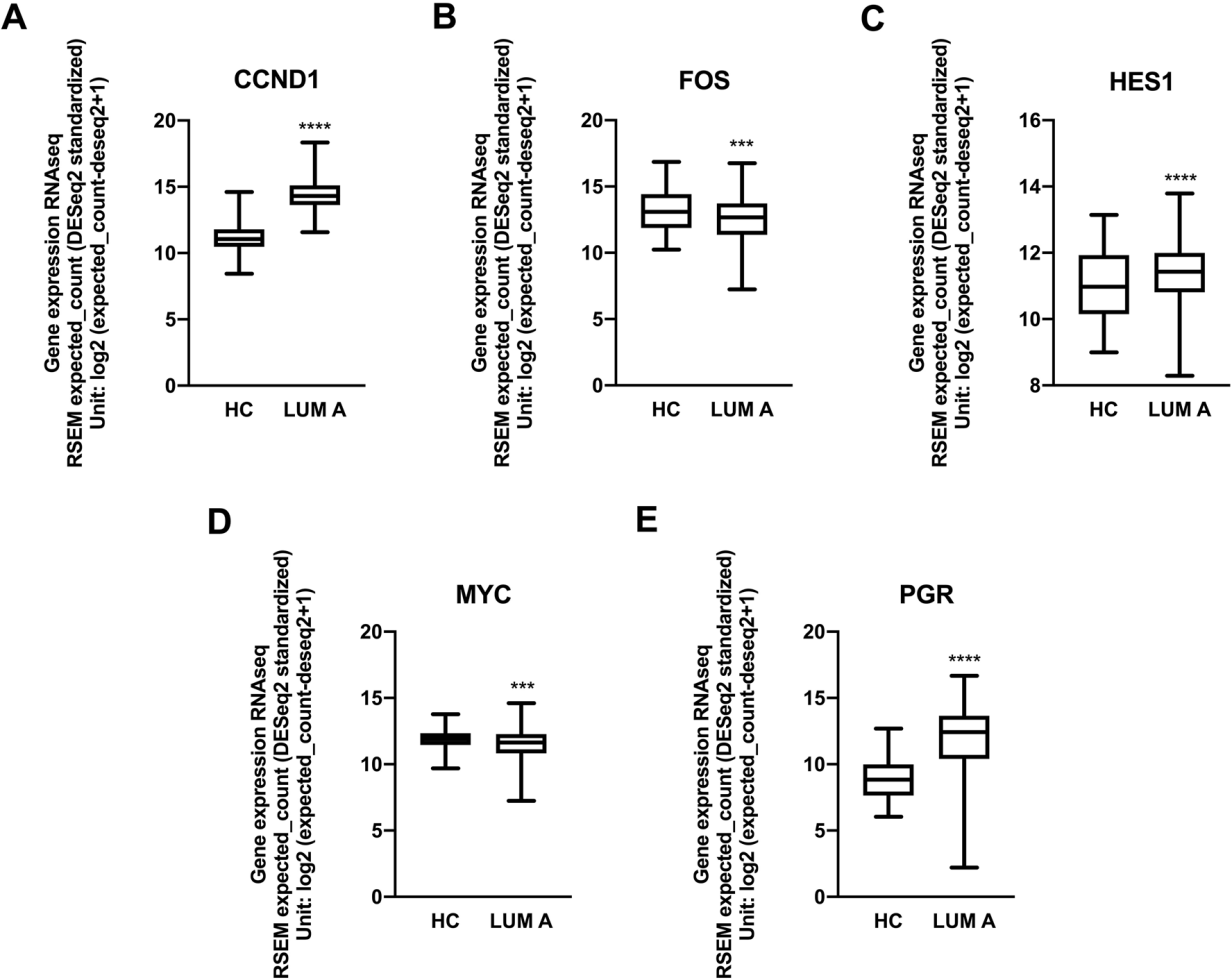
mRNA expression from TCGA-BC and GTEX datasets confirmed increased expression of *NRIP1* target genes. Box plot analysis of the *NRIP1* target genes *CCND1, FOS*, *HES1, MYC* and *PGR* from the GTCGA-BC/GTEX dataset. The mRNA levels of *CCND1* (**A**)*, HES1* (**C**)*, MYC* (**D**) and *PGR* (**E**) were increased in the luminal A patients, thus corroborating the RT-PCR results, while the mRNA levels of *FOS* (**B**) were decreased. The bars indicate the mean mRNA levels (± standard deviation). ***p < 0.001, ****p < 0.0001.

Using the same TCGA and GTEX datasets, the expression profiles of the same genes were investigated and as we can observed in Fig. 7 the expression of these genes corroborating with our results.

### NRIP1 alters the expression of important genes in the transformation of luminal A ductal cells

To verify the impact of the NRIP1 gene on luminal A breast cancer gene expression, we performed a functional analysis of *NRIP1* depletion in the MCF7 cell line using an siRNA approach. We verified the depletion 24, 48 and 72 h after transfection, and at 24 h, the mRNA levels were depleted by 80% compared with the scrambled control.

Using mRNA levels from 24 h and considering a ≥ 1.5-fold change as the cutoff to define overexpression or downregulation in an expression chip array assay, we identified 762 differentially expressed genes related to NRIP1 silencing (Supplementary Table S4), including the *PGR, ESR* and *CCDN1* genes*.*

**Figure 8:**
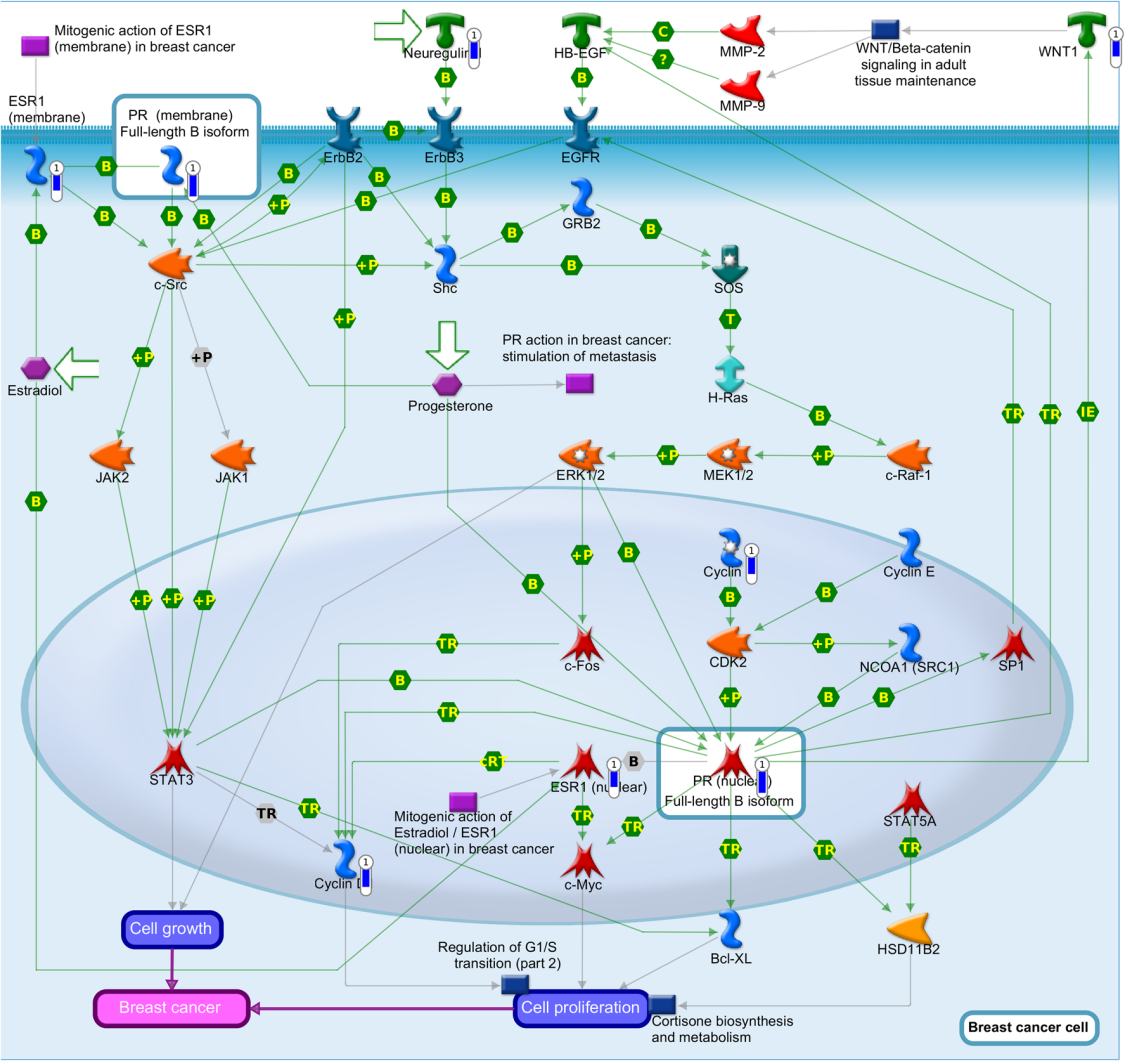
In silico analysis using MetaCore software. The pathway “PR action in breast cancer stimulation of cell growth and proliferation” was associated with differentially expressed genes related to NRIP1 silencing. Downregulated genes are marked with blue thermometer.

The in silico analysis of the signaling pathways that could be associated with the differentially expressed genes was performed using MetaCore software (Clarivate Analytics, USA), and it revealed that the main pathway related to NRIP1 silencing was “PR action in breast cancer-stimulation of cell growth and proliferation” (Fig. 8). The *PGR*, *ESR1* and *CCND1* genes were downregulated when NRIP1 was silenced, indicating the possible role of NRIP1 in breast cancer development.

To corroborate with these results, we also applied siRNA approach to deplete *NRIP1* using another Luminal A cell line (T47D). We also verified the depletion 24, 48 and 72 h after transfection, and 24 h was the depletion chosen time. To verify if *PGR*, *ESR1* and *CCND1* genes expression were also downregulated in this cell line when NRIP1 was silenced, we performed RT-qPCR (Supplementary Fig. S3). This result is in accordance with that obtained with MCF7 cell line, further reinforcing the possible role of NRIP1 in breast cancer development.

## Discussion

Breast cancer is a heterogeneous and complex disease, and the treatment decisions and prognosis are determined according to the different subtypes, which are based on histopathological, biological, and molecular characteristics^9,10^.

Although breast cancer is a highly studied tumor, most studies are related to the classification, treatment and progression of the disease to metastasis. Studies investigating events related to its initiation and initial progression to an invasive tumor are scarce. The transition of DCIS to invasive breast cancer has recently been studied at the molecular level and showed a gene expression signature that could predict DCIS progression^11^. However, the molecular mechanisms responsible for breast tumor initiation and the overall mechanisms that lead healthy cells to transform into luminal A tumors remain poorly understood.

Results of DEGS analysis indicates C-JUN and C-FOS as potential regulators. These genes have shown before important roles in the regulation of several cellular processes implicated in cancer acting in dimers called AP1. The *Jun* and *Fos* subfamilies are the most important AP-1 proteins that bind TPA responsive elements (TREs)^12^.

Some studies evaluated the expression of AP-1 family components from primary breast tumors from patients with invasive ductal and lobular carcinomas with adjacent nontumor tissue^13^. In our study, while the mRNA levels of C-JUN and C-FOS did not differ between the tumor and normal tissues, the C-JUN and C-FOS proteins were not only increased in expression but also activated in the luminal A patients, indicating a potential regulatory role. To test this hypothesis, we combined several experimental approaches to identify *NRIP1* as one of the regulated genes.

Nuclear receptor interacting protein 1 (*NRIP1*) is a coregulator of several nuclear receptors and transcription factors that can act as a coactivator or a corepressor. *NRIP1* is essential for normal mammary gland development and functions as a component in estrogen signaling^14–17^.

In the mammary gland, *NRIP1* modulates the expression of several ER target genes, such as *AREG*, *PGR*, *CCND1* and *STAT5a*, thus acting as a coregulator with ER. When *NRIP1* expression is ablated, the expression of *AREG* and *PGR* is lost, which leads to developmental defects^17,18^.

*NRIP1* has shown to be related to human cancers^16,18^. In BC, it was associated with estrogen receptors (ERs) and suggested to regulate proliferation and invasion^15^. Moreover, it was associated with the risk of breast cancer^16^.

*NRIP1* expression was found to be elevated in ductal carcinomas in situ^14^ and overexpressed in human breast cancer tissue, and its expression in BC cell lines was elevated compared with that in MCF10A^16^, suggesting that *NRIP1* is indeed altered in breast cancer cells.

As *NRIP1* is an overexpressed transcriptional coregulator in breast cancer, we focused on evaluating the genes that could be regulated by *NRIP1.* To evaluate the importance of *NRIP1*, we depleted this gene in the MCF7 and T47D cell lines using an siRNA approach and performed a chip array or RT-PCR assays, respectively. The results from chip array assay using MCF7 cell line, revealed 762 differentially expressed genes related to NRIP1 silencing, including the *PGR, ESR* and *CCDN1* genes*,* which we have shown to be upregulated in luminal A patients of our cohort and that of TCGA. When we depleted *NRIP1* using T47D cell line, the results reinforced our findings.

Aziz and coworkers and Yuan and coworkers showed that the inhibition of *NRIP1* expression in siRNA assay are related to apoptosis and cell growth inhibition^16,18,19^. Another group, using the same approach experiments, revealed that NRIP1 is needed to the regulatory complex required to stimulate breast cancer proliferation. Moreover, the genes that changed as a result of *NRIP1* knockdown in MCF7 cells were used to stratify patients with breast cancer who received adjuvant tamoxifen treatment^18^. Corroborating these studies, our in silico analysis of *NRIP1* silencing showed that signaling pathways related to breast cancer were altered.

Taken together, our results indicate that in the development of breast cancer, *NRIP1* is targeted by C-JUN and C-FOS in luminal A cells and plays a role in ductal cell transformation by regulating genes related to the disease. Thus, *NRIP1* is a target for future breast cancer therapy.

## Methods

### Study design

All patients included in this study were female (mean age: 56; age range 30–80 years) and were diagnosed with molecular subtype luminal A breast cancer, and samples were collected before the first chemotherapeutic regimen. Clinicopathological data were obtained from medical records (Table 1). These patients were stratified into four cohorts Microarray cohort (n = 4), Western Blot cohort (n = 4), RT-qPCR cohort (n = 28) and Immunohistochemistry cohort (n = 25) (Table 1). As control, healthy breast tissue was kindly provided by women undergoing reduction mammoplasty attended at Hospital Universitário de Londrina (mean age 51.2; age range 46–56 years). The study was designed and conducted in accordance with the ethical principles for medical research involving human subjects from the Declaration of Helsinki. This study was approved by the National Ethics Committee (Conselho Nacional de Ética em Pesquisa—CONEP) and the local Institutional Committee (Conselho de Ética em Pesquisa da Universidade Estadual do Oeste do Paraná—CAAE number 35524814.4.0000.0107). Experimental procedures were approved by the institutional board and all participants signed informed consent forms.

**Table 1:** Clinicopathological characteristics of the patients from all cohorts.

Variable	Total	Microarray cohort	WB cohort	RT-qPCR cohort	IHC cohort
Total number of patients	53	4	4	28	25
**Age at diagnosis (range, years)**	56 (30–80)	61.75 (57–68)	61.75 (57–68)	53.7 (30–76)	58.56 (30–80)
≤ 45	12	0	0	6	6
> 45	41	4	4	22	19
**Histological grade**					
I/II	49	4	4	25	24
III/IV	4	0	0	3	1
Infiltrative ductal carcinoma	53	4	4	28	25
**IHC molecular status**					
Positive ER	53	4	4	28	25
Positive PR	47	4	4	23	24
Positive HER2	0	0	0	0	0
Lymphonodal invasion	1	0	0	1	0

*IHC* immunohistochemistry, *ER* estrogen receptor, *PR* progesterone receptor, *HER2* human epidermal growth receptor 2, *WB* Western blot, *RT-qPCR* Real-time polymerase chain reaction.

### Expression chip array data analysis

Total RNA was obtained using a RNeasy Mini Kit (Qiagen, CA, USA) according to the manufacturer’s instructions. For the chip array assay, the RNA was processed, hybridized to a GeneChip Human Exon 1.0 ST Array (Affymetrix, CA, USA), washed, stained and scanned as previously described^20^. Partek software was used for data analysis^21^ and MetaCore software was used to evaluate pathway analysis and related processes. (Clarivate Analytics, USA).

All data have been deposited in the NCBI’s Gene Expression Omnibus (GEO) (GSE58102 and GSE180835).

### Western blot analysis

Protein extracts from luminal A patients and healthy breast tissues were obtained as previously described^22^. They (30 μg) were run on sodium dodecyl sulfate polyacrylamide gels (SDS-PAGE), transferred to nitrocellulose membranes (Bio-Rad, CA, USA) and incubated with anti-C-JUN (sc-1694) and anti-C-FOS (sc-7202) antibodies (Santa Cruz Biotechnology, TX, USA) and phosphorylated anti-C-JUN (ab32385) and C-FOS (ab27793) (Abcam, UK) antibodies. The detection of antibody binding was performed using Enhanced chemiluminescence Pierce Plus Western Blotting Substrate (Thermo Fisher Scientific, MA, USA) and normalization was accomplished by Rouge Ponceau staining.

### Cell lines

The MCF-7 (authenticated by the cell bank of Rio de Janeiro—BCRJ) and T47D (acquired from ATCC) Luminal A cell lines were grown in RPMI medium (GIBCO Life Technologies, Carlsbad, CA, USA)^23^. The control cell line HMEC (#A10565-acquired from Thermo Fisher Scientific, MA, USA) was grown in HuMEC medium. All cell lines were supplemented with 10% fetal bovine serum (FBS, HyClone, USA), 100 IU/mL penicillin, 100 µg/mL streptomycin (Invitrogen, CA, USA), and 2 mM l-glutamine (Invitrogen, CA, USA).

### Chip-on-chip assay

To identify the promoter targets of C-JUN and C-FOS, we used SimpleChIP Enzymatic Chromatin IP kit (Magnetic Beads) to perform chromatin immunoprecipitation (ChIP) assays according to the manufacturer’s instructions (Cell Signaling Technology, MA, USA), followed by a promoter chip array assay (Affymetrix, CA, USA). Briefly, chromatin from the MCF7 and HMEC cell lines, which was previously prepared and digested with *micrococcal nuclease,* was incubated with 2 μg of C-JUN antibody, C-FOS antibody (Santa Cruz Biotechnology, TX, USA) or the negative immunoprecipitation control normal anti-IgG rabbit antibody (#2729, Cell Signaling Technology, MA, USA). Then, the immunoprecipitated DNA was purified according to the manufacturer's instructions (Affymetrix CA, USA) and subsequently hybridized to a *Human Promoter 1.0R Array* (Affymetrix, CA, USA). After that, the arrays were washed, stained and scanned according to the manufacturer’s protocols.

### Promoter array analysis

The binding sites of C-FOS and C-JUN were identified using Tiling Analysis Software (TAS) version 1.1. Quantile normalization of the probe intensity data was applied to the enriched samples and the controls of both cell types. The probe analysis was conducted with a bandwidth of 500 bp, and the interval analysis selected promoter regions of at least 200 bp in length and a maximum gap of 50 bp between significantly enriched probes (pvalue < 0.01). These regions were subsequently matched with the promoter library of NCBI v.36 Human genome using BEDTools intersect program, which revealed the genes associated with the promoter regions.

### Prediction of C-JUN and C-FOS binding sites

To screen for putative C-JUN and C-FOS consensus binding sites, 2 kb upstream of the transcription start site from the selected genes was obtained from the NCBI database. The consensus binding sites were obtained using online prediction tools. The online software programs used were TRANSFAC (http://www.gene-regulation.com) and Genomatix (www.genomatix.de).

### Real-time polymerase chain reaction (RT-qPCR) analysis

To evaluate the putative C-JUN and C-FOS binding sites in the selected genes, purified DNA from the MCF7 cell lines after the ChIP assays with C-JUN, C-FOS or normal anti-IgG was used in RT-qPCR assays with specific primers (Supplementary Table S1) for each putative binding site. Rotor-Gene 6000 thermocycler (Qiagen, NWR, Germany) was used to perform the reactions using the following program: 95 °C for 10 min; 40 cycles at 95 °C for 20 s and 60 °C for 30 s; and a final extension at 72 °C for 30 s. The changes in C-FOS or C-JUN binding to DNA were calculated in relation to that in the IgG precipitated control. All samples were normalized to the input.

### Luciferase reporter assay

The promoter regions of the *NRP1* gene were amplified from the MCF7 cell line DNA by PCR using the following oligonucleotides: 5′-GCGCATTAGCAACTTCATTTC-3′ and 5′-GAGA ACCCGGAGACTCGAAC-3′ for the S1 (− 1586) region and 5′-GGAGCGTTGAGGATACGATT-3′ and 5′-GAGAACCCGGAGACTCGAAC-3′ for the S2 (− 1271) region. Each PCR amplicon was inserted into a PCR 2.1 TOPO plasmid (Invitrogen CA, USA). These resulting plasmids were digested with KpnI and XhoI (Promega, WI, USA) for the S1 region and HindII and XhoI (Promega, WI, USA) for the S2 region, and the promoter regions were cloned in the pGL3-Basic Vector (Promega, WI, USA) with DNA ligase (Invitrogen, CA, USA). Approximately 0.2 µg of each plasmid, including pGL3-Basic (without insert), was cotransfected along with 0.2 µg of pRL-TK Renilla plasmid into MCF-7 cells using Lipofectamine LTX with Plus Reagent (Thermo Fisher Scientific, MA, USA). The cells were harvested after 24 h, 48 h or 72 h of transfection, and Dual-Luciferase Reporter Assay System (Promega, WI, USA) on a Veritas Microplate Luminometer (Turner BioSystems) was used to quantify the luciferase activity. The promoter activity was evaluated by the firefly luciferase activity normalized to the Renilla vector compared to the pGL3 (Mock) signal.

### RT-qPCR analysis of the NRIP1 gene and its targets

To extract the total RNA from luminal A and healthy breast tissues and MCF7, T47D and HMEC cell lines we used TRIzol reagent (Invitrogen, CA, USA) according to the manufacturer’s instructions. To perform RT-qPCR analyses two micrograms of mRNA treated with amplification-grade DNase I was used (Invitrogen, CA, USA) and reverse transcribed with SuperScript III Reverse transcriptase (Invitrogen, CA, USA) following the manufacturer’s protocol. The reactions were performed as described above. DDCt method according to Livak and Schmittgen^24^ was used to calculate the fold change in expression. *GAPDH* was used as a normalization gene. The primers used are presented in supplementary table 1.

### Immunohistochemistry

Formalin-fixed paraffin-embedded biopsies of luminal A patients (IBC, n = 15) diagnosed from 2012 to 2018 and histologically confirmed by hematoxylin and eosin (H&E) staining were randomly chosen. Formalin-fixed paraffin-embedded normal breast tissue from mammoplasty (n = 3) was used as a procedure control. The samples were incubated with primary antibodies for 18 h at 4 °C. For the positive control, tissues determined by antibody manufacturer datasheets were used. The reaction was revealed with diaminobenzidine (DAB), followed by hematoxylin counterstaining. The negative controls were prepared with an antibody dilution solution without the primary antibody. The level of the proteins available in the control tissue and IBC biopsies was compared and analyzed with an unpaired t-test, and a Pearson test was performed to identify direct or indirect correlations.

### RNAi knockdown (siRNA)

1 × 10^5^ cell/mL of MCF-7 and T47D cells were plated in a 24-well plate and incubated overnight with RPMI-1640 media without antibiotics. NRIP1 siRNA (100 nM) (S15702, Thermo Fisher Scientific, MA, USA) and 2 μL of Lipofectamine 3000 (Thermo Fisher Scientific, MA, USA) were incubated separately in a final volume of 50 μL of RPMI-1640 media for 5 min. Subsequently, the siRNA and Lipofectamine were mixed, incubated for 30 min and then applied dropwise to the cell cultures. Scrambled siRNA (100 nM) (SC-37007, Santa Cruz) was used as a siRNA negative control. FITC-conjugated siRNA was used to evaluate the transfection efficiency by FACS. siRNA transfections were conducted for up to 72 h, and RT-qPCR analysis of the NRIP1 gene was used to evaluate the inhibition rates after 24 h, 48 and 72 h. The best inhibition rate was used to perform the ChIP array assay as described above or RT-qPCR using MCF7 or T47D cell lines, respectively. A pathway analysis and related processes were performed using MetaCore software (Clarivate Analytics, USA).

### mRNA expression data from the genotype-tissue expression project and the Cancer Genome Atlas

Healthy breast tissue samples (n = 178) from the public resource Genotype-Tissue Expression (GTEx) project and luminal A (n = 541) breast cancer (BC) patients were sampled using the UCSC Xena online tool (https://xena.ucsc.edu). The mRNA expression data were obtained by the RNA-seq strategy RSEM expected_count (DESeq standardized) dataset with DESeq normalization. This process is recommended for comparisons of tumor tissue with normal tissue. The TCGA-BC dataset was categorized according to the BC molecular subtypes by the cBioPortal online tool (https://www.cbioportal.org/) and TCGAbiolinks (R/Bioconductor package)^25^. Patients with DCIS (ductal in situ), neoadjuvant therapy, prior treatment/malignancy and inconclusive classification were further excluded from all analyses.

### Statistical analysis

All experiments were carried out in triplicate, and the data are expressed as the mean ± standard error of the mean. Unpaired Mann–Whitney test was used for all comparisons and a p-value < 0.05 was considered statistically significant. The analysis was performed using GraphPad Prism software (GraphPad Software Inc., CA, USA).

## Supplementary Information

Supplementary Information 1.

Supplementary Information 2.

Supplementary Information 3.

Supplementary Information 4.

Supplementary Legends.

Supplementary Figure S1.

Supplementary Figure S2.

Supplementary Figure S3.

Supplementary Table S1.

Supplementary Table S2.

Supplementary Table S3.

Supplementary Table S4.
