# Supplementary figures and images for: NRIP1 is activated by C-JUN/C-FOS and activates the expression of PGR, ESR1 and CCND1 in luminal A breast cancer
Source: Sci Rep. 2021 Oct 27;11:21159. doi: 10.1038/s41598-021-00291-w (PMC8551324; doi:10.1038/s41598-021-00291-w)

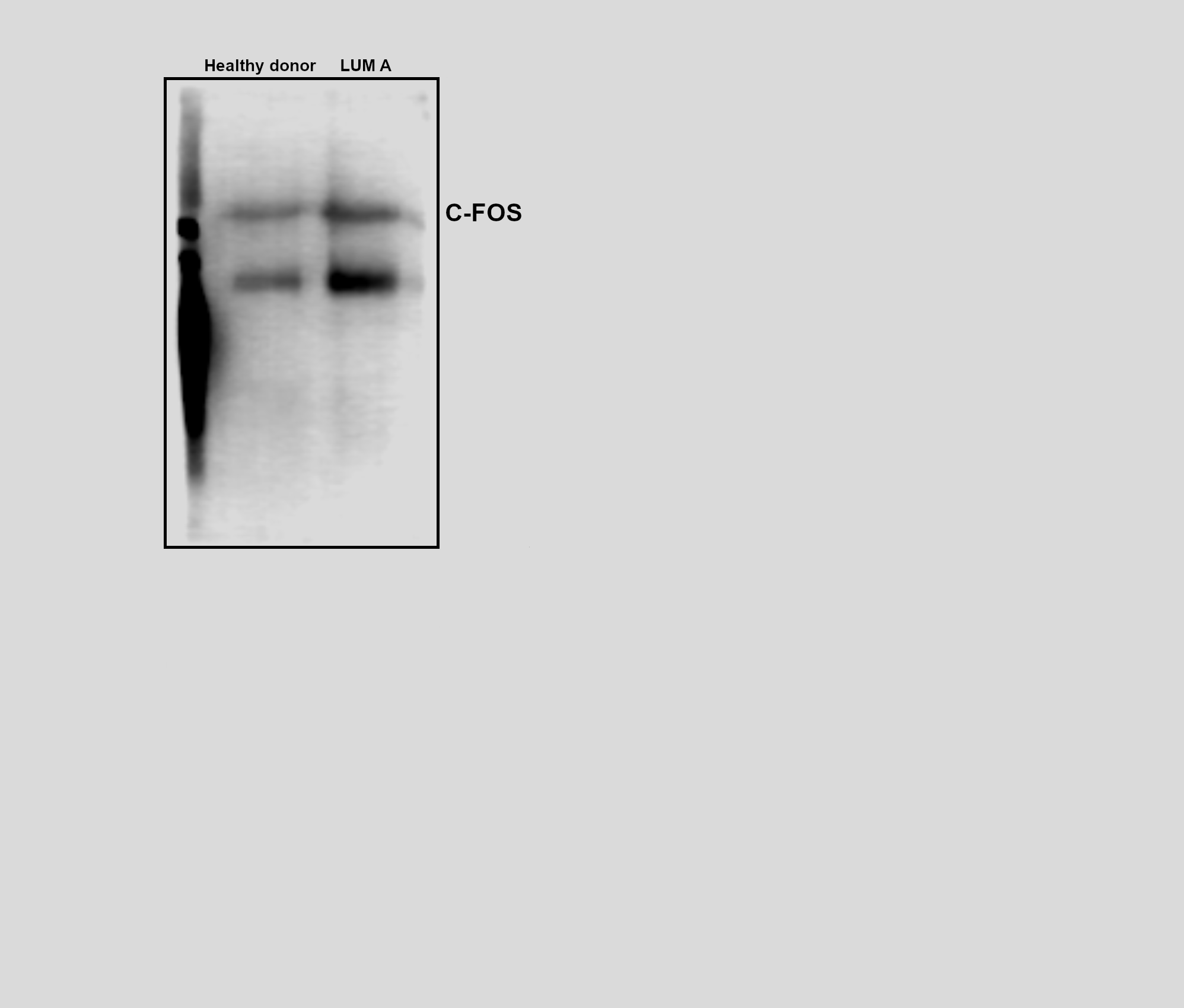

Supplement: Supplementary file 1 — Supplementary Information 1. [file 41598_2021_291_MOESM1_ESM.tif]

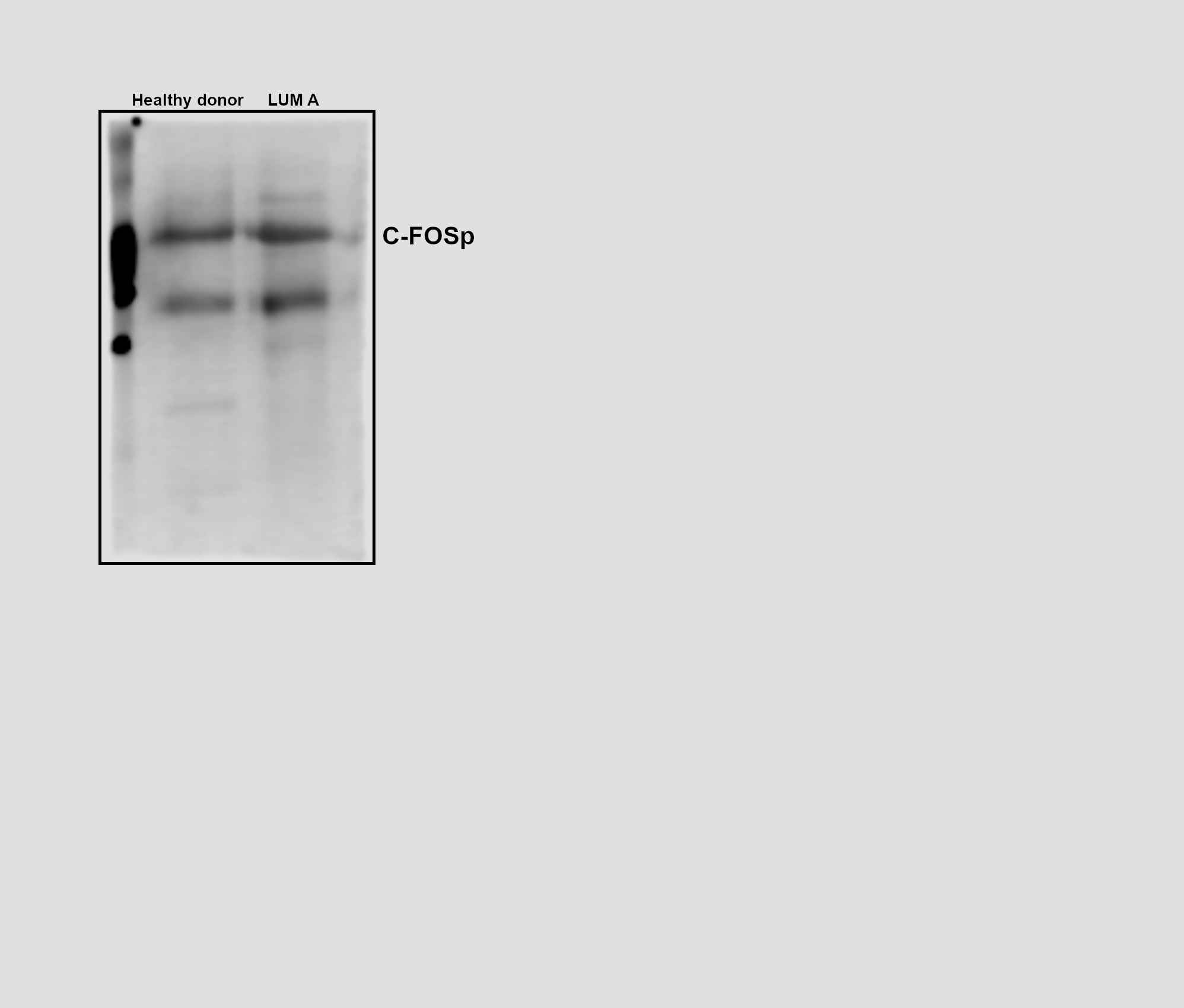

Supplement: Supplementary file 2 — Supplementary Information 2. [file 41598_2021_291_MOESM2_ESM.tif]

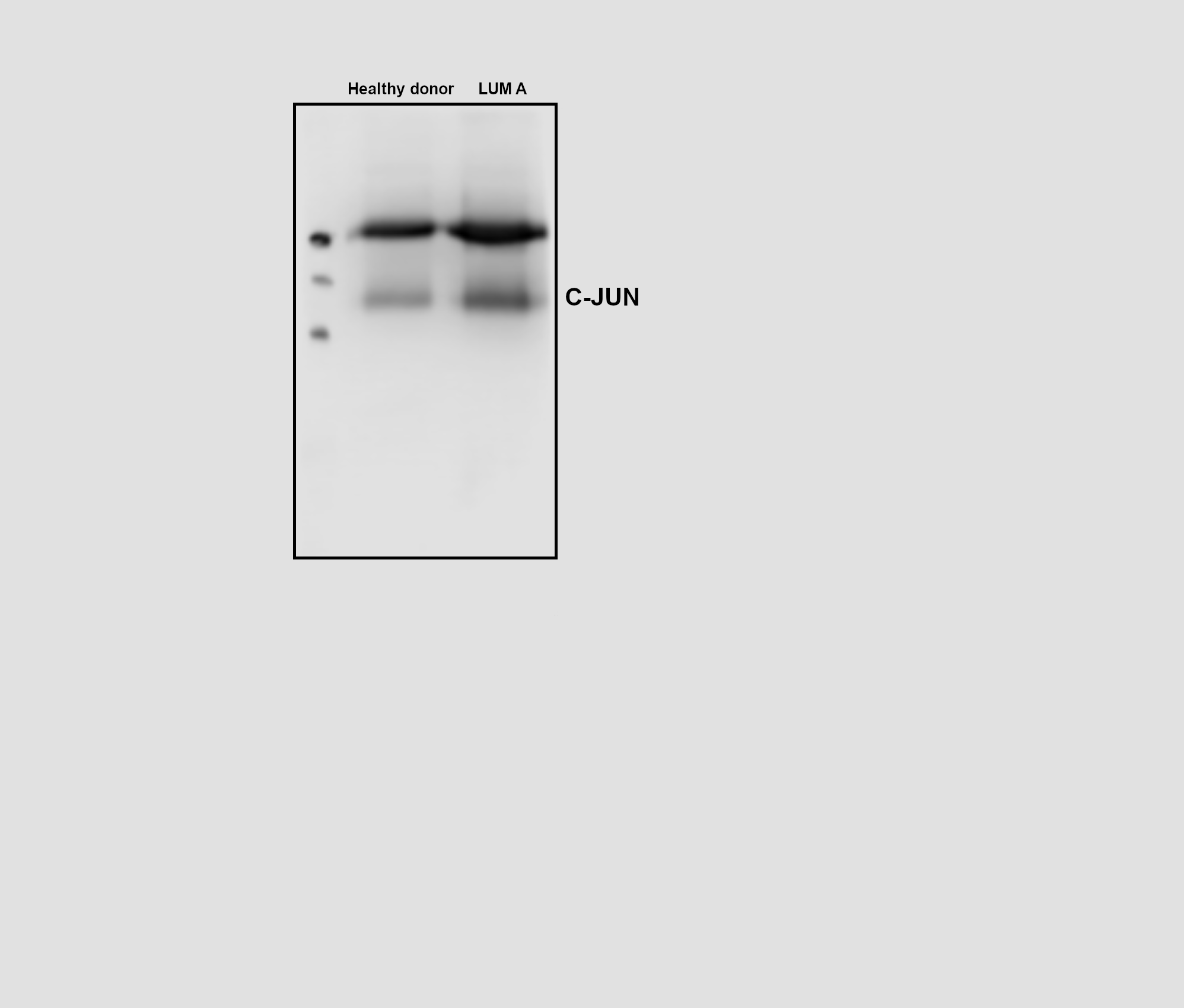

Supplement: Supplementary file 3 — Supplementary Information 3. [file 41598_2021_291_MOESM3_ESM.tif]

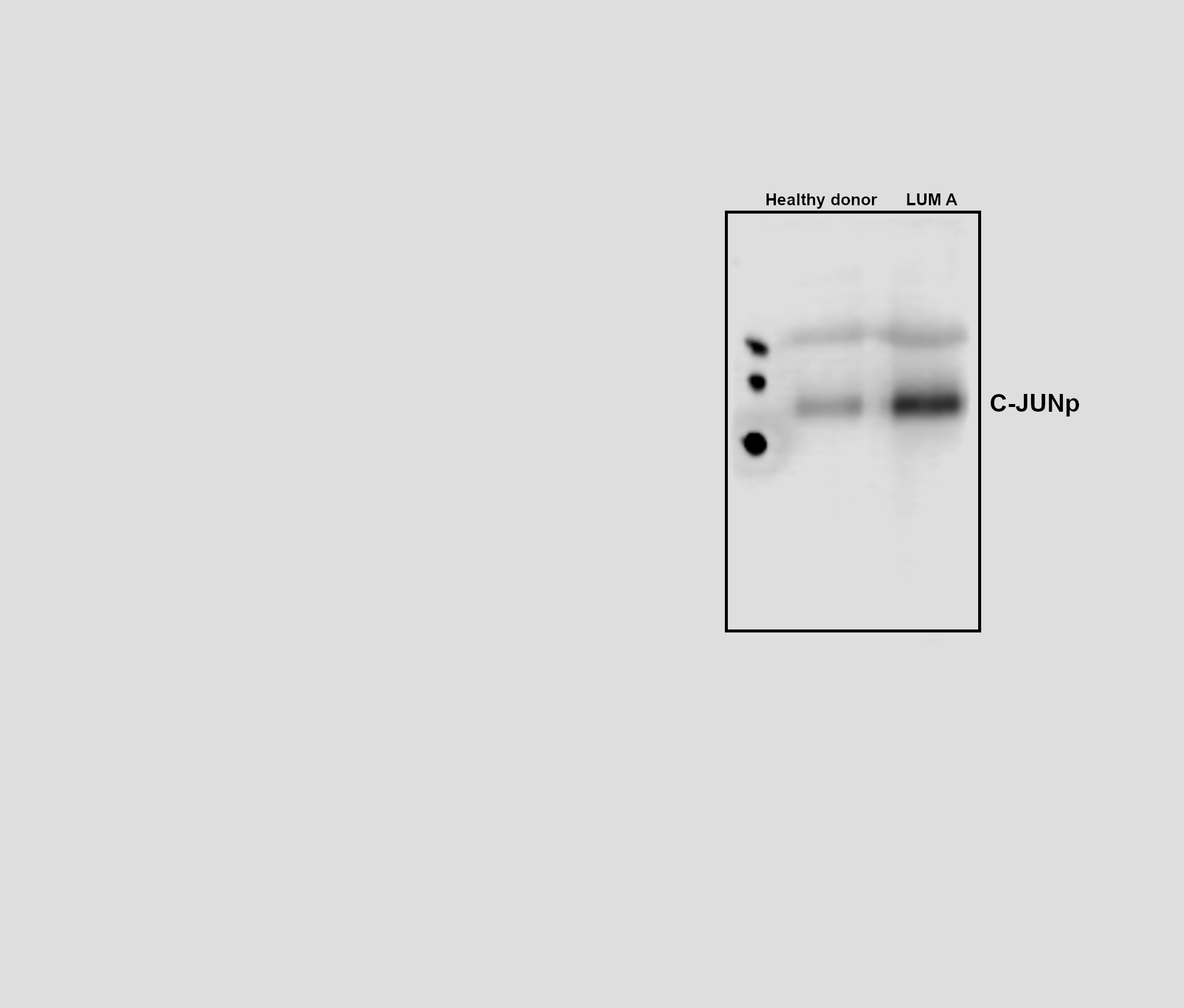

Supplement: Supplementary file 4 — Supplementary Information 4. [file 41598_2021_291_MOESM4_ESM.tif]

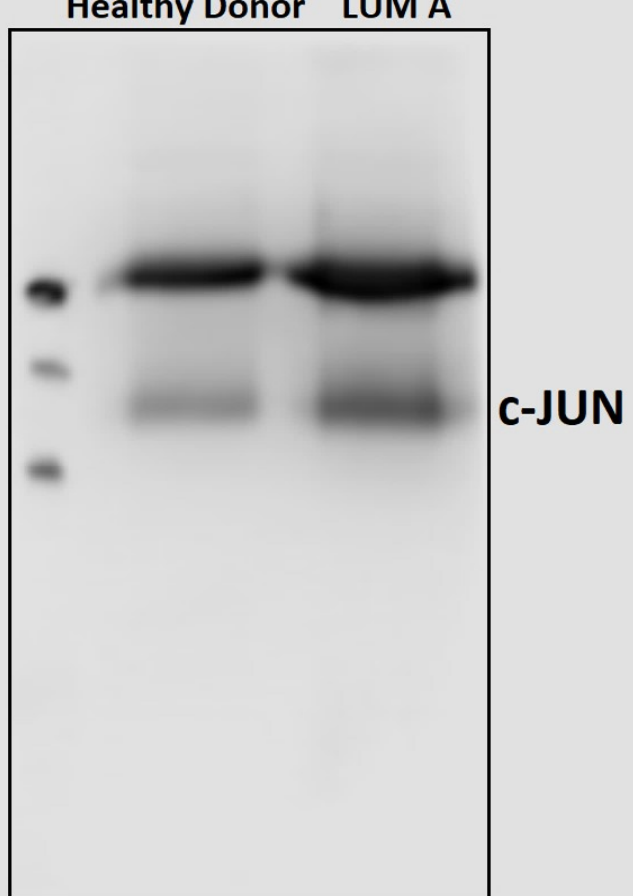

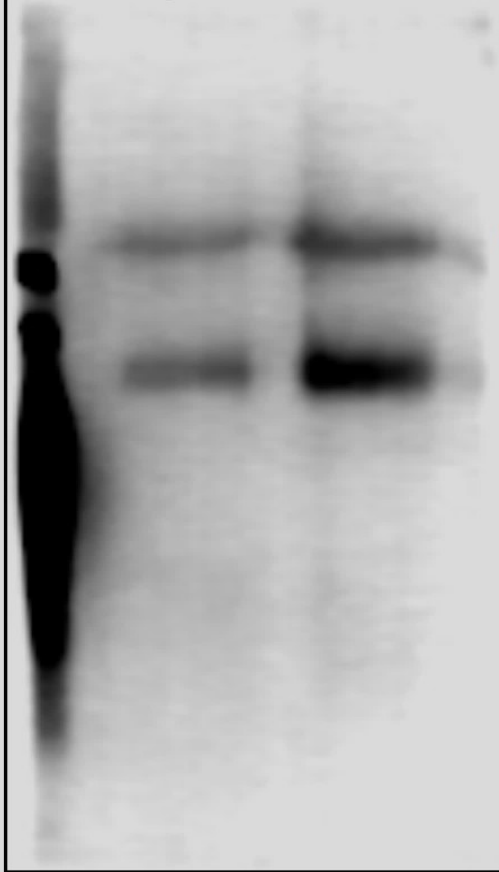

c-FOS

Healthy Donor    LUM A

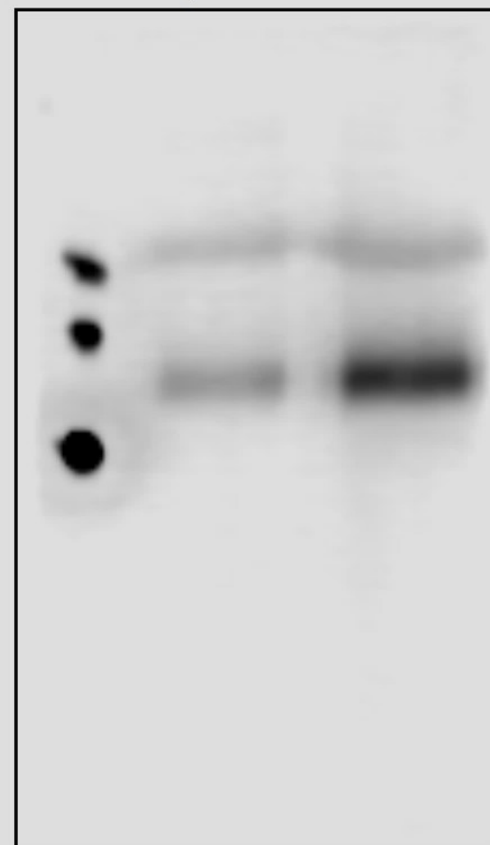

c-JUNp

Healthy Donor LUM A

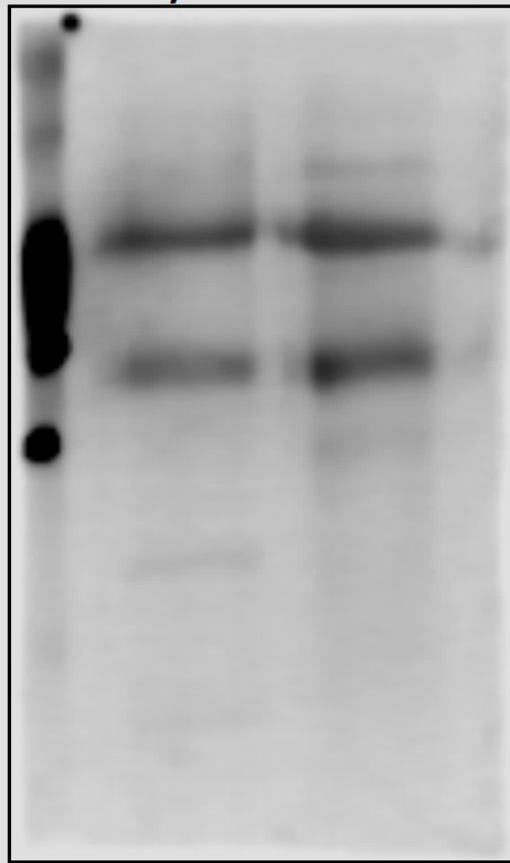

c-FOSp

Supplement: Supplementary file 6 — Supplementary Figure S1. [file 41598_2021_291_MOESM6_ESM.pdf]

A

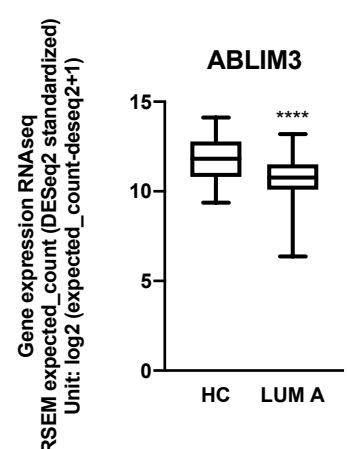

B

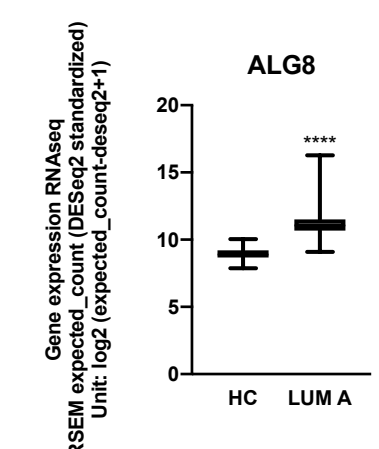

C

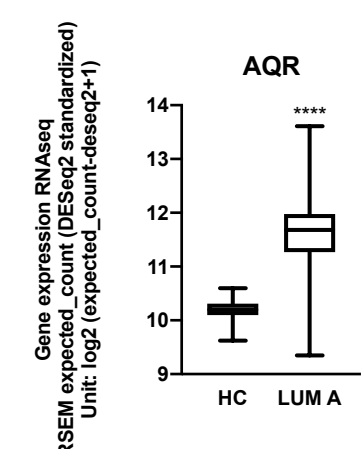

D

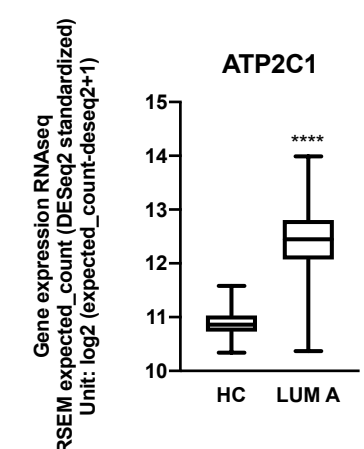

E

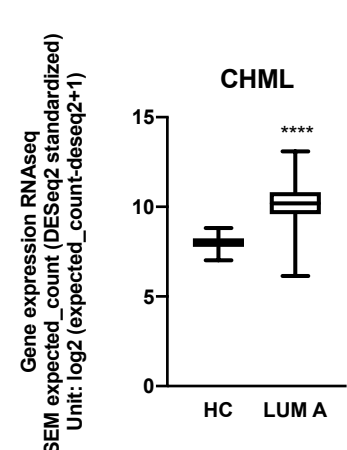

F

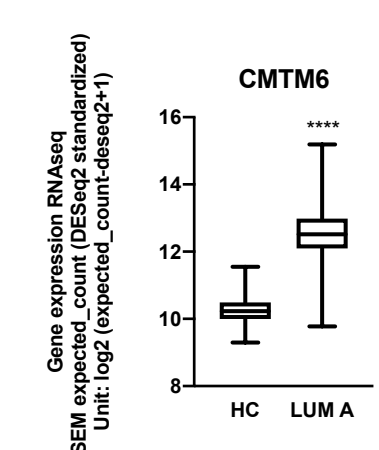

G

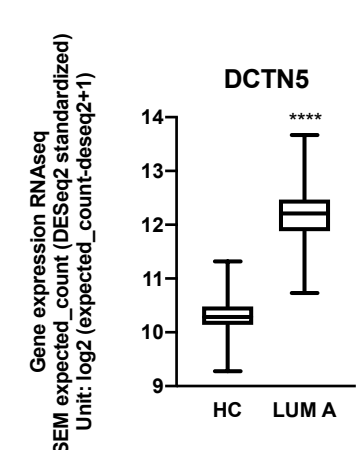

H

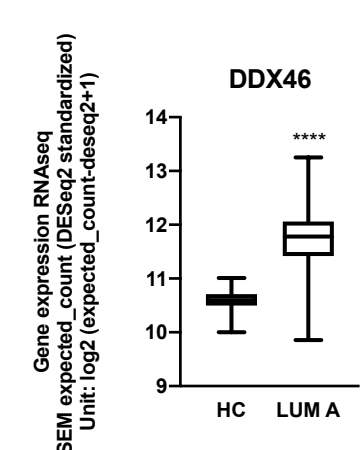

I

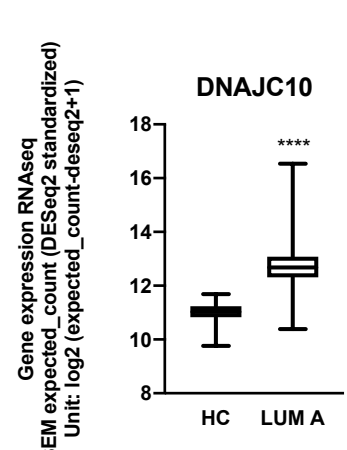

J

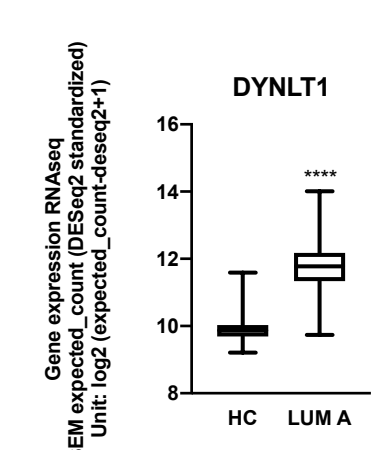

K

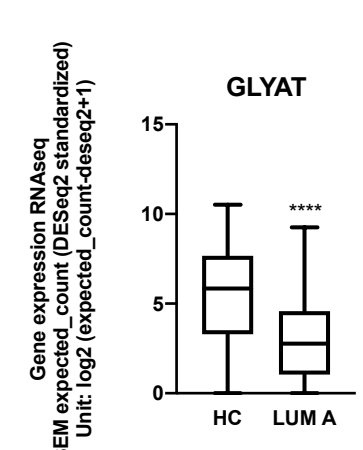

L

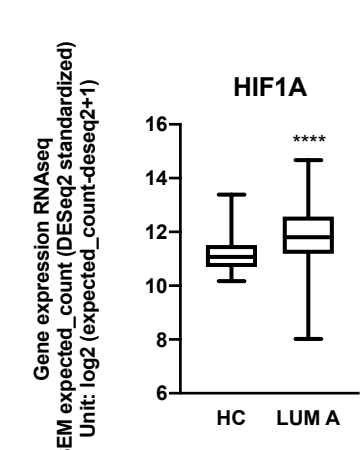

M

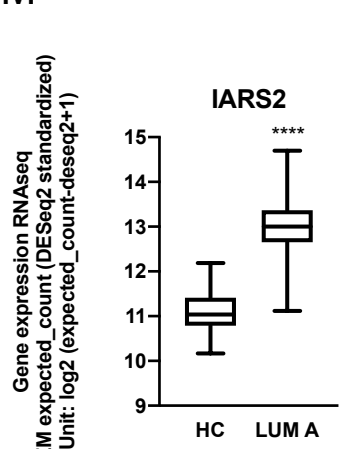

N

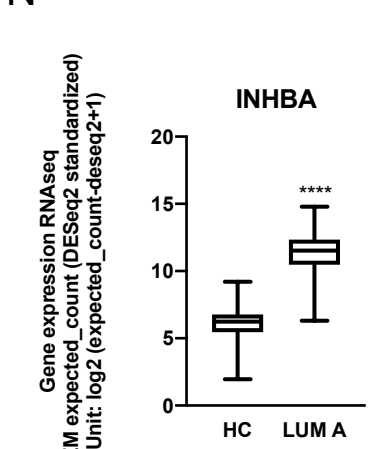

O

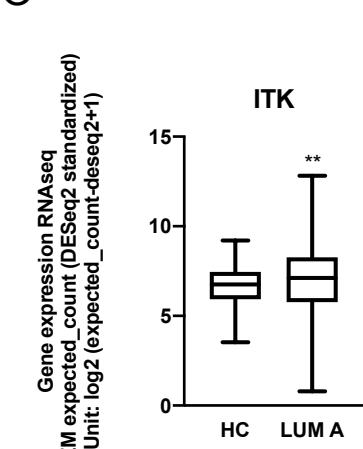

P

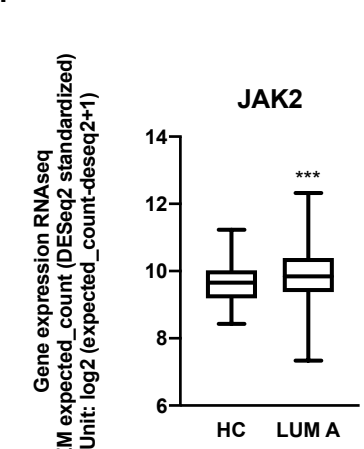

Q

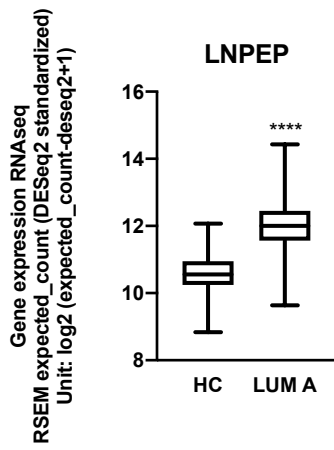

R

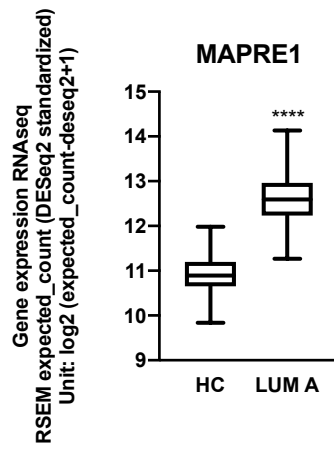

S

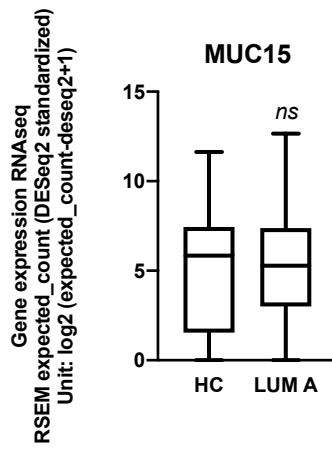

T

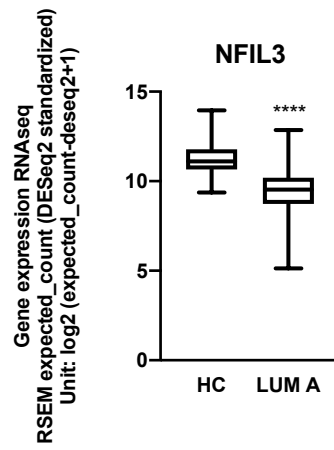

U

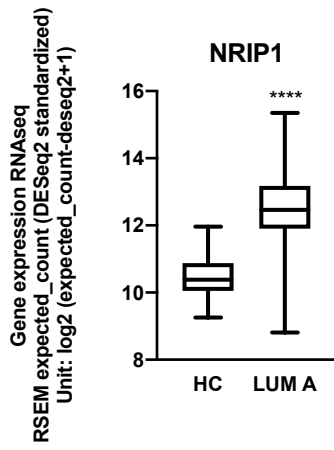

V

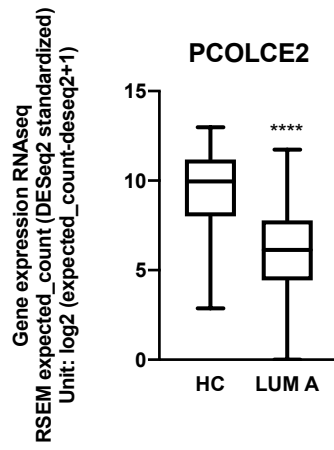

W

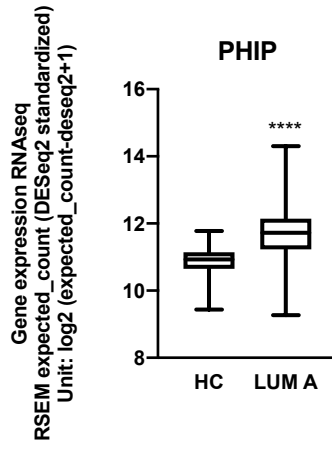

X

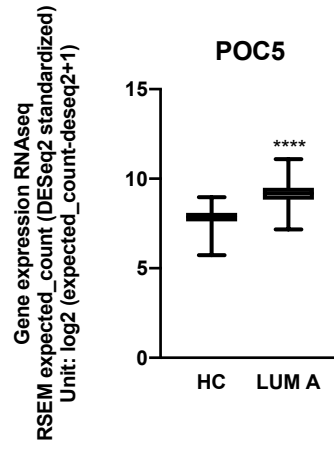

Y

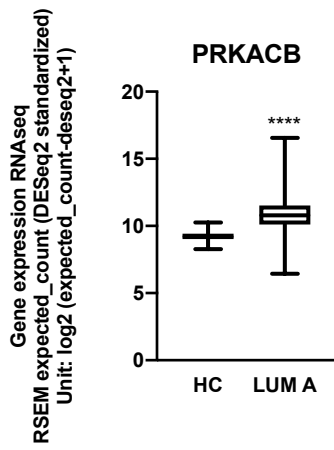

Z

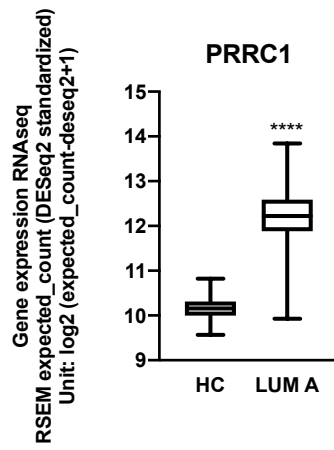

A'

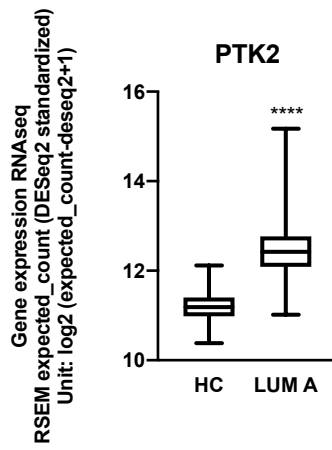

B'

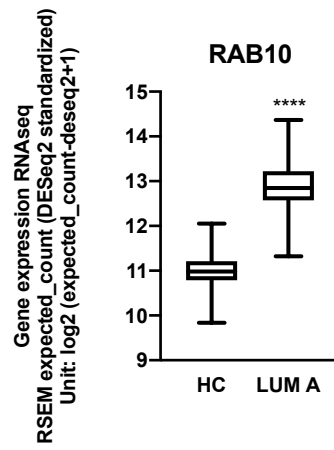

C'

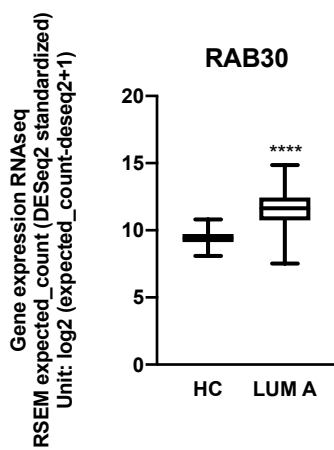

D'

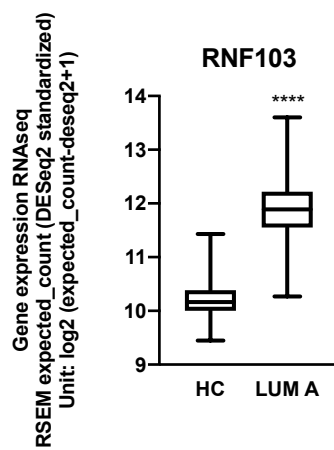

E'

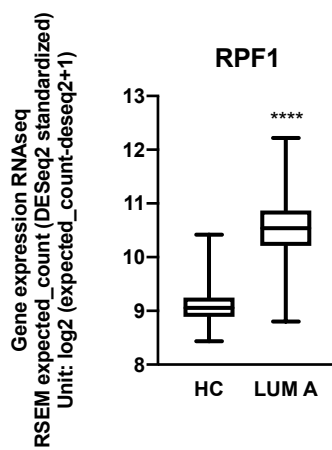

F'

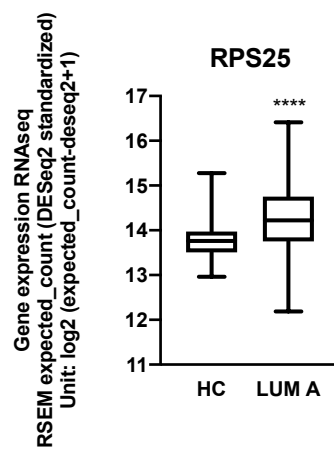

G'

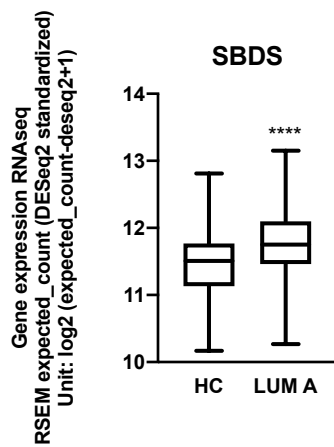

H'

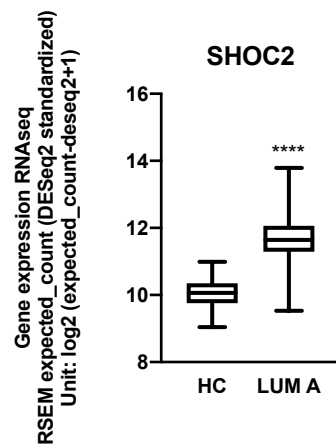

I'

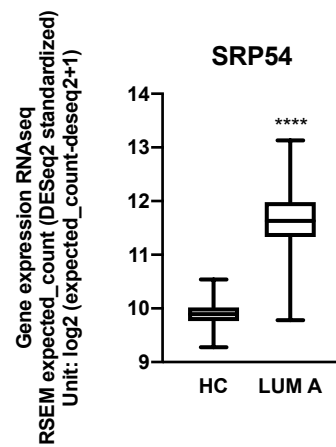

J'

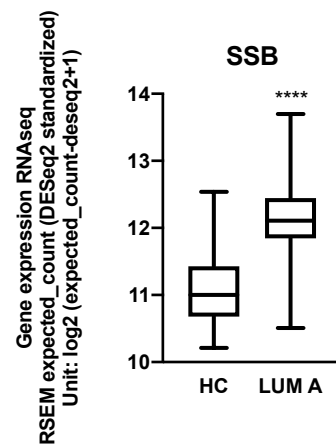

K'

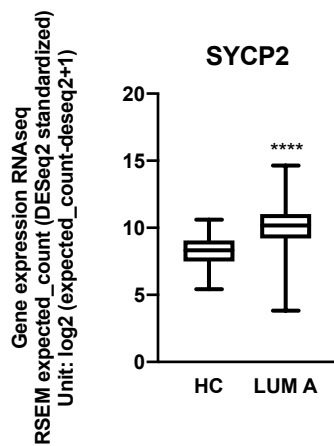

L'

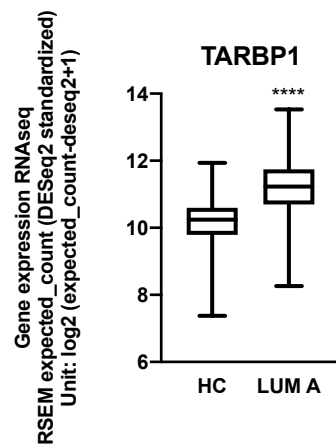

M'

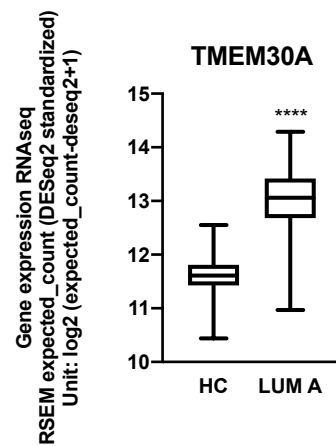

N'

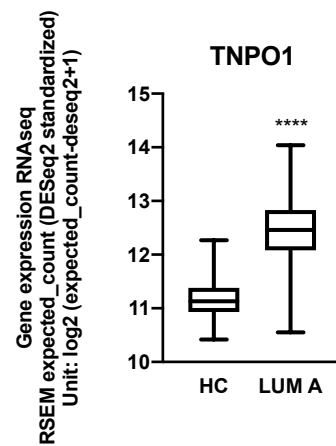

O'

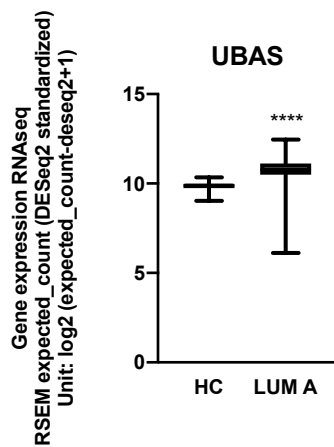

P'

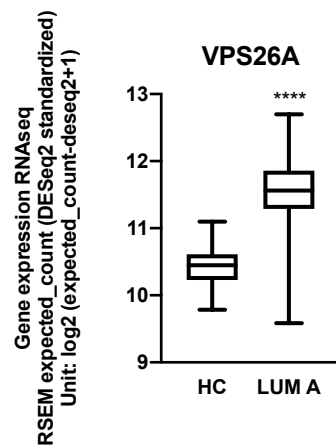

Q'

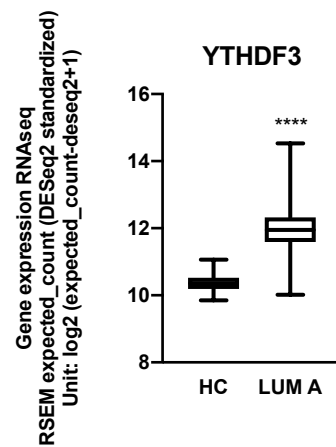

R'

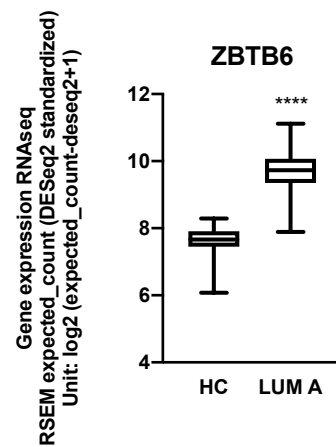

Supplement: Supplementary file 7 — Supplementary Figure S2. [file 41598_2021_291_MOESM7_ESM.pdf]

# si-NRIP1 T47D

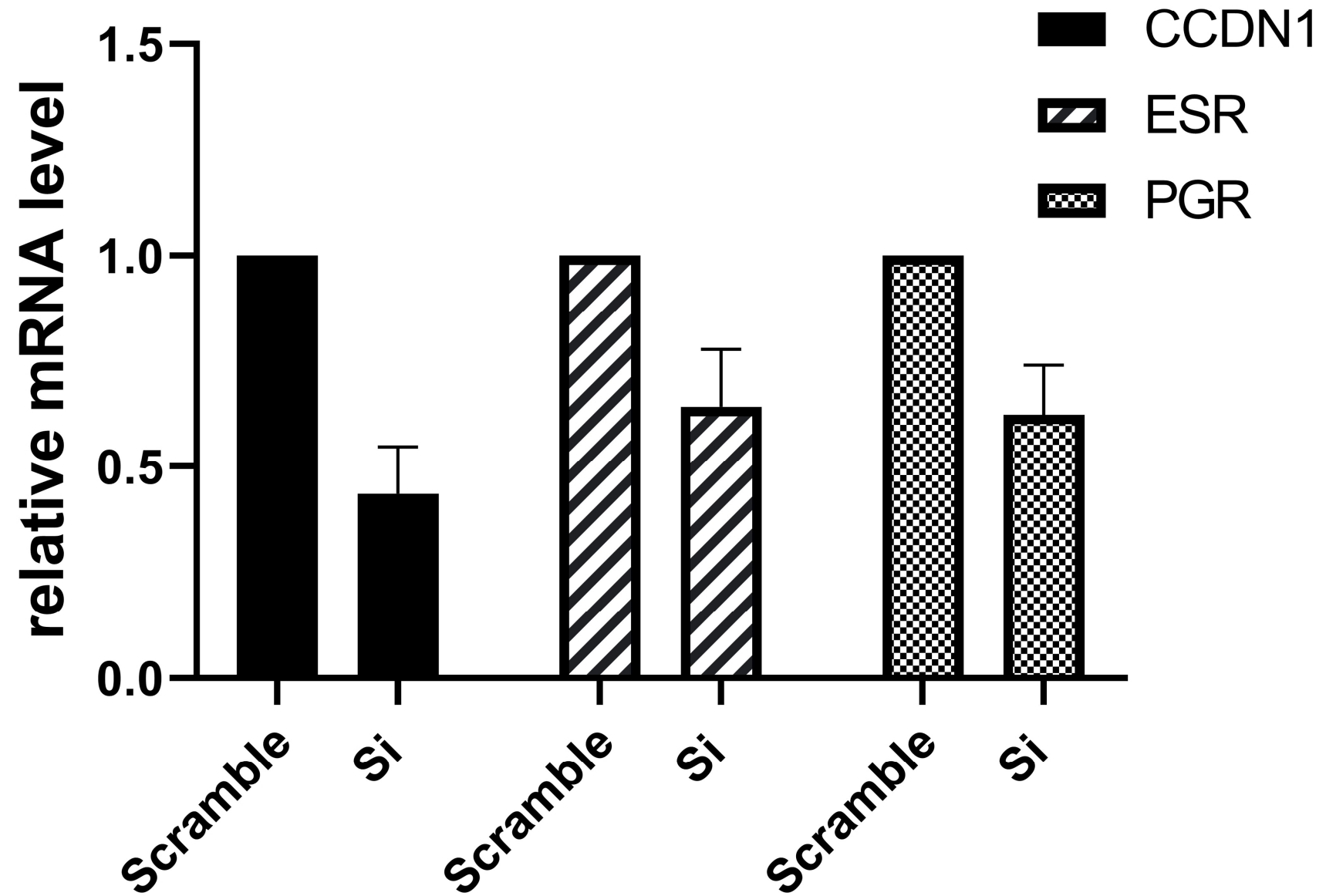

Supplement: Supplementary file 8 — Supplementary Figure S3. [file 41598_2021_291_MOESM8_ESM.pdf]
