# Supplementary material for: NRIP1 is activated by C-JUN/C-FOS and activates the expression of PGR, ESR1 and CCND1 in luminal A breast cancer
Source: Sci Rep. 2021 Oct 27;11:21159. doi: 10.1038/s41598-021-00291-w (PMC8551324; doi:10.1038/s41598-021-00291-w)
Supplement: Supplementary file 5 — Supplementary Legends. [file 41598_2021_291_MOESM5_ESM.docx]

## SUPPLEMENTARY FILES

**Supplementary Table 1**: List of primers used in RT-qPCR analysis.

**Supplementary Table 2**: List of the 2384 differentially expressed genes identified by the chip array assay using a ≥2-fold change as a cutoff to define overexpression or downregulation.

**Supplementary Table 3:** List of the differentially expressed genes that were identified by the chip array assay and potentially regulated by C-JUN and C-FOS.

**Supplementary Table 4:** List of 762 differentially expressed genes identified by the chip array assay related to NRIP1 silencing.

**Supplementary figure 1: Original images from Western blot assays.** Western blot analysis of C-JUN, C-FOS, C-JUN phosphorylated (c-JUNp) and C-FOS phosphorylated (c-FOSp) antibodies. Thirty micrograms of protein extracts from Healthy Donor and LUM A breast tissues were separated by SDS-PAGE and probed with the previously mentioned specific antibodies.

**Supplementary figure 2**: **Analysis of 45 genes regulated by C-JUN and/or C-FOS.** Box plot analysis of the 45 differentially expressed genes in luminal A that could be regulated by C-JUN and/or C-FOS transcription factors using the GTCGA-BC/GTEX dataset. The bars indicate the mean mRNA levels (± standard deviation). **p<0.01, ***p<0.001, ****p<0.0001.

**Supplementary figure 3: RT-qPCR of *NRIP1* target genes after *NRIP1* silencing using T47D cell line.** To determine the changes in the mRNA levels of *CCDN1*, *ESR1* and *PGR* genes after NRIP1 silencing, RT-qPCR assays were performed. The endogenous gene *GAPDH* was used for data normalization. The mRNA levels of *CCND1, ESR1* and *PGR* are decreased in T47D *NRIP1* silencing cell line. The bars indicate the mean mRNA levels (± standard deviation).
