# Supplementary material for: NRIP1 is activated by C-JUN/C-FOS and activates the expression of PGR, ESR1 and CCND1 in luminal A breast cancer
Source: Sci Rep. 2021 Oct 27;11:21159. doi: 10.1038/s41598-021-00291-w (PMC8551324; doi:10.1038/s41598-021-00291-w)
Supplement: Supplementary file 9 — Supplementary Table S1. [file 41598_2021_291_MOESM9_ESM.pdf]

Supplementary table 1 : List of primers used in RT-qPCR analysis.

	Fw	Rv
***NRIP1* gene**		
NRIP1 S1	5'AAGCGCATTAGCAACTTCATT TCC 3'	5'AAAGTACAGCTTAGTCTCACCCG 3'
NRIP1 S2	5'GGGGAGCGTTGAGGA TACGATTTT	5'CACTGAACCCGCCTCTAAATTGG 3'
***ZBTB6* gene**		
ZBTB6 S1	5' AGGAATAGAATTGGTGCAGAGGC 3'	5' TTTTGGTAGAGACGGGGTTTCAC 3';
ZBTB6 S2	5'GGAGAATTGCTTGAACCTGCTAGG 3'	5'GGTAGGCATCAAGTATTTGTGCTGA 3'
ZBTB6 S3	5'GATGCCTACCAAGTGCTAGACTT 3'	5' CCCAGTTTGAAACCTGTAATCCC 3'
ZBTB6 S4	5'GTGCCGTAACCGAACCTCA 3';	5' TTACCGGGTTGAGCCACC 3'
ZBTB6 S5	5' TGGCTCAACCCGGTAATCC 3	5' CACGCCCGGCTCATTTTTG 3'.
***DNAJC10* gene**		
DNAJC10 S1	5' CCTATAACCTCCTAATTGCCAGACC 3'	5' GGCTCCAGCTGTTCTTACTATATGG 3'
DNAJC10 S2	5' GCTGTGGGACAAAATGCAGTTC 3'	5' CTAGCGGACTGAAGAGGCAG 3'
***INHBA* gene**		
INHBA S1	5'ACTTGACCCCCA CAGGTTCAT 3'	5' GGCACAAAACAAACCCAGATCAGA 3'
***YTHDF-3* gene**		
YTHDF-3 S1	5' CCTGGAGGCATTGGAGATGGT 3'	5'AGTGAGGTTAGGAAACACGCCAC 3'
GAPDH	5' ATTCCACCCATGGCAAATTC 3'	5’ GGCGTGGATG GGTCTTTCA 3’;
NRIP1	5' ATTCCAACTGTGTTCCCATAGA 3'	5' CCCAAGTGTTTAGCAAGGATTG 3'
C-FOS	5' GGGCAAGGTGGAACAGTTAT 3'	5' CGCTTGGAGTGTA TCAGTCAG 3'
C-MYC	5' TCGGATTCTCTGCTCTCCT 3'	5' CTTCTTGTTCCTCCTCAGAGTC 3'
CCND1	5' TGGCCTCTAAGATGAAGGAGACC 3'	5' ACTTGAGCTTGTTCACCAGGAG 3'
HES1	5' AGAAGGCGGACATTCTGGAA 3';	5' CGTTCATGCACTCGCTGAAG 3'
PGR	5' GCATCAGGCTGTCATTATGG 3'	5' AGTAGTTGTGCTGCCCTTCC 3'
