# Supplementary material for: NRIP1 is activated by C-JUN/C-FOS and activates the expression of PGR, ESR1 and CCND1 in luminal A breast cancer
Source: Sci Rep. 2021 Oct 27;11:21159. doi: 10.1038/s41598-021-00291-w (PMC8551324; doi:10.1038/s41598-021-00291-w)
Supplement: Supplementary file 10 — Supplementary Table S2. [file 41598_2021_291_MOESM10_ESM.pdf]

Supplementary table 2: List of the 2384 differentially expressed genes identified by the chip array assay using a ≥2-fold change as a cutoff to define overexpression or downregulation.

Gene Symbol	gene_assignment	Fold-Change	Fold-Change(LUMINAL A vs. CONTROL)
POSTN	NM_006475 // POSTN // periostin, osteoblast specific factor // 13q13.3 // 10631	21,10	LUMINAL A up vs CONTROL
AGR2	NM_006408 // AGR2 // anterior gradient homolog 2 (Xenopus laevis) // 7p21.3 // 1	19,10	LUMINAL A up vs CONTROL
FN1	NM_212482 // FN1 // fibronectin 1 // 2q34 // 2335 /// NM_212475 // FN1 // fibron	17,66	LUMINAL A up vs CONTROL
DYNLL1	NM_001037494 // DYNLL1 // dynein, light chain, LC8-type 1 // 12q24.23 // 8655 //	17,53	LUMINAL A up vs CONTROL
CXCL11	NM_005409 // CXCL11 // chemokine (C-X-C motif) ligand 11 // 4q21.2 // 6373 /// E	15,88	LUMINAL A up vs CONTROL
CXCL9	NM_002416 // CXCL9 // chemokine (C-X-C motif) ligand 9 // 4q21 // 4283 /// ENST0	14,68	LUMINAL A up vs CONTROL
ASPN	NM_017680 // ASPN // asporin // 9q22 // 54829 /// ENST00000375544 // ASPN // asp	13,97	LUMINAL A up vs CONTROL
HLA-DPA1	NM_033554 // HLA-DPA1 // major histocompatibility complex, class II, DP alpha 1	13,18	LUMINAL A up vs CONTROL
COX6C	NM_004374 // COX6C // cytochrome c oxidase subunit VIc // 8q22-q23 // 1345 /// E	13,14	LUMINAL A up vs CONTROL
HLA-DPB1	NM_002121 // HLA-DPB1 // major histocompatibility complex, class II, DP beta 1 /	12,13	LUMINAL A up vs CONTROL
CYP2B7P1	NR_001278 // CYP2B7P1 // cytochrome P450, family 2, subfamily B, polypeptide 7 p	12,11	LUMINAL A up vs CONTROL
CXCL10	NM_001565 // CXCL10 // chemokine (C-X-C motif) ligand 10 // 4q21 // 3627 /// ENS	11,85	LUMINAL A up vs CONTROL
RPS24	NM_033022 // RPS24 // ribosomal protein S24 // 10q22-q23 // 6229 /// NM_00114228	11,57	LUMINAL A up vs CONTROL
GPR174	NM_032553 // GPR174 // G protein-coupled receptor 174 // Xq21.1 // 84636 /// ENS	11,44	LUMINAL A up vs CONTROL
RGS1	NM_002922 // RGS1 // regulator of G-protein signaling 1 // 1q31 // 5996 /// ENST	10,24	LUMINAL A up vs CONTROL
AGR3	NM_176813 // AGR3 // anterior gradient homolog 3 (Xenopus laevis) // 7p21.1 // 1	9,64	LUMINAL A up vs CONTROL
TSPAN1	NM_005727 // TSPAN1 // tetraspanin 1 // 1p34.1 // 10103 /// ENST00000372003 // T	9,42	LUMINAL A up vs CONTROL
FAM91A1	NM_144963 // FAM91A1 // family with sequence similarity 91, member A1 // 8q24.13	9,33	LUMINAL A up vs CONTROL
IGJ	NM_144646 // IGJ // immunoglobulin J polypeptide, linker protein for immunoglobu	9,19	LUMINAL A up vs CONTROL
MAL2	NM_052886 // MAL2 // mal, T-cell differentiation protein 2 // 8q23 // 114569 ///	9,10	LUMINAL A up vs CONTROL
CNIH4	NM_014184 // CNIH4 // cornichon homolog 4 (Drosophila) // 1q42.11 // 29097 /// E	8,95	LUMINAL A up vs CONTROL
IFI44L	NM_006820 // IFI44L // interferon-induced protein 44-like // 1p31.1 // 10964 ///	8,88	LUMINAL A up vs CONTROL
PLEKHF2	NM_024613 // PLEKHF2 // pleckstrin homology domain containing, family F (with FY	8,82	LUMINAL A up vs CONTROL
SH3BGRL	NM_003022 // SH3BGRL // SH3 domain binding glutamic acid-rich protein like // Xq	8,77	LUMINAL A up vs CONTROL
BAMBI	NM_012342 // BAMBI // BMP and activin membrane-bound inhibitor homolog (Xenopus	8,50	LUMINAL A up vs CONTROL
VCAN	NM_004385 // VCAN // versican // 5q14.3 // 1462 /// NM_001164097 // VCAN // vers	8,49	LUMINAL A up vs CONTROL
LYZ	NM_000239 // LYZ // lysozyme // 12q15 // 4069 /// ENST00000261267 // LYZ // lyso	8,09	LUMINAL A up vs CONTROL
SPP1	NM_001040058 // SPP1 // secreted phosphoprotein 1 // 4q21-q25 // 6696 /// NM_000	8,00	LUMINAL A up vs CONTROL
HLA-DRA	NM_019111 // HLA-DRA // major histocompatibility complex, class II, DR alpha //	7,94	LUMINAL A up vs CONTROL

CCL19	NM_006274 // CCL19 // chemokine (C-C motif) ligand 19 // 9p13 // 6363 /// ENST00	7,74	LUMINAL A up vs CONTROL
HLA-DQA2	NM_020056 // HLA-DQA2 // major histocompatibility complex, class II, DQ alpha 2	7,57	LUMINAL A up vs CONTROL
GZMK	NM_002104 // GZMK // granzyme K (granzyme 3; tryptase II) // 5q11-q12 // 3003 //	7,43	LUMINAL A up vs CONTROL
TMTC3	NM_181783 // TMTC3 // transmembrane and tetratricopeptide repeat containing 3 //	7,37	LUMINAL A up vs CONTROL
ERH	NM_004450 // ERH // enhancer of rudimentary homolog (Drosophila) // 14q24.1|7q34	7,36	LUMINAL A up vs CONTROL
CPA3	NM_001870 // CPA3 // carboxypeptidase A3 (mast cell) // 3q21-q25 // 1359 /// ENS	7,22	LUMINAL A up vs CONTROL
SLC39A6	NM_012319 // SLC39A6 // solute carrier family 39 (zinc transporter), member 6 //	7,13	LUMINAL A up vs CONTROL
RPS29	NM_001030001 // RPS29 // ribosomal protein S29 // 14q // 6235 /// NM_001032 // R	7,13	LUMINAL A up vs CONTROL
UGDH	NM_003359 // UGDH // UDP-glucose 6-dehydrogenase // 4p15.1 // 7358 /// ENST00000	7,09	LUMINAL A up vs CONTROL
NEBL	NM_006393 // NEBL // nebulette // 10p12 // 10529 /// NM_213569 // NEBL // nebule	7,05	LUMINAL A up vs CONTROL
CD53	NM_000560 // CD53 // CD53 molecule // 1p13 // 963 /// NM_001040033 // CD53 // CD	6,96	LUMINAL A up vs CONTROL
CXCR4	NM_001008540 // CXCR4 // chemokine (C-X-C motif) receptor 4 // 2q21 // 7852 ///	6,89	LUMINAL A up vs CONTROL
CISD1	NM_018464 // CISD1 // CDGSH iron sulfur domain 1 // 10q21.1 // 55847 /// ENST000	6,78	LUMINAL A up vs CONTROL
GLIPR1	NM_006851 // GLIPR1 // GLI pathogenesis-related 1 // 12q21.2 // 11010 /// NM_007	6,76	LUMINAL A up vs CONTROL
FAP	NM_004460 // FAP // fibroblast activation protein, alpha // 2q23 // 2191 /// ENS	6,74	LUMINAL A up vs CONTROL
TOR1AIP2	NM_022347 // TOR1AIP2 // torsin A interacting protein 2 // 1q25.2 // 163590 ///	6,72	LUMINAL A up vs CONTROL
SLC38A1	NM_030674 // SLC38A1 // solute carrier family 38, member 1 // 12q13.11 // 81539	6,69	LUMINAL A up vs CONTROL
NAT1	NM_001160170 // NAT1 // N-acetyltransferase 1 (arylamine N-acetyltransferase) //	6,68	LUMINAL A up vs CONTROL
B2M	NM_004048 // B2M // beta-2-microglobulin // 15q21-q22.2 // 567 /// ENST000003492	6,64	LUMINAL A up vs CONTROL
MFSD1	NM_022736 // MFSD1 // major facilitator superfamily domain containing 1 // 3q25.	6,50	LUMINAL A up vs CONTROL
SLC16A6	NM_001174166 // SLC16A6 // solute carrier family 16, member 6 (monocarboxylic ac	6,47	LUMINAL A up vs CONTROL
CKS2	NM_001827 // CKS2 // CDC28 protein kinase regulatory subunit 2 // 9q22 // 1164 /	6,44	LUMINAL A up vs CONTROL
F2RL2	NM_004101 // F2RL2 // coagulation factor II (thrombin) receptor-like 2 // 5q13 /	6,40	LUMINAL A up vs CONTROL
SQLE	NM_003129 // SQLE // squalene epoxidase // 8q24.1 // 6713 /// ENST00000265896 //	6,34	LUMINAL A up vs CONTROL
CALM2	NM_001743 // CALM2 // calmodulin 2 (phosphorylase kinase, delta) // 2p21 // 805	6,26	LUMINAL A up vs CONTROL
C4orf34	BC008502 // C4orf34 // chromosome 4 open reading frame 34 // 4p14 // 201895 ///	6,21	LUMINAL A up vs CONTROL
HIST1H4H	NM_003543 // HIST1H4H // histone cluster 1, H4h // 6p21.3 // 8365 /// BC120939 /	6,16	LUMINAL A up vs CONTROL
HIST1H2BK	NM_080593 // HIST1H2BK // histone cluster 1, H2bk // 6p21.33 // 85236 /// ENST00	6,09	LUMINAL A up vs CONTROL
HIST1H2BD	NM_138720 // HIST1H2BD // histone cluster 1, H2bd // 6p21.3 // 3017 /// NM_02106	6,04	LUMINAL A up vs CONTROL
CFB	NM_001710 // CFB // complement factor B // 6p21.3 // 629 /// BC004143 // CFB //	6,02	LUMINAL A up vs CONTROL
PIGX	NM_001166304 // PIGX // phosphatidylinositol glycan anchor biosynthesis, class X	5,94	LUMINAL A up vs CONTROL
TSPAN13	NM_014399 // TSPAN13 // tetraspanin 13 // 7p21.1 // 27075 /// ENST00000262067 //	5,93	LUMINAL A up vs CONTROL
NPNT	NM_001033047 // NPNT // nephronectin // 4q24 // 255743 /// NM_001163435 // TBCK	5,88	LUMINAL A up vs CONTROL

CTSK	NM_000396 // CTSK // cathepsin K // 1q21 // 1513 /// ENST00000271651 // CTSK //	5,84	LUMINAL A up vs CONTROL
PTPRC	NM_002838 // PTPRC // protein tyrosine phosphatase, receptor type, C // 1q31-q32	5,79	LUMINAL A up vs CONTROL
ATP6V1C1	NM_001695 // ATP6V1C1 // ATPase, H+ transporting, lysosomal 42kDa, V1 subunit C1	5,77	LUMINAL A up vs CONTROL
HAS2	NM_005328 // HAS2 // hyaluronan synthase 2 // 8q24.12 // 3037 /// ENST0000030392	5,72	LUMINAL A up vs CONTROL
CYBB	NM_000397 // CYBB // cytochrome b-245, beta polypeptide // Xp21.1 // 1536 /// EN	5,70	LUMINAL A up vs CONTROL
LAPTM4A	NM_014713 // LAPTM4A // lysosomal protein transmembrane 4 alpha // 2p24.1 // 974	5,69	LUMINAL A up vs CONTROL
HIST1H3H	NM_003536 // HIST1H3H // histone cluster 1, H3h // 6p22-p21.3 // 8357 /// BC0961	5,67	LUMINAL A up vs CONTROL
TPD52	NM_001025252 // TPD52 // tumor protein D52 // 8q21 // 7163 /// NM_001025253 // T	5,66	LUMINAL A up vs CONTROL
C10orf57	NM_025125 // C10orf57 // chromosome 10 open reading frame 57 // 10q22.3 // 80195	5,63	LUMINAL A up vs CONTROL
CEACAM6	NM_002483 // CEACAM6 // carcinoembryonic antigen-related cell adhesion molecule	5,62	LUMINAL A up vs CONTROL
DERL1	NM_024295 // DERL1 // Der1-like domain family, member 1 // 8q24.13 // 79139 ///	5,55	LUMINAL A up vs CONTROL
EIF3E	NM_001568 // EIF3E // eukaryotic translation initiation factor 3, subunit E // 8	5,55	LUMINAL A up vs CONTROL
IL7R	NM_002185 // IL7R // interleukin 7 receptor // 5p13 // 3575 /// ENST00000303115	5,52	LUMINAL A up vs CONTROL
ANKRD22	NM_144590 // ANKRD22 // ankyrin repeat domain 22 // 10q23.31 // 118932 /// ENST0	5,50	LUMINAL A up vs CONTROL
SAMD9L	NM_152703 // SAMD9L // sterile alpha motif domain containing 9-like // 7q21.2 //	5,50	LUMINAL A up vs CONTROL
SYCP2	NM_014258 // SYCP2 // synaptonemal complex protein 2 // 20q13.33 // 10388 /// EN	5,49	LUMINAL A up vs CONTROL
RAG1AP1	NM_018845 // RAG1AP1 // recombination activating gene 1 activating protein 1 //	5,46	LUMINAL A up vs CONTROL
CTSS	NM_004079 // CTSS // cathepsin S // 1q21 // 1520 /// ENST00000368985 // CTSS //	5,44	LUMINAL A up vs CONTROL
ACTR3	NM_005721 // ACTR3 // ARP3 actin-related protein 3 homolog (yeast) // 2q14.1 //	5,44	LUMINAL A up vs CONTROL
RWDD4A	NM_152682 // RWDD4A // RWD domain containing 4A // 4q35.1 // 201965 /// ENST0000	5,44	LUMINAL A up vs CONTROL
SDHC	NM_003001 // SDHC // succinate dehydrogenase complex, subunit C, integral membra	5,39	LUMINAL A up vs CONTROL
TM9SF2	NM_004800 // TM9SF2 // transmembrane 9 superfamily member 2 // 13q32.3 // 9375 /	5,37	LUMINAL A up vs CONTROL
BIRC3	NM_001165 // BIRC3 // baculoviral IAP repeat-containing 3 // 11q22 // 330 /// NM	5,34	LUMINAL A up vs CONTROL
TRPS1	NM_014112 // TRPS1 // trichorhinophalangeal syndrome I // 8q24.12 // 7227 /// EN	5,31	LUMINAL A up vs CONTROL
LYPLA1	NM_006330 // LYPLA1 // lysophospholipase I // 8q11.23 // 10434 /// ENST000003169	5,30	LUMINAL A up vs CONTROL
ARL6IP1	NM_015161 // ARL6IP1 // ADP-ribosylation factor-like 6 interacting protein 1 //	5,29	LUMINAL A up vs CONTROL
OLR1	NM_002543 // OLR1 // oxidized low density lipoprotein (lectin-like) receptor 1 /	5,24	LUMINAL A up vs CONTROL
IGK@	BC032451 // IGK@ // immunoglobulin kappa locus // 2p12 // 50802 /// BC016380 //	5,22	LUMINAL A up vs CONTROL
LUM	NM_002345 // LUM // lumican // 12q21.3-q22 // 4060 /// ENST00000266718 // LUM //	5,22	LUMINAL A up vs CONTROL
KITLG	NM_000899 // KITLG // KIT ligand // 12q22 // 4254 /// NM_003994 // KITLG // KIT	5,22	LUMINAL A up vs CONTROL
SFRP2	NM_003013 // SFRP2 // secreted frizzled-related protein 2 // 4q31.3 // 6423 ///	5,20	LUMINAL A up vs CONTROL
DYNC1I2	NM_001378 // DYNC1I2 // dynein, cytoplasmic 1, intermediate chain 2 // 2q31.1 //	5,16	LUMINAL A up vs CONTROL
C4orf3	NM_001001701 // C4orf3 // chromosome 4 open reading frame 3 // 4q26 // 401152 //	5,16	LUMINAL A up vs CONTROL

DAD1	NM_001344 // DAD1 // defender against cell death 1 // 14q11-q12 // 1603 /// ENST	5,16	LUMINAL A up vs CONTROL
PPIP5K2	NM_015216 // PPIP5K2 // diphosphoinositol pentakisphosphate kinase 2 // 5q21.1 /	5,15	LUMINAL A up vs CONTROL
TC2N	NM_152332 // TC2N // tandem C2 domains, nuclear // 14q32.12 // 123036 /// NM_001	5,15	LUMINAL A up vs CONTROL
CD24	NM_013230 // CD24 // CD24 molecule // 6q21 // 100133941 /// ENST00000382840 // C	5,09	LUMINAL A up vs CONTROL
CPNE3	NM_003909 // CPNE3 // copine III // 8q21.3 // 8895 /// ENST00000198765 // CPNE3	5,05	LUMINAL A up vs CONTROL
CX3CR1	NM_001171171 // CX3CR1 // chemokine (C-X3-C motif) receptor 1 // 3p21|3p21.3 //	5,03	LUMINAL A up vs CONTROL
ATP1B1	NM_001677 // ATP1B1 // ATPase, Na+/K+ transporting, beta 1 polypeptide // 1q24 /	5,03	LUMINAL A up vs CONTROL
LCP1	NM_002298 // LCP1 // lymphocyte cytosolic protein 1 (L-plastin) // 13q14.3 // 39	5,00	LUMINAL A up vs CONTROL
ZBTB41	NM_194314 // ZBTB41 // zinc finger and BTB domain containing 41 // 1q31.3 // 360	5,00	LUMINAL A up vs CONTROL
CA12	NM_001218 // CA12 // carbonic anhydrase XII // 15q22 // 771 /// NM_206925 // CA1	4,99	LUMINAL A up vs CONTROL
NIPAL2	NM_024759 // NIPAL2 // NIPA-like domain containing 2 // 8q22.2 // 79815 /// ENST	4,97	LUMINAL A up vs CONTROL
BZW1	NM_014670 // BZW1 // basic leucine zipper and W2 domains 1 // 2q33 // 9689 /// N	4,97	LUMINAL A up vs CONTROL
GRIA2	NM_001083619 // GRIA2 // glutamate receptor, ionotropic, AMPA 2 // 4q32-q33 // 2	4,97	LUMINAL A up vs CONTROL
SLC12A2	NM_001046 // SLC12A2 // solute carrier family 12 (sodium/potassium/chloride tran	4,96	LUMINAL A up vs CONTROL
PPP1R3C	NM_005398 // PPP1R3C // protein phosphatase 1, regulatory (inhibitor) subunit 3C	4,93	LUMINAL A up vs CONTROL
VAMP8	NM_003761 // VAMP8 // vesicle-associated membrane protein 8 (endobrevin) // 2p12	4,91	LUMINAL A up vs CONTROL
TMEM49	NM_030938 // TMEM49 // transmembrane protein 49 // 17q23.1 // 81671 /// NR_02949	4,89	LUMINAL A up vs CONTROL
GPR160	NM_014373 // GPR160 // G protein-coupled receptor 160 // 3q26.2-q27 // 26996 ///	4,88	LUMINAL A up vs CONTROL
LIPA	NM_001127605 // LIPA // lipase A, lysosomal acid, cholesterol esterase // 10q23.	4,87	LUMINAL A up vs CONTROL
TMEM144	NM_018342 // TMEM144 // transmembrane protein 144 // 4q32.1 // 55314 /// ENST000	4,87	LUMINAL A up vs CONTROL
TM9SF3	NM_020123 // TM9SF3 // transmembrane 9 superfamily member 3 // 10q24.1 // 56889	4,83	LUMINAL A up vs CONTROL
COL1A2	NM_000089 // COL1A2 // collagen, type I, alpha 2 // 7q22.1 // 1278 /// ENST00000	4,83	LUMINAL A up vs CONTROL
IGSF6	NM_005849 // IGSF6 // immunoglobulin superfamily, member 6 // 16p12-p13 // 10261	4,82	LUMINAL A up vs CONTROL
MMP11	NM_005940 // MMP11 // matrix metallopeptidase 11 (stromelysin 3) // 22q11.2|22q1	4,82	LUMINAL A up vs CONTROL
PCMTD1	NM_052937 // PCMTD1 // protein-L-isoaspartate (D-aspartate) O-methyltransferase	4,80	LUMINAL A up vs CONTROL
CASD1	NM_022900 // CASD1 // CAS1 domain containing 1 // 7q21.3 // 64921 /// ENST000002	4,79	LUMINAL A up vs CONTROL
DNAJC10	NM_018981 // DNAJC10 // DnaJ (Hsp40) homolog, subfamily C, member 10 // 2q32.1 /	4,79	LUMINAL A up vs CONTROL
FXYD3	NM_021910 // FXYD3 // FXYD domain containing ion transport regulator 3 // 19q13.	4,78	LUMINAL A up vs CONTROL
VPS13B	NM_017890 // VPS13B // vacuolar protein sorting 13 homolog B (yeast) // 8q22.2 /	4,77	LUMINAL A up vs CONTROL
SLC7A2	NM_003046 // SLC7A2 // solute carrier family 7 (cationic amino acid transporter,	4,76	LUMINAL A up vs CONTROL
HIST2H2AB	NM_175065 // HIST2H2AB // histone cluster 2, H2ab // 1q21 // 317772 /// ENST0000	4,74	LUMINAL A up vs CONTROL
CD69	NM_001781 // CD69 // CD69 molecule // 12p13-p12 // 969 /// NR_026672 // CD69 //	4,72	LUMINAL A up vs CONTROL
GPRC5A	NM_003979 // GPRC5A // G protein-coupled receptor, family C, group 5, member A /	4,71	LUMINAL A up vs CONTROL

VAV3	NM_006113 // VAV3 // vav 3 guanine nucleotide exchange factor // 1p13.3 // 10451	4,71	LUMINAL A up vs CONTROL
SLC40A1	NM_014585 // SLC40A1 // solute carrier family 40 (iron-regulated transporter), m	4,70	LUMINAL A up vs CONTROL
LRRC15	NM_001135057 // LRRC15 // leucine rich repeat containing 15 // 3q29 // 131578 //	4,68	LUMINAL A up vs CONTROL
TMCO1	NM_019026 // TMCO1 // transmembrane and coiled-coil domains 1 // 1q22-q25 // 544	4,68	LUMINAL A up vs CONTROL
TOP2A	NM_001067 // TOP2A // topoisomerase (DNA) II alpha 170kDa // 17q21-q22 // 7153 /	4,65	LUMINAL A up vs CONTROL
TFF1	NM_003225 // TFF1 // trefoil factor 1 // 21q22.3 // 7031 /// ENST00000291527 //	4,61	LUMINAL A up vs CONTROL
ESRP1	NM_017697 // ESRP1 // epithelial splicing regulatory protein 1 // 8q22.1 // 5484	4,58	LUMINAL A up vs CONTROL
TAS2R19	NM_176888 // TAS2R19 // taste receptor, type 2, member 19 // 12p13.2 // 259294 /	4,58	LUMINAL A up vs CONTROL
LOC286161	AK091672 // LOC286161 // hypothetical protein LOC286161 // 8p23.3 // 286161	4,55	LUMINAL A up vs CONTROL
HIST1H4E	NM_003545 // HIST1H4E // histone cluster 1, H4e // 6p21.3 // 8367 /// CR542180 /	4,53	LUMINAL A up vs CONTROL
CD9	NM_001769 // CD9 // CD9 molecule // 12p13.3 // 928 /// ENST00000382518 // CD9 //	4,53	LUMINAL A up vs CONTROL
DOCK10	NM_014689 // DOCK10 // dedicator of cytokinesis 10 // 2q36.2 // 55619 /// ENST00	4,53	LUMINAL A up vs CONTROL
SLC35F5	NM_025181 // SLC35F5 // solute carrier family 35, member F5 // 2q14.1 // 80255 /	4,52	LUMINAL A up vs CONTROL
UBA3	NM_003968 // UBA3 // ubiquitin-like modifier activating enzyme 3 // 3p24.3-p13 /	4,52	LUMINAL A up vs CONTROL
SFRP4	NM_003014 // SFRP4 // secreted frizzled-related protein 4 // 7p14.1 // 6424 ///	4,51	LUMINAL A up vs CONTROL
ZMPSTE24	NM_005857 // ZMPSTE24 // zinc metallopeptidase (STE24 homolog, S. cerevisiae) //	4,51	LUMINAL A up vs CONTROL
TSNAX	NM_005999 // TSNAX // translin-associated factor X // 1q42.1 // 7257 /// NR_0283	4,50	LUMINAL A up vs CONTROL
LAPTM5	NM_006762 // LAPTM5 // lysosomal protein transmembrane 5 // 1p34 // 7805 /// ENS	4,50	LUMINAL A up vs CONTROL
TMEM14B	NM_030969 // TMEM14B // transmembrane protein 14B // 6p25.1-p23 // 81853 /// NM_	4,49	LUMINAL A up vs CONTROL
HIST1H3I	NM_003533 // HIST1H3I // histone cluster 1, H3i // 6p22-p21.3 // 8354 /// BC0668	4,49	LUMINAL A up vs CONTROL
ZNF552	NM_024762 // ZNF552 // zinc finger protein 552 // 19q13.43 // 79818 /// ENST0000	4,48	LUMINAL A up vs CONTROL
PRLR	NM_000949 // PRLR // prolactin receptor // 5p13.2 // 5618 /// ENST00000382002 //	4,47	LUMINAL A up vs CONTROL
COPB2	NR_023350 // COPB2 // coatomer protein complex, subunit beta 2 (beta prime) // 3	4,47	LUMINAL A up vs CONTROL
RAD21	NM_006265 // RAD21 // RAD21 homolog (S. pombe) // 8q24 // 5885 /// ENST000002973	4,44	LUMINAL A up vs CONTROL
MORF4L2	NM_001142418 // MORF4L2 // mortality factor 4 like 2 // Xq22 // 9643 /// NM_0011	4,43	LUMINAL A up vs CONTROL
CD47	NM_001777 // CD47 // CD47 molecule // 3q13.1-q13.2 // 961 /// NM_001025079 // CD	4,42	LUMINAL A up vs CONTROL
STAT1	NM_007315 // STAT1 // signal transducer and activator of transcription 1, 91kDa	4,42	LUMINAL A up vs CONTROL
SLC39A8	NM_022154 // SLC39A8 // solute carrier family 39 (zinc transporter), member 8 //	4,41	LUMINAL A up vs CONTROL
COL3A1	NM_000090 // COL3A1 // collagen, type III, alpha 1 // 2q31 // 1281 /// ENST00000	4,40	LUMINAL A up vs CONTROL
HSN2	NM_213655 // HSN2 // hereditary sensory neuropathy, type II // 12p13.3 // 378465	4,39	LUMINAL A up vs CONTROL
LRBA	NM_006726 // LRBA // LPS-responsive vesicle trafficking, beach and anchor contai	4,39	LUMINAL A up vs CONTROL
ERGIC2	NM_016570 // ERGIC2 // ERGIC and golgi 2 // 12p11.22 // 51290 /// ENST0000036015	4,38	LUMINAL A up vs CONTROL
SC4MOL	NM_006745 // SC4MOL // sterol-C4-methyl oxidase-like // 4q32-q34 // 6307 /// NM_	4,38	LUMINAL A up vs CONTROL

HIST2H2BE	NM_003528 // HIST2H2BE // histone cluster 2, H2be // 1q21-q23 // 8349 /// ENST00	4,37	LUMINAL A up vs CONTROL
CSE1L	NM_001316 // CSE1L // CSE1 chromosome segregation 1-like (yeast) // 20q13 // 143	4,36	LUMINAL A up vs CONTROL
PTP4A1	NM_003463 // PTP4A1 // protein tyrosine phosphatase type IVA, member 1 // 6q12 /	4,34	LUMINAL A up vs CONTROL
MBOAT2	NM_138799 // MBOAT2 // membrane bound O-acyltransferase domain containing 2 // 2	4,33	LUMINAL A up vs CONTROL
ANKRD50	NM_020337 // ANKRD50 // ankyrin repeat domain 50 // 4q28.1 // 57182 /// NM_00116	4,32	LUMINAL A up vs CONTROL
ALCAM	NM_001627 // ALCAM // activated leukocyte cell adhesion molecule // 3q13.1 // 21	4,31	LUMINAL A up vs CONTROL
AZIN1	NM_015878 // AZIN1 // antizyme inhibitor 1 // 8q22.3 // 51582 /// NM_148174 // A	4,31	LUMINAL A up vs CONTROL
ZDHHC13	NM_019028 // ZDHHC13 // zinc finger, DHHC-type containing 13 // 11p15.1 // 54503	4,30	LUMINAL A up vs CONTROL
TRAM1	NM_014294 // TRAM1 // translocation associated membrane protein 1 // 8q13.3 // 2	4,30	LUMINAL A up vs CONTROL
JKAMP	NM_016475 // JKAMP // JNK1/MAPK8-associated membrane protein // 14q23.1 // 51528	4,29	LUMINAL A up vs CONTROL
KIAA0196	NM_014846 // KIAA0196 // KIAA0196 // 8q24.13 // 9897 /// ENST00000318410 // KIAA	4,29	LUMINAL A up vs CONTROL
ARRDC3	NM_020801 // ARRDC3 // arrestin domain containing 3 // 5q14.3 // 57561 /// ENST0	4,29	LUMINAL A up vs CONTROL
BTF3L4	NM_152265 // BTF3L4 // basic transcription factor 3-like 4 // 1p32.3 // 91408 //	4,28	LUMINAL A up vs CONTROL
TMEM161B	NM_153354 // TMEM161B // transmembrane protein 161B // 5q14.3 // 153396 /// ENST	4,27	LUMINAL A up vs CONTROL
EVI2B	NM_006495 // EVI2B // ecotropic viral integration site 2B // 17q11.2 // 2124 ///	4,26	LUMINAL A up vs CONTROL
SULF1	NM_001128205 // SULF1 // sulfatase 1 // 8q13.2-q13.3 // 23213 /// NM_015170 // S	4,26	LUMINAL A up vs CONTROL
C14orf129	NM_016472 // C14orf129 // chromosome 14 open reading frame 129 // 14q32.2 // 515	4,25	LUMINAL A up vs CONTROL
CD48	NM_001778 // CD48 // CD48 molecule // 1q21.3-q22 // 962 /// ENST00000368046 // C	4,25	LUMINAL A up vs CONTROL
KDELR2	NM_006854 // KDELR2 // KDEL (Lys-Asp-Glu-Leu) endoplasmic reticulum protein rete	4,23	LUMINAL A up vs CONTROL
SCGB2A2	NM_002411 // SCGB2A2 // secretoglobin, family 2A, member 2 // 11q13 // 4250 ///	4,20	LUMINAL A up vs CONTROL
TBL1XR1	NM_024665 // TBL1XR1 // transducin (beta)-like 1 X-linked receptor 1 // 3q26.32	4,19	LUMINAL A up vs CONTROL
SLC44A4	NM_025257 // SLC44A4 // solute carrier family 44, member 4 // 6p21.3 // 80736 //	4,18	LUMINAL A up vs CONTROL
P4HA1	NM_000917 // P4HA1 // prolyl 4-hydroxylase, alpha polypeptide I // 10q21.3-q23.1	4,18	LUMINAL A up vs CONTROL
MRPS14	NM_022100 // MRPS14 // mitochondrial ribosomal protein S14 // 1q23-q25 // 63931	4,17	LUMINAL A up vs CONTROL
SSR1	NM_003144 // SSR1 // signal sequence receptor, alpha // 6p24.3 // 6745 /// ENST0	4,17	LUMINAL A up vs CONTROL
POTEE	NM_001083538 // POTEE // POTE ankyrin domain family, member E // 2q21.1 // 44558	4,17	LUMINAL A up vs CONTROL
LASS6	NM_203463 // LASS6 // LAG1 homolog, ceramide synthase 6 // 2q24.3 // 253782 ///	4,16	LUMINAL A up vs CONTROL
PPT1	NM_000310 // PPT1 // palmitoyl-protein thioesterase 1 // 1p32 // 5538 /// NM_001	4,16	LUMINAL A up vs CONTROL
ATP6AP2	NM_005765 // ATP6AP2 // ATPase, H+ transporting, lysosomal accessory protein 2 /	4,16	LUMINAL A up vs CONTROL
C3orf63	NM_001112736 // C3orf63 // chromosome 3 open reading frame 63 // 3p14.3 // 23272	4,15	LUMINAL A up vs CONTROL
ZNF587	NM_032828 // ZNF587 // zinc finger protein 587 // 19q13.43 // 84914 /// ENST0000	4,15	LUMINAL A up vs CONTROL
PPP1CC	NM_002710 // PPP1CC // protein phosphatase 1, catalytic subunit, gamma isozyme /	4,13	LUMINAL A up vs CONTROL
WWP1	NM_007013 // WWP1 // WW domain containing E3 ubiquitin protein ligase 1 // 8q21	4,13	LUMINAL A up vs CONTROL

ARF4	NM_001660 // ARF4 // ADP-ribosylation factor 4 // 3p21.2-p21.1 // 378 /// ENST00	4,12	LUMINAL A up vs CONTROL
LMBR1	NM_022458 // LMBR1 // limb region 1 homolog (mouse) // 7q36 // 64327 /// ENST000	4,12	LUMINAL A up vs CONTROL
POLR2B	NM_000938 // POLR2B // polymerase (RNA) II (DNA directed) polypeptide B, 140kDa	4,11	LUMINAL A up vs CONTROL
C1orf162	NM_174896 // C1orf162 // chromosome 1 open reading frame 162 // 1p13.2 // 128346	4,09	LUMINAL A up vs CONTROL
TAS2R4	NM_016944 // TAS2R4 // taste receptor, type 2, member 4 // 7q31.3-q32 // 50832 /	4,08	LUMINAL A up vs CONTROL
SMAD2	NM_005901 // SMAD2 // SMAD family member 2 // 18q21.1 // 4087 /// NM_001003652 /	4,08	LUMINAL A up vs CONTROL
PDP1	NM_001161778 // PDP1 // pyruvate dehyrogenase phosphatase catalytic subunit 1 //	4,07	LUMINAL A up vs CONTROL
FAM198B	NM_001128424 // FAM198B // family with sequence similarity 198, member B // 4q32	4,06	LUMINAL A up vs CONTROL
NELL2	NM_001145108 // NELL2 // NEL-like 2 (chicken) // 12q13.11-q13.12 // 4753 /// NM_	4,06	LUMINAL A up vs CONTROL
ARL1	NM_001177 // ARL1 // ADP-ribosylation factor-like 1 // 12q23.2 // 400 /// ENST00	4,06	LUMINAL A up vs CONTROL
ZNF706	NM_001042510 // ZNF706 // zinc finger protein 706 // 8q22.3 // 51123 /// NM_0160	4,04	LUMINAL A up vs CONTROL
TNFAIP6	NM_007115 // TNFAIP6 // tumor necrosis factor, alpha-induced protein 6 // 2q23.3	4,04	LUMINAL A up vs CONTROL
H2AFZ	NM_002106 // H2AFZ // H2A histone family, member Z // 4q24 // 3015 /// ENST00000	4,01	LUMINAL A up vs CONTROL
PLAC8	NM_016619 // PLAC8 // placenta-specific 8 // 4q21.22 // 51316 /// NM_001130715 /	4,01	LUMINAL A up vs CONTROL
EDEM3	NM_025191 // EDEM3 // ER degradation enhancer, mannosidase alpha-like 3 // 1q24-	4,00	LUMINAL A up vs CONTROL
COMMD3	NM_012071 // COMMD3 // COMM domain containing 3 // 10pter-q22.1 // 23412 /// ENS	4,00	LUMINAL A up vs CONTROL
CD52	NM_001803 // CD52 // CD52 molecule // 1p36 // 1043 /// ENST00000374213 // CD52 /	3,99	LUMINAL A up vs CONTROL
TAF2	NM_003184 // TAF2 // TAF2 RNA polymerase II, TATA box binding protein (TBP)-asso	3,98	LUMINAL A up vs CONTROL
C3AR1	NM_004054 // C3AR1 // complement component 3a receptor 1 // 12p13.31 // 719 ///	3,98	LUMINAL A up vs CONTROL
MXRA5	NM_015419 // MXRA5 // matrix-remodelling associated 5 // Xp22.33 // 25878 /// EN	3,98	LUMINAL A up vs CONTROL
ZNF146	NM_007145 // ZNF146 // zinc finger protein 146 // 19q13.1 // 7705 /// NM_0010996	3,98	LUMINAL A up vs CONTROL
C7	NM_000587 // C7 // complement component 7 // 5p13 // 730 /// ENST00000313164 //	3,97	LUMINAL A up vs CONTROL
SCCPDH	NM_016002 // SCCPDH // saccharopine dehydrogenase (putative) // 1q44 // 51097 //	3,96	LUMINAL A up vs CONTROL
CLDN12	NM_012129 // CLDN12 // claudin 12 // 7q21 // 9069 /// ENST00000287916 // CLDN12	3,96	LUMINAL A up vs CONTROL
DCUN1D1	NM_020640 // DCUN1D1 // DCN1, defective in cullin neddylation 1, domain containi	3,96	LUMINAL A up vs CONTROL
TROVE2	NM_004600 // TROVE2 // TROVE domain family, member 2 // 1q31 // 6738 /// NM_0011	3,95	LUMINAL A up vs CONTROL
ZBED5	NM_021211 // ZBED5 // zinc finger, BED-type containing 5 // 11p15.3 // 58486 ///	3,94	LUMINAL A up vs CONTROL
HIATL1	NM_032558 // HIATL1 // hippocampus abundant transcript-like 1 // 9q22.32 // 8464	3,94	LUMINAL A up vs CONTROL
S100P	NM_005980 // S100P // S100 calcium binding protein P // 4p16 // 6286 /// ENST000	3,94	LUMINAL A up vs CONTROL
ARFGEF1	NM_006421 // ARFGEF1 // ADP-ribosylation factor guanine nucleotide-exchange fact	3,94	LUMINAL A up vs CONTROL
LSM14B	NM_144703 // LSM14B // LSM14B, SCD6 homolog B (S. cerevisiae) // 20q13.33 // 149	3,94	LUMINAL A up vs CONTROL
CXADR	NM_001338 // CXADR // coxsackie virus and adenovirus receptor // 21q21.1 // 1525	3,93	LUMINAL A up vs CONTROL
PSMA4	NM_002789 // PSMA4 // proteasome (prosome, macropain) subunit, alpha type, 4 //	3,93	LUMINAL A up vs CONTROL

SLC39A9	NM_018375 // SLC39A9 // solute carrier family 39 (zinc transporter), member 9 //	3,93	LUMINAL A up vs CONTROL
FCGR3A	NM_000569 // FCGR3A // Fc fragment of IgG, low affinity IIIa, receptor (CD16a) /	3,93	LUMINAL A up vs CONTROL
NTN4	NM_021229 // NTN4 // netrin 4 // 12q22-q23 // 59277 /// ENST00000343702 // NTN4	3,92	LUMINAL A up vs CONTROL
FPR3	NM_002030 // FPR3 // formyl peptide receptor 3 // 19q13.3-q13.4 // 2359 /// ENST	3,92	LUMINAL A up vs CONTROL
S100A14	NM_020672 // S100A14 // S100 calcium binding protein A14 // 1q21.3 // 57402 ///	3,92	LUMINAL A up vs CONTROL
POLR2K	NM_005034 // POLR2K // polymerase (RNA) II (DNA directed) polypeptide K, 7.0kDa	3,90	LUMINAL A up vs CONTROL
FYB	NM_001465 // FYB // FYN binding protein // 5p13.1 // 2533 /// NM_199335 // FYB /	3,89	LUMINAL A up vs CONTROL
CD3D	NM_000732 // CD3D // CD3d molecule, delta (CD3-TCR complex) // 11q23 // 915 ///	3,89	LUMINAL A up vs CONTROL
ERBB2IP	NM_018695 // ERBB2IP // erbb2 interacting protein // 5q12.3 // 55914 /// NM_0010	3,89	LUMINAL A up vs CONTROL
CEACAM5	NM_004363 // CEACAM5 // carcinoembryonic antigen-related cell adhesion molecule	3,88	LUMINAL A up vs CONTROL
RALGPS2	NM_152663 // RALGPS2 // Ral GEF with PH domain and SH3 binding motif 2 // 1q25.2	3,87	LUMINAL A up vs CONTROL
HIST1H4C	NM_003542 // HIST1H4C // histone cluster 1, H4c // 6p21.3 // 8364 /// BC130558 /	3,87	LUMINAL A up vs CONTROL
SLC38A2	NM_018976 // SLC38A2 // solute carrier family 38, member 2 // 12q // 54407 /// E	3,87	LUMINAL A up vs CONTROL
TAS2R31	NM_176885 // TAS2R31 // taste receptor, type 2, member 31 // 12p13.2 // 259290 /	3,86	LUMINAL A up vs CONTROL
COL10A1	NM_000493 // COL10A1 // collagen, type X, alpha 1 // 6q21-q22 // 1300 /// ENST00	3,86	LUMINAL A up vs CONTROL
DDX60	NM_017631 // DDX60 // DEAD (Asp-Glu-Ala-Asp) box polypeptide 60 // 4q32.3 // 556	3,85	LUMINAL A up vs CONTROL
ACTB	NM_001101 // ACTB // actin, beta // 7p15-p12 // 60 /// ENST00000331789 // ACTB /	3,84	LUMINAL A up vs CONTROL
C6orf211	AK298490 // C6orf211 // chromosome 6 open reading frame 211 // 6q25.1 // 79624 /	3,84	LUMINAL A up vs CONTROL
PARP9	NM_001146106 // PARP9 // poly (ADP-ribose) polymerase family, member 9 // 3q21 /	3,83	LUMINAL A up vs CONTROL
AOAH	NM_001177506 // AOAH // acyloxyacyl hydrolase (neutrophil) // 7p14-p12 // 313 //	3,83	LUMINAL A up vs CONTROL
HIST1H3C	NM_003531 // HIST1H3C // histone cluster 1, H3c // 6p21.3 // 8352 /// BC127610 /	3,83	LUMINAL A up vs CONTROL
HAT1	NM_003642 // HAT1 // histone acetyltransferase 1 // 2q31.2-q33.1 // 8520 /// NR_	3,83	LUMINAL A up vs CONTROL
C3orf1	NM_016589 // C3orf1 // chromosome 3 open reading frame 1 // 3q13.33 // 51300 ///	3,83	LUMINAL A up vs CONTROL
BMI1	NM_005180 // BMI1 // BMI1 polycomb ring finger oncogene // 10p11.23 // 648 /// E	3,83	LUMINAL A up vs CONTROL
CAPZA1	NM_006135 // CAPZA1 // capping protein (actin filament) muscle Z-line, alpha 1 /	3,82	LUMINAL A up vs CONTROL
NUDT12	NM_031438 // NUDT12 // nudix (nucleoside diphosphate linked moiety X)-type motif	3,82	LUMINAL A up vs CONTROL
DPY19L4	NM_181787 // DPY19L4 // dpy-19-like 4 (C. elegans) // 8q22.1 // 286148 /// ENST0	3,82	LUMINAL A up vs CONTROL
ROD1	NM_005156 // ROD1 // ROD1 regulator of differentiation 1 (S. pombe) // 9q32 // 9	3,81	LUMINAL A up vs CONTROL
STT3B	NM_178862 // STT3B // STT3, subunit of the oligosaccharyltransferase complex, ho	3,81	LUMINAL A up vs CONTROL
PTP4A2	NM_080391 // PTP4A2 // protein tyrosine phosphatase type IVA, member 2 // 1p35 /	3,80	LUMINAL A up vs CONTROL
EXOC6	NM_019053 // EXOC6 // exocyst complex component 6 // 10q23.33 // 54536 /// NM_00	3,79	LUMINAL A up vs CONTROL
GOLPH3L	NM_018178 // GOLPH3L // golgi phosphoprotein 3-like // 1q21.3 // 55204 /// ENST0	3,79	LUMINAL A up vs CONTROL
PPP2CA	NM_002715 // PPP2CA // protein phosphatase 2, catalytic subunit, alpha isozyme /	3,79	LUMINAL A up vs CONTROL

IFI44	NM_006417 // IFI44 // interferon-induced protein 44 // 1p31.1 // 10561 /// ENST0	3,78	LUMINAL A up vs CONTROL
CLEC3A	NM_005752 // CLEC3A // C-type lectin domain family 3, member A // 16q23 // 10143	3,78	LUMINAL A up vs CONTROL
PSMB5	NM_001144932 // PSMB5 // proteasome (prosome, macropain) subunit, beta type, 5 /	3,77	LUMINAL A up vs CONTROL
01/mar	NM_001166373 // MARCH1 // membrane-associated ring finger (C3HC4) 1 // 4q32.2-q3	3,77	LUMINAL A up vs CONTROL
OAT	NM_000274 // OAT // ornithine aminotransferase // 10q26 // 4942 /// NM_001171814	3,77	LUMINAL A up vs CONTROL
DPY30	NM_032574 // DPY30 // dpy-30 homolog (C. elegans) // 2p22.3 // 84661 /// ENST000	3,76	LUMINAL A up vs CONTROL
DNAJC13	NM_015268 // DNAJC13 // DnaJ (Hsp40) homolog, subfamily C, member 13 // 3q22.1 /	3,75	LUMINAL A up vs CONTROL
RAB18	NM_021252 // RAB18 // RAB18, member RAS oncogene family // 10p12.1 // 22931 ///	3,75	LUMINAL A up vs CONTROL
API5	NR_024625 // API5 // apoptosis inhibitor 5 // 11p11.2 // 8539 /// NM_001142930 /	3,74	LUMINAL A up vs CONTROL
ADIPOR1	NM_001127687 // ADIPOR1 // adiponectin receptor 1 // 1p36.13-q41 // 51094 /// NM	3,74	LUMINAL A up vs CONTROL
KIAA1191	NM_020444 // KIAA1191 // KIAA1191 // 5q35.2 // 57179 /// NM_001079685 // KIAA119	3,74	LUMINAL A up vs CONTROL
DNAJC1	NM_022365 // DNAJC1 // DnaJ (Hsp40) homolog, subfamily C, member 1 // 10p12.31 /	3,74	LUMINAL A up vs CONTROL
STT3A	NM_152713 // STT3A // STT3, subunit of the oligosaccharyltransferase complex, ho	3,73	LUMINAL A up vs CONTROL
SLC44A1	NM_080546 // SLC44A1 // solute carrier family 44, member 1 // 9q31.2 // 23446 //	3,72	LUMINAL A up vs CONTROL
SET	NM_001122821 // SET // SET nuclear oncogene // 9q34 // 6418 /// NM_003011 // SET	3,72	LUMINAL A up vs CONTROL
LRRCC1	NM_033402 // LRRCC1 // leucine rich repeat and coiled-coil domain containing 1 /	3,71	LUMINAL A up vs CONTROL
DENND1B	NM_144977 // DENND1B // DENN/MADD domain containing 1B // 1q31.3 // 163486 /// N	3,71	LUMINAL A up vs CONTROL
SAMD9	NM_017654 // SAMD9 // sterile alpha motif domain containing 9 // 7q21.2 // 54809	3,70	LUMINAL A up vs CONTROL
RARRES3	NM_004585 // RARRES3 // retinoic acid receptor responder (tazarotene induced) 3	3,70	LUMINAL A up vs CONTROL
DRAM2	NM_178454 // DRAM2 // DNA-damage regulated autophagy modulator 2 // 1p13.3 // 12	3,70	LUMINAL A up vs CONTROL
CRYZ	NM_001130042 // CRYZ // crystallin, zeta (quinone reductase) // 1p31-p22 // 1429	3,70	LUMINAL A up vs CONTROL
GPR137B	NM_003272 // GPR137B // G protein-coupled receptor 137B // 1q42-q43 // 7107 ///	3,69	LUMINAL A up vs CONTROL
HLA-DMB	NM_002118 // HLA-DMB // major histocompatibility complex, class II, DM beta // 6	3,69	LUMINAL A up vs CONTROL
CTNNB1	NM_001904 // CTNNB1 // catenin (cadherin-associated protein), beta 1, 88kDa // 3	3,69	LUMINAL A up vs CONTROL
ELOVL2	NM_017770 // ELOVL2 // elongation of very long chain fatty acids (FEN1/Elo2, SUR	3,69	LUMINAL A up vs CONTROL
PARP14	NM_017554 // PARP14 // poly (ADP-ribose) polymerase family, member 14 // 3q21.1	3,68	LUMINAL A up vs CONTROL
SCAMP1	NM_004866 // SCAMP1 // secretory carrier membrane protein 1 // 5q13.3-q14.1 // 9	3,68	LUMINAL A up vs CONTROL
MYSM1	NM_001085487 // MYSM1 // Myb-like, SWIRM and MPN domains 1 // 1p32.1 // 114803 /	3,68	LUMINAL A up vs CONTROL
RNF146	NM_030963 // RNF146 // ring finger protein 146 // 6q22.1-q22.33 // 81847 /// ENS	3,67	LUMINAL A up vs CONTROL
SCGB1D2	NM_006551 // SCGB1D2 // secretoglobin, family 1D, member 2 // 11q13 // 10647 ///	3,67	LUMINAL A up vs CONTROL
C15orf48	NM_032413 // C15orf48 // chromosome 15 open reading frame 48 // 15q21.1 // 84419	3,66	LUMINAL A up vs CONTROL
KIAA1429	NM_015496 // KIAA1429 // KIAA1429 // 8q22.1 // 25962 /// NM_183009 // KIAA1429 /	3,66	LUMINAL A up vs CONTROL
RAB31	NM_006868 // RAB31 // RAB31, member RAS oncogene family // 18p11.3 // 11031 ///	3,65	LUMINAL A up vs CONTROL

SOAT1	NM_003101 // SOAT1 // sterol O-acyltransferase 1 // 1q25 // 6646 /// ENST0000036	3,64	LUMINAL A up vs CONTROL
POT1	NM_015450 // POT1 // protection of telomeres 1 homolog (S. pombe) // 7q31.33 //	3,64	LUMINAL A up vs CONTROL
CYSLTR1	NM_006639 // CYSLTR1 // cysteinyl leukotriene receptor 1 // Xq13.2-q21.1 // 1080	3,64	LUMINAL A up vs CONTROL
COPB1	NM_016451 // COPB1 // coatomer protein complex, subunit beta 1 // 11p15.2 // 131	3,64	LUMINAL A up vs CONTROL
TIPRL	NM_152902 // TIPRL // TIP41, TOR signaling pathway regulator-like (S. cerevisiae	3,64	LUMINAL A up vs CONTROL
CLTC	NM_004859 // CLTC // clathrin, heavy chain (Hc) // 17q11-qter // 1213 /// ENST00	3,63	LUMINAL A up vs CONTROL
RAP1GDS1	NM_001100426 // RAP1GDS1 // RAP1, GTP-GDP dissociation stimulator 1 // 4q23-q25	3,63	LUMINAL A up vs CONTROL
CLGN	NM_001130675 // CLGN // calmegin // 4q28.3-q31.1 // 1047 /// NM_004362 // CLGN /	3,61	LUMINAL A up vs CONTROL
SRGN	NM_002727 // SRGN // serglycin // 10q22.1 // 5552 /// ENST00000242465 // SRGN //	3,61	LUMINAL A up vs CONTROL
SLC33A1	NM_004733 // SLC33A1 // solute carrier family 33 (acetyl-CoA transporter), membe	3,60	LUMINAL A up vs CONTROL
LUZP6	NM_001128619 // LUZP6 // leucine zipper protein 6 // 7q33 // 767558 /// NM_14580	3,60	LUMINAL A up vs CONTROL
HMGCS1	NM_001098272 // HMGCS1 // 3-hydroxy-3-methylglutaryl-CoA synthase 1 (soluble) //	3,60	LUMINAL A up vs CONTROL
LRRN1	NM_020873 // LRRN1 // leucine rich repeat neuronal 1 // 3p26.2 // 57633 /// ENST	3,60	LUMINAL A up vs CONTROL
MYO6	NM_004999 // MYO6 // myosin VI // 6q13 // 4646 /// ENST00000369977 // MYO6 // my	3,59	LUMINAL A up vs CONTROL
COG5	NM_006348 // COG5 // component of oligomeric golgi complex 5 // 7q22-q31 // 1046	3,59	LUMINAL A up vs CONTROL
LY96	NM_015364 // LY96 // lymphocyte antigen 96 // 8q21.11 // 23643 /// ENST000002848	3,59	LUMINAL A up vs CONTROL
EIF3H	NM_003756 // EIF3H // eukaryotic translation initiation factor 3, subunit H // 8	3,59	LUMINAL A up vs CONTROL
NUP155	NM_153485 // NUP155 // nucleoporin 155kDa // 5p13.1 // 9631 /// NM_004298 // NUP	3,58	LUMINAL A up vs CONTROL
LEPROTL1	NM_015344 // LEPROTL1 // leptin receptor overlapping transcript-like 1 // 8p21.2	3,56	LUMINAL A up vs CONTROL
ALG8	NM_024079 // ALG8 // asparagine-linked glycosylation 8, alpha-1,3-glucosyltransf	3,56	LUMINAL A up vs CONTROL
EPRS	NM_004446 // EPRS // glutamyl-prolyl-tRNA synthetase // 1q41-q42 // 2058 /// ENS	3,56	LUMINAL A up vs CONTROL
IL2RG	NM_000206 // IL2RG // interleukin 2 receptor, gamma (severe combined immunodefic	3,56	LUMINAL A up vs CONTROL
SERBP1	NM_001018067 // SERBP1 // SERPINE1 mRNA binding protein 1 // 1p31 // 26135 /// N	3,55	LUMINAL A up vs CONTROL
XPO1	NM_003400 // XPO1 // exportin 1 (CRM1 homolog, yeast) // 2p16 // 7514 /// ENST00	3,55	LUMINAL A up vs CONTROL
EXOC2	NM_018303 // EXOC2 // exocyst complex component 2 // 6p25.3 // 55770 /// ENST000	3,55	LUMINAL A up vs CONTROL
HSD17B7P2	NR_003086 // HSD17B7P2 // hydroxysteroid (17-beta) dehydrogenase 7 pseudogene 2	3,54	LUMINAL A up vs CONTROL
TLR6	NM_006068 // TLR6 // toll-like receptor 6 // 4p14 // 10333 /// ENST00000381950 /	3,54	LUMINAL A up vs CONTROL
RARS	NM_002887 // RARS // arginyl-tRNA synthetase // 5q35.1 // 5917 /// ENST000002315	3,54	LUMINAL A up vs CONTROL
HGF	NM_000601 // HGF // hepatocyte growth factor (hepapoietin A; scatter factor) //	3,54	LUMINAL A up vs CONTROL
PRRC1	NM_130809 // PRRC1 // proline-rich coiled-coil 1 // 5q23.2 // 133619 /// ENST000	3,53	LUMINAL A up vs CONTROL
DCTN5	NM_032486 // DCTN5 // dynactin 5 (p25) // 16p12.2 // 84516 /// ENST00000300087 /	3,53	LUMINAL A up vs CONTROL
TMEM87B	NM_032824 // TMEM87B // transmembrane protein 87B // 2q13 // 84910 /// ENST00000	3,53	LUMINAL A up vs CONTROL
PIGU	NM_080476 // PIGU // phosphatidylinositol glycan anchor biosynthesis, class U //	3,52	LUMINAL A up vs CONTROL

C6orf48	NM_001040437 // C6orf48 // chromosome 6 open reading frame 48 // 6p21.3 // 50854	3,52	LUMINAL A up vs CONTROL
PSMA2	NM_002787 // PSMA2 // proteasome (prosome, macropain) subunit, alpha type, 2 //	3,52	LUMINAL A up vs CONTROL
RNF103	NM_005667 // RNF103 // ring finger protein 103 // 2p11.2 // 7844 /// ENST0000023	3,52	LUMINAL A up vs CONTROL
KIAA1033	NM_015275 // KIAA1033 // KIAA1033 // 12q24.11 // 23325 /// ENST00000332180 // KI	3,52	LUMINAL A up vs CONTROL
SRP54	NM_003136 // SRP54 // signal recognition particle 54kDa // 14q13.2 // 6729 /// N	3,51	LUMINAL A up vs CONTROL
LMBRD1	NM_018368 // LMBRD1 // LMBR1 domain containing 1 // 6q13 // 55788 /// NM_002520	3,51	LUMINAL A up vs CONTROL
UQCRQ	NM_014402 // UQCRQ // ubiquinol-cytochrome c reductase, complex III subunit VII,	3,50	LUMINAL A up vs CONTROL
TAS2R50	NM_176890 // TAS2R50 // taste receptor, type 2, member 50 // 12p13.2 // 259296 /	3,50	LUMINAL A up vs CONTROL
HSP90AA1	NM_005348 // HSP90AA1 // heat shock protein 90kDa alpha (cytosolic), class A mem	3,50	LUMINAL A up vs CONTROL
GPR183	NM_004951 // GPR183 // G protein-coupled receptor 183 // 13q32.3 // 1880 /// ENS	3,50	LUMINAL A up vs CONTROL
CBWD3	NM_201453 // CBWD3 // COBW domain containing 3 // 9q13 // 445571 /// NM_00102491	3,50	LUMINAL A up vs CONTROL
FCGR2A	NM_001136219 // FCGR2A // Fc fragment of IgG, low affinity IIa, receptor (CD32)	3,50	LUMINAL A up vs CONTROL
SAMHD1	NM_015474 // SAMHD1 // SAM domain and HD domain 1 // 20pter-q12 // 25939 /// ENS	3,50	LUMINAL A up vs CONTROL
PRDX1	NM_002574 // PRDX1 // peroxiredoxin 1 // 1p34.1 // 5052 /// NM_181696 // PRDX1 /	3,49	LUMINAL A up vs CONTROL
ABCD3	NM_002858 // ABCD3 // ATP-binding cassette, sub-family D (ALD), member 3 // 1p22	3,49	LUMINAL A up vs CONTROL
C1orf103	NM_018372 // C1orf103 // chromosome 1 open reading frame 103 // 1p13.3 // 55791	3,49	LUMINAL A up vs CONTROL
COL6A3	NM_004369 // COL6A3 // collagen, type VI, alpha 3 // 2q37 // 1293 /// NM_057167	3,49	LUMINAL A up vs CONTROL
TXNDC12	NM_015913 // TXNDC12 // thioredoxin domain containing 12 (endoplasmic reticulum)	3,48	LUMINAL A up vs CONTROL
VANGL1	NM_138959 // VANGL1 // vang-like 1 (van gogh, Drosophila) // 1p11-p13.1 // 81839	3,48	LUMINAL A up vs CONTROL
FGD6	NM_018351 // FGD6 // FYVE, RhoGEF and PH domain containing 6 // 12q22 // 55785 /	3,47	LUMINAL A up vs CONTROL
CTHRC1	NM_138455 // CTHRC1 // collagen triple helix repeat containing 1 // 8q22.3 // 11	3,46	LUMINAL A up vs CONTROL
SC5DL	NM_006918 // SC5DL // sterol-C5-desaturase (ERG3 delta-5-desaturase homolog, S.	3,46	LUMINAL A up vs CONTROL
C6orf115	NM_021243 // C6orf115 // chromosome 6 open reading frame 115 // 6q24.1 // 58527	3,46	LUMINAL A up vs CONTROL
EPT1	NM_033505 // EPT1 // ethanolaminephosphotransferase 1 (CDP-ethanolamine-specific	3,46	LUMINAL A up vs CONTROL
AHR	NM_001621 // AHR // aryl hydrocarbon receptor // 7p15 // 196 /// ENST00000401808	3,45	LUMINAL A up vs CONTROL
COX16	NM_016468 // COX16 // COX16 cytochrome c oxidase assembly homolog (S. cerevisiae	3,44	LUMINAL A up vs CONTROL
NDUFA5	NM_005000 // NDUFA5 // NADH dehydrogenase (ubiquinone) 1 alpha subcomplex, 5, 13	3,44	LUMINAL A up vs CONTROL
DERL2	NM_016041 // DERL2 // Der1-like domain family, member 2 // 17p13.2 // 51009 ///	3,43	LUMINAL A up vs CONTROL
NPC2	NM_006432 // NPC2 // Niemann-Pick disease, type C2 // 14q24.3 // 10577 /// NM_00	3,42	LUMINAL A up vs CONTROL
C14orf135	NM_022495 // C14orf135 // chromosome 14 open reading frame 135 // 14q23.1 // 644	3,42	LUMINAL A up vs CONTROL
CLDN7	NM_001307 // CLDN7 // claudin 7 // 17p13 // 1366 /// ENST00000360325 // CLDN7 //	3,42	LUMINAL A up vs CONTROL
ENPP5	NM_021572 // ENPP5 // ectonucleotide pyrophosphatase/phosphodiesterase 5 (putati	3,41	LUMINAL A up vs CONTROL
CLCN3	NM_173872 // CLCN3 // chloride channel 3 // 4q33 // 1182 /// NM_001829 // CLCN3	3,41	LUMINAL A up vs CONTROL

BGN	NM_001711 // BGN // biglycan // Xq28 // 633 /// ENST00000331595 // BGN // biglyc	3,41	LUMINAL A up vs CONTROL
GFPT1	NM_002056 // GFPT1 // glutamine--fructose-6-phosphate transaminase 1 // 2p13 //	3,40	LUMINAL A up vs CONTROL
ATP2B1	NM_001001323 // ATP2B1 // ATPase, Ca++ transporting, plasma membrane 1 // 12q21.	3,40	LUMINAL A up vs CONTROL
NCOA3	NM_181659 // NCOA3 // nuclear receptor coactivator 3 // 20q12 // 8202 /// NM_006	3,40	LUMINAL A up vs CONTROL
C1orf43	NM_001098616 // C1orf43 // chromosome 1 open reading frame 43 // 1q21.2 // 25912	3,39	LUMINAL A up vs CONTROL
DTL	NM_016448 // DTL // denticleless homolog (Drosophila) // 1q32.1-q32.2 // 51514 /	3,39	LUMINAL A up vs CONTROL
RAB1A	NM_004161 // RAB1A // RAB1A, member RAS oncogene family // 2p14 // 5861 /// NM_0	3,39	LUMINAL A up vs CONTROL
PICALM	NM_007166 // PICALM // phosphatidylinositol binding clathrin assembly protein //	3,39	LUMINAL A up vs CONTROL
FXR1	NM_001013439 // FXR1 // fragile X mental retardation, autosomal homolog 1 // 3q2	3,38	LUMINAL A up vs CONTROL
YWHAH	NM_003405 // YWHAH // tyrosine 3-monooxygenase/tryptophan 5-monooxygenase activa	3,38	LUMINAL A up vs CONTROL
ERBB4	NM_005235 // ERBB4 // v-erb-a erythroblastic leukemia viral oncogene homolog 4 (	3,38	LUMINAL A up vs CONTROL
TMEM106B	NM_018374 // TMEM106B // transmembrane protein 106B // 7p21.3 // 54664 /// NM_00	3,38	LUMINAL A up vs CONTROL
KDELR3	NM_006855 // KDELR3 // KDEL (Lys-Asp-Glu-Leu) endoplasmic reticulum protein rete	3,38	LUMINAL A up vs CONTROL
C1orf27	NM_017847 // C1orf27 // chromosome 1 open reading frame 27 // 1q25 // 54953 ///	3,38	LUMINAL A up vs CONTROL
HMGCR	NM_000859 // HMGCR // 3-hydroxy-3-methylglutaryl-CoA reductase // 5q13.3-q14 //	3,37	LUMINAL A up vs CONTROL
LAPTM4B	NM_018407 // LAPTM4B // lysosomal protein transmembrane 4 beta // 8q22.1 // 5535	3,37	LUMINAL A up vs CONTROL
ATL3	ENST00000398868 // ATL3 // atlastin GTPase 3 // 11q12.3-q13.1 // 25923 /// AK090	3,37	LUMINAL A up vs CONTROL
MMP13	NM_002427 // MMP13 // matrix metallopeptidase 13 (collagenase 3) // 11q22.3 // 4	3,37	LUMINAL A up vs CONTROL
SERP1	NM_014445 // SERP1 // stress-associated endoplasmic reticulum protein 1 // 3q25.	3,37	LUMINAL A up vs CONTROL
BCLAF1	NM_014739 // BCLAF1 // BCL2-associated transcription factor 1 // 6q22-q23 // 977	3,37	LUMINAL A up vs CONTROL
OAS2	NM_002535 // OAS2 // 2'-5'-oligoadenylate synthetase 2, 69/71kDa // 12q24.2 // 4	3,37	LUMINAL A up vs CONTROL
USP53	NM_019050 // USP53 // ubiquitin specific peptidase 53 // 4q26 // 54532 /// ENST0	3,37	LUMINAL A up vs CONTROL
15/set	NM_004261 // SEP15 // 15 kDa selenoprotein // 1p31 // 9403 /// NM_203341 // SEP1	3,36	LUMINAL A up vs CONTROL
COL1A1	NM_000088 // COL1A1 // collagen, type I, alpha 1 // 17q21.33 // 1277 /// ENST000	3,36	LUMINAL A up vs CONTROL
PDCD4	NM_145341 // PDCD4 // programmed cell death 4 (neoplastic transformation inhibit	3,36	LUMINAL A up vs CONTROL
PSMC2	NM_002803 // PSMC2 // proteasome (prosome, macropain) 26S subunit, ATPase, 2 //	3,36	LUMINAL A up vs CONTROL
MUC1	NM_002456 // MUC1 // mucin 1, cell surface associated // 1q21 // 4582 /// NM_001	3,34	LUMINAL A up vs CONTROL
GJB2	NM_004004 // GJB2 // gap junction protein, beta 2, 26kDa // 13q11-q12 // 2706 //	3,33	LUMINAL A up vs CONTROL
EIF2AK2	NM_002759 // EIF2AK2 // eukaryotic translation initiation factor 2-alpha kinase	3,33	LUMINAL A up vs CONTROL
SEC61G	NM_014302 // SEC61G // Sec61 gamma subunit // 7p11.2 // 23480 /// NM_001012456 /	3,33	LUMINAL A up vs CONTROL
C5orf51	NM_175921 // C5orf51 // chromosome 5 open reading frame 51 // 5p13.1 // 285636 /	3,32	LUMINAL A up vs CONTROL
DCAF10	NM_024345 // DCAF10 // DDB1 and CUL4 associated factor 10 // 9p13.2 // 79269 ///	3,32	LUMINAL A up vs CONTROL
ABCA12	NM_173076 // ABCA12 // ATP-binding cassette, sub-family A (ABC1), member 12 // 2	3,32	LUMINAL A up vs CONTROL

PGM2	NM_018290 // PGM2 // phosphoglucomutase 2 // 4p14 // 55276 /// NM_000982 // RPL2	3,32	LUMINAL A up vs CONTROL
PLXNC1	NM_005761 // PLXNC1 // plexin C1 // 12q23.3 // 10154 /// ENST00000258526 // PLXN	3,32	LUMINAL A up vs CONTROL
SCYL2	NM_017988 // SCYL2 // SCY1-like 2 (S. cerevisiae) // 12q23.1 // 55681 /// ENST00	3,32	LUMINAL A up vs CONTROL
TOB1	NM_005749 // TOB1 // transducer of ERBB2, 1 // 17q21 // 10140 /// ENST0000026895	3,32	LUMINAL A up vs CONTROL
SLC30A6	NM_017964 // SLC30A6 // solute carrier family 30 (zinc transporter), member 6 //	3,32	LUMINAL A up vs CONTROL
CD46	NM_002389 // CD46 // CD46 molecule, complement regulatory protein // 1q32 // 417	3,31	LUMINAL A up vs CONTROL
ATP6V0E1	NM_003945 // ATP6V0E1 // ATPase, H+ transporting, lysosomal 9kDa, V0 subunit e1	3,31	LUMINAL A up vs CONTROL
NDRG3	NM_032013 // NDRG3 // NDRG family member 3 // 20q11.21-q11.23 // 57446 /// NM_02	3,31	LUMINAL A up vs CONTROL
TMBIM6	NM_001098576 // TMBIM6 // transmembrane BAX inhibitor motif containing 6 // 12q1	3,30	LUMINAL A up vs CONTROL
UBE2K	NM_005339 // UBE2K // ubiquitin-conjugating enzyme E2K (UBC1 homolog, yeast) //	3,30	LUMINAL A up vs CONTROL
RRM2	NM_001165931 // RRM2 // ribonucleotide reductase M2 // 2p25-p24 // 6241 /// NM_0	3,30	LUMINAL A up vs CONTROL
IVNS1ABP	NM_006469 // IVNS1ABP // influenza virus NS1A binding protein // 1q25.1-q31.1 //	3,30	LUMINAL A up vs CONTROL
HIAT1	NM_033055 // HIAT1 // hippocampus abundant transcript 1 // 1p21.2 // 64645 /// E	3,29	LUMINAL A up vs CONTROL
YME1L1	NM_139312 // YME1L1 // YME1-like 1 (S. cerevisiae) // 10p14 // 10730 /// NM_0142	3,29	LUMINAL A up vs CONTROL
RASGRP1	NM_005739 // RASGRP1 // RAS guanyl releasing protein 1 (calcium and DAG-regulate	3,29	LUMINAL A up vs CONTROL
SYT1	NM_001135805 // SYT1 // synaptotagmin I // 12cen-q21 // 6857 /// NM_005639 // SY	3,29	LUMINAL A up vs CONTROL
HIST1H2AC	NM_003512 // HIST1H2AC // histone cluster 1, H2ac // 6p21.3 // 8334 /// ENST0000	3,28	LUMINAL A up vs CONTROL
VPS35	NM_018206 // VPS35 // vacuolar protein sorting 35 homolog (S. cerevisiae) // 16q	3,28	LUMINAL A up vs CONTROL
TP53INP1	NM_033285 // TP53INP1 // tumor protein p53 inducible nuclear protein 1 // 8q22 /	3,28	LUMINAL A up vs CONTROL
PEX13	NM_002618 // PEX13 // peroxisomal biogenesis factor 13 // 2p14-p16 // 5194 /// E	3,28	LUMINAL A up vs CONTROL
HPS3	NM_032383 // HPS3 // Hermansky-Pudlak syndrome 3 // 3q24 // 84343 /// ENST000002	3,27	LUMINAL A up vs CONTROL
WDR75	NM_032168 // WDR75 // WD repeat domain 75 // 2q32.2 // 84128 /// ENST00000314761	3,27	LUMINAL A up vs CONTROL
CEPT1	NM_006090 // CEPT1 // choline/ethanolamine phosphotransferase 1 // 1p13.3 // 103	3,27	LUMINAL A up vs CONTROL
RAB25	NM_020387 // RAB25 // RAB25, member RAS oncogene family // 1q22 // 57111 /// ENS	3,27	LUMINAL A up vs CONTROL
TMEM106C	NM_001143842 // TMEM106C // transmembrane protein 106C // 12q13.1 // 79022 /// N	3,27	LUMINAL A up vs CONTROL
ST6GALNAC5	NM_030965 // ST6GALNAC5 // ST6 (alpha-N-acetyl-neuraminyl-2,3-beta-galactosyl-1,	3,27	LUMINAL A up vs CONTROL
THBS2	NM_003247 // THBS2 // thrombospondin 2 // 6q27 // 7058 /// ENST00000366787 // TH	3,26	LUMINAL A up vs CONTROL
IFIT1	NM_001548 // IFIT1 // interferon-induced protein with tetratricopeptide repeats	3,26	LUMINAL A up vs CONTROL
SRP72	NM_006947 // SRP72 // signal recognition particle 72kDa // 4q11 // 6731 /// ENST	3,26	LUMINAL A up vs CONTROL
BNIP3	NM_004052 // BNIP3 // BCL2/adenovirus E1B 19kDa interacting protein 3 // 10q26.3	3,26	LUMINAL A up vs CONTROL
PJA2	NM_014819 // PJA2 // praja ring finger 2 // 5q21.3 // 9867 /// ENST00000361189 /	3,26	LUMINAL A up vs CONTROL
NBN	NM_002485 // NBN // nibrin // 8q21 // 4683 /// ENST00000265433 // NBN // nibrin	3,26	LUMINAL A up vs CONTROL
FPGT	NM_003838 // FPGT // fucose-1-phosphate guanylyltransferase // 1p31.1 // 8790 //	3,25	LUMINAL A up vs CONTROL

INTS8	NM_017864 // INTS8 // integrator complex subunit 8 // 8q22.1 // 55656 /// NM_057	3,25	LUMINAL A up vs CONTROL
SEC22B	NM_004892 // SEC22B // SEC22 vesicle trafficking protein homolog B (S. cerevisia	3,25	LUMINAL A up vs CONTROL
VEZT	NM_017599 // VEZT // vezatin, adherens junctions transmembrane protein // 12q22	3,25	LUMINAL A up vs CONTROL
ATP6V1B2	NM_001693 // ATP6V1B2 // ATPase, H+ transporting, lysosomal 56/58kDa, V1 subunit	3,25	LUMINAL A up vs CONTROL
GFRA1	NM_005264 // GFRA1 // GDNF family receptor alpha 1 // 10q26.11 // 2674 /// NM_00	3,24	LUMINAL A up vs CONTROL
PSMD14	NM_005805 // PSMD14 // proteasome (prosome, macropain) 26S subunit, non-ATPase,	3,24	LUMINAL A up vs CONTROL
CEP170	NM_014812 // CEP170 // centrosomal protein 170kDa // 1q44 // 9859 /// NM_0010424	3,24	LUMINAL A up vs CONTROL
CSNK1A1	NM_001025105 // CSNK1A1 // casein kinase 1, alpha 1 // 5q32 // 1452 /// NM_00189	3,24	LUMINAL A up vs CONTROL
RBBP9	NM_006606 // RBBP9 // retinoblastoma binding protein 9 // 20p11.2 // 10741 /// E	3,24	LUMINAL A up vs CONTROL
06/mar	NM_005885 // MARCH6 // membrane-associated ring finger (C3HC4) 6 // 5p15.2 // 10	3,24	LUMINAL A up vs CONTROL
COL12A1	NM_004370 // COL12A1 // collagen, type XII, alpha 1 // 6q12-q13 // 1303 /// NM_0	3,24	LUMINAL A up vs CONTROL
CYP2B6	NM_000767 // CYP2B6 // cytochrome P450, family 2, subfamily B, polypeptide 6 //	3,24	LUMINAL A up vs CONTROL
ERBB3	NM_001982 // ERBB3 // v-erb-b2 erythroblastic leukemia viral oncogene homolog 3	3,24	LUMINAL A up vs CONTROL
F13A1	NM_000129 // F13A1 // coagulation factor XIII, A1 polypeptide // 6p25.3-p24.3 //	3,23	LUMINAL A up vs CONTROL
GCFC1	NM_016631 // GCFC1 // GC-rich sequence DNA-binding factor 1 // 21q21.3 // 94104	3,23	LUMINAL A up vs CONTROL
EIF1AX	NM_001412 // EIF1AX // eukaryotic translation initiation factor 1A, X-linked //	3,22	LUMINAL A up vs CONTROL
FUT8	NM_178155 // FUT8 // fucosyltransferase 8 (alpha (1,6) fucosyltransferase) // 14	3,22	LUMINAL A up vs CONTROL
LYSMD3	NM_198273 // LYSMD3 // LysM, putative peptidoglycan-binding, domain containing 3	3,22	LUMINAL A up vs CONTROL
SUDS3	NM_022491 // SUDS3 // suppressor of defective silencing 3 homolog (S. cerevisiae	3,22	LUMINAL A up vs CONTROL
VRK2	NM_001130483 // VRK2 // vaccinia related kinase 2 // 2p16-p15 // 7444 /// NM_001	3,21	LUMINAL A up vs CONTROL
MGAT4A	NM_012214 // MGAT4A // mannosyl (alpha-1,3-)-glycoprotein beta-1,4-N-acetylgluco	3,21	LUMINAL A up vs CONTROL
GPATCH2	NM_018040 // GPATCH2 // G patch domain containing 2 // 1q41 // 55105 /// ENST000	3,20	LUMINAL A up vs CONTROL
SHOC2	NM_007373 // SHOC2 // soc-2 suppressor of clear homolog (C. elegans) // 10q25 //	3,20	LUMINAL A up vs CONTROL
RAB2A	NM_002865 // RAB2A // RAB2A, member RAS oncogene family // 8q12.1 // 5862 /// EN	3,20	LUMINAL A up vs CONTROL
LAMP2	NM_002294 // LAMP2 // lysosomal-associated membrane protein 2 // Xq24 // 3920 //	3,20	LUMINAL A up vs CONTROL
PRPF40A	NM_017892 // PRPF40A // PRP40 pre-mRNA processing factor 40 homolog A (S. cerevi	3,20	LUMINAL A up vs CONTROL
F2R	NM_001992 // F2R // coagulation factor II (thrombin) receptor // 5q13 // 2149 //	3,20	LUMINAL A up vs CONTROL
INTS7	NM_015434 // INTS7 // integrator complex subunit 7 // 1p36.13-q42.3 // 25896 ///	3,19	LUMINAL A up vs CONTROL
HIF1A	NM_001530 // HIF1A // hypoxia inducible factor 1, alpha subunit (basic helix-loo	3,18	LUMINAL A up vs CONTROL
TMEM9B	NM_020644 // TMEM9B // TMEM9 domain family, member B // 11p15.3 // 56674 /// ENS	3,18	LUMINAL A up vs CONTROL
CLIC4	NM_013943 // CLIC4 // chloride intracellular channel 4 // 1p36.11 // 25932 /// E	3,18	LUMINAL A up vs CONTROL
SLC25A32	NM_030780 // SLC25A32 // solute carrier family 25, member 32 // 8q22.3 // 81034	3,18	LUMINAL A up vs CONTROL
DCAF16	NM_017741 // DCAF16 // DDB1 and CUL4 associated factor 16 // 4p15.31 // 54876 //	3,17	LUMINAL A up vs CONTROL

FAM36A	NM_198076 // FAM36A // family with sequence similarity 36, member A // 1q44 // 1	3,17	LUMINAL A up vs CONTROL
BET1	NM_005868 // BET1 // blocked early in transport 1 homolog (S. cerevisiae) // 7q2	3,17	LUMINAL A up vs CONTROL
DYNLT3	NM_006520 // DYNLT3 // dynein, light chain, Tctex-type 3 // Xp21 // 6990 /// ENS	3,17	LUMINAL A up vs CONTROL
RPL31	NM_001099693 // RPL31 // ribosomal protein L31 // 2q11.2 // 6160 /// NM_00109857	3,17	LUMINAL A up vs CONTROL
RAB30	NM_014488 // RAB30 // RAB30, member RAS oncogene family // 11q12-q14 // 27314 //	3,16	LUMINAL A up vs CONTROL
LMAN1	NM_005570 // LMAN1 // lectin, mannose-binding, 1 // 18q21.3-q22 // 3998 /// ENST	3,16	LUMINAL A up vs CONTROL
TTC35	NM_014673 // TTC35 // tetratricopeptide repeat domain 35 // 8q23.1 // 9694 /// E	3,16	LUMINAL A up vs CONTROL
CCT2	NM_006431 // CCT2 // chaperonin containing TCP1, subunit 2 (beta) // 12q15 // 10	3,16	LUMINAL A up vs CONTROL
TMEM123	NM_052932 // TMEM123 // transmembrane protein 123 // 11q22.1 // 114908 /// ENST0	3,16	LUMINAL A up vs CONTROL
USMG5	NM_032747 // USMG5 // up-regulated during skeletal muscle growth 5 homolog (mous	3,16	LUMINAL A up vs CONTROL
MFAP3	NR_024152 // MFAP3 // microfibrillar-associated protein 3 // 5q32-q33.2 // 4238	3,16	LUMINAL A up vs CONTROL
CMAS	NM_018686 // CMAS // cytidine monophosphate N-acetylneuraminic acid synthetase /	3,15	LUMINAL A up vs CONTROL
RPL15	NM_002948 // RPL15 // ribosomal protein L15 // 3p24.2 // 6138 /// ENST0000041369	3,15	LUMINAL A up vs CONTROL
COPA	NM_001098398 // COPA // coatomer protein complex, subunit alpha // 1q23-q25 // 1	3,15	LUMINAL A up vs CONTROL
SERINC3	NM_006811 // SERINC3 // serine incorporator 3 // 20q13.1-q13.3 // 10955 /// NM_1	3,13	LUMINAL A up vs CONTROL
GBP5	NM_052942 // GBP5 // guanylate binding protein 5 // 1p22.2 // 115362 /// NM_0011	3,13	LUMINAL A up vs CONTROL
MRPL42	NM_014050 // MRPL42 // mitochondrial ribosomal protein L42 // 12q22 // 28977 ///	3,13	LUMINAL A up vs CONTROL
FBN1	NM_000138 // FBN1 // fibrillin 1 // 15q21.1 // 2200 /// ENST00000316623 // FBN1	3,13	LUMINAL A up vs CONTROL
UBL3	NM_007106 // UBL3 // ubiquitin-like 3 // 13q12-q13 // 5412 /// ENST00000380680 /	3,13	LUMINAL A up vs CONTROL
NCAM2	NM_004540 // NCAM2 // neural cell adhesion molecule 2 // 21q21.1 // 4685 /// ENS	3,12	LUMINAL A up vs CONTROL
GALNT1	NM_020474 // GALNT1 // UDP-N-acetyl-alpha-D-galactosamine:polypeptide N-acetylga	3,12	LUMINAL A up vs CONTROL
NDUFB5	NM_002492 // NDUFB5 // NADH dehydrogenase (ubiquinone) 1 beta subcomplex, 5, 16k	3,11	LUMINAL A up vs CONTROL
PDE7A	NM_002603 // PDE7A // phosphodiesterase 7A // 8q13 // 5150 /// NM_002604 // PDE7	3,11	LUMINAL A up vs CONTROL
YWHAB	NM_003404 // YWHAB // tyrosine 3-monooxygenase/tryptophan 5-monooxygenase activa	3,10	LUMINAL A up vs CONTROL
CMTM6	NM_017801 // CMTM6 // CKLF-like MARVEL transmembrane domain containing 6 // 3p22	3,10	LUMINAL A up vs CONTROL
GPNMB	NM_001005340 // GPNMB // glycoprotein (transmembrane) nmb // 7p15 // 10457 /// N	3,10	LUMINAL A up vs CONTROL
PIGN	NM_176787 // PIGN // phosphatidylinositol glycan anchor biosynthesis, class N //	3,10	LUMINAL A up vs CONTROL
C9orf150	NM_203403 // C9orf150 // chromosome 9 open reading frame 150 // 9p23 // 286343 /	3,10	LUMINAL A up vs CONTROL
PGGT1B	NM_005023 // PGGT1B // protein geranylgeranyltransferase type I, beta subunit //	3,10	LUMINAL A up vs CONTROL
STXBP3	NM_007269 // STXBP3 // syntaxin binding protein 3 // 1p13.3 // 6814 /// ENST0000	3,09	LUMINAL A up vs CONTROL
YIPF5	NM_001024947 // YIPF5 // Yip1 domain family, member 5 // 5q31.3 // 81555 /// NM_	3,09	LUMINAL A up vs CONTROL
KLHL28	NM_017658 // KLHL28 // kelch-like 28 (Drosophila) // 14q21.2 // 54813 /// ENST00	3,09	LUMINAL A up vs CONTROL
KDM5B	NM_006618 // KDM5B // lysine (K)-specific demethylase 5B // 1q32.1 // 10765 ///	3,09	LUMINAL A up vs CONTROL

MSR1	NM_002445 // MSR1 // macrophage scavenger receptor 1 // 8p22 // 4481 /// NM_1387	3,09	LUMINAL A up vs CONTROL
PRKAG1	NM_212461 // PRKAG1 // protein kinase, AMP-activated, gamma 1 non-catalytic subu	3,08	LUMINAL A up vs CONTROL
RPE	NM_199229 // RPE // ribulose-5-phosphate-3-epimerase // 2q32-q33.3 // 6120 /// N	3,08	LUMINAL A up vs CONTROL
NPEPPS	NM_006310 // NPEPPS // aminopeptidase puromycin sensitive // 17q21 // 9520 /// E	3,08	LUMINAL A up vs CONTROL
PIGM	NM_145167 // PIGM // phosphatidylinositol glycan anchor biosynthesis, class M //	3,08	LUMINAL A up vs CONTROL
CTGF	NM_001901 // CTGF // connective tissue growth factor // 6q23.1 // 1490 /// ENST0	3,08	LUMINAL A up vs CONTROL
MACC1	NM_182762 // MACC1 // metastasis associated in colon cancer 1 // 7p21.1 // 34638	3,08	LUMINAL A up vs CONTROL
BLZF1	NM_003666 // BLZF1 // basic leucine zipper nuclear factor 1 // 1q24 // 8548 ///	3,08	LUMINAL A up vs CONTROL
ADSS	NM_001126 // ADSS // adenylosuccinate synthase // 1cen-q12 // 159 /// ENST000003	3,08	LUMINAL A up vs CONTROL
TMEM209	NM_032842 // TMEM209 // transmembrane protein 209 // 7q32.2 // 84928 /// ENST000	3,08	LUMINAL A up vs CONTROL
BUB3	NM_004725 // BUB3 // budding uninhibited by benzimidazoles 3 homolog (yeast) //	3,08	LUMINAL A up vs CONTROL
DPM1	NM_003859 // DPM1 // dolichyl-phosphate mannosyltransferase polypeptide 1, catal	3,07	LUMINAL A up vs CONTROL
HLA-DQB2	NR_003937 // HLA-DQB2 // major histocompatibility complex, class II, DQ beta 2 /	3,07	LUMINAL A up vs CONTROL
IFI27	NM_001130080 // IFI27 // interferon, alpha-inducible protein 27 // 14q32 // 3429	3,07	LUMINAL A up vs CONTROL
IDE	NM_004969 // IDE // insulin-degrading enzyme // 10q23-q25 // 3416 /// NM_0011659	3,07	LUMINAL A up vs CONTROL
TUG1	NR_002323 // TUG1 // taurine upregulated 1 (non-protein coding) // 22q12.2 // 55	3,07	LUMINAL A up vs CONTROL
TMEM33	NM_018126 // TMEM33 // transmembrane protein 33 // 4p13 // 55161 /// NM_00102995	3,07	LUMINAL A up vs CONTROL
TPRG1	NM_198485 // TPRG1 // tumor protein p63 regulated 1 // 3q28 // 285386 /// ENST00	3,06	LUMINAL A up vs CONTROL
DIRC2	NM_032839 // DIRC2 // disrupted in renal carcinoma 2 // 3q21.1 // 84925 /// ENST	3,06	LUMINAL A up vs CONTROL
USP9X	NM_001039590 // USP9X // ubiquitin specific peptidase 9, X-linked // Xp11.4 // 8	3,06	LUMINAL A up vs CONTROL
NSF	NM_006178 // NSF // N-ethylmaleimide-sensitive factor // 17q21 // 4905 /// ENST0	3,06	LUMINAL A up vs CONTROL
HSPA13	NM_006948 // HSPA13 // heat shock protein 70kDa family, member 13 // 21q11.1|21q	3,05	LUMINAL A up vs CONTROL
PCMT1	NM_005389 // PCMT1 // protein-L-isoaspartate (D-aspartate) O-methyltransferase /	3,05	LUMINAL A up vs CONTROL
NAP1L1	NM_004537 // NAP1L1 // nucleosome assembly protein 1-like 1 // 12q21.2 // 4673 /	3,05	LUMINAL A up vs CONTROL
SPCS1	NM_014041 // SPCS1 // signal peptidase complex subunit 1 homolog (S. cerevisiae)	3,05	LUMINAL A up vs CONTROL
C16orf61	NM_020188 // C16orf61 // chromosome 16 open reading frame 61 // 16q23.2 // 56942	3,05	LUMINAL A up vs CONTROL
VPS45	NM_007259 // VPS45 // vacuolar protein sorting 45 homolog (S. cerevisiae) // 1q2	3,04	LUMINAL A up vs CONTROL
MBTPS2	NM_015884 // MBTPS2 // membrane-bound transcription factor peptidase, site 2 //	3,04	LUMINAL A up vs CONTROL
GDAP2	NM_017686 // GDAP2 // ganglioside induced differentiation associated protein 2 /	3,04	LUMINAL A up vs CONTROL
ARHGDIB	NM_001175 // ARHGDIB // Rho GDP dissociation inhibitor (GDI) beta // 12p12.3 //	3,04	LUMINAL A up vs CONTROL
PAPD4	NM_001114394 // PAPD4 // PAP associated domain containing 4 // 5q14.1 // 167153	3,04	LUMINAL A up vs CONTROL
PMP22	NM_000304 // PMP22 // peripheral myelin protein 22 // 17p12-p11.2 // 5376 /// NM	3,04	LUMINAL A up vs CONTROL
ICOS	NM_012092 // ICOS // inducible T-cell co-stimulator // 2q33 // 29851 /// ENST000	3,03	LUMINAL A up vs CONTROL

SP1	NM_138473 // SP1 // Sp1 transcription factor // 12q13.1 // 6667 /// NM_003109 //	3,03	LUMINAL A up vs CONTROL
RAB11A	NM_004663 // RAB11A // RAB11A, member RAS oncogene family // 15q21.3-q22.31 // 8	3,03	LUMINAL A up vs CONTROL
ATP6V1A	NM_001690 // ATP6V1A // ATPase, H+ transporting, lysosomal 70kDa, V1 subunit A /	3,03	LUMINAL A up vs CONTROL
CILP	NM_003613 // CILP // cartilage intermediate layer protein, nucleotide pyrophosph	3,03	LUMINAL A up vs CONTROL
MOBKL1B	NM_018221 // MOBKL1B // MOB1, Mps One Binder kinase activator-like 1B (yeast) //	3,02	LUMINAL A up vs CONTROL
PSMB9	NM_002800 // PSMB9 // proteasome (prosome, macropain) subunit, beta type, 9 (lar	3,02	LUMINAL A up vs CONTROL
PSMA3	NM_002788 // PSMA3 // proteasome (prosome, macropain) subunit, alpha type, 3 //	3,01	LUMINAL A up vs CONTROL
ARL17A	NM_001113738 // ARL17A // ADP-ribosylation factor-like 17A // 17q21.31 // 51326	3,01	LUMINAL A up vs CONTROL
UBE2W	NM_001001481 // UBE2W // ubiquitin-conjugating enzyme E2W (putative) // 8q21.11	3,01	LUMINAL A up vs CONTROL
ADAM10	NM_001110 // ADAM10 // ADAM metallopeptidase domain 10 // 15q2|15q22 // 102 ///	3,00	LUMINAL A up vs CONTROL
TAS2R20	NM_176889 // TAS2R20 // taste receptor, type 2, member 20 // 12p13.2 // 259295 /	3,00	LUMINAL A up vs CONTROL
ENPP1	NM_006208 // ENPP1 // ectonucleotide pyrophosphatase/phosphodiesterase 1 // 6q22	3,00	LUMINAL A up vs CONTROL
SYPL1	NM_006754 // SYPL1 // synaptophysin-like 1 // 7q22.3 // 6856 /// NM_182715 // SY	3,00	LUMINAL A up vs CONTROL
LEF1	NM_016269 // LEF1 // lymphoid enhancer-binding factor 1 // 4q23-q25 // 51176 ///	3,00	LUMINAL A up vs CONTROL
MAPKSP1	NM_021970 // MAPKSP1 // MAPK scaffold protein 1 // 4q23 // 8649 /// NR_024170 //	3,00	LUMINAL A up vs CONTROL
NCKAP1L	NM_005337 // NCKAP1L // NCK-associated protein 1-like // 12q13.1 // 3071 /// ENS	3,00	LUMINAL A up vs CONTROL
C17orf87	NM_207103 // C17orf87 // chromosome 17 open reading frame 87 // 17p13.2 // 38832	2,99	LUMINAL A up vs CONTROL
EPCAM	NM_002354 // EPCAM // epithelial cell adhesion molecule // 2p21 // 4072 /// ENST	2,99	LUMINAL A up vs CONTROL
C1orf9	NM_014283 // C1orf9 // chromosome 1 open reading frame 9 // 1q24 // 51430 /// NM	2,99	LUMINAL A up vs CONTROL
HIST1H3B	NM_003537 // HIST1H3B // histone cluster 1, H3b // 6p21.3 // 8358 /// BC096133 /	2,99	LUMINAL A up vs CONTROL
SSR3	NM_007107 // SSR3 // signal sequence receptor, gamma (translocon-associated prot	2,99	LUMINAL A up vs CONTROL
SF3B1	NM_012433 // SF3B1 // splicing factor 3b, subunit 1, 155kDa // 2q33.1 // 23451 /	2,98	LUMINAL A up vs CONTROL
PARN	NM_002582 // PARN // poly(A)-specific ribonuclease (deadenylation nuclease) // 1	2,98	LUMINAL A up vs CONTROL
SFT2D2	NM_199344 // SFT2D2 // SFT2 domain containing 2 // 1q24.2 // 375035 /// ENST0000	2,98	LUMINAL A up vs CONTROL
EIF2S1	NM_004094 // EIF2S1 // eukaryotic translation initiation factor 2, subunit 1 alp	2,98	LUMINAL A up vs CONTROL
EIF4A2	NM_001967 // EIF4A2 // eukaryotic translation initiation factor 4A2 // 3q28 // 1	2,98	LUMINAL A up vs CONTROL
PRCP	NM_199418 // PRCP // prolylcarboxypeptidase (angiotensinase C) // 11q14 // 5547	2,98	LUMINAL A up vs CONTROL
GCNT1	NM_001097634 // GCNT1 // glucosaminyl (N-acetyl) transferase 1, core 2 // 9q13 /	2,97	LUMINAL A up vs CONTROL
TOP2B	NM_001068 // TOP2B // topoisomerase (DNA) II beta 180kDa // 3p24 // 7155 /// ENS	2,97	LUMINAL A up vs CONTROL
C7orf36	NM_020192 // C7orf36 // chromosome 7 open reading frame 36 // 7p14.1 // 57002 //	2,96	LUMINAL A up vs CONTROL
CALCRL	NM_005795 // CALCRL // calcitonin receptor-like // 2q32.1 // 10203 /// ENST00000	2,96	LUMINAL A up vs CONTROL
HIST1H2AL	NM_003511 // HIST1H2AL // histone cluster 1, H2al // 6p22-p21.3 // 8332 /// BC11	2,96	LUMINAL A up vs CONTROL
SPOPL	NM_001001664 // SPOPL // speckle-type POZ protein-like // 2q22.1 // 339745 /// E	2,96	LUMINAL A up vs CONTROL

STC1	NM_003155 // STC1 // stanniocalcin 1 // 8p21-p11.2 // 6781 /// ENST00000290271 /	2,95	LUMINAL A up vs CONTROL
ZNF644	NM_201269 // ZNF644 // zinc finger protein 644 // 1p22.2 // 84146 /// NM_016620	2,95	LUMINAL A up vs CONTROL
STK3	NM_006281 // STK3 // serine/threonine kinase 3 (STE20 homolog, yeast) // 8q22.2	2,95	LUMINAL A up vs CONTROL
GOT2	NM_002080 // GOT2 // glutamic-oxaloacetic transaminase 2, mitochondrial (asparta	2,95	LUMINAL A up vs CONTROL
MMADHC	NM_015702 // MMADHC // methylmalonic aciduria (cobalamin deficiency) cblD type,	2,94	LUMINAL A up vs CONTROL
SSR4	NM_006280 // SSR4 // signal sequence receptor, delta (translocon-associated prot	2,93	LUMINAL A up vs CONTROL
SELL	NM_000655 // SELL // selectin L // 1q23-q25 // 6402 /// NR_029467 // SELL // sel	2,93	LUMINAL A up vs CONTROL
SRPRB	NM_021203 // SRPRB // signal recognition particle receptor, B subunit // 3q22.1	2,93	LUMINAL A up vs CONTROL
COX7A2	NR_029466 // COX7A2 // cytochrome c oxidase subunit VIIa polypeptide 2 (liver) /	2,93	LUMINAL A up vs CONTROL
GNAI3	NM_006496 // GNAI3 // guanine nucleotide binding protein (G protein), alpha inhi	2,93	LUMINAL A up vs CONTROL
GMFG	NM_004877 // GMFG // glia maturation factor, gamma // 19q13.2 // 9535 /// ENST00	2,93	LUMINAL A up vs CONTROL
POC1B	NM_172240 // POC1B // POC1 centriolar protein homolog B (Chlamydomonas) // 12q21	2,93	LUMINAL A up vs CONTROL
KIAA0528	BC143878 // KIAA0528 // KIAA0528 // 12p12.1 // 9847 /// AK295862 // KIAA0528 //	2,93	LUMINAL A up vs CONTROL
RBBP7	NM_002893 // RBBP7 // retinoblastoma binding protein 7 // Xp22.2 // 5931 /// ENS	2,93	LUMINAL A up vs CONTROL
CRBN	NM_016302 // CRBN // cereblon // 3p26.2 // 51185 /// NM_001173482 // CRBN // cer	2,93	LUMINAL A up vs CONTROL
DMXL2	NM_001174116 // DMXL2 // Dmx-like 2 // 15q21.2 // 23312 /// NM_015263 // DMXL2 /	2,93	LUMINAL A up vs CONTROL
C6orf62	NM_030939 // C6orf62 // chromosome 6 open reading frame 62 // 6p22.3 // 81688 //	2,93	LUMINAL A up vs CONTROL
GOLGA8B	NR_027410 // GOLGA8B // golgin A8 family, member B // 15q14 // 440270 /// NM_001	2,92	LUMINAL A up vs CONTROL
ADNP	NM_015339 // ADNP // activity-dependent neuroprotector homeobox // 20q13.13 // 2	2,92	LUMINAL A up vs CONTROL
C11orf10	NM_014206 // C11orf10 // chromosome 11 open reading frame 10 // 11q12-q13.1 // 7	2,92	LUMINAL A up vs CONTROL
NSMCE2	NM_173685 // NSMCE2 // non-SMC element 2, MMS21 homolog (S. cerevisiae) // 8q24.	2,92	LUMINAL A up vs CONTROL
TYW3	NR_027962 // TYW3 // tRNA-yW synthesizing protein 3 homolog (S. cerevisiae) // 1	2,92	LUMINAL A up vs CONTROL
SLC19A2	NM_006996 // SLC19A2 // solute carrier family 19 (thiamine transporter), member	2,92	LUMINAL A up vs CONTROL
RSL24D1	NM_016304 // RSL24D1 // ribosomal L24 domain containing 1 // 15q21 // 51187 ///	2,91	LUMINAL A up vs CONTROL
MRPL45	NM_032351 // MRPL45 // mitochondrial ribosomal protein L45 // 17q21.2 // 84311 /	2,91	LUMINAL A up vs CONTROL
FAM36A	NM_198076 // FAM36A // family with sequence similarity 36, member A // 1q44 // 1	2,91	LUMINAL A up vs CONTROL
SEC23B	NM_006363 // SEC23B // Sec23 homolog B (S. cerevisiae) // 20p11.23 // 10483 ///	2,91	LUMINAL A up vs CONTROL
CDC42SE2	NM_020240 // CDC42SE2 // CDC42 small effector 2 // 5q23.3-q31.1 // 56990 /// NM_	2,91	LUMINAL A up vs CONTROL
HSP90B1	NM_003299 // HSP90B1 // heat shock protein 90kDa beta (Grp94), member 1 // 12q24	2,91	LUMINAL A up vs CONTROL
RALGAPB	NM_020336 // RALGAPB // Ral GTPase activating protein, beta subunit (non-catalyt	2,91	LUMINAL A up vs CONTROL
C4orf41	NM_021942 // C4orf41 // chromosome 4 open reading frame 41 // 4q35.1 // 60684 //	2,91	LUMINAL A up vs CONTROL
ALOX5AP	NM_001629 // ALOX5AP // arachidonate 5-lipoxygenase-activating protein // 13q12	2,91	LUMINAL A up vs CONTROL
SLC30A7	NM_133496 // SLC30A7 // solute carrier family 30 (zinc transporter), member 7 //	2,91	LUMINAL A up vs CONTROL

RAP1A	NM_001010935 // RAP1A // RAP1A, member of RAS oncogene family // 1p13.3 // 5906	2,91	LUMINAL A up vs CONTROL
MFSD8	NM_152778 // MFSD8 // major facilitator superfamily domain containing 8 // 4q28.	2,90	LUMINAL A up vs CONTROL
GDI2	NM_001494 // GDI2 // GDP dissociation inhibitor 2 // 10p15 // 2665 /// NM_001115	2,90	LUMINAL A up vs CONTROL
HEXB	NM_000521 // HEXB // hexosaminidase B (beta polypeptide) // 5q13 // 3074 /// ENS	2,90	LUMINAL A up vs CONTROL
FRS2	NM_006654 // FRS2 // fibroblast growth factor receptor substrate 2 // 12q15 // 1	2,90	LUMINAL A up vs CONTROL
PPP1CB	NM_002709 // PPP1CB // protein phosphatase 1, catalytic subunit, beta isozyme //	2,89	LUMINAL A up vs CONTROL
SLC38A9	NM_173514 // SLC38A9 // solute carrier family 38, member 9 // 5q11.2 // 153129 /	2,89	LUMINAL A up vs CONTROL
SACM1L	NM_014016 // SACM1L // SAC1 suppressor of actin mutations 1-like (yeast) // 3p21	2,88	LUMINAL A up vs CONTROL
DYNLT1	NM_006519 // DYNLT1 // dynein, light chain, Tctex-type 1 // 6q25.2-q25.3 // 6993	2,88	LUMINAL A up vs CONTROL
GRPR	NM_005314 // GRPR // gastrin-releasing peptide receptor // Xp22.2-p22.13 // 2925	2,88	LUMINAL A up vs CONTROL
IFI30	NM_006332 // IFI30 // interferon, gamma-inducible protein 30 // 19p13.1 // 10437	2,88	LUMINAL A up vs CONTROL
OTUD6B	NM_016023 // OTUD6B // OTU domain containing 6B // 8q21.3 // 51633 /// AK293843	2,88	LUMINAL A up vs CONTROL
TMED2	NM_006815 // TMED2 // transmembrane emp24 domain trafficking protein 2 // 12q24.	2,88	LUMINAL A up vs CONTROL
GLCE	NM_015554 // GLCE // glucuronic acid epimerase // 15q23 // 26035 /// ENST0000026	2,87	LUMINAL A up vs CONTROL
ZDHHC21	NM_178566 // ZDHHC21 // zinc finger, DHHC-type containing 21 // 9p22.3 // 340481	2,87	LUMINAL A up vs CONTROL
PUM2	NM_015317 // PUM2 // pumilio homolog 2 (Drosophila) // 2p22-p21 // 23369 /// ENS	2,87	LUMINAL A up vs CONTROL
CKLF	NM_016951 // CKLF // chemokine-like factor // 16q21 // 51192 /// NM_001040138 //	2,87	LUMINAL A up vs CONTROL
ARFIP1	NM_001025595 // ARFIP1 // ADP-ribosylation factor interacting protein 1 // 4q31.	2,87	LUMINAL A up vs CONTROL
KCTD3	NM_016121 // KCTD3 // potassium channel tetramerisation domain containing 3 // 1	2,87	LUMINAL A up vs CONTROL
SLC35A1	NM_006416 // SLC35A1 // solute carrier family 35 (CMP-sialic acid transporter),	2,87	LUMINAL A up vs CONTROL
YPEL5	NM_001127401 // YPEL5 // yippee-like 5 (Drosophila) // 2p23.1 // 51646 /// NM_00	2,87	LUMINAL A up vs CONTROL
HNRNPH2	NM_019597 // HNRNPH2 // heterogeneous nuclear ribonucleoprotein H2 (H') // Xq22	2,87	LUMINAL A up vs CONTROL
SEC24D	NM_014822 // SEC24D // SEC24 family, member D (S. cerevisiae) // 4q26 // 9871 //	2,87	LUMINAL A up vs CONTROL
CHMP5	NM_016410 // CHMP5 // chromatin modifying protein 5 // 9p13.3 // 51510 /// ENST0	2,86	LUMINAL A up vs CONTROL
XRCC5	NM_021141 // XRCC5 // X-ray repair complementing defective repair in Chinese ham	2,86	LUMINAL A up vs CONTROL
SCRN1	NM_014766 // SCRN1 // secernin 1 // 7p14.3-p14.1 // 9805 /// NM_001145513 // SCR	2,86	LUMINAL A up vs CONTROL
SLC30A5	NM_022902 // SLC30A5 // solute carrier family 30 (zinc transporter), member 5 //	2,86	LUMINAL A up vs CONTROL
NOX4	NM_016931 // NOX4 // NADPH oxidase 4 // 11q14.2-q21 // 50507 /// NR_026571 // NO	2,86	LUMINAL A up vs CONTROL
PSEN1	NM_000021 // PSEN1 // presenilin 1 // 14q24.3 // 5663 /// NM_007318 // PSEN1 //	2,86	LUMINAL A up vs CONTROL
SLC25A40	NM_018843 // SLC25A40 // solute carrier family 25, member 40 // 7q21.12 // 55972	2,86	LUMINAL A up vs CONTROL
XRN1	NM_019001 // XRN1 // 5'-3' exoribonuclease 1 // 3q23 // 54464 /// NM_001042604 /	2,86	LUMINAL A up vs CONTROL
PAK2	NM_002577 // PAK2 // p21 protein (Cdc42/Rac)-activated kinase 2 // 3q29 // 5062	2,85	LUMINAL A up vs CONTROL
GGPS1	NM_004837 // GGPS1 // geranylgeranyl diphosphate synthase 1 // 1q43 // 9453 ///	2,85	LUMINAL A up vs CONTROL

ARV1	NM_022786 // ARV1 // ARV1 homolog (S. cerevisiae) // 1q42.2 // 64801 /// ENST000	2,85	LUMINAL A up vs CONTROL
PAICS	NM_001079525 // PAICS // phosphoribosylaminoimidazole carboxylase, phosphoribosy	2,85	LUMINAL A up vs CONTROL
YTHDF3	NM_152758 // YTHDF3 // YTH domain family, member 3 // 8q12.3 // 253943 /// ENST0	2,85	LUMINAL A up vs CONTROL
GALNT7	NM_017423 // GALNT7 // UDP-N-acetyl-alpha-D-galactosamine:polypeptide N-acetylga	2,85	LUMINAL A up vs CONTROL
C16orf63	NM_144600 // C16orf63 // chromosome 16 open reading frame 63 // 16p13.11 // 1238	2,85	LUMINAL A up vs CONTROL
CCR1	NM_001295 // CCR1 // chemokine (C-C motif) receptor 1 // 3p21 // 1230 /// ENST00	2,85	LUMINAL A up vs CONTROL
CLEC2D	NM_001004419 // CLEC2D // C-type lectin domain family 2, member D // 12p13 // 29	2,85	LUMINAL A up vs CONTROL
GPR52	NM_005684 // GPR52 // G protein-coupled receptor 52 // 1q24 // 9293 /// ENST0000	2,84	LUMINAL A up vs CONTROL
TMEM30A	NM_018247 // TMEM30A // transmembrane protein 30A // 6q14.1 // 55754 /// NM_0011	2,84	LUMINAL A up vs CONTROL
TTC37	NM_014639 // TTC37 // tetratricopeptide repeat domain 37 // 5q15 // 9652 /// ENS	2,84	LUMINAL A up vs CONTROL
SEC31A	NM_014933 // SEC31A // SEC31 homolog A (S. cerevisiae) // 4q21.22 // 22872 /// N	2,84	LUMINAL A up vs CONTROL
C11orf58	NM_001142705 // C11orf58 // chromosome 11 open reading frame 58 // 11p15.1 // 10	2,84	LUMINAL A up vs CONTROL
UGCG	NM_003358 // UGCG // UDP-glucose ceramide glucosyltransferase // 9q31 // 7357 //	2,84	LUMINAL A up vs CONTROL
NT5C2	NM_012229 // NT5C2 // 5'-nucleotidase, cytosolic II // 10q24.32-q24.33 // 22978	2,84	LUMINAL A up vs CONTROL
DENND1B	NM_001142795 // DENND1B // DENN/MADD domain containing 1B // 1q31.3 // 163486 //	2,84	LUMINAL A up vs CONTROL
EMP2	NM_001424 // EMP2 // epithelial membrane protein 2 // 16p13.2 // 2013 /// ENST00	2,84	LUMINAL A up vs CONTROL
EZR	NM_003379 // EZR // ezrin // 6q25.2-q26 // 7430 /// NM_001111077 // EZR // ezrin	2,83	LUMINAL A up vs CONTROL
NRIP1	NM_003489 // NRIP1 // nuclear receptor interacting protein 1 // 21q11.2 // 8204	2,83	LUMINAL A up vs CONTROL
TMEM87A	NM_015497 // TMEM87A // transmembrane protein 87A // 15q15.1 // 25963 /// NM_001	2,83	LUMINAL A up vs CONTROL
GPD2	NM_001083112 // GPD2 // glycerol-3-phosphate dehydrogenase 2 (mitochondrial) //	2,83	LUMINAL A up vs CONTROL
LOC100128816	AY358109 // LOC100128816 // ACAH3104 // 12p13.32 // 100128816 /// ENST0000044386	2,83	LUMINAL A up vs CONTROL
RRM2B	NM_015713 // RRM2B // ribonucleotide reductase M2 B (TP53 inducible) // 8q23.1 /	2,83	LUMINAL A up vs CONTROL
NDST4	NM_022569 // NDST4 // N-deacetylase/N-sulfotransferase (heparan glucosaminyl) 4	2,83	LUMINAL A up vs CONTROL
VTA1	NM_016485 // VTA1 // Vps20-associated 1 homolog (S. cerevisiae) // 6q24.1 // 515	2,83	LUMINAL A up vs CONTROL
CD84	NM_003874 // CD84 // CD84 molecule // 1q24 // 8832 /// ENST00000368054 // CD84 /	2,83	LUMINAL A up vs CONTROL
TBCK	NM_001163436 // TBCK // TBC1 domain containing kinase // 4q24 // 93627 /// NM_00	2,83	LUMINAL A up vs CONTROL
ARMC1	NM_018120 // ARMC1 // armadillo repeat containing 1 // 8q13.1 // 55156 /// ENST0	2,83	LUMINAL A up vs CONTROL
CDS2	NM_003818 // CDS2 // CDP-diacylglycerol synthase (phosphatidate cytidylyltransfe	2,83	LUMINAL A up vs CONTROL
ABCC5	NM_005688 // ABCC5 // ATP-binding cassette, sub-family C (CFTR/MRP), member 5 //	2,82	LUMINAL A up vs CONTROL
SELT	NM_016275 // SELT // selenoprotein T // 3q25.1 // 51714 /// ENST00000397894 // S	2,82	LUMINAL A up vs CONTROL
C14orf1	NM_007176 // C14orf1 // chromosome 14 open reading frame 1 // 14q24.3 // 11161 /	2,82	LUMINAL A up vs CONTROL
TMBIM4	NM_016056 // TMBIM4 // transmembrane BAX inhibitor motif containing 4 // 12q14.1	2,82	LUMINAL A up vs CONTROL
RNF160	NM_015565 // RNF160 // ring finger protein 160 // 21q22.11 // 26046 /// ENST0000	2,82	LUMINAL A up vs CONTROL

RABGGTB	NM_004582 // RABGGTB // Rab geranylgeranyltransferase, beta subunit // 1p31 // 5	2,81	LUMINAL A up vs CONTROL
TRMT112	NM_016404 // TRMT112 // tRNA methyltransferase 11-2 homolog (S. cerevisiae) // 1	2,81	LUMINAL A up vs CONTROL
TMEM19	NM_018279 // TMEM19 // transmembrane protein 19 // 12q21.1 // 55266 /// ENST0000	2,81	LUMINAL A up vs CONTROL
CANX	NM_001746 // CANX // calnexin // 5q35 // 821 /// NM_001024649 // CANX // calnexi	2,81	LUMINAL A up vs CONTROL
EDNRA	NM_001957 // EDNRA // endothelin receptor type A // 4q31.22 // 1909 /// NM_00116	2,81	LUMINAL A up vs CONTROL
C20orf103	NM_012261 // C20orf103 // chromosome 20 open reading frame 103 // 20p12 // 24141	2,81	LUMINAL A up vs CONTROL
NSL1	NM_001042549 // NSL1 // NSL1, MIND kinetochore complex component, homolog (S. ce	2,81	LUMINAL A up vs CONTROL
PEBP1	NM_002567 // PEBP1 // phosphatidylethanolamine binding protein 1 // 12q24.23 //	2,81	LUMINAL A up vs CONTROL
SGK3	NM_013257 // SGK3 // serum/glucocorticoid regulated kinase family, member 3 // 8	2,80	LUMINAL A up vs CONTROL
MED14	NM_004229 // MED14 // mediator complex subunit 14 // Xp11.4-p11.2 // 9282 /// EN	2,80	LUMINAL A up vs CONTROL
ITGA4	NM_000885 // ITGA4 // integrin, alpha 4 (antigen CD49D, alpha 4 subunit of VLA-4	2,80	LUMINAL A up vs CONTROL
FGL2	NM_006682 // FGL2 // fibrinogen-like 2 // 7q11.23 // 10875 /// ENST00000248598 /	2,80	LUMINAL A up vs CONTROL
SGMS2	NM_001136258 // SGMS2 // sphingomyelin synthase 2 // 4q25 // 166929 /// NM_15262	2,80	LUMINAL A up vs CONTROL
RNF19A	NM_183419 // RNF19A // ring finger protein 19A // 8q22 // 25897 /// NM_015435 //	2,80	LUMINAL A up vs CONTROL
HLA-DMA	NM_006120 // HLA-DMA // major histocompatibility complex, class II, DM alpha //	2,80	LUMINAL A up vs CONTROL
MRPL3	NM_007208 // MRPL3 // mitochondrial ribosomal protein L3 // 3q21-q23 // 11222 //	2,80	LUMINAL A up vs CONTROL
KIAA1324	NM_020775 // KIAA1324 // KIAA1324 // 1p13.3 // 57535 /// ENST00000234923 // KIAA	2,80	LUMINAL A up vs CONTROL
RAB3GAP2	NM_012414 // RAB3GAP2 // RAB3 GTPase activating protein subunit 2 (non-catalytic	2,79	LUMINAL A up vs CONTROL
ITGB3BP	NM_014288 // ITGB3BP // integrin beta 3 binding protein (beta3-endonexin) // 1p3	2,79	LUMINAL A up vs CONTROL
GNPTAB	NM_024312 // GNPTAB // N-acetylglucosamine-1-phosphate transferase, alpha and be	2,79	LUMINAL A up vs CONTROL
CASK	NM_003688 // CASK // calcium/calmodulin-dependent serine protein kinase (MAGUK f	2,79	LUMINAL A up vs CONTROL
USP34	NM_014709 // USP34 // ubiquitin specific peptidase 34 // 2p15 // 9736 /// ENST00	2,79	LUMINAL A up vs CONTROL
M6PR	NM_002355 // M6PR // mannose-6-phosphate receptor (cation dependent) // 12p13 //	2,79	LUMINAL A up vs CONTROL
TMEM59	NM_004872 // TMEM59 // transmembrane protein 59 // 1p36-p31 // 9528 /// ENST0000	2,79	LUMINAL A up vs CONTROL
HIGD2A	NM_138820 // HIGD2A // HIG1 hypoxia inducible domain family, member 2A // 5q35.2	2,79	LUMINAL A up vs CONTROL
TIMM23	NM_006327 // TIMM23 // translocase of inner mitochondrial membrane 23 homolog (y	2,78	LUMINAL A up vs CONTROL
OMD	NM_005014 // OMD // osteomodulin // 9q22.31 // 4958 /// ENST00000375550 // OMD /	2,78	LUMINAL A up vs CONTROL
MFN1	NM_033540 // MFN1 // mitofusin 1 // 3q26.33 // 55669 /// ENST00000263969 // MFN1	2,78	LUMINAL A up vs CONTROL
MANEA	NM_024641 // MANEA // mannosidase, endo-alpha // 6q16.1 // 79694 /// ENST0000035	2,78	LUMINAL A up vs CONTROL
SLC35A3	NM_012243 // SLC35A3 // solute carrier family 35 (UDP-N-acetylglucosamine (UDP-G	2,78	LUMINAL A up vs CONTROL
SIDT1	NM_017699 // SIDT1 // SID1 transmembrane family, member 1 // 3q13.2 // 54847 ///	2,77	LUMINAL A up vs CONTROL
ABI1	NM_005470 // ABI1 // abl-interactor 1 // 10p11.2 // 10006 /// NM_001012750 // AB	2,77	LUMINAL A up vs CONTROL
ACBD3	NM_022735 // ACBD3 // acyl-CoA binding domain containing 3 // 1q42.12 // 64746 /	2,77	LUMINAL A up vs CONTROL

ZNF33A	NM_006954 // ZNF33A // zinc finger protein 33A // 10p11.2 // 7581 /// NM_006974	2,77	LUMINAL A up vs CONTROL
TMEM67	NM_153704 // TMEM67 // transmembrane protein 67 // 8q22.1 // 91147 /// NM_001142	2,77	LUMINAL A up vs CONTROL
RPF2	NM_032194 // RPF2 // ribosome production factor 2 homolog (S. cerevisiae) // 6q2	2,77	LUMINAL A up vs CONTROL
MAN2A1	NM_002372 // MAN2A1 // mannosidase, alpha, class 2A, member 1 // 5q21-q22 // 412	2,77	LUMINAL A up vs CONTROL
TMEM39A	NM_018266 // TMEM39A // transmembrane protein 39A // 3q13.33 // 55254 /// ENST00	2,77	LUMINAL A up vs CONTROL
ZNF195	NM_001130520 // ZNF195 // zinc finger protein 195 // 11p15.5 // 7748 /// NM_0011	2,77	LUMINAL A up vs CONTROL
TUBB	NM_178014 // TUBB // tubulin, beta // 6p21.33 // 203068 /// ENST00000383564 // T	2,77	LUMINAL A up vs CONTROL
ANLN	NM_018685 // ANLN // anillin, actin binding protein // 7p15-p14 // 54443 /// ENS	2,77	LUMINAL A up vs CONTROL
UBE2G1	NM_003342 // UBE2G1 // ubiquitin-conjugating enzyme E2G 1 (UBC7 homolog, yeast)	2,77	LUMINAL A up vs CONTROL
PSMA1	NM_148976 // PSMA1 // proteasome (prosome, macropain) subunit, alpha type, 1 //	2,77	LUMINAL A up vs CONTROL
PAPOLA	NM_032632 // PAPOLA // poly(A) polymerase alpha // 14q32.31 // 10914 /// ENST000	2,76	LUMINAL A up vs CONTROL
LPXN	NM_001143995 // LPXN // leupaxin // 11q12.1 // 9404 /// NM_004811 // LPXN // leu	2,76	LUMINAL A up vs CONTROL
TIMM17A	NM_006335 // TIMM17A // translocase of inner mitochondrial membrane 17 homolog A	2,76	LUMINAL A up vs CONTROL
OR52H1	NM_001005289 // OR52H1 // olfactory receptor, family 52, subfamily H, member 1 /	2,76	LUMINAL A up vs CONTROL
CD96	NM_198196 // CD96 // CD96 molecule // 3q13.13-q13.2 // 10225 /// NM_005816 // CD	2,76	LUMINAL A up vs CONTROL
TMEM97	NM_014573 // TMEM97 // transmembrane protein 97 // 17q11.2 // 27346 /// ENST0000	2,76	LUMINAL A up vs CONTROL
RSAD2	NM_080657 // RSAD2 // radical S-adenosyl methionine domain containing 2 // 2p25.	2,76	LUMINAL A up vs CONTROL
TCP1	NM_030752 // TCP1 // t-complex 1 // 6q25.3-q26 // 6950 /// NM_001008897 // TCP1	2,76	LUMINAL A up vs CONTROL
SLC35A5	NM_017945 // SLC35A5 // solute carrier family 35, member A5 // 3q13.2 // 55032 /	2,76	LUMINAL A up vs CONTROL
HSD17B11	NM_016245 // HSD17B11 // hydroxysteroid (17-beta) dehydrogenase 11 // 4q22.1 //	2,75	LUMINAL A up vs CONTROL
ATIC	NM_004044 // ATIC // 5-aminoimidazole-4-carboxamide ribonucleotide formyltransfe	2,75	LUMINAL A up vs CONTROL
ZNF141	NM_003441 // ZNF141 // zinc finger protein 141 // 4p16.3 // 7700 /// ENST0000024	2,75	LUMINAL A up vs CONTROL
ZBTB1	NM_001123329 // ZBTB1 // zinc finger and BTB domain containing 1 // 14q23.3 // 2	2,75	LUMINAL A up vs CONTROL
ENTPD1	NM_001776 // ENTPD1 // ectonucleoside triphosphate diphosphohydrolase 1 // 10q24	2,75	LUMINAL A up vs CONTROL
RECQL	NM_002907 // RECQL // RecQ protein-like (DNA helicase Q1-like) // 12p12 // 5965	2,75	LUMINAL A up vs CONTROL
LNPEP	NM_005575 // LNPEP // leucyl/cystinyl aminopeptidase // 5q15 // 4012 /// NM_1759	2,75	LUMINAL A up vs CONTROL
MGC72080	NR_002822 // MGC72080 // MGC72080 pseudogene // 7q21.3 // 389538 /// BC062733 //	2,75	LUMINAL A up vs CONTROL
ABCE1	NM_002940 // ABCE1 // ATP-binding cassette, sub-family E (OABP), member 1 // 4q3	2,75	LUMINAL A up vs CONTROL
CRABP2	NM_001878 // CRABP2 // cellular retinoic acid binding protein 2 // 1q21.3 // 138	2,75	LUMINAL A up vs CONTROL
NOP58	NM_015934 // NOP58 // NOP58 ribonucleoprotein homolog (yeast) // 2q33.1 // 51602	2,75	LUMINAL A up vs CONTROL
CSTF1	NM_001033522 // CSTF1 // cleavage stimulation factor, 3' pre-RNA, subunit 1, 50k	2,75	LUMINAL A up vs CONTROL
ERLEC1	NM_015701 // ERLEC1 // endoplasmic reticulum lectin 1 // 2p16.2 // 27248 /// NM_	2,75	LUMINAL A up vs CONTROL
IGFBP7	NM_001553 // IGFBP7 // insulin-like growth factor binding protein 7 // 4q12 // 3	2,75	LUMINAL A up vs CONTROL

ZFAND1	NR_033193 // ZFAND1 // zinc finger, AN1-type domain 1 // 8q21.13 // 79752 /// NM	2,74	LUMINAL A up vs CONTROL
ZBTB6	NM_006626 // ZBTB6 // zinc finger and BTB domain containing 6 // 9q33.2 // 10773	2,74	LUMINAL A up vs CONTROL
STAM2	NM_005843 // STAM2 // signal transducing adaptor molecule (SH3 domain and ITAM m	2,74	LUMINAL A up vs CONTROL
IARS2	NM_018060 // IARS2 // isoleucyl-tRNA synthetase 2, mitochondrial // 1q41 // 5569	2,74	LUMINAL A up vs CONTROL
RTCD1	NM_001130841 // RTCD1 // RNA terminal phosphate cyclase domain 1 // 1p21.2 // 86	2,74	LUMINAL A up vs CONTROL
ZC3H11A	NM_014827 // ZC3H11A // zinc finger CCCH-type containing 11A // 1q32.1 // 9877 /	2,74	LUMINAL A up vs CONTROL
IDO1	NM_002164 // IDO1 // indoleamine 2,3-dioxygenase 1 // 8p12-p11 // 3620 /// ENST0	2,74	LUMINAL A up vs CONTROL
BIRC6	NM_016252 // BIRC6 // baculoviral IAP repeat-containing 6 // 2p22-p21 // 57448 /	2,73	LUMINAL A up vs CONTROL
MMP2	NM_004530 // MMP2 // matrix metallopeptidase 2 (gelatinase A, 72kDa gelatinase,	2,73	LUMINAL A up vs CONTROL
NARS	NM_004539 // NARS // asparaginyl-tRNA synthetase // 18q21.2-q21.3 // 4677 /// EN	2,73	LUMINAL A up vs CONTROL
NDUFC2	NM_004549 // NDUFC2 // NADH dehydrogenase (ubiquinone) 1, subcomplex unknown, 2,	2,73	LUMINAL A up vs CONTROL
CCNB1	NM_031966 // CCNB1 // cyclin B1 // 5q12 // 891 /// ENST00000256442 // CCNB1 // c	2,73	LUMINAL A up vs CONTROL
RASSF8	NM_007211 // RASSF8 // Ras association (RalGDS/AF-6) domain family (N-terminal)	2,73	LUMINAL A up vs CONTROL
C3orf38	NM_173824 // C3orf38 // chromosome 3 open reading frame 38 // 3p11.1 // 285237 /	2,73	LUMINAL A up vs CONTROL
CETN3	NM_004365 // CETN3 // centrin, EF-hand protein, 3 (CDC31 homolog, yeast) // 5q14	2,73	LUMINAL A up vs CONTROL
CEP97	NM_024548 // CEP97 // centrosomal protein 97kDa // 3q12.3 // 79598 /// ENST00000	2,73	LUMINAL A up vs CONTROL
NDUFC1	NM_002494 // NDUFC1 // NADH dehydrogenase (ubiquinone) 1, subcomplex unknown, 1,	2,73	LUMINAL A up vs CONTROL
WDR26	NM_025160 // WDR26 // WD repeat domain 26 // 1q42.11-q42.12 // 80232 /// NM_0011	2,73	LUMINAL A up vs CONTROL
LRRC37A3	NM_199340 // LRRC37A3 // leucine rich repeat containing 37, member A3 // 17q24.1	2,73	LUMINAL A up vs CONTROL
SUZ12	NM_015355 // SUZ12 // suppressor of zeste 12 homolog (Drosophila) // 17q11.2 //	2,72	LUMINAL A up vs CONTROL
PEX2	NM_001172086 // PEX2 // peroxisomal biogenesis factor 2 // 8q21.1 // 5828 /// NM	2,72	LUMINAL A up vs CONTROL
PREPL	NM_001171606 // PREPL // prolyl endopeptidase-like // 2p21 // 9581 /// NM_006036	2,72	LUMINAL A up vs CONTROL
FAM96A	NM_032231 // FAM96A // family with sequence similarity 96, member A // 15q22.31	2,72	LUMINAL A up vs CONTROL
FAM164A	NM_016010 // FAM164A // family with sequence similarity 164, member A // 8q21.12	2,72	LUMINAL A up vs CONTROL
ZDHHC20	AK098818 // ZDHHC20 // zinc finger, DHHC-type containing 20 // 13q12.11 // 25383	2,72	LUMINAL A up vs CONTROL
ETNK1	NM_018638 // ETNK1 // ethanolamine kinase 1 // 12p12.1 // 55500 /// NM_001039481	2,72	LUMINAL A up vs CONTROL
SERINC5	NM_001174072 // SERINC5 // serine incorporator 5 // 5q14.1 // 256987 /// NM_1782	2,72	LUMINAL A up vs CONTROL
MIOS	NM_019005 // MIOS // missing oocyte, meiosis regulator, homolog (Drosophila) //	2,71	LUMINAL A up vs CONTROL
C5orf13	NM_001142481 // C5orf13 // chromosome 5 open reading frame 13 // 5q22.1 // 9315	2,71	LUMINAL A up vs CONTROL
SLMO2	NM_016045 // SLMO2 // slowmo homolog 2 (Drosophila) // 20q13.32 // 51012 /// ENS	2,71	LUMINAL A up vs CONTROL
TAB2	NM_015093 // TAB2 // TGF-beta activated kinase 1/MAP3K7 binding protein 2 // 6q2	2,71	LUMINAL A up vs CONTROL
FKBP14	NM_017946 // FKBP14 // FK506 binding protein 14, 22 kDa // 7p14.3 // 55033 /// E	2,70	LUMINAL A up vs CONTROL
METTL9	NM_016025 // METTL9 // methyltransferase like 9 // 16p13-p12 // 51108 /// NM_001	2,70	LUMINAL A up vs CONTROL

CXCL13	NM_006419 // CXCL13 // chemokine (C-X-C motif) ligand 13 // 4q21 // 10563 /// EN	2,70	LUMINAL A up vs CONTROL
C5orf28	NM_022483 // C5orf28 // chromosome 5 open reading frame 28 // 5p12 // 64417 ///	2,70	LUMINAL A up vs CONTROL
MS4A6A	NM_152852 // MS4A6A // membrane-spanning 4-domains, subfamily A, member 6A // 11	2,70	LUMINAL A up vs CONTROL
FAM91A1	NM_144963 // FAM91A1 // family with sequence similarity 91, member A1 // 8q24.13	2,70	LUMINAL A up vs CONTROL
GLT8D2	NM_031302 // GLT8D2 // glycosyltransferase 8 domain containing 2 // 12q // 83468	2,70	LUMINAL A up vs CONTROL
CROT	NM_001143935 // CROT // carnitine O-octanoyltransferase // 7q21.1 // 54677 /// N	2,69	LUMINAL A up vs CONTROL
CLDND1	NM_001040199 // CLDND1 // claudin domain containing 1 // 3q12.1 // 56650 /// NM_	2,69	LUMINAL A up vs CONTROL
ST6GALNAC2	NM_006456 // ST6GALNAC2 // ST6 (alpha-N-acetyl-neuraminyl-2,3-beta-galactosyl-1,	2,69	LUMINAL A up vs CONTROL
FRYL	NM_015030 // FRYL // FRY-like // 4p11 // 285527 /// ENST00000358350 // FRYL // F	2,69	LUMINAL A up vs CONTROL
PYROXD1	NM_024854 // PYROXD1 // pyridine nucleotide-disulphide oxidoreductase domain 1 /	2,69	LUMINAL A up vs CONTROL
TOM1L1	NM_005486 // TOM1L1 // target of myb1 (chicken)-like 1 // 17q23.2 // 10040 /// E	2,69	LUMINAL A up vs CONTROL
SLC20A1	NM_005415 // SLC20A1 // solute carrier family 20 (phosphate transporter), member	2,69	LUMINAL A up vs CONTROL
COX7A2L	NM_004718 // COX7A2L // cytochrome c oxidase subunit VIIa polypeptide 2 like //	2,69	LUMINAL A up vs CONTROL
PLXDC2	NM_032812 // PLXDC2 // plexin domain containing 2 // 10p12.31 // 84898 /// ENST0	2,69	LUMINAL A up vs CONTROL
NUDCD1	NM_001128211 // NUDCD1 // NudC domain containing 1 // 8q23 // 84955 /// NM_03286	2,69	LUMINAL A up vs CONTROL
USO1	NM_003715 // USO1 // USO1 vesicle docking protein homolog (yeast) // 4q21.1 // 8	2,69	LUMINAL A up vs CONTROL
CBX5	NM_001127322 // CBX5 // chromobox homolog 5 (HP1 alpha homolog, Drosophila) // 1	2,69	LUMINAL A up vs CONTROL
HLTF	NM_003071 // HLTF // helicase-like transcription factor // 3q25.1-q26.1 // 6596	2,69	LUMINAL A up vs CONTROL
LRRC17	NM_005824 // LRRC17 // leucine rich repeat containing 17 // 7q22.1 // 10234 ///	2,69	LUMINAL A up vs CONTROL
UTP15	NM_032175 // UTP15 // UTP15, U3 small nucleolar ribonucleoprotein, homolog (S. c	2,69	LUMINAL A up vs CONTROL
GTF3C3	NM_012086 // GTF3C3 // general transcription factor IIIC, polypeptide 3, 102kDa	2,69	LUMINAL A up vs CONTROL
ADAMDEC1	NM_014479 // ADAMDEC1 // ADAM-like, decysin 1 // 8p21.2 // 27299 /// NM_00114527	2,69	LUMINAL A up vs CONTROL
ATXN10	NM_013236 // ATXN10 // ataxin 10 // 22q13.31 // 25814 /// NM_001167621 // ATXN10	2,68	LUMINAL A up vs CONTROL
C15orf37	NR_028330 // C15orf37 // chromosome 15 open reading frame 37 // 15q25.1 // 28368	2,68	LUMINAL A up vs CONTROL
MED7	NM_001100816 // MED7 // mediator complex subunit 7 // 5q33.3 // 9443 /// NM_0042	2,68	LUMINAL A up vs CONTROL
RBM39	NM_184234 // RBM39 // RNA binding motif protein 39 // 20q11.22 // 9584 /// NM_00	2,68	LUMINAL A up vs CONTROL
RASA1	NM_002890 // RASA1 // RAS p21 protein activator (GTPase activating protein) 1 //	2,68	LUMINAL A up vs CONTROL
C1orf21	NM_030806 // C1orf21 // chromosome 1 open reading frame 21 // 1q25 // 81563 ///	2,68	LUMINAL A up vs CONTROL
INHBA	NM_002192 // INHBA // inhibin, beta A // 7p15-p13 // 3624 /// ENST00000242208 //	2,68	LUMINAL A up vs CONTROL
PTDSS1	NM_014754 // PTDSS1 // phosphatidylserine synthase 1 // 8q22 // 9791 /// ENST000	2,68	LUMINAL A up vs CONTROL
TAX1BP1	NM_006024 // TAX1BP1 // Tax1 (human T-cell leukemia virus type I) binding protei	2,68	LUMINAL A up vs CONTROL
SPINT2	NM_021102 // SPINT2 // serine peptidase inhibitor, Kunitz type, 2 // 19q13.1 //	2,68	LUMINAL A up vs CONTROL
IFIT2	NM_001547 // IFIT2 // interferon-induced protein with tetratricopeptide repeats	2,68	LUMINAL A up vs CONTROL

PAIP2	NM_001033112 // PAIP2 // poly(A) binding protein interacting protein 2 // 5q31.2	2,68	LUMINAL A up vs CONTROL
GTPBP10	NM_033107 // GTPBP10 // GTP-binding protein 10 (putative) // 7q21.13 // 85865 //	2,68	LUMINAL A up vs CONTROL
METTL14	NM_020961 // METTL14 // methyltransferase like 14 // 4q26 // 57721 /// ENST00000	2,67	LUMINAL A up vs CONTROL
MTRR	NM_002454 // MTRR // 5-methyltetrahydrofolate-homocysteine methyltransferase red	2,67	LUMINAL A up vs CONTROL
PANK3	NM_024594 // PANK3 // pantothenate kinase 3 // 5q34 // 79646 /// NR_029520 // MI	2,67	LUMINAL A up vs CONTROL
MBOAT1	NM_001080480 // MBOAT1 // membrane bound O-acyltransferase domain containing 1 /	2,67	LUMINAL A up vs CONTROL
MTFR1	NM_014637 // MTFR1 // mitochondrial fission regulator 1 // 8q13.1 // 9650 /// NM	2,67	LUMINAL A up vs CONTROL
SPCS3	NM_021928 // SPCS3 // signal peptidase complex subunit 3 homolog (S. cerevisiae)	2,67	LUMINAL A up vs CONTROL
ACADSB	NM_001609 // ACADSB // acyl-CoA dehydrogenase, short/branched chain // 10q26.13	2,67	LUMINAL A up vs CONTROL
PSMA5	NM_002790 // PSMA5 // proteasome (prosome, macropain) subunit, alpha type, 5 //	2,67	LUMINAL A up vs CONTROL
METAP1	NM_015143 // METAP1 // methionyl aminopeptidase 1 // 4q23 // 23173 /// ENST00000	2,67	LUMINAL A up vs CONTROL
DTX3L	NM_138287 // DTX3L // deltex 3-like (Drosophila) // 3q21.1 // 151636 /// ENST000	2,67	LUMINAL A up vs CONTROL
ZNF91	NM_003430 // ZNF91 // zinc finger protein 91 // 19p13.1-p12 // 7644 /// ENST0000	2,66	LUMINAL A up vs CONTROL
PFN2	NM_053024 // PFN2 // profilin 2 // 3q25.1-q25.2 // 5217 /// NM_002628 // PFN2 //	2,66	LUMINAL A up vs CONTROL
OAZ1	NM_004152 // OAZ1 // ornithine decarboxylase antizyme 1 // 19p13.3 // 4946 /// E	2,66	LUMINAL A up vs CONTROL
SELPLG	NM_003006 // SELPLG // selectin P ligand // 12q24 // 6404 /// ENST00000388962 //	2,66	LUMINAL A up vs CONTROL
CAND1	NM_018448 // CAND1 // cullin-associated and neddylation-dissociated 1 // 12q14 /	2,66	LUMINAL A up vs CONTROL
EPM2AIP1	NM_014805 // EPM2AIP1 // EPM2A (laforin) interacting protein 1 // 3p22.1 // 9852	2,66	LUMINAL A up vs CONTROL
HSPH1	NM_006644 // HSPH1 // heat shock 105kDa/110kDa protein 1 // 13q12.3 // 10808 ///	2,66	LUMINAL A up vs CONTROL
PCM1	NM_006197 // PCM1 // pericentriolar material 1 // 8p22-p21.3 // 5108 /// ENST000	2,66	LUMINAL A up vs CONTROL
WRB	NM_004627 // WRB // tryptophan rich basic protein // 21q22.3 // 7485 /// NM_0011	2,66	LUMINAL A up vs CONTROL
SCARNA4	NR_003005 // SCARNA4 // small Cajal body-specific RNA 4 // 1q22 // 677771 /// NR	2,66	LUMINAL A up vs CONTROL
MAT2A	NM_005911 // MAT2A // methionine adenosyltransferase II, alpha // 2p11.2 // 4144	2,66	LUMINAL A up vs CONTROL
ME2	NM_002396 // ME2 // malic enzyme 2, NAD(+)-dependent, mitochondrial // 6p25-p24|	2,66	LUMINAL A up vs CONTROL
GOLT1B	NM_016072 // GOLT1B // golgi transport 1 homolog B (S. cerevisiae) // 12p12.1 //	2,66	LUMINAL A up vs CONTROL
LASS2	NM_181746 // LASS2 // LAG1 homolog, ceramide synthase 2 // 1q21.3 // 29956 /// N	2,66	LUMINAL A up vs CONTROL
NUDT21	NM_007006 // NUDT21 // nudix (nucleoside diphosphate linked moiety X)-type motif	2,65	LUMINAL A up vs CONTROL
ABHD13	NM_032859 // ABHD13 // abhydrolase domain containing 13 // 13q33.3 // 84945 ///	2,65	LUMINAL A up vs CONTROL
HSPA4	NM_002154 // HSPA4 // heat shock 70kDa protein 4 // 5q31.1-q31.2 // 3308 /// ENS	2,65	LUMINAL A up vs CONTROL
DDX1	NM_004939 // DDX1 // DEAD (Asp-Glu-Ala-Asp) box polypeptide 1 // 2p24 // 1653 //	2,65	LUMINAL A up vs CONTROL
LOC96610	NR_027293 // LOC96610 // BMS1 homolog, ribosome assembly protein (yeast) pseudog	2,65	LUMINAL A up vs CONTROL
SDAD1	NM_018115 // SDAD1 // SDA1 domain containing 1 // 4q21.1 // 55153 /// ENST000003	2,65	LUMINAL A up vs CONTROL
ZNF480	AK303271 // ZNF480 // zinc finger protein 480 // 19q13.41 // 147657 /// BC065503	2,65	LUMINAL A up vs CONTROL

MRPS10	NM_018141 // MRPS10 // mitochondrial ribosomal protein S10 // 6p21.1 // 55173 //	2,65	LUMINAL A up vs CONTROL
DHX9	NR_033302 // DHX9 // DEAH (Asp-Glu-Ala-His) box polypeptide 9 // 1q25 // 1660 //	2,65	LUMINAL A up vs CONTROL
LRPPRC	NM_133259 // LRPPRC // leucine-rich PPR-motif containing // 2p21 // 10128 /// EN	2,65	LUMINAL A up vs CONTROL
KLHL9	NM_018847 // KLHL9 // kelch-like 9 (Drosophila) // 9p22 // 55958 /// ENST0000035	2,64	LUMINAL A up vs CONTROL
ATAD2	NM_014109 // ATAD2 // ATPase family, AAA domain containing 2 // 8q24.13 // 29028	2,64	LUMINAL A up vs CONTROL
SAMD12	NM_207506 // SAMD12 // sterile alpha motif domain containing 12 // 8q24.12 // 40	2,64	LUMINAL A up vs CONTROL
FAR1	NM_032228 // FAR1 // fatty acyl CoA reductase 1 // 11p15.2 // 84188 /// ENST0000	2,64	LUMINAL A up vs CONTROL
EIF3M	NM_006360 // EIF3M // eukaryotic translation initiation factor 3, subunit M // 1	2,64	LUMINAL A up vs CONTROL
RBM47	NM_001098634 // RBM47 // RNA binding motif protein 47 // 4p13-p12 // 54502 /// N	2,64	LUMINAL A up vs CONTROL
PAK1	NM_001128620 // PAK1 // p21 protein (Cdc42/Rac)-activated kinase 1 // 11q13-q14	2,63	LUMINAL A up vs CONTROL
KRT19	NM_002276 // KRT19 // keratin 19 // 17q21.2 // 3880 /// ENST00000361566 // KRT19	2,63	LUMINAL A up vs CONTROL
CASP3	NM_004346 // CASP3 // caspase 3, apoptosis-related cysteine peptidase // 4q34 //	2,63	LUMINAL A up vs CONTROL
ZBTB33	NM_006777 // ZBTB33 // zinc finger and BTB domain containing 33 // Xq23 // 10009	2,63	LUMINAL A up vs CONTROL
XBP1	NM_005080 // XBP1 // X-box binding protein 1 // 22q12.1|22q12 // 7494 /// NM_001	2,63	LUMINAL A up vs CONTROL
MDM4	NM_002393 // MDM4 // Mdm4 p53 binding protein homolog (mouse) // 1q32 // 4194 //	2,63	LUMINAL A up vs CONTROL
KCTD6	NM_153331 // KCTD6 // potassium channel tetramerisation domain containing 6 // 3	2,62	LUMINAL A up vs CONTROL
HPRT1	NM_000194 // HPRT1 // hypoxanthine phosphoribosyltransferase 1 // Xq26.1 // 3251	2,62	LUMINAL A up vs CONTROL
SLC16A1	NM_001166496 // SLC16A1 // solute carrier family 16, member 1 (monocarboxylic ac	2,62	LUMINAL A up vs CONTROL
GPR65	NM_003608 // GPR65 // G protein-coupled receptor 65 // 14q31-q32.1 // 8477 /// E	2,62	LUMINAL A up vs CONTROL
SMC4	NM_005496 // SMC4 // structural maintenance of chromosomes 4 // 3q26.1 // 10051	2,62	LUMINAL A up vs CONTROL
PRPF4B	NM_003913 // PRPF4B // PRP4 pre-mRNA processing factor 4 homolog B (yeast) // 6p	2,62	LUMINAL A up vs CONTROL
UQCRC2	NM_003366 // UQCRC2 // ubiquinol-cytochrome c reductase core protein II // 16p12	2,62	LUMINAL A up vs CONTROL
ATP6V1D	NM_015994 // ATP6V1D // ATPase, H+ transporting, lysosomal 34kDa, V1 subunit D /	2,62	LUMINAL A up vs CONTROL
SCGB2A1	NM_002407 // SCGB2A1 // secretoglobin, family 2A, member 1 // 11q13 // 4246 ///	2,62	LUMINAL A up vs CONTROL
TFEC	NM_012252 // TFEC // transcription factor EC // 7q31.2 // 22797 /// NM_001018058	2,62	LUMINAL A up vs CONTROL
LARS	NM_020117 // LARS // leucyl-tRNA synthetase // 5q32 // 51520 /// ENST00000394434	2,62	LUMINAL A up vs CONTROL
SFRS3	NM_003017 // SFRS3 // splicing factor, arginine/serine-rich 3 // 6p21 // 6428 //	2,61	LUMINAL A up vs CONTROL
ITGAV	NM_002210 // ITGAV // integrin, alpha V (vitronectin receptor, alpha polypeptide	2,61	LUMINAL A up vs CONTROL
PHF20L1	NM_016018 // PHF20L1 // PHD finger protein 20-like 1 // 8q24.22 // 51105 /// NM_	2,61	LUMINAL A up vs CONTROL
PLA2G7	NM_001168357 // PLA2G7 // phospholipase A2, group VII (platelet-activating facto	2,61	LUMINAL A up vs CONTROL
SNX13	NM_015132 // SNX13 // sorting nexin 13 // 7p21.1 // 23161 /// ENST00000428135 //	2,61	LUMINAL A up vs CONTROL
HSD17B4	NM_000414 // HSD17B4 // hydroxysteroid (17-beta) dehydrogenase 4 // 5q21 // 3295	2,61	LUMINAL A up vs CONTROL
SETX	NM_015046 // SETX // senataxin // 9q34.13 // 23064 /// ENST00000224140 // SETX /	2,61	LUMINAL A up vs CONTROL

GSR	NM_000637 // GSR // glutathione reductase // 8p21.1 // 2936 /// ENST00000221130	2,61	LUMINAL A up vs CONTROL
RCN1	NM_002901 // RCN1 // reticulocalbin 1, EF-hand calcium binding domain // 11p13 /	2,61	LUMINAL A up vs CONTROL
IPO7	NM_006391 // IPO7 // importin 7 // 11p15.4 // 10527 /// NR_002962 // SNORA23 //	2,61	LUMINAL A up vs CONTROL
PRKDC	NM_006904 // PRKDC // protein kinase, DNA-activated, catalytic polypeptide // 8q	2,60	LUMINAL A up vs CONTROL
LARP4	NM_052879 // LARP4 // La ribonucleoprotein domain family, member 4 // 12q13.12 /	2,60	LUMINAL A up vs CONTROL
FAM49B	BC017297 // FAM49B // family with sequence similarity 49, member B // 8q24.21 //	2,60	LUMINAL A up vs CONTROL
PBX1	NM_002585 // PBX1 // pre-B-cell leukemia homeobox 1 // 1q23 // 5087 /// ENST0000	2,60	LUMINAL A up vs CONTROL
ANK3	NM_020987 // ANK3 // ankyrin 3, node of Ranvier (ankyrin G) // 10q21 // 288 ///	2,60	LUMINAL A up vs CONTROL
VPS13C	NM_020821 // VPS13C // vacuolar protein sorting 13 homolog C (S. cerevisiae) //	2,59	LUMINAL A up vs CONTROL
STRBP	NM_001171137 // STRBP // spermatid perinuclear RNA binding protein // 9q33.3 //	2,59	LUMINAL A up vs CONTROL
C5orf42	BC144069 // C5orf42 // chromosome 5 open reading frame 42 // 5p13.2 // 65250 ///	2,59	LUMINAL A up vs CONTROL
SRD5A3	NM_024592 // SRD5A3 // steroid 5 alpha-reductase 3 // 4q12 // 79644 /// ENST0000	2,59	LUMINAL A up vs CONTROL
GNAQ	NM_002072 // GNAQ // guanine nucleotide binding protein (G protein), q polypepti	2,59	LUMINAL A up vs CONTROL
SLC25A24	NM_213651 // SLC25A24 // solute carrier family 25 (mitochondrial carrier; phosph	2,59	LUMINAL A up vs CONTROL
NCK1	NM_006153 // NCK1 // NCK adaptor protein 1 // 3q21 // 4690 /// ENST00000288986 /	2,59	LUMINAL A up vs CONTROL
CD2	NM_001767 // CD2 // CD2 molecule // 1p13.1 // 914 /// ENST00000369478 // CD2 //	2,59	LUMINAL A up vs CONTROL
SERINC1	NM_020755 // SERINC1 // serine incorporator 1 // 6q22.31 // 57515 /// ENST000003	2,59	LUMINAL A up vs CONTROL
CDC42	NM_001039802 // CDC42 // cell division cycle 42 (GTP binding protein, 25kDa) //	2,59	LUMINAL A up vs CONTROL
ALG1L	NM_001015050 // ALG1L // asparagine-linked glycosylation 1-like // 3q21.2 // 200	2,58	LUMINAL A up vs CONTROL
CHMP2A	NM_014453 // CHMP2A // chromatin modifying protein 2A // 19q // 27243 /// NM_198	2,58	LUMINAL A up vs CONTROL
SMC2	NM_001042550 // SMC2 // structural maintenance of chromosomes 2 // 9q31.1 // 105	2,58	LUMINAL A up vs CONTROL
FARSB	NM_005687 // FARSB // phenylalanyl-tRNA synthetase, beta subunit // 2q36.1 // 10	2,58	LUMINAL A up vs CONTROL
CLDN1	NM_021101 // CLDN1 // claudin 1 // 3q28-q29 // 9076 /// ENST00000295522 // CLDN1	2,58	LUMINAL A up vs CONTROL
ENY2	NM_020189 // ENY2 // enhancer of yellow 2 homolog (Drosophila) // 8q23.1 // 5694	2,58	LUMINAL A up vs CONTROL
LOC100288114	AK302988 // LOC100288114 // hypothetical protein LOC100288114 // 19q13.43 // 100	2,58	LUMINAL A up vs CONTROL
CBX3	NM_016587 // CBX3 // chromobox homolog 3 (HP1 gamma homolog, Drosophila) // 7p15	2,57	LUMINAL A up vs CONTROL
REEP5	NM_005669 // REEP5 // receptor accessory protein 5 // 5q22-q23 // 7905 /// ENST0	2,57	LUMINAL A up vs CONTROL
INPP4B	NM_003866 // INPP4B // inositol polyphosphate-4-phosphatase, type II, 105kDa //	2,57	LUMINAL A up vs CONTROL
ZNF148	NM_021964 // ZNF148 // zinc finger protein 148 // 3q21 // 7707 /// ENST000003606	2,57	LUMINAL A up vs CONTROL
ZNF138	NM_001160183 // ZNF138 // zinc finger protein 138 // 7q11.21-q11.23 // 7697 ///	2,57	LUMINAL A up vs CONTROL
ARPP19	NM_006628 // ARPP19 // cAMP-regulated phosphoprotein, 19kDa // 15q21.2 // 10776	2,57	LUMINAL A up vs CONTROL
TMEM14A	NM_014051 // TMEM14A // transmembrane protein 14A // 6p12.2 // 28978 /// ENST000	2,57	LUMINAL A up vs CONTROL
SLAMF7	NM_021181 // SLAMF7 // SLAM family member 7 // 1q23.1-q24.1 // 57823 /// ENST000	2,57	LUMINAL A up vs CONTROL

CRIPT	NM_014171 // CRIPT // cysteine-rich PDZ-binding protein // 2p21 // 9419 /// ENST	2,57	LUMINAL A up vs CONTROL
PSMB2	NM_002794 // PSMB2 // proteasome (prosome, macropain) subunit, beta type, 2 // 1	2,57	LUMINAL A up vs CONTROL
ASAH1	NM_177924 // ASAH1 // N-acylsphingosine amidohydrolase (acid ceramidase) 1 // 8p	2,57	LUMINAL A up vs CONTROL
TMEM60	NM_032936 // TMEM60 // transmembrane protein 60 // 7q11.23 // 85025 /// ENST0000	2,57	LUMINAL A up vs CONTROL
PEX11B	NM_003846 // PEX11B // peroxisomal biogenesis factor 11 beta // 1q21.1 // 8799 /	2,56	LUMINAL A up vs CONTROL
NONO	NM_001145408 // NONO // non-POU domain containing, octamer-binding // Xq13.1 //	2,56	LUMINAL A up vs CONTROL
C12orf4	NM_020374 // C12orf4 // chromosome 12 open reading frame 4 // 12p13.3 // 57102 /	2,56	LUMINAL A up vs CONTROL
CYP2R1	NM_024514 // CYP2R1 // cytochrome P450, family 2, subfamily R, polypeptide 1 //	2,56	LUMINAL A up vs CONTROL
MTX2	NR_027850 // MTX2 // metaxin 2 // 2q31.1 // 10651 /// NM_006554 // MTX2 // metax	2,56	LUMINAL A up vs CONTROL
UHMK1	NM_175866 // UHMK1 // U2AF homology motif (UHM) kinase 1 // 1q23.3 // 127933 ///	2,56	LUMINAL A up vs CONTROL
SPATA5	NM_145207 // SPATA5 // spermatogenesis associated 5 // 4q28.1 // 166378 /// ENST	2,56	LUMINAL A up vs CONTROL
ATP8B1	NM_005603 // ATP8B1 // ATPase, aminophospholipid transporter, class I, type 8B,	2,56	LUMINAL A up vs CONTROL
SH3PXD2B	NM_001017995 // SH3PXD2B // SH3 and PX domains 2B // 5q35.1 // 285590 /// ENST00	2,56	LUMINAL A up vs CONTROL
COG6	NR_026745 // COG6 // component of oligomeric golgi complex 6 // 13q14.11 // 5751	2,56	LUMINAL A up vs CONTROL
AP3B1	NM_003664 // AP3B1 // adaptor-related protein complex 3, beta 1 subunit // 5q14.	2,56	LUMINAL A up vs CONTROL
LPAR6	NM_005767 // LPAR6 // lysophosphatidic acid receptor 6 // 13q14 // 10161 /// NM_	2,56	LUMINAL A up vs CONTROL
GOLM1	NM_016548 // GOLM1 // golgi membrane protein 1 // 9q21.33 // 51280 /// NM_177937	2,55	LUMINAL A up vs CONTROL
GMPS	NM_003875 // GMPS // guanine monphosphate synthetase // 3q24 // 8833 /// ENST000	2,55	LUMINAL A up vs CONTROL
CHN1	NM_001822 // CHN1 // chimerin (chimaerin) 1 // 2q31.1 // 1123 /// NM_001025201 /	2,55	LUMINAL A up vs CONTROL
COG2	NM_007357 // COG2 // component of oligomeric golgi complex 2 // 1q42.2 // 22796	2,55	LUMINAL A up vs CONTROL
SLC25A36	NM_001104647 // SLC25A36 // solute carrier family 25, member 36 // 3q23 // 55186	2,55	LUMINAL A up vs CONTROL
THOC2	NM_001081550 // THOC2 // THO complex 2 // Xq25-q26.3 // 57187 /// ENST0000024583	2,55	LUMINAL A up vs CONTROL
GABPA	NM_002040 // GABPA // GA binding protein transcription factor, alpha subunit 60k	2,55	LUMINAL A up vs CONTROL
TTC8	NM_144596 // TTC8 // tetratricopeptide repeat domain 8 // 14q31.3 // 123016 ///	2,55	LUMINAL A up vs CONTROL
ANKRD36B	NM_025190 // ANKRD36B // ankyrin repeat domain 36B // 2q11.2 // 57730 /// ENST00	2,55	LUMINAL A up vs CONTROL
DBT	NM_001918 // DBT // dihydrolipoamide branched chain transacylase E2 // 1p31 // 1	2,55	LUMINAL A up vs CONTROL
TBK1	NM_013254 // TBK1 // TANK-binding kinase 1 // 12q14.1 // 29110 /// ENST000003317	2,55	LUMINAL A up vs CONTROL
CPD	NM_001304 // CPD // carboxypeptidase D // 17q11.2 // 1362 /// ENST00000225719 //	2,55	LUMINAL A up vs CONTROL
SUCNR1	NM_033050 // SUCNR1 // succinate receptor 1 // 3q24-q25.1 // 56670 /// ENST00000	2,55	LUMINAL A up vs CONTROL
MRPL50	NM_019051 // MRPL50 // mitochondrial ribosomal protein L50 // 9q31.1 // 54534 //	2,55	LUMINAL A up vs CONTROL
COPZ1	NM_016057 // COPZ1 // coatomer protein complex, subunit zeta 1 // 12q13.2-q13.3	2,55	LUMINAL A up vs CONTROL
SLAMF6	NM_052931 // SLAMF6 // SLAM family member 6 // 1q23.2 // 114836 /// AY358159 //	2,54	LUMINAL A up vs CONTROL
DLD	NM_000108 // DLD // dihydrolipoamide dehydrogenase // 7q31-q32 // 1738 /// ENST0	2,54	LUMINAL A up vs CONTROL

LCLAT1	NM_182551 // LCLAT1 // lysocardiolipin acyltransferase 1 // 2p23.1 // 253558 ///	2,54	LUMINAL A up vs CONTROL
CAP1	NM_006367 // CAP1 // CAP, adenylate cyclase-associated protein 1 (yeast) // 1p34	2,54	LUMINAL A up vs CONTROL
TUBA1C	NM_032704 // TUBA1C // tubulin, alpha 1c // 12q12-q14 // 84790 /// ENST000003010	2,54	LUMINAL A up vs CONTROL
TMEM14C	NM_001165258 // TMEM14C // transmembrane protein 14C // 6p24.2 // 51522 /// NM_0	2,54	LUMINAL A up vs CONTROL
SSR2	NM_003145 // SSR2 // signal sequence receptor, beta (translocon-associated prote	2,54	LUMINAL A up vs CONTROL
FZD6	NM_003506 // FZD6 // frizzled homolog 6 (Drosophila) // 8q22.3-q23.1 // 8323 ///	2,54	LUMINAL A up vs CONTROL
ZNF267	NM_003414 // ZNF267 // zinc finger protein 267 // 16p11.2 // 10308 /// ENST00000	2,54	LUMINAL A up vs CONTROL
C14orf156	NM_031210 // C14orf156 // chromosome 14 open reading frame 156 // 14q24.3 // 818	2,53	LUMINAL A up vs CONTROL
C1orf25	NM_030934 // C1orf25 // chromosome 1 open reading frame 25 // 1q25.2 // 81627 //	2,53	LUMINAL A up vs CONTROL
HNRNPR	NM_001102398 // HNRNPR // heterogeneous nuclear ribonucleoprotein R // 1p36.12 /	2,53	LUMINAL A up vs CONTROL
UGGT1	NR_027671 // UGGT1 // UDP-glucose glycoprotein glucosyltransferase 1 // 2q14.3 /	2,53	LUMINAL A up vs CONTROL
IL18	NM_001562 // IL18 // interleukin 18 (interferon-gamma-inducing factor) // 11q22.	2,53	LUMINAL A up vs CONTROL
PQLC3	NM_152391 // PQLC3 // PQ loop repeat containing 3 // 2p25.1 // 130814 /// ENST00	2,53	LUMINAL A up vs CONTROL
C22orf30	NM_173566 // C22orf30 // chromosome 22 open reading frame 30 // 22q12.2-q12.3 //	2,53	LUMINAL A up vs CONTROL
CD58	NR_026665 // CD58 // CD58 molecule // 1p13 // 965 /// NM_001779 // CD58 // CD58	2,53	LUMINAL A up vs CONTROL
RNF14	NM_004290 // RNF14 // ring finger protein 14 // 5q23.3-q31.1 // 9604 /// NM_1834	2,53	LUMINAL A up vs CONTROL
KLRK1	NM_007360 // KLRK1 // killer cell lectin-like receptor subfamily K, member 1 //	2,52	LUMINAL A up vs CONTROL
TRAPPC4	NM_016146 // TRAPPC4 // trafficking protein particle complex 4 // 11q23.3 // 513	2,52	LUMINAL A up vs CONTROL
FARS2	NM_006567 // FARS2 // phenylalanyl-tRNA synthetase 2, mitochondrial // 6p25.1 //	2,52	LUMINAL A up vs CONTROL
C8orf37	NM_177965 // C8orf37 // chromosome 8 open reading frame 37 // 8q22.1 // 157657 /	2,52	LUMINAL A up vs CONTROL
UTP23	NM_032334 // UTP23 // UTP23, small subunit (SSU) processome component, homolog (	2,52	LUMINAL A up vs CONTROL
TRAPPC6B	NM_001079537 // TRAPPC6B // trafficking protein particle complex 6B // 14q21.1 /	2,52	LUMINAL A up vs CONTROL
EI24	NM_004879 // EI24 // etoposide induced 2.4 mRNA // 11q24 // 9538 /// NM_00100727	2,52	LUMINAL A up vs CONTROL
IGFBP5	NM_000599 // IGFBP5 // insulin-like growth factor binding protein 5 // 2q33-q36	2,52	LUMINAL A up vs CONTROL
07/mar	NM_022826 // MARCH7 // membrane-associated ring finger (C3HC4) 7 // 2q24.2 // 64	2,52	LUMINAL A up vs CONTROL
USP1	NM_003368 // USP1 // ubiquitin specific peptidase 1 // 1p31.3 // 7398 /// NM_001	2,52	LUMINAL A up vs CONTROL
ATM	NM_000051 // ATM // ataxia telangiectasia mutated // 11q22-q23 // 472 /// NM_138	2,52	LUMINAL A up vs CONTROL
KIAA1715	NM_030650 // KIAA1715 // KIAA1715 // 2q31 // 80856 /// ENST00000272748 // KIAA17	2,52	LUMINAL A up vs CONTROL
C15orf44	AK296134 // C15orf44 // chromosome 15 open reading frame 44 // 15q22.31 // 81556	2,52	LUMINAL A up vs CONTROL
RAD50	NM_005732 // RAD50 // RAD50 homolog (S. cerevisiae) // 5q31 // 10111 /// ENST000	2,51	LUMINAL A up vs CONTROL
SLC44A5	NM_152697 // SLC44A5 // solute carrier family 44, member 5 // 1p31.1 // 204962 /	2,51	LUMINAL A up vs CONTROL
CA2	NM_000067 // CA2 // carbonic anhydrase II // 8q22 // 760 /// ENST00000285379 //	2,51	LUMINAL A up vs CONTROL
PRDX3	NM_006793 // PRDX3 // peroxiredoxin 3 // 10q25-q26 // 10935 /// NM_014098 // PRD	2,51	LUMINAL A up vs CONTROL

DDX18	NM_006773 // DDX18 // DEAD (Asp-Glu-Ala-Asp) box polypeptide 18 // 2q14.1 // 888	2,51	LUMINAL A up vs CONTROL
IPO11	NM_016338 // IPO11 // importin 11 // 5q12.1 // 51194 /// NM_001134779 // IPO11 /	2,51	LUMINAL A up vs CONTROL
ATG4C	NM_032852 // ATG4C // ATG4 autophagy related 4 homolog C (S. cerevisiae) // 1p31	2,51	LUMINAL A up vs CONTROL
STK17B	NM_004226 // STK17B // serine/threonine kinase 17b // 2q32.3 // 9262 /// ENST000	2,51	LUMINAL A up vs CONTROL
TMEM68	NM_152417 // TMEM68 // transmembrane protein 68 // 8q12.1 // 137695 /// ENST0000	2,51	LUMINAL A up vs CONTROL
HIST1H3D	NM_003530 // HIST1H3D // histone cluster 1, H3d // 6p21.3 // 8351 /// NM_021065	2,51	LUMINAL A up vs CONTROL
TM7SF3	NM_016551 // TM7SF3 // transmembrane 7 superfamily member 3 // 12q11-q12 // 5176	2,51	LUMINAL A up vs CONTROL
MCCC2	NM_022132 // MCCC2 // methylcrotonoyl-CoA carboxylase 2 (beta) // 5q12-q13 // 64	2,51	LUMINAL A up vs CONTROL
OR52N5	NM_001001922 // OR52N5 // olfactory receptor, family 52, subfamily N, member 5 /	2,50	LUMINAL A up vs CONTROL
MPV17	NM_002437 // MPV17 // MpV17 mitochondrial inner membrane protein // 2p23.3 // 43	2,50	LUMINAL A up vs CONTROL
ORMDL2	NM_014182 // ORMDL2 // ORM1-like 2 (S. cerevisiae) // 12q13.2 // 29095 /// ENST0	2,50	LUMINAL A up vs CONTROL
MBNL1	NM_021038 // MBNL1 // muscleblind-like (Drosophila) // 3q25 // 4154 /// NM_20729	2,50	LUMINAL A up vs CONTROL
GALNT3	NM_004482 // GALNT3 // UDP-N-acetyl-alpha-D-galactosamine:polypeptide N-acetylga	2,50	LUMINAL A up vs CONTROL
CENPF	NM_016343 // CENPF // centromere protein F, 350/400ka (mitosin) // 1q32-q41 // 1	2,50	LUMINAL A up vs CONTROL
RHOT1	NM_001033568 // RHOT1 // ras homolog gene family, member T1 // 17q11.2 // 55288	2,50	LUMINAL A up vs CONTROL
PRIM1	NM_000946 // PRIM1 // primase, DNA, polypeptide 1 (49kDa) // 12q13 // 5557 /// E	2,50	LUMINAL A up vs CONTROL
SPATA17	NM_138796 // SPATA17 // spermatogenesis associated 17 // 1q41 // 128153 /// ENST	2,49	LUMINAL A up vs CONTROL
ABI3BP	NM_015429 // ABI3BP // ABI family, member 3 (NESH) binding protein // 3q12 // 25	2,49	LUMINAL A up vs CONTROL
TGFBR1	NM_004612 // TGFBR1 // transforming growth factor, beta receptor 1 // 9q22 // 70	2,49	LUMINAL A up vs CONTROL
RNF139	NM_007218 // RNF139 // ring finger protein 139 // 8q24 // 11236 /// ENST00000303	2,49	LUMINAL A up vs CONTROL
ITPR2	NM_002223 // ITPR2 // inositol 1,4,5-triphosphate receptor, type 2 // 12p11 // 3	2,49	LUMINAL A up vs CONTROL
UBE2T	NM_014176 // UBE2T // ubiquitin-conjugating enzyme E2T (putative) // 1q32.1 // 2	2,49	LUMINAL A up vs CONTROL
POLK	NM_016218 // POLK // polymerase (DNA directed) kappa // 5q13 // 51426 /// ENST00	2,49	LUMINAL A up vs CONTROL
ACTG1	NM_001614 // ACTG1 // actin, gamma 1 // 17q25 // 71 /// ENST00000331925 // ACTG1	2,49	LUMINAL A up vs CONTROL
PIGB	NM_004855 // PIGB // phosphatidylinositol glycan anchor biosynthesis, class B //	2,48	LUMINAL A up vs CONTROL
CMBL	NM_138809 // CMBL // carboxymethylenebutenolidase homolog (Pseudomonas) // 5p15.	2,48	LUMINAL A up vs CONTROL
ATAD1	NM_032810 // ATAD1 // ATPase family, AAA domain containing 1 // 10q23.31 // 8489	2,48	LUMINAL A up vs CONTROL
IL1R1	NM_000877 // IL1R1 // interleukin 1 receptor, type I // 2q12 // 3554 /// ENST000	2,48	LUMINAL A up vs CONTROL
TMEM62	NM_024956 // TMEM62 // transmembrane protein 62 // 15q15.2 // 80021 /// ENST0000	2,48	LUMINAL A up vs CONTROL
KIF2A	NM_001098511 // KIF2A // kinesin heavy chain member 2A // 5q12-q13 // 3796 /// N	2,48	LUMINAL A up vs CONTROL
CHM	NM_000390 // CHM // choroideremia (Rab escort protein 1) // Xq21.2 // 1121 /// N	2,48	LUMINAL A up vs CONTROL
NAA20	NM_016100 // NAA20 // N(alpha)-acetyltransferase 20, NatB catalytic subunit // 2	2,48	LUMINAL A up vs CONTROL
AFTPH	NM_203437 // AFTPH // aftiphilin // 2p14 // 54812 /// NM_017657 // AFTPH // afti	2,48	LUMINAL A up vs CONTROL

C20orf112	NM_080616 // C20orf112 // chromosome 20 open reading frame 112 // 20q11.1-q11.23	2,48	LUMINAL A up vs CONTROL
ORC5L	NM_002553 // ORC5L // origin recognition complex, subunit 5-like (yeast) // 7q22	2,48	LUMINAL A up vs CONTROL
TIA1	NM_022173 // TIA1 // TIA1 cytotoxic granule-associated RNA binding protein // 2p	2,48	LUMINAL A up vs CONTROL
CSNK1A1	NM_001025105 // CSNK1A1 // casein kinase 1, alpha 1 // 5q32 // 1452 /// NM_00189	2,48	LUMINAL A up vs CONTROL
CCT5	NM_012073 // CCT5 // chaperonin containing TCP1, subunit 5 (epsilon) // 5p15.2 /	2,48	LUMINAL A up vs CONTROL
HIST1H2BG	NM_003518 // HIST1H2BG // histone cluster 1, H2bg // 6p21.3 // 8339	2,47	LUMINAL A up vs CONTROL
ZDHHC20	NM_153251 // ZDHHC20 // zinc finger, DHHC-type containing 20 // 13q12.11 // 2538	2,47	LUMINAL A up vs CONTROL
BZW2	NM_001159767 // BZW2 // basic leucine zipper and W2 domains 2 // 7p21.1 // 28969	2,47	LUMINAL A up vs CONTROL
RBM18	NR_027125 // RBM18 // RNA binding motif protein 18 // 9q33.2 // 92400 /// NR_027	2,47	LUMINAL A up vs CONTROL
ARHGAP29	NM_004815 // ARHGAP29 // Rho GTPase activating protein 29 // 1p22.1-p21.3 // 941	2,47	LUMINAL A up vs CONTROL
RAB10	NM_016131 // RAB10 // RAB10, member RAS oncogene family // 2p23.3 // 10890 /// E	2,47	LUMINAL A up vs CONTROL
HIST2H2BF	NM_001024599 // HIST2H2BF // histone cluster 2, H2bf // 1q21.2 // 440689 /// NM_	2,47	LUMINAL A up vs CONTROL
SLC35B1	NM_005827 // SLC35B1 // solute carrier family 35, member B1 // 17q21.33 // 10237	2,47	LUMINAL A up vs CONTROL
FIG4	NM_014845 // FIG4 // FIG4 homolog, SAC1 lipid phosphatase domain containing (S.	2,47	LUMINAL A up vs CONTROL
RICTOR	NM_152756 // RICTOR // RPTOR independent companion of MTOR, complex 2 // 5p13.1	2,47	LUMINAL A up vs CONTROL
ZC3HAV1L	BC020784 // ZC3HAV1L // zinc finger CCCH-type, antiviral 1-like // 7q34 // 92092	2,46	LUMINAL A up vs CONTROL
ZFAND5	NM_001102420 // ZFAND5 // zinc finger, AN1-type domain 5 // 9q13-q21 // 7763 ///	2,46	LUMINAL A up vs CONTROL
CGGBP1	NM_001008390 // CGGBP1 // CGG triplet repeat binding protein 1 // 3p12-p11.1 //	2,46	LUMINAL A up vs CONTROL
ALG6	NM_013339 // ALG6 // asparagine-linked glycosylation 6, alpha-1,3-glucosyltransf	2,46	LUMINAL A up vs CONTROL
SPPL3	NM_139015 // SPPL3 // signal peptide peptidase 3 // 12q24.31 // 121665 /// ENST0	2,46	LUMINAL A up vs CONTROL
LYRM5	NM_001001660 // LYRM5 // LYR motif containing 5 // 12p12.1 // 144363 /// ENST000	2,46	LUMINAL A up vs CONTROL
UBR5	NM_015902 // UBR5 // ubiquitin protein ligase E3 component n-recognin 5 // 8q22	2,46	LUMINAL A up vs CONTROL
CNIH	NM_005776 // CNIH // cornichon homolog (Drosophila) // 14q22.2 // 10175 /// ENST	2,46	LUMINAL A up vs CONTROL
CAPZA2	NM_006136 // CAPZA2 // capping protein (actin filament) muscle Z-line, alpha 2 /	2,46	LUMINAL A up vs CONTROL
CDH11	NM_001797 // CDH11 // cadherin 11, type 2, OB-cadherin (osteoblast) // 16q22.1 /	2,46	LUMINAL A up vs CONTROL
ARL6IP5	NM_006407 // ARL6IP5 // ADP-ribosylation-like factor 6 interacting protein 5 //	2,46	LUMINAL A up vs CONTROL
ZNF675	NM_138330 // ZNF675 // zinc finger protein 675 // 19p12 // 171392 /// ENST000003	2,45	LUMINAL A up vs CONTROL
UBA5	NM_024818 // UBA5 // ubiquitin-like modifier activating enzyme 5 // 3q22.1 // 79	2,45	LUMINAL A up vs CONTROL
UBD	NM_006398 // UBD // ubiquitin D // 6p21.3 // 10537 /// ENST00000377050 // UBD //	2,45	LUMINAL A up vs CONTROL
APOBEC3B	NM_004900 // APOBEC3B // apolipoprotein B mRNA editing enzyme, catalytic polypep	2,45	LUMINAL A up vs CONTROL
ELP2	NM_018255 // ELP2 // elongation protein 2 homolog (S. cerevisiae) // 18q12.2 //	2,45	LUMINAL A up vs CONTROL
CPSF2	NM_017437 // CPSF2 // cleavage and polyadenylation specific factor 2, 100kDa //	2,45	LUMINAL A up vs CONTROL
PHF6	NM_032458 // PHF6 // PHD finger protein 6 // Xq26.3 // 84295 /// NM_001015877 //	2,45	LUMINAL A up vs CONTROL

RIT1	NM_006912 // RIT1 // Ras-like without CAAX 1 // 1q22 // 6016 /// ENST00000368323	2,45	LUMINAL A up vs CONTROL
CALU	NM_001219 // CALU // calumenin // 7q32.1 // 813 /// NM_001130674 // CALU // calu	2,45	LUMINAL A up vs CONTROL
NBEA	NM_015678 // NBEA // neurobeachin // 13q13 // 26960 /// ENST00000400445 // NBEA	2,45	LUMINAL A up vs CONTROL
RPL8	NM_000973 // RPL8 // ribosomal protein L8 // 8q24.3 // 6132 /// NM_033301 // RPL	2,45	LUMINAL A up vs CONTROL
MED13	NM_005121 // MED13 // mediator complex subunit 13 // 17q22-q23 // 9969 /// ENST0	2,45	LUMINAL A up vs CONTROL
MED23	NM_004830 // MED23 // mediator complex subunit 23 // 6q22.33-q24.1 // 9439 /// N	2,45	LUMINAL A up vs CONTROL
PSME4	NM_014614 // PSME4 // proteasome (prosome, macropain) activator subunit 4 // 2p1	2,45	LUMINAL A up vs CONTROL
MAP4K5	NM_198794 // MAP4K5 // mitogen-activated protein kinase kinase kinase kinase 5 /	2,45	LUMINAL A up vs CONTROL
CCNC	NM_005190 // CCNC // cyclin C // 6q21 // 892 /// NM_001013399 // CCNC // cyclin	2,45	LUMINAL A up vs CONTROL
PRY	NM_004676 // PRY // PTPN13-like, Y-linked // Yq11.223 // 9081 /// NM_001002758 /	2,45	LUMINAL A up vs CONTROL
TFRC	NM_003234 // TFRC // transferrin receptor (p90, CD71) // 3q29 // 7037 /// NM_001	2,45	LUMINAL A up vs CONTROL
CCDC125	NM_176816 // CCDC125 // coiled-coil domain containing 125 // 5q13.2 // 202243 //	2,44	LUMINAL A up vs CONTROL
CAMSAP1L1	NM_203459 // CAMSAP1L1 // calmodulin regulated spectrin-associated protein 1-lik	2,44	LUMINAL A up vs CONTROL
ARFGEF2	NM_006420 // ARFGEF2 // ADP-ribosylation factor guanine nucleotide-exchange fact	2,44	LUMINAL A up vs CONTROL
MSL2	NM_018133 // MSL2 // male-specific lethal 2 homolog (Drosophila) // 3q22.3 // 55	2,44	LUMINAL A up vs CONTROL
C18orf32	BC093004 // C18orf32 // chromosome 18 open reading frame 32 // 18q21.1 // 497661	2,44	LUMINAL A up vs CONTROL
SLC38A6	NR_033344 // SLC38A6 // solute carrier family 38, member 6 // 14q23.1 // 145389	2,44	LUMINAL A up vs CONTROL
DICER1	NM_177438 // DICER1 // dicer 1, ribonuclease type III // 14q32.13 // 23405 /// N	2,44	LUMINAL A up vs CONTROL
CDKN1B	NM_004064 // CDKN1B // cyclin-dependent kinase inhibitor 1B (p27, Kip1) // 12p13	2,43	LUMINAL A up vs CONTROL
PDCD1LG2	NM_025239 // PDCD1LG2 // programmed cell death 1 ligand 2 // 9p24.2 // 80380 ///	2,43	LUMINAL A up vs CONTROL
GNPNAT1	NM_198066 // GNPNAT1 // glucosamine-phosphate N-acetyltransferase 1 // 14q22.1 /	2,43	LUMINAL A up vs CONTROL
UBE2N	NM_003348 // UBE2N // ubiquitin-conjugating enzyme E2N (UBC13 homolog, yeast) //	2,43	LUMINAL A up vs CONTROL
SLC10A7	NM_001029998 // SLC10A7 // solute carrier family 10 (sodium/bile acid cotranspor	2,43	LUMINAL A up vs CONTROL
TFF3	NM_003226 // TFF3 // trefoil factor 3 (intestinal) // 21q22.3 // 7033 /// ENST00	2,43	LUMINAL A up vs CONTROL
MON2	NM_015026 // MON2 // MON2 homolog (S. cerevisiae) // 12q14.1 // 23041 /// ENST00	2,43	LUMINAL A up vs CONTROL
SUPT4H1	NM_003168 // SUPT4H1 // suppressor of Ty 4 homolog 1 (S. cerevisiae) // 17q21-q2	2,43	LUMINAL A up vs CONTROL
CDH1	NM_004360 // CDH1 // cadherin 1, type 1, E-cadherin (epithelial) // 16q22.1 // 9	2,43	LUMINAL A up vs CONTROL
SBNO1	NM_001167856 // SBNO1 // strawberry notch homolog 1 (Drosophila) // 12q24.31 //	2,43	LUMINAL A up vs CONTROL
RAD51C	NM_058216 // RAD51C // RAD51 homolog C (S. cerevisiae) // 17q22-q23 // 5889 ///	2,43	LUMINAL A up vs CONTROL
EPB41L5	NM_020909 // EPB41L5 // erythrocyte membrane protein band 4.1 like 5 // 2q14.2 /	2,43	LUMINAL A up vs CONTROL
RIOK2	NM_018343 // RIOK2 // RIO kinase 2 (yeast) // 5q15 // 55781 /// NM_001159749 //	2,42	LUMINAL A up vs CONTROL
ARL5B	NM_178815 // ARL5B // ADP-ribosylation factor-like 5B // 10p12.31 // 221079 ///	2,42	LUMINAL A up vs CONTROL
ENC1	NM_003633 // ENC1 // ectodermal-neural cortex 1 (with BTB-like domain) // 5q12-q	2,42	LUMINAL A up vs CONTROL

FAM111A	NM_001142520 // FAM111A // family with sequence similarity 111, member A // 11q1	2,42	LUMINAL A up vs CONTROL
MRPS33	NM_016071 // MRPS33 // mitochondrial ribosomal protein S33 // 7q32-q34 // 51650	2,42	LUMINAL A up vs CONTROL
SEC24B	NM_006323 // SEC24B // SEC24 family, member B (S. cerevisiae) // 4q25 // 10427 /	2,42	LUMINAL A up vs CONTROL
SLC30A9	NM_006345 // SLC30A9 // solute carrier family 30 (zinc transporter), member 9 //	2,42	LUMINAL A up vs CONTROL
LYST	NM_000081 // LYST // lysosomal trafficking regulator // 1q42.1-q42.2 // 1130 ///	2,42	LUMINAL A up vs CONTROL
IFT80	NM_020800 // IFT80 // intraflagellar transport 80 homolog (Chlamydomonas) // 3q2	2,42	LUMINAL A up vs CONTROL
CNOT7	NM_013354 // CNOT7 // CCR4-NOT transcription complex, subunit 7 // 8p22-p21.3 //	2,42	LUMINAL A up vs CONTROL
SGPL1	NM_003901 // SGPL1 // sphingosine-1-phosphate lyase 1 // 10q21 // 8879 /// ENST0	2,42	LUMINAL A up vs CONTROL
CCDC144A	NM_014695 // CCDC144A // coiled-coil domain containing 144A // 17p11.2 // 9720 /	2,42	LUMINAL A up vs CONTROL
SFRS9	NM_003769 // SFRS9 // splicing factor, arginine/serine-rich 9 // 12q24.31 // 868	2,42	LUMINAL A up vs CONTROL
CXorf21	NM_025159 // CXorf21 // chromosome X open reading frame 21 // Xp21.2 // 80231 //	2,42	LUMINAL A up vs CONTROL
ARPC2	NM_152862 // ARPC2 // actin related protein 2/3 complex, subunit 2, 34kDa // 2q3	2,42	LUMINAL A up vs CONTROL
PSMB7	NM_002799 // PSMB7 // proteasome (prosome, macropain) subunit, beta type, 7 // 9	2,41	LUMINAL A up vs CONTROL
AIMP1	NM_004757 // AIMP1 // aminoacyl tRNA synthetase complex-interacting multifunctio	2,41	LUMINAL A up vs CONTROL
GBP4	NM_052941 // GBP4 // guanylate binding protein 4 // 1p22.2 // 115361 /// NM_0529	2,41	LUMINAL A up vs CONTROL
C15orf24	NM_020154 // C15orf24 // chromosome 15 open reading frame 24 // 15q14 // 56851 /	2,41	LUMINAL A up vs CONTROL
LMBR1	NM_022458 // LMBR1 // limb region 1 homolog (mouse) // 7q36 // 64327 /// ENST000	2,41	LUMINAL A up vs CONTROL
DAZAP2	NM_014764 // DAZAP2 // DAZ associated protein 2 // 12q12 // 9802 /// NM_00113626	2,41	LUMINAL A up vs CONTROL
GLT8D1	NM_001010983 // GLT8D1 // glycosyltransferase 8 domain containing 1 // 3p21.1 //	2,41	LUMINAL A up vs CONTROL
NCEH1	NM_001146276 // NCEH1 // neutral cholesterol ester hydrolase 1 // 3q26.31 // 575	2,41	LUMINAL A up vs CONTROL
PPCS	NM_024664 // PPCS // phosphopantothenoylcysteine synthetase // 1p34.2 // 79717 /	2,41	LUMINAL A up vs CONTROL
CISD2	NM_001008388 // CISD2 // CDGSH iron sulfur domain 2 // 4q24 // 493856 /// ENST00	2,41	LUMINAL A up vs CONTROL
NEK7	NM_133494 // NEK7 // NIMA (never in mitosis gene a)-related kinase 7 // 1q31.3 /	2,41	LUMINAL A up vs CONTROL
CACNG4	NM_014405 // CACNG4 // calcium channel, voltage-dependent, gamma subunit 4 // 17	2,41	LUMINAL A up vs CONTROL
TAS2R13	NM_023920 // TAS2R13 // taste receptor, type 2, member 13 // 12p13 // 50838 ///	2,41	LUMINAL A up vs CONTROL
COMMD10	NM_016144 // COMMD10 // COMM domain containing 10 // 5q23.1 // 51397 /// ENST000	2,41	LUMINAL A up vs CONTROL
SECISBP2	NM_024077 // SECISBP2 // SECIS binding protein 2 // 9q22.2 // 79048 /// ENST0000	2,41	LUMINAL A up vs CONTROL
TRPM7	NM_017672 // TRPM7 // transient receptor potential cation channel, subfamily M,	2,40	LUMINAL A up vs CONTROL
KIAA0101	NM_014736 // KIAA0101 // KIAA0101 // 15q22.31 // 9768 /// NM_001029989 // KIAA01	2,40	LUMINAL A up vs CONTROL
CHML	NM_001821 // CHML // choroideremia-like (Rab escort protein 2) // 1q42-qter // 1	2,40	LUMINAL A up vs CONTROL
WDR36	NM_139281 // WDR36 // WD repeat domain 36 // 5q22.1 // 134430 /// ENST0000032365	2,40	LUMINAL A up vs CONTROL
TMEM9	NM_016456 // TMEM9 // transmembrane protein 9 // --- // 252839 /// ENST000003673	2,40	LUMINAL A up vs CONTROL
SLC17A5	NM_012434 // SLC17A5 // solute carrier family 17 (anion/sugar transporter), memb	2,40	LUMINAL A up vs CONTROL

MRPL51	NM_016497 // MRPL51 // mitochondrial ribosomal protein L51 // 12p13.3-p13.1 // 5	2,40	LUMINAL A up vs CONTROL
OAS1	NM_016816 // OAS1 // 2',5'-oligoadenylate synthetase 1, 40/46kDa // 12q24.1 // 4	2,40	LUMINAL A up vs CONTROL
NPAT	NM_002519 // NPAT // nuclear protein, ataxia-telangiectasia locus // 11q22-q23 /	2,40	LUMINAL A up vs CONTROL
PWP1	NM_007062 // PWP1 // PWP1 homolog (S. cerevisiae) // 12q23.3 // 11137 /// ENST00	2,40	LUMINAL A up vs CONTROL
MED21	NM_004264 // MED21 // mediator complex subunit 21 // 12p11.23 // 9412 /// ENST00	2,39	LUMINAL A up vs CONTROL
DCTPP1	NM_024096 // DCTPP1 // dCTP pyrophosphatase 1 // 16p11.2 // 79077 /// ENST000003	2,39	LUMINAL A up vs CONTROL
MOSPD2	NM_152581 // MOSPD2 // motile sperm domain containing 2 // Xp22.2 // 158747 ///	2,39	LUMINAL A up vs CONTROL
LTA4H	NM_000895 // LTA4H // leukotriene A4 hydrolase // 12q22 // 4048 /// ENST00000228	2,39	LUMINAL A up vs CONTROL
TAF7	NM_005642 // TAF7 // TAF7 RNA polymerase II, TATA box binding protein (TBP)-asso	2,39	LUMINAL A up vs CONTROL
GTF2B	NM_001514 // GTF2B // general transcription factor IIB // 1p22-p21 // 2959 /// E	2,39	LUMINAL A up vs CONTROL
LANCL1	NM_006055 // LANCL1 // LanC lantibiotic synthetase component C-like 1 (bacterial	2,39	LUMINAL A up vs CONTROL
MMP16	AL136588 // MMP16 // matrix metallopeptidase 16 (membrane-inserted) // 8q21.3 //	2,39	LUMINAL A up vs CONTROL
ALG13	NM_001168385 // ALG13 // asparagine-linked glycosylation 13 homolog (S. cerevisi	2,39	LUMINAL A up vs CONTROL
ADAM12	NM_003474 // ADAM12 // ADAM metallopeptidase domain 12 // 10q26.3 // 8038 /// NM	2,39	LUMINAL A up vs CONTROL
CHURC1	NM_145165 // CHURC1 // churchill domain containing 1 // 14q23.3 // 91612 /// BC0	2,38	LUMINAL A up vs CONTROL
PSMD1	NM_002807 // PSMD1 // proteasome (prosome, macropain) 26S subunit, non-ATPase, 1	2,38	LUMINAL A up vs CONTROL
TMEM184C	NM_018241 // TMEM184C // transmembrane protein 184C // 4q31.23 // 55751 /// ENST	2,38	LUMINAL A up vs CONTROL
PHTF2	NM_001127358 // PHTF2 // putative homeodomain transcription factor 2 // 7q11.23-	2,38	LUMINAL A up vs CONTROL
FAM26E	NM_153711 // FAM26E // family with sequence similarity 26, member E // 6q22.1 //	2,38	LUMINAL A up vs CONTROL
SERPINA3	NM_001085 // SERPINA3 // serpin peptidase inhibitor, clade A (alpha-1 antiprotei	2,38	LUMINAL A up vs CONTROL
GHITM	NM_014394 // GHITM // growth hormone inducible transmembrane protein // 10q23.1	2,38	LUMINAL A up vs CONTROL
CSNK2A1	NM_177559 // CSNK2A1 // casein kinase 2, alpha 1 polypeptide // 20p13 // 1457 //	2,38	LUMINAL A up vs CONTROL
SSB	NM_003142 // SSB // Sjogren syndrome antigen B (autoantigen La) // 2q31.1 // 674	2,38	LUMINAL A up vs CONTROL
HRSP12	NM_005836 // HRSP12 // heat-responsive protein 12 // 8q22 // 10247 /// ENST00000	2,38	LUMINAL A up vs CONTROL
RERG	NM_032918 // RERG // RAS-like, estrogen-regulated, growth inhibitor // 12p12.3 /	2,38	LUMINAL A up vs CONTROL
ATP5C1	NM_001001973 // ATP5C1 // ATP synthase, H+ transporting, mitochondrial F1 comple	2,37	LUMINAL A up vs CONTROL
COL11A1	NM_001854 // COL11A1 // collagen, type XI, alpha 1 // 1p21 // 1301 /// NM_080629	2,37	LUMINAL A up vs CONTROL
MRPS21	NM_018997 // MRPS21 // mitochondrial ribosomal protein S21 // 1q21 // 54460 ///	2,37	LUMINAL A up vs CONTROL
PRKACB	NM_182948 // PRKACB // protein kinase, cAMP-dependent, catalytic, beta // 1p36.1	2,37	LUMINAL A up vs CONTROL
TM2D2	NM_031940 // TM2D2 // TM2 domain containing 2 // 8p11.22 // 83877 /// NM_0010243	2,37	LUMINAL A up vs CONTROL
DTWD2	NM_173666 // DTWD2 // DTW domain containing 2 // 5q23.1 // 285605 /// ENST000003	2,37	LUMINAL A up vs CONTROL
POLR2H	NM_006232 // POLR2H // polymerase (RNA) II (DNA directed) polypeptide H // 3q28	2,37	LUMINAL A up vs CONTROL
EAF2	NM_018456 // EAF2 // ELL associated factor 2 // 3q13.33 // 55840 /// ENST0000027	2,37	LUMINAL A up vs CONTROL

APOO	NM_024122 // APOO // apolipoprotein O // Xp22.11 // 79135 /// NR_026545 // APOO	2,37	LUMINAL A up vs CONTROL
KIFAP3	NM_014970 // KIFAP3 // kinesin-associated protein 3 // 1q24.2 // 22920 /// ENST0	2,36	LUMINAL A up vs CONTROL
SNRPB2	NM_003092 // SNRPB2 // small nuclear ribonucleoprotein polypeptide B'' // 20p12.	2,36	LUMINAL A up vs CONTROL
REPS2	NM_004726 // REPS2 // RALBP1 associated Eps domain containing 2 // Xp22.2-p22.13	2,36	LUMINAL A up vs CONTROL
AP2B1	NM_001030006 // AP2B1 // adaptor-related protein complex 2, beta 1 subunit // 17	2,36	LUMINAL A up vs CONTROL
TTC3	NM_003316 // TTC3 // tetratricopeptide repeat domain 3 // 21q22.2 // 7267 /// NM	2,36	LUMINAL A up vs CONTROL
ACSL3	NM_004457 // ACSL3 // acyl-CoA synthetase long-chain family member 3 // 2q34-q35	2,36	LUMINAL A up vs CONTROL
TXNIP	NM_006472 // TXNIP // thioredoxin interacting protein // 1q21.1 // 10628 /// ENS	2,36	LUMINAL A up vs CONTROL
MRPS17	NM_015969 // MRPS17 // mitochondrial ribosomal protein S17 // 7p11 // 51373 ///	2,35	LUMINAL A up vs CONTROL
RFC4	NM_002916 // RFC4 // replication factor C (activator 1) 4, 37kDa // 3q27 // 5984	2,35	LUMINAL A up vs CONTROL
SPATS2	NM_023071 // SPATS2 // spermatogenesis associated, serine-rich 2 // 12q13.12 //	2,35	LUMINAL A up vs CONTROL
DZIP3	NM_014648 // DZIP3 // DAZ interacting protein 3, zinc finger // 3q13.13 // 9666	2,35	LUMINAL A up vs CONTROL
C12orf35	NM_018169 // C12orf35 // chromosome 12 open reading frame 35 // 12p11.21 // 5519	2,35	LUMINAL A up vs CONTROL
PLEK	NM_002664 // PLEK // pleckstrin // 2p14-p13.3 // 5341 /// ENST00000234313 // PLE	2,35	LUMINAL A up vs CONTROL
SPARC	NM_003118 // SPARC // secreted protein, acidic, cysteine-rich (osteonectin) // 5	2,35	LUMINAL A up vs CONTROL
SDCBP	NM_005625 // SDCBP // syndecan binding protein (syntenin) // 8q12 // 6386 /// NM	2,35	LUMINAL A up vs CONTROL
TOMM20	NM_014765 // TOMM20 // translocase of outer mitochondrial membrane 20 homolog (y	2,35	LUMINAL A up vs CONTROL
PTRH2	NM_016077 // PTRH2 // peptidyl-tRNA hydrolase 2 // 17q23.1 // 51651 /// ENST0000	2,35	LUMINAL A up vs CONTROL
RIPK2	NM_003821 // RIPK2 // receptor-interacting serine-threonine kinase 2 // 8q21 //	2,35	LUMINAL A up vs CONTROL
C8orf59	NM_001099670 // C8orf59 // chromosome 8 open reading frame 59 // 8q21.2 // 40146	2,35	LUMINAL A up vs CONTROL
RPL30	NM_000989 // RPL30 // ribosomal protein L30 // 8q22 // 6156 /// NR_002581 // SNO	2,35	LUMINAL A up vs CONTROL
NEDD8	NM_006156 // NEDD8 // neural precursor cell expressed, developmentally down-regu	2,34	LUMINAL A up vs CONTROL
C4orf29	BC034253 // C4orf29 // chromosome 4 open reading frame 29 // 4q28.2 // 80167 ///	2,34	LUMINAL A up vs CONTROL
GPX8	NM_001008397 // GPX8 // glutathione peroxidase 8 (putative) // 5q11.2 // 493869	2,34	LUMINAL A up vs CONTROL
PIK3R4	NM_014602 // PIK3R4 // phosphoinositide-3-kinase, regulatory subunit 4 // 3q22.1	2,34	LUMINAL A up vs CONTROL
FCGR1A	NM_000566 // FCGR1A // Fc fragment of IgG, high affinity Ia, receptor (CD64) //	2,34	LUMINAL A up vs CONTROL
TOPBP1	NM_007027 // TOPBP1 // topoisomerase (DNA) II binding protein 1 // 3q22.1 // 110	2,34	LUMINAL A up vs CONTROL
SPATS2L	NM_001100422 // SPATS2L // spermatogenesis associated, serine-rich 2-like // 2q3	2,34	LUMINAL A up vs CONTROL
ZNF107	NM_016220 // ZNF107 // zinc finger protein 107 // 7q11.2 // 51427 /// NM_0010137	2,34	LUMINAL A up vs CONTROL
SMC6	NM_001142286 // SMC6 // structural maintenance of chromosomes 6 // 2p24.2 // 796	2,34	LUMINAL A up vs CONTROL
DHX29	NM_019030 // DHX29 // DEAH (Asp-Glu-Ala-His) box polypeptide 29 // 5q11.2 // 545	2,34	LUMINAL A up vs CONTROL
PAN3	NM_175854 // PAN3 // PAN3 poly(A) specific ribonuclease subunit homolog (S. cere	2,34	LUMINAL A up vs CONTROL
HSPB1	NM_001540 // HSPB1 // heat shock 27kDa protein 1 // 7q11.23 // 3315 /// ENST0000	2,33	LUMINAL A up vs CONTROL

SNAPC3	NM_001039697 // SNAPC3 // small nuclear RNA activating complex, polypeptide 3, 5	2,33	LUMINAL A up vs CONTROL
FBXO3	NM_033406 // FBXO3 // F-box protein 3 // 11p13 // 26273 /// NM_012175 // FBXO3 /	2,33	LUMINAL A up vs CONTROL
CP110	NM_014711 // CP110 // CP110 protein // 16p12.3 // 9738 /// ENST00000396212 // CP	2,33	LUMINAL A up vs CONTROL
PDS5A	NM_001100399 // PDS5A // PDS5, regulator of cohesion maintenance, homolog A (S.	2,33	LUMINAL A up vs CONTROL
IL13RA1	NM_001560 // IL13RA1 // interleukin 13 receptor, alpha 1 // Xq24 // 3597 /// ENS	2,33	LUMINAL A up vs CONTROL
CFH	NM_000186 // CFH // complement factor H // 1q32 // 3075 /// NM_001014975 // CFH	2,33	LUMINAL A up vs CONTROL
DHX32	NM_018180 // DHX32 // DEAH (Asp-Glu-Ala-His) box polypeptide 32 // 10q26.2 // 55	2,33	LUMINAL A up vs CONTROL
ITGA2	NM_002203 // ITGA2 // integrin, alpha 2 (CD49B, alpha 2 subunit of VLA-2 recepto	2,33	LUMINAL A up vs CONTROL
ZFYVE16	NM_001105251 // ZFYVE16 // zinc finger, FYVE domain containing 16 // 5q14 // 976	2,33	LUMINAL A up vs CONTROL
RALGAPA1	NM_014990 // RALGAPA1 // Ral GTPase activating protein, alpha subunit 1 (catalyt	2,33	LUMINAL A up vs CONTROL
RRM1	NM_001033 // RRM1 // ribonucleotide reductase M1 // 11p15.5 // 6240 /// ENST0000	2,33	LUMINAL A up vs CONTROL
ZNF721	NM_133474 // ZNF721 // zinc finger protein 721 // 4p16.3 // 170960 /// NM_001137	2,33	LUMINAL A up vs CONTROL
GLO1	NM_006708 // GLO1 // glyoxalase I // 6p21.3-p21.1 // 2739 /// ENST00000373365 //	2,33	LUMINAL A up vs CONTROL
TNFSF10	NM_003810 // TNFSF10 // tumor necrosis factor (ligand) superfamily, member 10 //	2,33	LUMINAL A up vs CONTROL
MKRN1	NM_013446 // MKRN1 // makorin ring finger protein 1 // 7q34 // 23608 /// NM_0011	2,33	LUMINAL A up vs CONTROL
SEC23A	NM_006364 // SEC23A // Sec23 homolog A (S. cerevisiae) // 14q21.1 // 10484 /// E	2,33	LUMINAL A up vs CONTROL
CD37	NM_001774 // CD37 // CD37 molecule // 19q13.3 // 951 /// NM_001040031 // CD37 //	2,33	LUMINAL A up vs CONTROL
C14orf101	NM_017799 // C14orf101 // chromosome 14 open reading frame 101 // 14q22.3 // 549	2,33	LUMINAL A up vs CONTROL
CCNT2	NM_058241 // CCNT2 // cyclin T2 // 2q21.3 // 905 /// NM_001241 // CCNT2 // cycli	2,32	LUMINAL A up vs CONTROL
MINPP1	NM_004897 // MINPP1 // multiple inositol-polyphosphate phosphatase 1 // 10q23 //	2,32	LUMINAL A up vs CONTROL
SEPSECS	NM_016955 // SEPSECS // Sep (O-phosphoserine) tRNA:Sec (selenocysteine) tRNA syn	2,32	LUMINAL A up vs CONTROL
TIMP1	NM_003254 // TIMP1 // TIMP metallopeptidase inhibitor 1 // Xp11.3-p11.23 // 7076	2,32	LUMINAL A up vs CONTROL
UBXN8	NM_005671 // UBXN8 // UBX domain protein 8 // 8p12-p11.2 // 7993 /// ENST0000026	2,32	LUMINAL A up vs CONTROL
RANBP9	NM_005493 // RANBP9 // RAN binding protein 9 // 6p23 // 10048 /// ENST0000001161	2,32	LUMINAL A up vs CONTROL
TMX1	NM_030755 // TMX1 // thioredoxin-related transmembrane protein 1 // 14q22.1 // 8	2,32	LUMINAL A up vs CONTROL
DDX58	NM_014314 // DDX58 // DEAD (Asp-Glu-Ala-Asp) box polypeptide 58 // 9p12 // 23586	2,32	LUMINAL A up vs CONTROL
P2RY13	NM_176894 // P2RY13 // purinergic receptor P2Y, G-protein coupled, 13 // 3q24 //	2,32	LUMINAL A up vs CONTROL
EDIL3	NM_005711 // EDIL3 // EGF-like repeats and discoidin I-like domains 3 // 5q14 //	2,31	LUMINAL A up vs CONTROL
PIK3CA	NM_006218 // PIK3CA // phosphoinositide-3-kinase, catalytic, alpha polypeptide /	2,31	LUMINAL A up vs CONTROL
CPEB2	NM_182485 // CPEB2 // cytoplasmic polyadenylation element binding protein 2 // 4	2,31	LUMINAL A up vs CONTROL
CSDE1	NM_001130523 // CSDE1 // cold shock domain containing E1, RNA-binding // 1p22 //	2,31	LUMINAL A up vs CONTROL
PRSS8	NM_002773 // PRSS8 // protease, serine, 8 // 16p11.2 // 5652 /// ENST00000317508	2,31	LUMINAL A up vs CONTROL
APH1A	NM_016022 // APH1A // anterior pharynx defective 1 homolog A (C. elegans) // 1p3	2,31	LUMINAL A up vs CONTROL

DCN	NM_001920 // DCN // decorin // 12q21.33 // 1634 /// NM_133503 // DCN // decorin	2,31	LUMINAL A up vs CONTROL
SAR1A	NM_001142648 // SAR1A // SAR1 homolog A (S. cerevisiae) // 10q22.1 // 56681 ///	2,31	LUMINAL A up vs CONTROL
SFRS1	NM_001078166 // SFRS1 // splicing factor, arginine/serine-rich 1 // 17q21.3-q22	2,31	LUMINAL A up vs CONTROL
SLC46A3	NM_181785 // SLC46A3 // solute carrier family 46, member 3 // 13q12.3 // 283537	2,31	LUMINAL A up vs CONTROL
MTCH2	NM_014342 // MTCH2 // mitochondrial carrier homolog 2 (C. elegans) // 11p11.2 //	2,31	LUMINAL A up vs CONTROL
CYP1B1	NM_000104 // CYP1B1 // cytochrome P450, family 1, subfamily B, polypeptide 1 //	2,31	LUMINAL A up vs CONTROL
SLC22A15	NM_018420 // SLC22A15 // solute carrier family 22, member 15 // 1p13.1 // 55356	2,31	LUMINAL A up vs CONTROL
OXNAD1	NM_138381 // OXNAD1 // oxidoreductase NAD-binding domain containing 1 // 3p25-p2	2,30	LUMINAL A up vs CONTROL
SBDS	NM_016038 // SBDS // Shwachman-Bodian-Diamond syndrome // 7q11.21 // 51119 /// N	2,30	LUMINAL A up vs CONTROL
UBE3A	NM_130839 // UBE3A // ubiquitin protein ligase E3A // 15q11-q13 // 7337 /// NM_0	2,30	LUMINAL A up vs CONTROL
THUMPD3	NM_015453 // THUMPD3 // THUMP domain containing 3 // 3p25.3 // 25917 /// NM_0011	2,30	LUMINAL A up vs CONTROL
BIRC2	NM_001166 // BIRC2 // baculoviral IAP repeat-containing 2 // 11q22 // 329 /// EN	2,30	LUMINAL A up vs CONTROL
REL	NM_002908 // REL // v-rel reticuloendotheliosis viral oncogene homolog (avian) /	2,30	LUMINAL A up vs CONTROL
GUF1	NM_021927 // GUF1 // GUF1 GTPase homolog (S. cerevisiae) // 4p12 // 60558 /// EN	2,30	LUMINAL A up vs CONTROL
SKP1	NM_006930 // SKP1 // S-phase kinase-associated protein 1 // 5q31 // 6500 /// NM_	2,30	LUMINAL A up vs CONTROL
CBR4	NM_032783 // CBR4 // carbonyl reductase 4 // 4q32.3 // 84869 /// ENST00000306193	2,30	LUMINAL A up vs CONTROL
PRPS1	NM_002764 // PRPS1 // phosphoribosyl pyrophosphate synthetase 1 // Xq21.32-q24 /	2,30	LUMINAL A up vs CONTROL
BRP44	NR_026550 // BRP44 // brain protein 44 // 1q24 // 25874 /// NM_001143674 // BRP4	2,30	LUMINAL A up vs CONTROL
GALNT5	NM_014568 // GALNT5 // UDP-N-acetyl-alpha-D-galactosamine:polypeptide N-acetylga	2,30	LUMINAL A up vs CONTROL
PRRX1	NM_006902 // PRRX1 // paired related homeobox 1 // 1q24 // 5396 /// NM_022716 //	2,30	LUMINAL A up vs CONTROL
NANS	NM_018946 // NANS // N-acetylneuraminic acid synthase // 9p24.1-p23 // 54187 ///	2,29	LUMINAL A up vs CONTROL
NRAS	NM_002524 // NRAS // neuroblastoma RAS viral (v-ras) oncogene homolog // 1p13.2	2,29	LUMINAL A up vs CONTROL
YWHAZ	NM_145690 // YWHAZ // tyrosine 3-monooxygenase/tryptophan 5-monooxygenase activa	2,29	LUMINAL A up vs CONTROL
CNOT1	NM_016284 // CNOT1 // CCR4-NOT transcription complex, subunit 1 // 16q21 // 2301	2,29	LUMINAL A up vs CONTROL
BCL2A1	NM_001114735 // BCL2A1 // BCL2-related protein A1 // 15q24.3 // 597 /// NM_00404	2,29	LUMINAL A up vs CONTROL
SEL1L	NM_005065 // SEL1L // sel-1 suppressor of lin-12-like (C. elegans) // 14q24.3-q3	2,29	LUMINAL A up vs CONTROL
TRAF5	NM_145759 // TRAF5 // TNF receptor-associated factor 5 // 1q32 // 7188 /// NM_00	2,29	LUMINAL A up vs CONTROL
SLC1A1	NM_004170 // SLC1A1 // solute carrier family 1 (neuronal/epithelial high affinit	2,29	LUMINAL A up vs CONTROL
IFI6	NM_002038 // IFI6 // interferon, alpha-inducible protein 6 // 1p35 // 2537 /// N	2,29	LUMINAL A up vs CONTROL
TNPO1	NM_002270 // TNPO1 // transportin 1 // 5q13.2 // 3842 /// NM_153188 // TNPO1 //	2,29	LUMINAL A up vs CONTROL
BTRC	NM_033637 // BTRC // beta-transducin repeat containing // 10q24.32 // 8945 /// N	2,29	LUMINAL A up vs CONTROL
NDUFB9	NM_005005 // NDUFB9 // NADH dehydrogenase (ubiquinone) 1 beta subcomplex, 9, 22k	2,29	LUMINAL A up vs CONTROL
MKLN1	NM_001145354 // MKLN1 // muskelin 1, intracellular mediator containing kelch mot	2,29	LUMINAL A up vs CONTROL

ASCC3	NM_006828 // ASCC3 // activating signal cointegrator 1 complex subunit 3 // 6q16	2,29	LUMINAL A up vs CONTROL
SRI	NM_003130 // SRI // sorcin // 7q21.1 // 6717 /// NM_198901 // SRI // sorcin // 7	2,29	LUMINAL A up vs CONTROL
PTPN22	NM_015967 // PTPN22 // protein tyrosine phosphatase, non-receptor type 22 (lymph	2,28	LUMINAL A up vs CONTROL
BMPR2	NM_001204 // BMPR2 // bone morphogenetic protein receptor, type II (serine/threo	2,28	LUMINAL A up vs CONTROL
CRNKL1	NM_016652 // CRNKL1 // crooked neck pre-mRNA splicing factor-like 1 (Drosophila)	2,28	LUMINAL A up vs CONTROL
CCNG2	NM_004354 // CCNG2 // cyclin G2 // 4q21.1 // 901 /// ENST00000316355 // CCNG2 //	2,28	LUMINAL A up vs CONTROL
SLC7A7	NM_003982 // SLC7A7 // solute carrier family 7 (cationic amino acid transporter,	2,28	LUMINAL A up vs CONTROL
CD180	NM_005582 // CD180 // CD180 molecule // 5q12 // 4064 /// ENST00000256447 // CD18	2,28	LUMINAL A up vs CONTROL
DSTN	NM_001011546 // DSTN // destrin (actin depolymerizing factor) // 20p12.1 // 1103	2,28	LUMINAL A up vs CONTROL
NUP153	NM_005124 // NUP153 // nucleoporin 153kDa // 6p22.3 // 9972 /// ENST00000262077	2,27	LUMINAL A up vs CONTROL
BRWD1	NM_033656 // BRWD1 // bromodomain and WD repeat domain containing 1 // 21q22.2 /	2,27	LUMINAL A up vs CONTROL
ARL8B	NM_018184 // ARL8B // ADP-ribosylation factor-like 8B // 3p26.1 // 55207 /// ENS	2,27	LUMINAL A up vs CONTROL
DHX15	NM_001358 // DHX15 // DEAH (Asp-Glu-Ala-His) box polypeptide 15 // 4p15.3 // 166	2,27	LUMINAL A up vs CONTROL
HINT1	NR_024610 // HINT1 // histidine triad nucleotide binding protein 1 // 5q31.2 //	2,27	LUMINAL A up vs CONTROL
UBXN4	NM_014607 // UBXN4 // UBX domain protein 4 // 2q21.3 // 23190 /// ENST0000027263	2,27	LUMINAL A up vs CONTROL
DCTD	NM_001012732 // DCTD // dCMP deaminase // 4q35.1 // 1635 /// NM_001921 // DCTD /	2,27	LUMINAL A up vs CONTROL
RIF1	NM_018151 // RIF1 // RAP1 interacting factor homolog (yeast) // 2q23.3 // 55183	2,27	LUMINAL A up vs CONTROL
MR1	NM_001531 // MR1 // major histocompatibility complex, class I-related // 1q25.3	2,27	LUMINAL A up vs CONTROL
FBXO28	NM_015176 // FBXO28 // F-box protein 28 // 1q42.11 // 23219 /// NM_001136115 //	2,27	LUMINAL A up vs CONTROL
UFC1	NM_016406 // UFC1 // ubiquitin-fold modifier conjugating enzyme 1 // 1q23.3 // 5	2,27	LUMINAL A up vs CONTROL
SENP1	NM_014554 // SENP1 // SUMO1/sentrin specific peptidase 1 // 12q13.1 // 29843 ///	2,27	LUMINAL A up vs CONTROL
TBC1D23	NM_018309 // TBC1D23 // TBC1 domain family, member 23 // 3q12.1-q12.2 // 55773 /	2,27	LUMINAL A up vs CONTROL
NCKAP1	NM_013436 // NCKAP1 // NCK-associated protein 1 // 2q32 // 10787 /// NM_205842 /	2,27	LUMINAL A up vs CONTROL
VPS41	NM_014396 // VPS41 // vacuolar protein sorting 41 homolog (S. cerevisiae) // 7p1	2,27	LUMINAL A up vs CONTROL
SYTL2	NM_206927 // SYTL2 // synaptotagmin-like 2 // 11q14 // 54843 /// NM_032943 // SY	2,27	LUMINAL A up vs CONTROL
CADPS2	NM_017954 // CADPS2 // Ca++-dependent secretion activator 2 // 7q31.3 // 93664 /	2,27	LUMINAL A up vs CONTROL
TLR5	NM_003268 // TLR5 // toll-like receptor 5 // 1q41-q42 // 7100 /// ENST0000036688	2,27	LUMINAL A up vs CONTROL
TDG	NM_003211 // TDG // thymine-DNA glycosylase // 12q24.1 // 6996 /// ENST000003928	2,27	LUMINAL A up vs CONTROL
PLBD1	NM_024829 // PLBD1 // phospholipase B domain containing 1 // 12p13.1 // 79887 //	2,27	LUMINAL A up vs CONTROL
C10orf81	NM_024889 // C10orf81 // chromosome 10 open reading frame 81 // 10q25.3 // 79949	2,27	LUMINAL A up vs CONTROL
SAR1B	NM_001033503 // SAR1B // SAR1 homolog B (S. cerevisiae) // 5q31.1 // 51128 /// N	2,26	LUMINAL A up vs CONTROL
DCTN4	NM_001135643 // DCTN4 // dynactin 4 (p62) // 5q31-q32 // 51164 /// NM_016221 //	2,26	LUMINAL A up vs CONTROL
IRAK4	NM_001114182 // IRAK4 // interleukin-1 receptor-associated kinase 4 // 12q12 //	2,26	LUMINAL A up vs CONTROL

VDAC3	NM_001135694 // VDAC3 // voltage-dependent anion channel 3 // 8p11.2 // 7419 ///	2,26	LUMINAL A up vs CONTROL
DMXL1	NM_005509 // DMXL1 // Dmx-like 1 // 5q22 // 1657 /// ENST00000311085 // DMXL1 //	2,26	LUMINAL A up vs CONTROL
TMEM98	NM_015544 // TMEM98 // transmembrane protein 98 // 17q11.2 // 26022 /// NM_00103	2,26	LUMINAL A up vs CONTROL
HLA-B	NM_005514 // HLA-B // major histocompatibility complex, class I, B // 6p21.3 //	2,26	LUMINAL A up vs CONTROL
STK17A	NM_004760 // STK17A // serine/threonine kinase 17a // 7p12-p14 // 9263 /// ENST0	2,26	LUMINAL A up vs CONTROL
ATP5G1	NM_005175 // ATP5G1 // ATP synthase, H+ transporting, mitochondrial F0 complex,	2,26	LUMINAL A up vs CONTROL
ATP6V0B	NM_004047 // ATP6V0B // ATPase, H+ transporting, lysosomal 21kDa, V0 subunit b /	2,26	LUMINAL A up vs CONTROL
SEC11C	NM_033280 // SEC11C // SEC11 homolog C (S. cerevisiae) // 18q21.32 // 90701 ///	2,26	LUMINAL A up vs CONTROL
TTC39A	NM_001080494 // TTC39A // tetratricopeptide repeat domain 39A // 1p32.3 // 22996	2,26	LUMINAL A up vs CONTROL
SPTLC1	NM_006415 // SPTLC1 // serine palmitoyltransferase, long chain base subunit 1 //	2,26	LUMINAL A up vs CONTROL
TMED10	NM_006827 // TMED10 // transmembrane emp24-like trafficking protein 10 (yeast) /	2,26	LUMINAL A up vs CONTROL
MEAF6	NM_022756 // MEAF6 // MYST/Esa1-associated factor 6 // 1p35.3-p33 // 64769 /// B	2,26	LUMINAL A up vs CONTROL
MYB	NM_001130173 // MYB // v-myb myeloblastosis viral oncogene homolog (avian) // 6q	2,26	LUMINAL A up vs CONTROL
ARHGAP32	NM_001142685 // ARHGAP32 // Rho GTPase activating protein 32 // 11q24-q25 // 974	2,26	LUMINAL A up vs CONTROL
PSMD10	NM_002814 // PSMD10 // proteasome (prosome, macropain) 26S subunit, non-ATPase,	2,25	LUMINAL A up vs CONTROL
ZNF207	NM_001098507 // ZNF207 // zinc finger protein 207 // 17q11.2 // 7756 /// NM_0034	2,25	LUMINAL A up vs CONTROL
C7orf44	NM_018224 // C7orf44 // chromosome 7 open reading frame 44 // 7p13 // 55744 ///	2,25	LUMINAL A up vs CONTROL
KIAA0368	NM_001080398 // KIAA0368 // KIAA0368 // 9q31.3 // 23392 /// ENST00000338205 // K	2,25	LUMINAL A up vs CONTROL
FKBP4	NM_002014 // FKBP4 // FK506 binding protein 4, 59kDa // 12p13.33 // 2288 /// ENS	2,25	LUMINAL A up vs CONTROL
SLC9A3R1	NM_004252 // SLC9A3R1 // solute carrier family 9 (sodium/hydrogen exchanger), me	2,25	LUMINAL A up vs CONTROL
PDCD6IP	NM_013374 // PDCD6IP // programmed cell death 6 interacting protein // 3p22.3 //	2,25	LUMINAL A up vs CONTROL
E2F5	NM_001951 // E2F5 // E2F transcription factor 5, p130-binding // 8q21.2 // 1875	2,25	LUMINAL A up vs CONTROL
UEVLD	NM_001040697 // UEVLD // UEV and lactate/malate dehyrogenase domains // 11p15.1	2,25	LUMINAL A up vs CONTROL
DDX5	NM_004396 // DDX5 // DEAD (Asp-Glu-Ala-Asp) box polypeptide 5 // 17q21 // 1655 /	2,25	LUMINAL A up vs CONTROL
IFITM1	NM_003641 // IFITM1 // interferon induced transmembrane protein 1 (9-27) // 11p1	2,25	LUMINAL A up vs CONTROL
C3orf10	NM_018462 // C3orf10 // chromosome 3 open reading frame 10 // 3p25.3 // 55845 //	2,24	LUMINAL A up vs CONTROL
CPNE1	NM_152930 // CPNE1 // copine I // 20q11.22 // 8904 /// NM_152931 // CPNE1 // cop	2,24	LUMINAL A up vs CONTROL
PIGK	NM_005482 // PIGK // phosphatidylinositol glycan anchor biosynthesis, class K //	2,24	LUMINAL A up vs CONTROL
PFDN1	NM_002622 // PFDN1 // prefoldin subunit 1 // 5q31 // 5201 /// ENST00000261813 //	2,24	LUMINAL A up vs CONTROL
LRRC6	NM_012472 // LRRC6 // leucine rich repeat containing 6 // 8q24.22 // 23639 /// E	2,24	LUMINAL A up vs CONTROL
RPF1	NM_025065 // RPF1 // ribosome production factor 1 homolog (S. cerevisiae) // 1p2	2,24	LUMINAL A up vs CONTROL
PGRMC1	NM_006667 // PGRMC1 // progesterone receptor membrane component 1 // Xq22-q24 //	2,24	LUMINAL A up vs CONTROL
ANKRA2	NM_023039 // ANKRA2 // ankyrin repeat, family A (RFXANK-like), 2 // 5q12-q13 //	2,24	LUMINAL A up vs CONTROL

ITK	NM_005546 // ITK // IL2-inducible T-cell kinase // 5q31-q32 // 3702 /// ENST0000	2,24	LUMINAL A up vs CONTROL
HERC4	NM_022079 // HERC4 // hect domain and RLD 4 // 10q21.3 // 26091 /// NM_015601 //	2,24	LUMINAL A up vs CONTROL
PPP3CA	NM_000944 // PPP3CA // protein phosphatase 3, catalytic subunit, alpha isozyme /	2,24	LUMINAL A up vs CONTROL
CCBL2	NM_001008661 // CCBL2 // cysteine conjugate-beta lyase 2 // 1p22.2 // 56267 ///	2,24	LUMINAL A up vs CONTROL
BPNT1	NM_006085 // BPNT1 // 3'(2'), 5'-bisphosphate nucleotidase 1 // 1q41 // 10380 //	2,24	LUMINAL A up vs CONTROL
GNPAT	NM_014236 // GNPAT // glyceronephosphate O-acyltransferase // 1q42 // 8443 /// E	2,24	LUMINAL A up vs CONTROL
PLRG1	NM_002669 // PLRG1 // pleiotropic regulator 1 (PRL1 homolog, Arabidopsis) // 4q3	2,23	LUMINAL A up vs CONTROL
SLC11A2	NM_001174129 // SLC11A2 // solute carrier family 11 (proton-coupled divalent met	2,23	LUMINAL A up vs CONTROL
DDX47	NM_016355 // DDX47 // DEAD (Asp-Glu-Ala-Asp) box polypeptide 47 // 12p13.1 // 51	2,23	LUMINAL A up vs CONTROL
LARP4B	NM_015155 // LARP4B // La ribonucleoprotein domain family, member 4B // 10p15.3	2,23	LUMINAL A up vs CONTROL
C1S	NM_201442 // C1S // complement component 1, s subcomponent // 12p13 // 716 /// N	2,23	LUMINAL A up vs CONTROL
MOCS2	NM_176806 // MOCS2 // molybdenum cofactor synthesis 2 // 5q11 // 4338 /// NM_004	2,23	LUMINAL A up vs CONTROL
SCYL3	NM_181093 // SCYL3 // SCY1-like 3 (S. cerevisiae) // 1q24.2 // 57147 /// NM_0204	2,23	LUMINAL A up vs CONTROL
TFB2M	NM_022366 // TFB2M // transcription factor B2, mitochondrial // 1q44 // 64216 //	2,23	LUMINAL A up vs CONTROL
PIK3CB	NM_006219 // PIK3CB // phosphoinositide-3-kinase, catalytic, beta polypeptide //	2,23	LUMINAL A up vs CONTROL
NIP7	NM_016101 // NIP7 // nuclear import 7 homolog (S. cerevisiae) // 16q22.1 // 5138	2,23	LUMINAL A up vs CONTROL
PSME1	NM_176783 // PSME1 // proteasome (prosome, macropain) activator subunit 1 (PA28	2,23	LUMINAL A up vs CONTROL
PUS7L	NM_001098615 // PUS7L // pseudouridylate synthase 7 homolog (S. cerevisiae)-like	2,23	LUMINAL A up vs CONTROL
SLC5A3	NM_006933 // SLC5A3 // solute carrier family 5 (sodium/myo-inositol cotransporte	2,23	LUMINAL A up vs CONTROL
AP1G1	NM_001030007 // AP1G1 // adaptor-related protein complex 1, gamma 1 subunit // 1	2,23	LUMINAL A up vs CONTROL
C3orf59	NM_178496 // C3orf59 // chromosome 3 open reading frame 59 // 3q29 // 151963 ///	2,23	LUMINAL A up vs CONTROL
XRN2	NM_012255 // XRN2 // 5'-3' exoribonuclease 2 // 20p11.2-p11.1 // 22803 /// ENST0	2,23	LUMINAL A up vs CONTROL
MCL1	NM_021960 // MCL1 // myeloid cell leukemia sequence 1 (BCL2-related) // 1q21 //	2,23	LUMINAL A up vs CONTROL
OSBPL8	NM_020841 // OSBPL8 // oxysterol binding protein-like 8 // 12q14 // 114882 /// N	2,23	LUMINAL A up vs CONTROL
MPZL2	NM_144765 // MPZL2 // myelin protein zero-like 2 // 11q24 // 10205 /// NM_005797	2,22	LUMINAL A up vs CONTROL
ACP1	NR_024080 // ACP1 // acid phosphatase 1, soluble // 2p25 // 52 /// NM_007099 //	2,22	LUMINAL A up vs CONTROL
TMEM45A	NM_018004 // TMEM45A // transmembrane protein 45A // 3q12.2 // 55076 /// ENST000	2,22	LUMINAL A up vs CONTROL
IKZF5	NM_022466 // IKZF5 // IKAROS family zinc finger 5 (Pegasus) // 10q26 // 64376 //	2,22	LUMINAL A up vs CONTROL
SNX4	NM_003794 // SNX4 // sorting nexin 4 // 3q21.2 // 8723 /// ENST00000251775 // SN	2,22	LUMINAL A up vs CONTROL
LOC100128868	AY143171 // LOC100128868 // testin-related protein TRG // 7q31.2 // 100128868	2,22	LUMINAL A up vs CONTROL
IARS	NM_013417 // IARS // isoleucyl-tRNA synthetase // 9q21 // 3376 /// NM_002161 //	2,22	LUMINAL A up vs CONTROL
KTN1	NM_182926 // KTN1 // kinectin 1 (kinesin receptor) // 14q22.1 // 3895 /// NM_001	2,22	LUMINAL A up vs CONTROL
POLB	NM_002690 // POLB // polymerase (DNA directed), beta // 8p11.2 // 5423 /// ENST0	2,22	LUMINAL A up vs CONTROL

MGST2	NM_002413 // MGST2 // microsomal glutathione S-transferase 2 // 4q28.3 // 4258 /	2,22	LUMINAL A up vs CONTROL
TSTA3	NM_003313 // TSTA3 // tissue specific transplantation antigen P35B // 8q24.3 //	2,22	LUMINAL A up vs CONTROL
IFNGR1	NM_000416 // IFNGR1 // interferon gamma receptor 1 // 6q23.3 // 3459 /// ENST000	2,22	LUMINAL A up vs CONTROL
GGCT	NM_024051 // GGCT // gamma-glutamylcyclotransferase // 7p15-p14 // 79017 /// ENS	2,22	LUMINAL A up vs CONTROL
PGAP2	NR_027017 // PGAP2 // post-GPI attachment to proteins 2 // 11p15.5 // 27315 ///	2,22	LUMINAL A up vs CONTROL
ABHD10	NM_018394 // ABHD10 // abhydrolase domain containing 10 // 3q13.2 // 55347 /// E	2,22	LUMINAL A up vs CONTROL
NIPA2	NM_030922 // NIPA2 // non imprinted in Prader-Willi/Angelman syndrome 2 // 15q11	2,22	LUMINAL A up vs CONTROL
APOOL	NM_198450 // APOOL // apolipoprotein O-like // Xq21.1 // 139322 /// ENST00000436	2,22	LUMINAL A up vs CONTROL
KLHDC2	NM_014315 // KLHDC2 // kelch domain containing 2 // 14q21.3 // 23588 /// ENST000	2,22	LUMINAL A up vs CONTROL
PHIP	NM_017934 // PHIP // pleckstrin homology domain interacting protein // 6q14 // 5	2,22	LUMINAL A up vs CONTROL
C5orf24	NM_001135586 // C5orf24 // chromosome 5 open reading frame 24 // 5q31.1 // 13455	2,21	LUMINAL A up vs CONTROL
GTF2H3	NM_001516 // GTF2H3 // general transcription factor IIH, polypeptide 3, 34kDa //	2,21	LUMINAL A up vs CONTROL
MRPL30	NM_145212 // MRPL30 // mitochondrial ribosomal protein L30 // 2q11.2 // 51263 //	2,21	LUMINAL A up vs CONTROL
FLVCR1	NM_014053 // FLVCR1 // feline leukemia virus subgroup C cellular receptor 1 // 1	2,21	LUMINAL A up vs CONTROL
CTBS	NM_004388 // CTBS // chitobiase, di-N-acetyl- // 1p22 // 1486 /// NM_001081472 /	2,21	LUMINAL A up vs CONTROL
DR1	NM_001938 // DR1 // down-regulator of transcription 1, TBP-binding (negative cof	2,21	LUMINAL A up vs CONTROL
ALG11	NM_001004127 // ALG11 // asparagine-linked glycosylation 11, alpha-1,2-mannosylt	2,21	LUMINAL A up vs CONTROL
DUSP11	NM_003584 // DUSP11 // dual specificity phosphatase 11 (RNA/RNP complex 1-intera	2,21	LUMINAL A up vs CONTROL
TRAT1	NM_016388 // TRAT1 // T cell receptor associated transmembrane adaptor 1 // 3q13	2,21	LUMINAL A up vs CONTROL
TRMT12	NM_017956 // TRMT12 // tRNA methyltransferase 12 homolog (S. cerevisiae) // 8q24	2,21	LUMINAL A up vs CONTROL
TXNDC9	NM_005783 // TXNDC9 // thioredoxin domain containing 9 // 2q11.2 // 10190 /// EN	2,21	LUMINAL A up vs CONTROL
CHORDC1	NM_012124 // CHORDC1 // cysteine and histidine-rich domain (CHORD)-containing 1	2,21	LUMINAL A up vs CONTROL
HSPA9	NM_004134 // HSPA9 // heat shock 70kDa protein 9 (mortalin) // 5q31.1 // 3313 //	2,21	LUMINAL A up vs CONTROL
GPRIN3	NM_198281 // GPRIN3 // GPRIN family member 3 // 4q22.1 // 285513 /// ENST0000033	2,21	LUMINAL A up vs CONTROL
HIST4H4	NM_175054 // HIST4H4 // histone cluster 4, H4 // 12p12.3 // 121504	2,21	LUMINAL A up vs CONTROL
MATR3	NM_199189 // MATR3 // matrin 3 // 5q31.2 // 9782 /// NM_018834 // MATR3 // matri	2,21	LUMINAL A up vs CONTROL
PHOSPHO2	NM_001008489 // PHOSPHO2 // phosphatase, orphan 2 // 2q31.1 // 493911 /// NM_144	2,20	LUMINAL A up vs CONTROL
TDP2	NM_016614 // TDP2 // tyrosyl-DNA phosphodiesterase 2 // 6p22.3-p22.1 // 51567 //	2,20	LUMINAL A up vs CONTROL
NGLY1	NM_018297 // NGLY1 // N-glycanase 1 // 3p24.2 // 55768 /// NM_001145293 // NGLY1	2,20	LUMINAL A up vs CONTROL
RAB27B	NM_004163 // RAB27B // RAB27B, member RAS oncogene family // 18q21.2 // 5874 ///	2,20	LUMINAL A up vs CONTROL
ZFC3H1	NM_144982 // ZFC3H1 // zinc finger, C3H1-type containing // 12q21.1 // 196441 //	2,20	LUMINAL A up vs CONTROL
ALDH18A1	NM_002860 // ALDH18A1 // aldehyde dehydrogenase 18 family, member A1 // 10q24.3	2,20	LUMINAL A up vs CONTROL
NUP133	NM_018230 // NUP133 // nucleoporin 133kDa // 1q42.13 // 55746 /// ENST0000026139	2,20	LUMINAL A up vs CONTROL

ZDHHC17	NM_015336 // ZDHHC17 // zinc finger, DHHC-type containing 17 // 12q21.2 // 23390	2,20	LUMINAL A up vs CONTROL
ID2	NM_002166 // ID2 // inhibitor of DNA binding 2, dominant negative helix-loop-hel	2,20	LUMINAL A up vs CONTROL
MRPS35	NM_021821 // MRPS35 // mitochondrial ribosomal protein S35 // 12p11 // 60488 ///	2,20	LUMINAL A up vs CONTROL
NME1	NM_198175 // NME1 // non-metastatic cells 1, protein (NM23A) expressed in // 17q	2,20	LUMINAL A up vs CONTROL
RNASEH2B	NM_024570 // RNASEH2B // ribonuclease H2, subunit B // 13q14.3 // 79621 /// NM_0	2,20	LUMINAL A up vs CONTROL
RACGAP1	NM_013277 // RACGAP1 // Rac GTPase activating protein 1 // 12q13.12 // 29127 ///	2,20	LUMINAL A up vs CONTROL
TSG101	NM_006292 // TSG101 // tumor susceptibility gene 101 // 11p15 // 7251 /// ENST00	2,20	LUMINAL A up vs CONTROL
KIAA0174	NM_014761 // KIAA0174 // KIAA0174 // 16q22.2 // 9798 /// ENST00000329908 // KIAA	2,20	LUMINAL A up vs CONTROL
TSPAN31	NM_005981 // TSPAN31 // tetraspanin 31 // 12q13.3 // 6302 /// ENST00000257910 //	2,20	LUMINAL A up vs CONTROL
PTK2	NM_153831 // PTK2 // PTK2 protein tyrosine kinase 2 // 8q24-qter // 5747 /// NM_	2,20	LUMINAL A up vs CONTROL
ITFG1	NM_030790 // ITFG1 // integrin alpha FG-GAP repeat containing 1 // 16q12.1 // 81	2,20	LUMINAL A up vs CONTROL
PRO2012	BC019830 // PRO2012 // hypothetical protein PRO2012 // 1q42.13 // 55478	2,19	LUMINAL A up vs CONTROL
CXXC5	NM_016463 // CXXC5 // CXXC finger 5 // 5q31.2 // 51523 /// ENST00000302517 // CX	2,19	LUMINAL A up vs CONTROL
ADAM28	NM_014265 // ADAM28 // ADAM metallopeptidase domain 28 // 8p21.2 // 10863 /// NM	2,19	LUMINAL A up vs CONTROL
MFSD11	NM_024311 // MFSD11 // major facilitator superfamily domain containing 11 // 17q	2,19	LUMINAL A up vs CONTROL
PAPD5	NM_001040284 // PAPD5 // PAP associated domain containing 5 // 16q12.1 // 64282	2,19	LUMINAL A up vs CONTROL
MED31	NM_016060 // MED31 // mediator complex subunit 31 // 17p13.1 // 51003 /// ENST00	2,19	LUMINAL A up vs CONTROL
HOOK1	NM_015888 // HOOK1 // hook homolog 1 (Drosophila) // 1p32.1 // 51361 /// ENST000	2,19	LUMINAL A up vs CONTROL
GPR155	NM_001033045 // GPR155 // G protein-coupled receptor 155 // 2q31.1 // 151556 ///	2,19	LUMINAL A up vs CONTROL
SMAD5	NM_001001419 // SMAD5 // SMAD family member 5 // 5q31 // 4090 /// NM_005903 // S	2,19	LUMINAL A up vs CONTROL
PRH1	NM_006250 // PRH1 // proline-rich protein HaeIII subfamily 1 // 12p13.2 // 5554	2,19	LUMINAL A up vs CONTROL
AKAP9	NM_005751 // AKAP9 // A kinase (PRKA) anchor protein (yotiao) 9 // 7q21-q22 // 1	2,19	LUMINAL A up vs CONTROL
SSBP2	NM_012446 // SSBP2 // single-stranded DNA binding protein 2 // 5q14.1 // 23635 /	2,19	LUMINAL A up vs CONTROL
SGK1	NM_001143676 // SGK1 // serum/glucocorticoid regulated kinase 1 // 6q23 // 6446	2,19	LUMINAL A up vs CONTROL
VAMP4	NM_003762 // VAMP4 // vesicle-associated membrane protein 4 // 1q24-q25 // 8674	2,19	LUMINAL A up vs CONTROL
TMED7	NM_181836 // TMED7 // transmembrane emp24 protein transport domain containing 7	2,19	LUMINAL A up vs CONTROL
UBR2	NM_015255 // UBR2 // ubiquitin protein ligase E3 component n-recognin 2 // 6p21.	2,18	LUMINAL A up vs CONTROL
PPIC	NM_000943 // PPIC // peptidylprolyl isomerase C (cyclophilin C) // 5q23.2 // 548	2,18	LUMINAL A up vs CONTROL
NUFIP2	NM_020772 // NUFIP2 // nuclear fragile X mental retardation protein interacting	2,18	LUMINAL A up vs CONTROL
APPBP2	NM_006380 // APPBP2 // amyloid beta precursor protein (cytoplasmic tail) binding	2,18	LUMINAL A up vs CONTROL
RNF141	NM_016422 // RNF141 // ring finger protein 141 // 11p15.4 // 50862 /// ENST00000	2,18	LUMINAL A up vs CONTROL
CKAP2	NM_018204 // CKAP2 // cytoskeleton associated protein 2 // 13q14 // 26586 /// NM	2,18	LUMINAL A up vs CONTROL
ITGBL1	NM_004791 // ITGBL1 // integrin, beta-like 1 (with EGF-like repeat domains) // 1	2,18	LUMINAL A up vs CONTROL

RGS5	NM_003617 // RGS5 // regulator of G-protein signaling 5 // 1q23.1 // 8490 /// EN	2,18	LUMINAL A up vs CONTROL
AP1AR	NM_018569 // AP1AR // adaptor-related protein complex 1 associated regulatory pr	2,18	LUMINAL A up vs CONTROL
TLR7	NM_016562 // TLR7 // toll-like receptor 7 // Xp22.3 // 51284 /// ENST00000380659	2,18	LUMINAL A up vs CONTROL
CCT7	NM_001166285 // CCT7 // chaperonin containing TCP1, subunit 7 (eta) // 2p13.2 //	2,18	LUMINAL A up vs CONTROL
RPN2	NM_002951 // RPN2 // ribophorin II // 20q12-q13.1 // 6185 /// NM_001135771 // RP	2,18	LUMINAL A up vs CONTROL
SAT1	NR_027783 // SAT1 // spermidine/spermine N1-acetyltransferase 1 // Xp22.1 // 630	2,18	LUMINAL A up vs CONTROL
PMS2	NM_000535 // PMS2 // PMS2 postmeiotic segregation increased 2 (S. cerevisiae) //	2,18	LUMINAL A up vs CONTROL
OPA1	NM_130837 // OPA1 // optic atrophy 1 (autosomal dominant) // 3q28-q29|3q28-q29 /	2,18	LUMINAL A up vs CONTROL
MUT	NM_000255 // MUT // methylmalonyl CoA mutase // 6p12.3 // 4594 /// ENST000002748	2,18	LUMINAL A up vs CONTROL
NEDD1	NM_152905 // NEDD1 // neural precursor cell expressed, developmentally down-regu	2,18	LUMINAL A up vs CONTROL
BCAP29	NM_018844 // BCAP29 // B-cell receptor-associated protein 29 // 7q22-q31 // 5597	2,18	LUMINAL A up vs CONTROL
YTHDF1	NM_017798 // YTHDF1 // YTH domain family, member 1 // 20q13.33 // 54915 /// ENST	2,17	LUMINAL A up vs CONTROL
SNX27	NM_030918 // SNX27 // sorting nexin family member 27 // 1q21.3 // 81609 /// ENST	2,17	LUMINAL A up vs CONTROL
CREG1	NM_003851 // CREG1 // cellular repressor of E1A-stimulated genes 1 // 1q24 // 88	2,17	LUMINAL A up vs CONTROL
DDX6	NM_004397 // DDX6 // DEAD (Asp-Glu-Ala-Asp) box polypeptide 6 // 11q23.3 // 1656	2,17	LUMINAL A up vs CONTROL
CCL5	NM_002985 // CCL5 // chemokine (C-C motif) ligand 5 // 17q11.2-q12 // 6352 /// E	2,17	LUMINAL A up vs CONTROL
ANXA5	NM_001154 // ANXA5 // annexin A5 // 4q26-q28|4q28-q32 // 308 /// ENST00000296511	2,17	LUMINAL A up vs CONTROL
TWSG1	NM_020648 // TWSG1 // twisted gastrulation homolog 1 (Drosophila) // 18p11.3 //	2,17	LUMINAL A up vs CONTROL
TRIM22	NM_006074 // TRIM22 // tripartite motif-containing 22 // 11p15 // 10346 /// ENST	2,17	LUMINAL A up vs CONTROL
AMN1	NM_001113402 // AMN1 // antagonist of mitotic exit network 1 homolog (S. cerevis	2,17	LUMINAL A up vs CONTROL
07/set	NM_001788 // SEPT7 // septin 7 // 7p14.3-p14.1 // 989 /// NM_001011553 // SEPT7	2,17	LUMINAL A up vs CONTROL
GTF2H5	NM_207118 // GTF2H5 // general transcription factor IIH, polypeptide 5 // 6q25.3	2,17	LUMINAL A up vs CONTROL
PSMB1	NM_002793 // PSMB1 // proteasome (prosome, macropain) subunit, beta type, 1 // 6	2,17	LUMINAL A up vs CONTROL
SPRYD4	NM_207344 // SPRYD4 // SPRY domain containing 4 // 12q13.3 // 283377 /// ENST000	2,17	LUMINAL A up vs CONTROL
UBA6	NM_018227 // UBA6 // ubiquitin-like modifier activating enzyme 6 // 4q13.2 // 55	2,17	LUMINAL A up vs CONTROL
ILF2	NM_004515 // ILF2 // interleukin enhancer binding factor 2, 45kDa // 1q21.3 // 3	2,17	LUMINAL A up vs CONTROL
NF1	NM_001042492 // NF1 // neurofibromin 1 // 17q11.2 // 4763 /// NM_000267 // NF1 /	2,17	LUMINAL A up vs CONTROL
ZMYM2	NM_003453 // ZMYM2 // zinc finger, MYM-type 2 // 13q11-q12 // 7750 /// NM_197968	2,16	LUMINAL A up vs CONTROL
SEC63	NM_007214 // SEC63 // SEC63 homolog (S. cerevisiae) // 6q21 // 11231 /// ENST000	2,16	LUMINAL A up vs CONTROL
PCNP	NM_020357 // PCNP // PEST proteolytic signal containing nuclear protein // 3q12.	2,16	LUMINAL A up vs CONTROL
IFIT5	NM_012420 // IFIT5 // interferon-induced protein with tetratricopeptide repeats	2,16	LUMINAL A up vs CONTROL
DOCK2	NM_004946 // DOCK2 // dedicator of cytokinesis 2 // 5q35.1 // 1794 /// ENST00000	2,16	LUMINAL A up vs CONTROL
CCDC76	NM_019083 // CCDC76 // coiled-coil domain containing 76 // 1pter-q31.3 // 54482	2,16	LUMINAL A up vs CONTROL

ZNF528	NM_032423 // ZNF528 // zinc finger protein 528 // 19q13 // 84436 /// AK056552 //	2,16	LUMINAL A up vs CONTROL
ELMOD2	NM_153702 // ELMOD2 // ELMO/CED-12 domain containing 2 // 4q31.1 // 255520 /// E	2,16	LUMINAL A up vs CONTROL
TBC1D15	NM_022771 // TBC1D15 // TBC1 domain family, member 15 // 12q21.1 // 64786 /// NM	2,16	LUMINAL A up vs CONTROL
SPG21	NM_001127889 // SPG21 // spastic paraplegia 21 (autosomal recessive, Mast syndro	2,16	LUMINAL A up vs CONTROL
PSMD5	NM_005047 // PSMD5 // proteasome (prosome, macropain) 26S subunit, non-ATPase, 5	2,16	LUMINAL A up vs CONTROL
AP1M2	NM_005498 // AP1M2 // adaptor-related protein complex 1, mu 2 subunit // 19p13.2	2,16	LUMINAL A up vs CONTROL
DEPDC6	NM_022783 // DEPDC6 // DEP domain containing 6 // 8q24.12 // 64798 /// ENST00000	2,16	LUMINAL A up vs CONTROL
ARF1	NM_001024226 // ARF1 // ADP-ribosylation factor 1 // 1q42 // 375 /// NM_00102422	2,16	LUMINAL A up vs CONTROL
C10orf119	NM_024834 // C10orf119 // chromosome 10 open reading frame 119 // 10q26.11 // 79	2,16	LUMINAL A up vs CONTROL
BTN3A2	NM_007047 // BTN3A2 // butyrophilin, subfamily 3, member A2 // 6p22.1 // 11118 /	2,16	LUMINAL A up vs CONTROL
YIPF1	NM_018982 // YIPF1 // Yip1 domain family, member 1 // 1p33-p32.1 // 54432 /// EN	2,16	LUMINAL A up vs CONTROL
NKAIN1	NM_024522 // NKAIN1 // Na+/K+ transporting ATPase interacting 1 // 1p35.2 // 795	2,16	LUMINAL A up vs CONTROL
C19orf2	NM_003796 // C19orf2 // chromosome 19 open reading frame 2 // 19q12 // 8725 ///	2,16	LUMINAL A up vs CONTROL
WDR41	NM_018268 // WDR41 // WD repeat domain 41 // 5q13.3 // 55255 /// ENST00000296679	2,16	LUMINAL A up vs CONTROL
RBM12B	NM_203390 // RBM12B // RNA binding motif protein 12B // 8q22.1 // 389677 /// ENS	2,15	LUMINAL A up vs CONTROL
MORC3	NM_015358 // MORC3 // MORC family CW-type zinc finger 3 // 21q22.13 // 23515 ///	2,15	LUMINAL A up vs CONTROL
STAU2	NM_014393 // STAU2 // staufen, RNA binding protein, homolog 2 (Drosophila) // 8q	2,15	LUMINAL A up vs CONTROL
ATP8A1	NM_006095 // ATP8A1 // ATPase, aminophospholipid transporter (APLT), class I, ty	2,15	LUMINAL A up vs CONTROL
NNT	NM_012343 // NNT // nicotinamide nucleotide transhydrogenase // 5p13.1-cen // 23	2,15	LUMINAL A up vs CONTROL
RAB27A	NM_004580 // RAB27A // RAB27A, member RAS oncogene family // 15q15-q21.1 // 5873	2,15	LUMINAL A up vs CONTROL
KPNB1	NM_002265 // KPNB1 // karyopherin (importin) beta 1 // 17q21.32 // 3837 /// ENST	2,15	LUMINAL A up vs CONTROL
ATG3	NM_022488 // ATG3 // ATG3 autophagy related 3 homolog (S. cerevisiae) // 3q13.2	2,15	LUMINAL A up vs CONTROL
SFRS12IP1	NM_173829 // SFRS12IP1 // SFRS12-interacting protein 1 // 5q12.3 // 285672 /// E	2,15	LUMINAL A up vs CONTROL
NUP37	NM_024057 // NUP37 // nucleoporin 37kDa // 12q23.2 // 79023 /// ENST00000251074	2,15	LUMINAL A up vs CONTROL
STK39	NM_013233 // STK39 // serine threonine kinase 39 (STE20/SPS1 homolog, yeast) //	2,15	LUMINAL A up vs CONTROL
POC5	NM_001099271 // POC5 // POC5 centriolar protein homolog (Chlamydomonas) // 5q13.	2,15	LUMINAL A up vs CONTROL
SLAMF8	NM_020125 // SLAMF8 // SLAM family member 8 // 1q23.2 // 56833 /// ENST000002897	2,15	LUMINAL A up vs CONTROL
GIMAP2	NM_015660 // GIMAP2 // GTPase, IMAP family member 2 // 7q36.1 // 26157 /// ENST0	2,15	LUMINAL A up vs CONTROL
CORO1A	NM_007074 // CORO1A // coronin, actin binding protein, 1A // 16p11.2 // 11151 //	2,15	LUMINAL A up vs CONTROL
GOLGA5	NM_005113 // GOLGA5 // golgin A5 // 14q32.12-q32.13 // 9950 /// ENST00000163416	2,15	LUMINAL A up vs CONTROL
EVI2A	NM_001003927 // EVI2A // ecotropic viral integration site 2A // 17q11.2 // 2123	2,15	LUMINAL A up vs CONTROL
XPOT	NM_007235 // XPOT // exportin, tRNA (nuclear export receptor for tRNAs) // 12q14	2,15	LUMINAL A up vs CONTROL
C5orf43	NM_001048249 // C5orf43 // chromosome 5 open reading frame 43 // 5q12.1 // 64315	2,15	LUMINAL A up vs CONTROL

ORC3L	NM_181837 // ORC3L // origin recognition complex, subunit 3-like (yeast) // 6q14	2,15	LUMINAL A up vs CONTROL
TARBP1	NM_005646 // TARBP1 // TAR (HIV-1) RNA binding protein 1 // 1q42.3 // 6894 /// E	2,15	LUMINAL A up vs CONTROL
SLC39A11	NM_001159770 // SLC39A11 // solute carrier family 39 (metal ion transporter), me	2,15	LUMINAL A up vs CONTROL
DCP2	NM_152624 // DCP2 // DCP2 decapping enzyme homolog (S. cerevisiae) // 5q22.2 //	2,15	LUMINAL A up vs CONTROL
IGBP1	NM_001551 // IGBP1 // immunoglobulin (CD79A) binding protein 1 // Xq13.1-q13.3 /	2,15	LUMINAL A up vs CONTROL
LRRC40	NM_017768 // LRRC40 // leucine rich repeat containing 40 // 1p31.1 // 55631 ///	2,14	LUMINAL A up vs CONTROL
ZNF638	NM_014497 // ZNF638 // zinc finger protein 638 // 2p13.2-p13.1 // 27332 /// NM_0	2,14	LUMINAL A up vs CONTROL
TMEM165	NM_018475 // TMEM165 // transmembrane protein 165 // 4q12 // 55858 /// ENST00000	2,14	LUMINAL A up vs CONTROL
PCMTD2	NM_018257 // PCMTD2 // protein-L-isoaspartate (D-aspartate) O-methyltransferase	2,14	LUMINAL A up vs CONTROL
GSTCD	NM_001031720 // GSTCD // glutathione S-transferase, C-terminal domain containing	2,14	LUMINAL A up vs CONTROL
RUNX2	NM_001024630 // RUNX2 // runt-related transcription factor 2 // 6p21 // 860 ///	2,14	LUMINAL A up vs CONTROL
ZNF627	NM_145295 // ZNF627 // zinc finger protein 627 // 19p13.2 // 199692 /// ENST0000	2,14	LUMINAL A up vs CONTROL
FKBP1A	NM_000801 // FKBP1A // FK506 binding protein 1A, 12kDa // 20p13 // 2280 /// NM_0	2,14	LUMINAL A up vs CONTROL
MTMR6	NM_004685 // MTMR6 // myotubularin related protein 6 // 13q12 // 9107 /// ENST00	2,14	LUMINAL A up vs CONTROL
DENND2D	NM_024901 // DENND2D // DENN/MADD domain containing 2D // 1p13.3 // 79961 /// EN	2,14	LUMINAL A up vs CONTROL
C11orf54	NM_014039 // C11orf54 // chromosome 11 open reading frame 54 // 11q21 // 28970 /	2,14	LUMINAL A up vs CONTROL
TGFBI	NM_000358 // TGFBI // transforming growth factor, beta-induced, 68kDa // 5q31 //	2,14	LUMINAL A up vs CONTROL
JAK2	NM_004972 // JAK2 // Janus kinase 2 // 9p24 // 3717 /// ENST00000381652 // JAK2	2,14	LUMINAL A up vs CONTROL
CEP350	NM_014810 // CEP350 // centrosomal protein 350kDa // 1p36.13-q41 // 9857 /// ENS	2,14	LUMINAL A up vs CONTROL
ANXA4	NM_001153 // ANXA4 // annexin A4 // 2p13 // 307 /// ENST00000394295 // ANXA4 //	2,14	LUMINAL A up vs CONTROL
EXOC5	NM_006544 // EXOC5 // exocyst complex component 5 // 14q22.3 // 10640 /// ENST00	2,14	LUMINAL A up vs CONTROL
SNX2	NM_003100 // SNX2 // sorting nexin 2 // 5q23 // 6643 /// ENST00000379516 // SNX2	2,13	LUMINAL A up vs CONTROL
KCNJ3	NM_002239 // KCNJ3 // potassium inwardly-rectifying channel, subfamily J, member	2,13	LUMINAL A up vs CONTROL
FBXO7	NM_012179 // FBXO7 // F-box protein 7 // 22q12-q13 // 25793 /// NM_001033024 //	2,13	LUMINAL A up vs CONTROL
ERAP2	NM_022350 // ERAP2 // endoplasmic reticulum aminopeptidase 2 // 5q15 // 64167 //	2,13	LUMINAL A up vs CONTROL
LY86	NM_004271 // LY86 // lymphocyte antigen 86 // 6p25.1 // 9450 /// ENST00000379953	2,13	LUMINAL A up vs CONTROL
KIAA1524	NM_020890 // KIAA1524 // KIAA1524 // 3q13.13 // 57650 /// ENST00000295746 // KIA	2,13	LUMINAL A up vs CONTROL
MMP7	NM_002423 // MMP7 // matrix metallopeptidase 7 (matrilysin, uterine) // 11q21-q2	2,13	LUMINAL A up vs CONTROL
KIF3A	NM_007054 // KIF3A // kinesin family member 3A // 5q31 // 11127 /// ENST00000378	2,13	LUMINAL A up vs CONTROL
CD55	NM_001114752 // CD55 // CD55 molecule, decay accelerating factor for complement	2,13	LUMINAL A up vs CONTROL
TMEM154	NM_152680 // TMEM154 // transmembrane protein 154 // 4q31.3 // 201799 /// ENST00	2,13	LUMINAL A up vs CONTROL
UBE2V2	NM_003350 // UBE2V2 // ubiquitin-conjugating enzyme E2 variant 2 // 8q11.21 // 7	2,13	LUMINAL A up vs CONTROL
GPN3	NM_001164372 // GPN3 // GPN-loop GTPase 3 // 12q24.11 // 51184 /// NM_001164373	2,13	LUMINAL A up vs CONTROL

ZNHIT6	NM_017953 // ZNHIT6 // zinc finger, HIT type 6 // 1p22.3 // 54680 /// NM_0011706	2,13	LUMINAL A up vs CONTROL
LPCAT3	NM_005768 // LPCAT3 // lysophosphatidylcholine acyltransferase 3 // 12p13 // 101	2,13	LUMINAL A up vs CONTROL
TRAFD1	NM_001143906 // TRAFD1 // TRAF-type zinc finger domain containing 1 // 12q // 10	2,13	LUMINAL A up vs CONTROL
FIBIN	NM_203371 // FIBIN // fin bud initiation factor homolog (zebrafish) // 11p14.2 /	2,13	LUMINAL A up vs CONTROL
PDIA6	NM_005742 // PDIA6 // protein disulfide isomerase family A, member 6 // 2p25.1 /	2,13	LUMINAL A up vs CONTROL
PEX1	NM_000466 // PEX1 // peroxisomal biogenesis factor 1 // 7q21.2 // 5189 /// ENST0	2,13	LUMINAL A up vs CONTROL
ANKRD49	NM_017704 // ANKRD49 // ankyrin repeat domain 49 // 11q21 // 54851 /// ENST00000	2,13	LUMINAL A up vs CONTROL
C9orf80	NM_021218 // C9orf80 // chromosome 9 open reading frame 80 // 9q32 // 58493 ///	2,13	LUMINAL A up vs CONTROL
NUCB2	NM_005013 // NUCB2 // nucleobindin 2 // 11p15.1-p14 // 4925 /// ENST00000323688	2,13	LUMINAL A up vs CONTROL
TMEM168	NM_022484 // TMEM168 // transmembrane protein 168 // 7q31.32 // 64418 /// ENST00	2,13	LUMINAL A up vs CONTROL
C12orf23	NM_152261 // C12orf23 // chromosome 12 open reading frame 23 // 12q23.3 // 90488	2,13	LUMINAL A up vs CONTROL
TBCE	NM_001079515 // TBCE // tubulin folding cofactor E // 1q42.3 // 6905 /// NM_0031	2,13	LUMINAL A up vs CONTROL
CTSD	NM_001909 // CTSD // cathepsin D // 11p15.5 // 1509 /// ENST00000236671 // CTSD	2,12	LUMINAL A up vs CONTROL
SNAP23	NM_003825 // SNAP23 // synaptosomal-associated protein, 23kDa // 15q15.1-q15.2 /	2,12	LUMINAL A up vs CONTROL
UBE2E1	NM_003341 // UBE2E1 // ubiquitin-conjugating enzyme E2E 1 (UBC4/5 homolog, yeast	2,12	LUMINAL A up vs CONTROL
LSM14A	NM_001114093 // LSM14A // LSM14A, SCD6 homolog A (S. cerevisiae) // 19q13.11 //	2,12	LUMINAL A up vs CONTROL
AQR	NM_014691 // AQR // aquarius homolog (mouse) // 15q14 // 9716 /// ENST0000015647	2,12	LUMINAL A up vs CONTROL
GFM1	NM_024996 // GFM1 // G elongation factor, mitochondrial 1 // 3q25.1-q26.2 // 854	2,12	LUMINAL A up vs CONTROL
FAM174A	NM_198507 // FAM174A // family with sequence similarity 174, member A // 5q21.1	2,12	LUMINAL A up vs CONTROL
TRAPPC3	NM_014408 // TRAPPC3 // trafficking protein particle complex 3 // 1p34.3 // 2709	2,12	LUMINAL A up vs CONTROL
ARHGAP5	NM_001030055 // ARHGAP5 // Rho GTPase activating protein 5 // 14q12 // 394 /// N	2,12	LUMINAL A up vs CONTROL
SSBP1	NM_003143 // SSBP1 // single-stranded DNA binding protein 1 // 7q34 // 6742 ///	2,12	LUMINAL A up vs CONTROL
ATF2	NM_001880 // ATF2 // activating transcription factor 2 // 2q32 // 1386 /// ENST0	2,12	LUMINAL A up vs CONTROL
INTS12	NM_020395 // INTS12 // integrator complex subunit 12 // 4q24 // 57117 /// NM_001	2,12	LUMINAL A up vs CONTROL
SMAD2	NM_005901 // SMAD2 // SMAD family member 2 // 18q21.1 // 4087 /// NM_001003652 /	2,12	LUMINAL A up vs CONTROL
ZNF217	NM_006526 // ZNF217 // zinc finger protein 217 // 20q13.2 // 7764 /// ENST000003	2,12	LUMINAL A up vs CONTROL
CCDC109B	NM_017918 // CCDC109B // coiled-coil domain containing 109B // 4q25 // 55013 ///	2,12	LUMINAL A up vs CONTROL
USP16	NM_006447 // USP16 // ubiquitin specific peptidase 16 // 21q22.11 // 10600 /// N	2,12	LUMINAL A up vs CONTROL
SNX14	NM_153816 // SNX14 // sorting nexin 14 // 6q14.3 // 57231 /// NM_020468 // SNX14	2,12	LUMINAL A up vs CONTROL
NT5C3	NR_029372 // NT5C3 // 5'-nucleotidase, cytosolic III // 7p14.3 // 51251 /// NM_0	2,12	LUMINAL A up vs CONTROL
CUL3	NM_003590 // CUL3 // cullin 3 // 2q36.2 // 8452 /// ENST00000264414 // CUL3 // c	2,12	LUMINAL A up vs CONTROL
KMO	NM_003679 // KMO // kynurenine 3-monooxygenase (kynurenine 3-hydroxylase) // 1q4	2,12	LUMINAL A up vs CONTROL
ZG16B	NM_145252 // ZG16B // zymogen granule protein 16 homolog B (rat) // 16p13.3 // 1	2,12	LUMINAL A up vs CONTROL

MYD88	NM_001172567 // MYD88 // myeloid differentiation primary response gene (88) // 3	2,12	LUMINAL A up vs CONTROL
SPARCL1	NM_001128310 // SPARCL1 // SPARC-like 1 (hevin) // 4q22.1 // 8404 /// NM_004684	2,12	LUMINAL A up vs CONTROL
C3orf52	NM_024616 // C3orf52 // chromosome 3 open reading frame 52 // 3q13.2 // 79669 //	2,12	LUMINAL A up vs CONTROL
COG3	NM_031431 // COG3 // component of oligomeric golgi complex 3 // 13q14.13 // 8354	2,11	LUMINAL A up vs CONTROL
ECT2	NM_018098 // ECT2 // epithelial cell transforming sequence 2 oncogene // 3q26.1-	2,11	LUMINAL A up vs CONTROL
GK	NM_001128127 // GK // glycerol kinase // Xp21.3 // 2710 /// NM_203391 // GK // g	2,11	LUMINAL A up vs CONTROL
SPTLC3	NM_018327 // SPTLC3 // serine palmitoyltransferase, long chain base subunit 3 //	2,11	LUMINAL A up vs CONTROL
NAA50	NM_025146 // NAA50 // N(alpha)-acetyltransferase 50, NatE catalytic subunit // 3	2,11	LUMINAL A up vs CONTROL
COMMD6	NM_203497 // COMMD6 // COMM domain containing 6 // 13q22 // 170622 /// NM_203495	2,11	LUMINAL A up vs CONTROL
NAALADL2	NM_207015 // NAALADL2 // N-acetylated alpha-linked acidic dipeptidase-like 2 //	2,11	LUMINAL A up vs CONTROL
CD63	NM_001780 // CD63 // CD63 molecule // 12q12-q13 // 967 /// NM_001040034 // CD63	2,11	LUMINAL A up vs CONTROL
KCMF1	NM_020122 // KCMF1 // potassium channel modulatory factor 1 // 2p11.2 // 56888 /	2,11	LUMINAL A up vs CONTROL
CXorf26	BC001220 // CXorf26 // chromosome X open reading frame 26 // Xq13.3 // 51260 ///	2,11	LUMINAL A up vs CONTROL
FASTKD2	NM_001136194 // FASTKD2 // FAST kinase domains 2 // 2q33.3 // 22868 /// NM_00113	2,11	LUMINAL A up vs CONTROL
PCNA	NM_002592 // PCNA // proliferating cell nuclear antigen // 20pter-p12 // 5111 //	2,11	LUMINAL A up vs CONTROL
CST4	NM_001899 // CST4 // cystatin S // 20p11.21 // 1472 /// ENST00000217423 // CST4	2,11	LUMINAL A up vs CONTROL
NUSAP1	NM_016359 // NUSAP1 // nucleolar and spindle associated protein 1 // 15q15.1 //	2,11	LUMINAL A up vs CONTROL
MUDENG	NM_018229 // MUDENG // MU-2/AP1M2 domain containing, death-inducing // 14q22.3 /	2,10	LUMINAL A up vs CONTROL
NME7	NM_013330 // NME7 // non-metastatic cells 7, protein expressed in (nucleoside-di	2,10	LUMINAL A up vs CONTROL
STAG2	NM_001042750 // STAG2 // stromal antigen 2 // Xq25 // 10735 /// NM_001042749 //	2,10	LUMINAL A up vs CONTROL
COL5A2	NM_000393 // COL5A2 // collagen, type V, alpha 2 // 2q14-q32 // 1290 /// ENST000	2,10	LUMINAL A up vs CONTROL
ALG1	NM_019109 // ALG1 // asparagine-linked glycosylation 1, beta-1,4-mannosyltransfe	2,10	LUMINAL A up vs CONTROL
KCNJ8	NM_004982 // KCNJ8 // potassium inwardly-rectifying channel, subfamily J, member	2,10	LUMINAL A up vs CONTROL
NIPBL	NM_015384 // NIPBL // Nipped-B homolog (Drosophila) // 5p13.2 // 25836 /// NM_13	2,10	LUMINAL A up vs CONTROL
CLDN4	NM_001305 // CLDN4 // claudin 4 // 7q11.23 // 1364 /// ENST00000435050 // CLDN4	2,10	LUMINAL A up vs CONTROL
VBP1	NM_003372 // VBP1 // von Hippel-Lindau binding protein 1 // Xq28 // 7411 /// ENS	2,10	LUMINAL A up vs CONTROL
C3	NM_000064 // C3 // complement component 3 // 19p13.3-p13.2 // 718 /// ENST000002	2,10	LUMINAL A up vs CONTROL
PPP6C	NM_001123355 // PPP6C // protein phosphatase 6, catalytic subunit // 9q33.3 // 5	2,10	LUMINAL A up vs CONTROL
SFT2D1	NM_145169 // SFT2D1 // SFT2 domain containing 1 // 6q27 // 113402 /// ENST000003	2,10	LUMINAL A up vs CONTROL
PMPCB	NM_004279 // PMPCB // peptidase (mitochondrial processing) beta // 7q22.1 // 951	2,10	LUMINAL A up vs CONTROL
ATP13A3	NM_024524 // ATP13A3 // ATPase type 13A3 // 3q29 // 79572 /// ENST00000439040 //	2,10	LUMINAL A up vs CONTROL
CDC40	NM_015891 // CDC40 // cell division cycle 40 homolog (S. cerevisiae) // 6q21 //	2,10	LUMINAL A up vs CONTROL
C12orf51	NM_001109662 // C12orf51 // chromosome 12 open reading frame 51 // 12q24.13 // 2	2,10	LUMINAL A up vs CONTROL

DEGS1	NM_003676 // DEGS1 // degenerative spermatocyte homolog 1, lipid desaturase (Dro	2,10	LUMINAL A up vs CONTROL
UBB	NM_018955 // UBB // ubiquitin B // 17p12-p11.2 // 7314 /// BC000379 // UBB // ub	2,10	LUMINAL A up vs CONTROL
SYAP1	NM_032796 // SYAP1 // synapse associated protein 1, SAP47 homolog (Drosophila) /	2,09	LUMINAL A up vs CONTROL
FLRT3	NM_198391 // FLRT3 // fibronectin leucine rich transmembrane protein 3 // 20p11	2,09	LUMINAL A up vs CONTROL
STMN1	NM_203401 // STMN1 // stathmin 1 // 1p36.1-p35 // 3925 /// NM_005563 // STMN1 //	2,09	LUMINAL A up vs CONTROL
FUBP1	NM_003902 // FUBP1 // far upstream element (FUSE) binding protein 1 // 1p31.1 //	2,09	LUMINAL A up vs CONTROL
MPHOSPH9	NM_022782 // MPHOSPH9 // M-phase phosphoprotein 9 // 12q24.31 // 10198 /// ENST0	2,09	LUMINAL A up vs CONTROL
CTSH	NM_004390 // CTSH // cathepsin H // 15q24-q25 // 1512 /// ENST00000220166 // CTS	2,09	LUMINAL A up vs CONTROL
EPSTI1	NM_001002264 // EPSTI1 // epithelial stromal interaction 1 (breast) // 13q13.3 /	2,09	LUMINAL A up vs CONTROL
JMJD1C	NM_032776 // JMJD1C // jumonji domain containing 1C // 10q21.3 // 221037 /// NM_	2,09	LUMINAL A up vs CONTROL
THY1	NM_006288 // THY1 // Thy-1 cell surface antigen // 11q22.3-q23 // 7070 /// ENST0	2,09	LUMINAL A up vs CONTROL
MIF	NM_002415 // MIF // macrophage migration inhibitory factor (glycosylation-inhibi	2,09	LUMINAL A up vs CONTROL
CTPS2	AF226667 // CTPS2 // CTP synthase II // Xp22 // 56474 /// ENST00000380207 // CTP	2,09	LUMINAL A up vs CONTROL
POLR3K	NM_016310 // POLR3K // polymerase (RNA) III (DNA directed) polypeptide K, 12.3 k	2,09	LUMINAL A up vs CONTROL
CDC42SE1	NM_001038707 // CDC42SE1 // CDC42 small effector 1 // 1q21.3 // 56882 /// NM_020	2,09	LUMINAL A up vs CONTROL
ZNF525	NR_003699 // ZNF525 // zinc finger protein 525 // 19q13.42 // 170958 /// NM_1383	2,09	LUMINAL A up vs CONTROL
FUNDC1	NM_173794 // FUNDC1 // FUN14 domain containing 1 // Xp11.3 // 139341 /// ENST000	2,09	LUMINAL A up vs CONTROL
CCNE2	NM_057749 // CCNE2 // cyclin E2 // 8q22.1 // 9134 /// ENST00000308108 // CCNE2 /	2,09	LUMINAL A up vs CONTROL
KDM6A	NM_021140 // KDM6A // lysine (K)-specific demethylase 6A // Xp11.2 // 7403 /// E	2,09	LUMINAL A up vs CONTROL
AHNAK2	NM_138420 // AHNAK2 // AHNAK nucleoprotein 2 // 14q32.33 // 113146 /// ENST00000	2,09	LUMINAL A up vs CONTROL
ZNRD1	NM_170783 // ZNRD1 // zinc ribbon domain containing 1 // 6p21.3 // 30834 /// NM_	2,09	LUMINAL A up vs CONTROL
LYPLAL1	NM_138794 // LYPLAL1 // lysophospholipase-like 1 // 1q41 // 127018 /// ENST00000	2,09	LUMINAL A up vs CONTROL
TTC39B	NM_152574 // TTC39B // tetratricopeptide repeat domain 39B // 9p22.3 // 158219 /	2,09	LUMINAL A up vs CONTROL
NR1D2	NM_005126 // NR1D2 // nuclear receptor subfamily 1, group D, member 2 // 3p24.2	2,09	LUMINAL A up vs CONTROL
USP7	NM_003470 // USP7 // ubiquitin specific peptidase 7 (herpes virus-associated) //	2,09	LUMINAL A up vs CONTROL
BBX	NM_001142568 // BBX // bobby sox homolog (Drosophila) // 3q13.1 // 56987 /// NM_	2,08	LUMINAL A up vs CONTROL
C5orf30	BC009203 // C5orf30 // chromosome 5 open reading frame 30 // 5q21.1 // 90355 ///	2,08	LUMINAL A up vs CONTROL
COPS5	NM_006837 // COPS5 // COP9 constitutive photomorphogenic homolog subunit 5 (Arab	2,08	LUMINAL A up vs CONTROL
WIPF1	NM_003387 // WIPF1 // WAS/WASL interacting protein family, member 1 // 2q31.1 //	2,08	LUMINAL A up vs CONTROL
OCR1	AF314543 // OCR1 // ovarian cancer-related protein 1 // 1q32.1 // 100128298	2,08	LUMINAL A up vs CONTROL
ZRANB2	NM_005455 // ZRANB2 // zinc finger, RAN-binding domain containing 2 // 1p31 // 9	2,08	LUMINAL A up vs CONTROL
VTCN1	NM_024626 // VTCN1 // V-set domain containing T cell activation inhibitor 1 // 1	2,08	LUMINAL A up vs CONTROL
CNOT6	NM_015455 // CNOT6 // CCR4-NOT transcription complex, subunit 6 // 5q35.3 // 574	2,08	LUMINAL A up vs CONTROL

GAPT	NM_152687 // GAPT // GRB2-binding adaptor protein, transmembrane // 5q11.2 // 20	2,08	LUMINAL A up vs CONTROL
PAK1IP1	NM_017906 // PAK1IP1 // PAK1 interacting protein 1 // 6p24.2 // 55003 /// ENST00	2,08	LUMINAL A up vs CONTROL
PGAP1	NM_024989 // PGAP1 // post-GPI attachment to proteins 1 // 2q33.1 // 80055 /// E	2,07	LUMINAL A up vs CONTROL
YIPF4	NM_032312 // YIPF4 // Yip1 domain family, member 4 // 2p22.3 // 84272 /// ENST00	2,07	LUMINAL A up vs CONTROL
C8orf38	NM_152416 // C8orf38 // chromosome 8 open reading frame 38 // 8q22.1 // 137682 /	2,07	LUMINAL A up vs CONTROL
TRIP12	NM_004238 // TRIP12 // thyroid hormone receptor interactor 12 // 2q36.3 // 9320	2,07	LUMINAL A up vs CONTROL
ANO10	NM_018075 // ANO10 // anoctamin 10 // 3p22.1 // 55129 /// ENST00000292246 // ANO	2,07	LUMINAL A up vs CONTROL
PTER	NM_030664 // PTER // phosphotriesterase related // 10p12 // 9317 /// NM_00100148	2,07	LUMINAL A up vs CONTROL
ATP2C1	NM_014382 // ATP2C1 // ATPase, Ca++ transporting, type 2C, member 1 // 3q22.1 //	2,07	LUMINAL A up vs CONTROL
EXOSC1	NM_016046 // EXOSC1 // exosome component 1 // 10q24 // 51013 /// ENST00000370902	2,07	LUMINAL A up vs CONTROL
PPHLN1	NM_016488 // PPHLN1 // periphilin 1 // 12q12 // 51535 /// NM_001143787 // PPHLN1	2,07	LUMINAL A up vs CONTROL
CNOT2	NM_014515 // CNOT2 // CCR4-NOT transcription complex, subunit 2 // 12q15 // 4848	2,07	LUMINAL A up vs CONTROL
TCN1	NM_001062 // TCN1 // transcobalamin I (vitamin B12 binding protein, R binder fam	2,07	LUMINAL A up vs CONTROL
KIAA0391	NM_014672 // KIAA0391 // KIAA0391 // 14q13.2 // 9692 /// ENST00000250377 // KIAA	2,07	LUMINAL A up vs CONTROL
MTIF2	NM_002453 // MTIF2 // mitochondrial translational initiation factor 2 // 2p14-p1	2,07	LUMINAL A up vs CONTROL
BARD1	NM_000465 // BARD1 // BRCA1 associated RING domain 1 // 2q34-q35 // 580 /// ENST	2,07	LUMINAL A up vs CONTROL
ACAP2	NM_012287 // ACAP2 // ArfGAP with coiled-coil, ankyrin repeat and PH domains 2 /	2,07	LUMINAL A up vs CONTROL
HBP1	NM_012257 // HBP1 // HMG-box transcription factor 1 // 7q22-q31 // 26959 /// NM_	2,07	LUMINAL A up vs CONTROL
BFAR	NM_016561 // BFAR // bifunctional apoptosis regulator // 16p13.12 // 51283 /// E	2,07	LUMINAL A up vs CONTROL
HEATR1	NM_018072 // HEATR1 // HEAT repeat containing 1 // 1q43 // 55127 /// ENST0000036	2,06	LUMINAL A up vs CONTROL
SEC22A	NM_012430 // SEC22A // SEC22 vesicle trafficking protein homolog A (S. cerevisia	2,06	LUMINAL A up vs CONTROL
RPL14	NM_001034996 // RPL14 // ribosomal protein L14 // 3p22-p21.2 // 9045 /// NM_0039	2,06	LUMINAL A up vs CONTROL
CKAP5	NM_001008938 // CKAP5 // cytoskeleton associated protein 5 // 11p11.2 // 9793 //	2,06	LUMINAL A up vs CONTROL
ERO1L	NM_014584 // ERO1L // ERO1-like (S. cerevisiae) // 14q22.1 // 30001 /// ENST0000	2,06	LUMINAL A up vs CONTROL
GDF9	NM_005260 // GDF9 // growth differentiation factor 9 // 5q31.1 // 2661 /// ENST0	2,06	LUMINAL A up vs CONTROL
SMC3	NM_005445 // SMC3 // structural maintenance of chromosomes 3 // 10q25 // 9126 //	2,06	LUMINAL A up vs CONTROL
INSIG2	NM_016133 // INSIG2 // insulin induced gene 2 // 2q14.2 // 51141 /// ENST0000024	2,06	LUMINAL A up vs CONTROL
SMARCAD1	NM_001128429 // SMARCAD1 // SWI/SNF-related, matrix-associated actin-dependent r	2,06	LUMINAL A up vs CONTROL
DSN1	NM_001145316 // DSN1 // DSN1, MIND kinetochore complex component, homolog (S. ce	2,06	LUMINAL A up vs CONTROL
ELP3	NM_018091 // ELP3 // elongation protein 3 homolog (S. cerevisiae) // 8p21.1 // 5	2,06	LUMINAL A up vs CONTROL
ATG2B	NM_018036 // ATG2B // ATG2 autophagy related 2 homolog B (S. cerevisiae) // 14q3	2,06	LUMINAL A up vs CONTROL
VCAM1	NM_001078 // VCAM1 // vascular cell adhesion molecule 1 // 1p32-p31 // 7412 ///	2,06	LUMINAL A up vs CONTROL
SMG1	NM_015092 // SMG1 // SMG1 homolog, phosphatidylinositol 3-kinase-related kinase	2,06	LUMINAL A up vs CONTROL

MMP16	NM_005941 // MMP16 // matrix metallopeptidase 16 (membrane-inserted) // 8q21.3 /	2,06	LUMINAL A up vs CONTROL
POMP	NM_015932 // POMP // proteasome maturation protein // 13q12.3 // 51371 /// ENST0	2,06	LUMINAL A up vs CONTROL
UFD1L	NM_005659 // UFD1L // ubiquitin fusion degradation 1 like (yeast) // 22q11.21 //	2,06	LUMINAL A up vs CONTROL
ENAH	NM_001008493 // ENAH // enabled homolog (Drosophila) // 1q42.12 // 55740 /// NM_	2,06	LUMINAL A up vs CONTROL
EIF2AK1	NM_014413 // EIF2AK1 // eukaryotic translation initiation factor 2-alpha kinase	2,06	LUMINAL A up vs CONTROL
TLK1	NM_012290 // TLK1 // tousled-like kinase 1 // 2q31.1 // 9874 /// NM_001136554 //	2,06	LUMINAL A up vs CONTROL
ZNF678	NR_033184 // ZNF678 // zinc finger protein 678 // 1q42.13 // 339500 /// NM_17854	2,06	LUMINAL A up vs CONTROL
MAPRE1	NM_012325 // MAPRE1 // microtubule-associated protein, RP/EB family, member 1 //	2,06	LUMINAL A up vs CONTROL
G2E3	NM_017769 // G2E3 // G2/M-phase specific E3 ubiquitin ligase // 14q12 // 55632 /	2,06	LUMINAL A up vs CONTROL
SOCS4	NM_199421 // SOCS4 // suppressor of cytokine signaling 4 // 14q22.2-q22.3 // 122	2,05	LUMINAL A up vs CONTROL
ASPM	NM_018136 // ASPM // asp (abnormal spindle) homolog, microcephaly associated (Dr	2,05	LUMINAL A up vs CONTROL
LONRF2	NM_198461 // LONRF2 // LON peptidase N-terminal domain and ring finger 2 // 2q11	2,05	LUMINAL A up vs CONTROL
KRAS	NM_033360 // KRAS // v-Ki-ras2 Kirsten rat sarcoma viral oncogene homolog // 12p	2,05	LUMINAL A up vs CONTROL
HERC6	NM_017912 // HERC6 // hect domain and RLD 6 // 4q22.1 // 55008 /// NM_001165136	2,05	LUMINAL A up vs CONTROL
ZDHHC6	NM_022494 // ZDHHC6 // zinc finger, DHHC-type containing 6 // 10q25.2 // 64429 /	2,05	LUMINAL A up vs CONTROL
DHRS7	NM_016029 // DHRS7 // dehydrogenase/reductase (SDR family) member 7 // 14q23.1 /	2,05	LUMINAL A up vs CONTROL
WSB1	NM_015626 // WSB1 // WD repeat and SOCS box-containing 1 // 17q11.1 // 26118 ///	2,05	LUMINAL A up vs CONTROL
NXT2	NM_018698 // NXT2 // nuclear transport factor 2-like export factor 2 // Xq23 //	2,05	LUMINAL A up vs CONTROL
UNC50	NM_014044 // UNC50 // unc-50 homolog (C. elegans) // 2q11.2 // 25972 /// ENST000	2,05	LUMINAL A up vs CONTROL
IBTK	NM_015525 // IBTK // inhibitor of Bruton agammaglobulinemia tyrosine kinase // 6	2,05	LUMINAL A up vs CONTROL
PIGY	NM_032906 // PIGY // phosphatidylinositol glycan anchor biosynthesis, class Y //	2,04	LUMINAL A up vs CONTROL
DNAJC12	NM_021800 // DNAJC12 // DnaJ (Hsp40) homolog, subfamily C, member 12 // 10q22.1	2,04	LUMINAL A up vs CONTROL
TAP1	NM_000593 // TAP1 // transporter 1, ATP-binding cassette, sub-family B (MDR/TAP)	2,04	LUMINAL A up vs CONTROL
C5orf22	NM_018356 // C5orf22 // chromosome 5 open reading frame 22 // 5p13.3 // 55322 //	2,04	LUMINAL A up vs CONTROL
GBP1	NM_002053 // GBP1 // guanylate binding protein 1, interferon-inducible, 67kDa //	2,04	LUMINAL A up vs CONTROL
MRS2	NM_020662 // MRS2 // MRS2 magnesium homeostasis factor homolog (S. cerevisiae) /	2,04	LUMINAL A up vs CONTROL
HSD17B12	NM_016142 // HSD17B12 // hydroxysteroid (17-beta) dehydrogenase 12 // 11p11.2 //	2,04	LUMINAL A up vs CONTROL
HEATR1	NM_018072 // HEATR1 // HEAT repeat containing 1 // 1q43 // 55127 /// ENST0000036	2,04	LUMINAL A up vs CONTROL
SPPL2A	NM_032802 // SPPL2A // signal peptide peptidase-like 2A // 15q21.2 // 84888 ///	2,04	LUMINAL A up vs CONTROL
TARDBP	NM_007375 // TARDBP // TAR DNA binding protein // 1p36.22 // 23435 /// ENST00000	2,04	LUMINAL A up vs CONTROL
TM4SF1	NM_014220 // TM4SF1 // transmembrane 4 L six family member 1 // 3q21-q25 // 4071	2,04	LUMINAL A up vs CONTROL
TMEM69	NM_016486 // TMEM69 // transmembrane protein 69 // 1p34.1 // 51249 /// ENST00000	2,04	LUMINAL A up vs CONTROL
RAPGEF6	NM_001164386 // RAPGEF6 // Rap guanine nucleotide exchange factor (GEF) 6 // 5q3	2,04	LUMINAL A up vs CONTROL

DMTF1	NR_024549 // DMTF1 // cyclin D binding myb-like transcription factor 1 // 7q21 /	2,04	LUMINAL A up vs CONTROL
FCER1G	NM_004106 // FCER1G // Fc fragment of IgE, high affinity I, receptor for; gamma	2,04	LUMINAL A up vs CONTROL
PPIL3	NM_130906 // PPIL3 // peptidylprolyl isomerase (cyclophilin)-like 3 // 2q33.1 //	2,04	LUMINAL A up vs CONTROL
HTATIP2	NM_001098522 // HTATIP2 // HIV-1 Tat interactive protein 2, 30kDa // 11p15.1 //	2,04	LUMINAL A up vs CONTROL
DDX46	NM_014829 // DDX46 // DEAD (Asp-Glu-Ala-Asp) box polypeptide 46 // 5q31.1 // 987	2,04	LUMINAL A up vs CONTROL
THADA	NM_022065 // THADA // thyroid adenoma associated // 2p21 // 63892 /// NM_0010839	2,04	LUMINAL A up vs CONTROL
PACRGL	NM_145048 // PACRGL // PARK2 co-regulated-like // 4p15.31 // 133015 /// NM_00113	2,04	LUMINAL A up vs CONTROL
ATP1A1	NM_000701 // ATP1A1 // ATPase, Na+/K+ transporting, alpha 1 polypeptide // 1p21	2,04	LUMINAL A up vs CONTROL
LUC7L3	NM_016424 // LUC7L3 // LUC7-like 3 (S. cerevisiae) // 17q21.33 // 51747 /// NM_0	2,04	LUMINAL A up vs CONTROL
STEAP4	NM_024636 // STEAP4 // STEAP family member 4 // 7q21.12 // 79689 /// ENST0000038	2,04	LUMINAL A up vs CONTROL
CSTF3	NM_001326 // CSTF3 // cleavage stimulation factor, 3' pre-RNA, subunit 3, 77kDa	2,04	LUMINAL A up vs CONTROL
ANKRD46	NM_198401 // ANKRD46 // ankyrin repeat domain 46 // 8q22.2 // 157567 /// ENST000	2,03	LUMINAL A up vs CONTROL
C5orf33	NM_001085411 // C5orf33 // chromosome 5 open reading frame 33 // 5p13.2 // 13368	2,03	LUMINAL A up vs CONTROL
NDUFA6	NM_002490 // NDUFA6 // NADH dehydrogenase (ubiquinone) 1 alpha subcomplex, 6, 14	2,03	LUMINAL A up vs CONTROL
CDS1	NM_001263 // CDS1 // CDP-diacylglycerol synthase (phosphatidate cytidylyltransfe	2,03	LUMINAL A up vs CONTROL
CCR4	NM_005508 // CCR4 // chemokine (C-C motif) receptor 4 // 3p24 // 1233 /// ENST00	2,03	LUMINAL A up vs CONTROL
PIK3C3	NM_002647 // PIK3C3 // phosphoinositide-3-kinase, class 3 // 18q12.3 // 5289 ///	2,03	LUMINAL A up vs CONTROL
SFRS13A	NM_054016 // SFRS13A // splicing factor, arginine/serine-rich 13A // 1p36.11 //	2,03	LUMINAL A up vs CONTROL
VRK1	NM_003384 // VRK1 // vaccinia related kinase 1 // 14q32 // 7443 /// ENST00000216	2,03	LUMINAL A up vs CONTROL
CD74	NM_001025159 // CD74 // CD74 molecule, major histocompatibility complex, class I	2,03	LUMINAL A up vs CONTROL
VPS26A	NM_004896 // VPS26A // vacuolar protein sorting 26 homolog A (S. pombe) // 10q21	2,03	LUMINAL A up vs CONTROL
LHFPL2	NM_005779 // LHFPL2 // lipoma HMGIC fusion partner-like 2 // 5q14.1 // 10184 ///	2,03	LUMINAL A up vs CONTROL
LPCAT2	NM_017839 // LPCAT2 // lysophosphatidylcholine acyltransferase 2 // 16q12.2 // 5	2,03	LUMINAL A up vs CONTROL
FAM165B	NM_058182 // FAM165B // family with sequence similarity 165, member B // 21q22.1	2,03	LUMINAL A up vs CONTROL
RPP30	NM_001104546 // RPP30 // ribonuclease P/MRP 30kDa subunit // 10q23.31 // 10556 /	2,03	LUMINAL A up vs CONTROL
PVALB	NM_002854 // PVALB // parvalbumin // 22q12-q13.1|22q13.1 // 5816 /// ENST0000021	2,03	LUMINAL A up vs CONTROL
TMEM56	NM_152487 // TMEM56 // transmembrane protein 56 // 1p21.3 // 148534 /// ENST0000	2,03	LUMINAL A up vs CONTROL
MUC15	NM_001135091 // MUC15 // mucin 15, cell surface associated // 11p14.3 // 143662	2,03	LUMINAL A up vs CONTROL
KNTC1	NM_014708 // KNTC1 // kinetochore associated 1 // 12q24.31 // 9735 /// ENST00000	2,03	LUMINAL A up vs CONTROL
MAPK1	NM_002745 // MAPK1 // mitogen-activated protein kinase 1 // 22q11.2|22q11.21 //	2,02	LUMINAL A up vs CONTROL
GMFB	NM_004124 // GMFB // glia maturation factor, beta // 14q22.2 // 2764 /// ENST000	2,02	LUMINAL A up vs CONTROL
COIL	NM_004645 // COIL // coilin // 17q22-q23 // 8161 /// ENST00000240316 // COIL //	2,02	LUMINAL A up vs CONTROL
ERP27	NM_152321 // ERP27 // endoplasmic reticulum protein 27 // 12p12.3 // 121506 ///	2,02	LUMINAL A up vs CONTROL

SERPINE2	NM_001136529 // SERPINE2 // serpin peptidase inhibitor, clade E (nexin, plasmino	2,02	LUMINAL A up vs CONTROL
MAPK9	NM_139068 // MAPK9 // mitogen-activated protein kinase 9 // 5q35 // 5601 /// NM_	2,02	LUMINAL A up vs CONTROL
ADAR	NM_001111 // ADAR // adenosine deaminase, RNA-specific // 1q21.1-q21.2 // 103 //	2,02	LUMINAL A up vs CONTROL
TRA2A	AB052759 // TRA2A // transformer 2 alpha homolog (Drosophila) // 7p15.3 // 29896	2,02	LUMINAL A up vs CONTROL
NSUN3	NM_022072 // NSUN3 // NOP2/Sun domain family, member 3 // 3q11.1 // 63899 /// EN	2,02	LUMINAL A up vs CONTROL
GCA	NM_012198 // GCA // grancalcin, EF-hand calcium binding protein // 2q24.2 // 258	2,02	LUMINAL A up vs CONTROL
ERAP1	NM_001040458 // ERAP1 // endoplasmic reticulum aminopeptidase 1 // 5q15 // 51752	2,02	LUMINAL A up vs CONTROL
HIST1H3F	NM_021018 // HIST1H3F // histone cluster 1, H3f // 6p22.2 // 8968 /// BC096131 /	2,02	LUMINAL A up vs CONTROL
NIPSNAP3A	NM_015469 // NIPSNAP3A // nipsnap homolog 3A (C. elegans) // 9q31.1 // 25934 ///	2,02	LUMINAL A up vs CONTROL
APEX1	NM_001641 // APEX1 // APEX nuclease (multifunctional DNA repair enzyme) 1 // 14q	2,02	LUMINAL A up vs CONTROL
PIP5K1A	NM_001135638 // PIP5K1A // phosphatidylinositol-4-phosphate 5-kinase, type I, al	2,02	LUMINAL A up vs CONTROL
TAPT1	NM_153365 // TAPT1 // transmembrane anterior posterior transformation 1 // 4p15.	2,02	LUMINAL A up vs CONTROL
CAPRIN1	NM_005898 // CAPRIN1 // cell cycle associated protein 1 // 11p13 // 4076 /// NM_	2,02	LUMINAL A up vs CONTROL
TRIP11	NM_004239 // TRIP11 // thyroid hormone receptor interactor 11 // 14q31-q32 // 93	2,01	LUMINAL A up vs CONTROL
HIST1H2AE	NM_021052 // HIST1H2AE // histone cluster 1, H2ae // 6p22.2-p21.1 // 3012 /// BC	2,01	LUMINAL A up vs CONTROL
ZNF623	NM_001082480 // ZNF623 // zinc finger protein 623 // 8q24.3 // 9831 /// NM_01478	2,01	LUMINAL A up vs CONTROL
NMD3	NM_015938 // NMD3 // NMD3 homolog (S. cerevisiae) // 3q26.1 // 51068 /// ENST000	2,01	LUMINAL A up vs CONTROL
CHUK	NM_001278 // CHUK // conserved helix-loop-helix ubiquitous kinase // 10q24-q25 /	2,01	LUMINAL A up vs CONTROL
TPP2	NM_003291 // TPP2 // tripeptidyl peptidase II // 13q32-q33 // 7174 /// ENST00000	2,01	LUMINAL A up vs CONTROL
PPP4R1	NM_001042388 // PPP4R1 // protein phosphatase 4, regulatory subunit 1 // 18p11.2	2,01	LUMINAL A up vs CONTROL
BMPR1B	NM_001203 // BMPR1B // bone morphogenetic protein receptor, type IB // 4q22-q24	2,01	LUMINAL A up vs CONTROL
CTSC	NM_001814 // CTSC // cathepsin C // 11q14.1-q14.3 // 1075 /// NM_148170 // CTSC	2,01	LUMINAL A up vs CONTROL
PRPF39	NM_017922 // PRPF39 // PRP39 pre-mRNA processing factor 39 homolog (S. cerevisia	2,01	LUMINAL A up vs CONTROL
PARP8	NM_024615 // PARP8 // poly (ADP-ribose) polymerase family, member 8 // 5q11.1 //	2,01	LUMINAL A up vs CONTROL
TUBA4A	NM_006000 // TUBA4A // tubulin, alpha 4a // 2q35 // 7277 /// NR_026909 // STK16	2,01	LUMINAL A up vs CONTROL
TMEM116	NM_138341 // TMEM116 // transmembrane protein 116 // 12q24.13 // 89894 /// ENST0	2,01	LUMINAL A up vs CONTROL
TRIM37	NM_015294 // TRIM37 // tripartite motif-containing 37 // 17q23.2 // 4591 /// NM_	2,01	LUMINAL A up vs CONTROL
DHX36	NM_020865 // DHX36 // DEAH (Asp-Glu-Ala-His) box polypeptide 36 // 3p13-q23 // 1	2,01	LUMINAL A up vs CONTROL
ZNF678	NM_178549 // ZNF678 // zinc finger protein 678 // 1q42.13 // 339500 /// NR_03318	2,01	LUMINAL A up vs CONTROL
MANBA	NM_005908 // MANBA // mannosidase, beta A, lysosomal // 4q22-q25 // 4126 /// ENS	2,01	LUMINAL A up vs CONTROL
SEC62	NM_003262 // SEC62 // SEC62 homolog (S. cerevisiae) // 3q26.2 // 7095 /// ENST00	2,01	LUMINAL A up vs CONTROL
DYNC1LI1	NM_016141 // DYNC1LI1 // dynein, cytoplasmic 1, light intermediate chain 1 // 3p	2,01	LUMINAL A up vs CONTROL
EFHC1	NR_033327 // EFHC1 // EF-hand domain (C-terminal) containing 1 // 6p12.3 // 1143	2,01	LUMINAL A up vs CONTROL

HMGB2	NM_001130688 // HMGB2 // high-mobility group box 2 // 4q31 // 3148 /// NM_002129	2,01	LUMINAL A up vs CONTROL
FAM134B	NM_001034850 // FAM134B // family with sequence similarity 134, member B // 5p15	2,01	LUMINAL A up vs CONTROL
G3BP2	NM_203505 // G3BP2 // GTPase activating protein (SH3 domain) binding protein 2 /	2,01	LUMINAL A up vs CONTROL
NDUFA9	NM_005002 // NDUFA9 // NADH dehydrogenase (ubiquinone) 1 alpha subcomplex, 9, 39	2,01	LUMINAL A up vs CONTROL
TLR3	NM_003265 // TLR3 // toll-like receptor 3 // 4q35 // 7098 /// ENST00000296795 //	2,00	LUMINAL A up vs CONTROL
RALB	NM_002881 // RALB // v-ral simian leukemia viral oncogene homolog B (ras related	2,00	LUMINAL A up vs CONTROL
PSMG2	NM_020232 // PSMG2 // proteasome (prosome, macropain) assembly chaperone 2 // 18	2,00	LUMINAL A up vs CONTROL
HMCN1	NM_031935 // HMCN1 // hemicentin 1 // 1q25.3-q31.1 // 83872 /// ENST00000271588	2,00	LUMINAL A up vs CONTROL
TRIM33	NM_015906 // TRIM33 // tripartite motif-containing 33 // 1p13.1 // 51592 /// NM_	2,00	LUMINAL A up vs CONTROL
TCTN2	NM_024809 // TCTN2 // tectonic family member 2 // 12q24.31 // 79867 /// NM_00114	2,00	LUMINAL A up vs CONTROL
APPL1	NM_012096 // APPL1 // adaptor protein, phosphotyrosine interaction, PH domain an	2,00	LUMINAL A up vs CONTROL
DDX52	NM_152300 // DDX52 // DEAD (Asp-Glu-Ala-Asp) box polypeptide 52 // 17q21.1 // 11	2,00	LUMINAL A up vs CONTROL
ZCCHC17	NM_016505 // ZCCHC17 // zinc finger, CCHC domain containing 17 // 1p35.2 // 5153	2,00	LUMINAL A up vs CONTROL
RANBP2	NM_006267 // RANBP2 // RAN binding protein 2 // 2q12.3 // 5903 /// NM_005054 //	2,00	LUMINAL A up vs CONTROL
MRPS30	NM_016640 // MRPS30 // mitochondrial ribosomal protein S30 // 5q11 // 10884 ///	2,00	LUMINAL A up vs CONTROL
ISCU	NM_014301 // ISCU // iron-sulfur cluster scaffold homolog (E. coli) // 12q24.1 /	2,00	LUMINAL A up vs CONTROL
YTHDC2	NM_022828 // YTHDC2 // YTH domain containing 2 // 5q22.2 // 64848 /// ENST000001	2,00	LUMINAL A up vs CONTROL
PTPRJ	NM_002843 // PTPRJ // protein tyrosine phosphatase, receptor type, J // 11p11.2	2,00	LUMINAL A up vs CONTROL
SPTLC2	NM_004863 // SPTLC2 // serine palmitoyltransferase, long chain base subunit 2 //	2,00	LUMINAL A up vs CONTROL
ZC3H7A	NM_014153 // ZC3H7A // zinc finger CCCH-type containing 7A // 16p13-p12 // 29066	2,00	LUMINAL A up vs CONTROL
NDUFA12	NM_018838 // NDUFA12 // NADH dehydrogenase (ubiquinone) 1 alpha subcomplex, 12 /	2,00	LUMINAL A up vs CONTROL
TAS2R3	NM_016943 // TAS2R3 // taste receptor, type 2, member 3 // 7q31.3-q32 // 50831 /	2,00	LUMINAL A up vs CONTROL
CSNK1G3	NM_004384 // CSNK1G3 // casein kinase 1, gamma 3 // 5q23 // 1456 /// NM_00104472	2,00	LUMINAL A up vs CONTROL
CNOT6L	NM_144571 // CNOT6L // CCR4-NOT transcription complex, subunit 6-like // 4q13.3	2,00	LUMINAL A up vs CONTROL
02/set	NM_001008491 // SEPT2 // septin 2 // 2q37 // 4735 /// NM_001008492 // SEPT2 // s	2,00	LUMINAL A up vs CONTROL
INTS2	NR_026641 // INTS2 // integrator complex subunit 2 // 17q23.2 // 57508 /// NM_02	2,00	LUMINAL A up vs CONTROL
CETN2	NM_004344 // CETN2 // centrin, EF-hand protein, 2 // Xq28 // 1069 /// ENST000003	2,00	LUMINAL A up vs CONTROL
ANKRD28	NM_015199 // ANKRD28 // ankyrin repeat domain 28 // 3p25.1 // 23243 /// ENST0000	2,00	LUMINAL A up vs CONTROL
COPS2	NM_004236 // COPS2 // COP9 constitutive photomorphogenic homolog subunit 2 (Arab	2,00	LUMINAL A up vs CONTROL
FAM48A	NM_001014286 // FAM48A // family with sequence similarity 48, member A // 13q13.	2,00	LUMINAL A up vs CONTROL
ADK	NM_006721 // ADK // adenosine kinase // 10q22|10q11-q24 // 132 /// NM_001123 //	2,00	LUMINAL A up vs CONTROL
KCNA2	NM_004974 // KCNA2 // potassium voltage-gated channel, shaker-related subfamily,	-2,00	LUMINAL A down vs CONTROL
PCP4	NM_006198 // PCP4 // Purkinje cell protein 4 // 21q22.2 // 5121 /// ENST00000328	-2,00	LUMINAL A down vs CONTROL

ALDH3B2	NM_000695 // ALDH3B2 // aldehyde dehydrogenase 3 family, member B2 // 11q13 // 2	-2,00	LUMINAL A down vs CONTROL
C3orf36	NM_025041 // C3orf36 // chromosome 3 open reading frame 36 // 3q22.1 // 80111 //	-2,00	LUMINAL A down vs CONTROL
02/mar	NM_016496 // MARCH2 // membrane-associated ring finger (C3HC4) 2 // 19p13.2 // 5	-2,00	LUMINAL A down vs CONTROL
LCE5A	NM_178438 // LCE5A // late cornified envelope 5A // 1q21.3 // 254910 /// ENST000	-2,00	LUMINAL A down vs CONTROL
GIPC2	NM_017655 // GIPC2 // GIPC PDZ domain containing family, member 2 // 1p31.1 // 5	-2,00	LUMINAL A down vs CONTROL
MAMDC2	NM_153267 // MAMDC2 // MAM domain containing 2 // 9q21.12 // 256691 /// NM_00098	-2,00	LUMINAL A down vs CONTROL
PACSIN3	NM_016223 // PACSIN3 // protein kinase C and casein kinase substrate in neurons	-2,00	LUMINAL A down vs CONTROL
C6orf174	NM_001012279 // C6orf174 // chromosome 6 open reading frame 174 // 6q22.33 // 38	-2,00	LUMINAL A down vs CONTROL
ETFB	NM_001985 // ETFB // electron-transfer-flavoprotein, beta polypeptide // 19q13.3	-2,01	LUMINAL A down vs CONTROL
HIP1R	NM_003959 // HIP1R // huntingtin interacting protein 1 related // 12q24 // 9026	-2,01	LUMINAL A down vs CONTROL
CORO6	NM_032854 // CORO6 // coronin 6 // 17q11.2 // 84940 /// ENST00000345068 // CORO6	-2,01	LUMINAL A down vs CONTROL
AGT	NM_000029 // AGT // angiotensinogen (serpin peptidase inhibitor, clade A, member	-2,01	LUMINAL A down vs CONTROL
BIN3	NM_018688 // BIN3 // bridging integrator 3 // 8p21.3 // 55909 /// ENST0000027641	-2,01	LUMINAL A down vs CONTROL
MOCS1	NM_005943 // MOCS1 // molybdenum cofactor synthesis 1 // 6p21.3 // 4337 /// NM_0	-2,01	LUMINAL A down vs CONTROL
ITSN1	NM_003024 // ITSN1 // intersectin 1 (SH3 domain protein) // 21q22.1-q22.2 // 645	-2,01	LUMINAL A down vs CONTROL
FAT2	NM_001447 // FAT2 // FAT tumor suppressor homolog 2 (Drosophila) // 5q32-q33 //	-2,01	LUMINAL A down vs CONTROL
MYL7	NM_021223 // MYL7 // myosin, light chain 7, regulatory // 7p21-p11.2 // 58498 //	-2,01	LUMINAL A down vs CONTROL
FAM10A4	NR_002183 // FAM10A4 // ST13-like tumor suppressor // 13q14.3 // 145165 /// NM_0	-2,01	LUMINAL A down vs CONTROL
PSMB6	NM_002798 // PSMB6 // proteasome (prosome, macropain) subunit, beta type, 6 // 1	-2,01	LUMINAL A down vs CONTROL
CCRL2	NM_003965 // CCRL2 // chemokine (C-C motif) receptor-like 2 // 3p21 // 9034 ///	-2,01	LUMINAL A down vs CONTROL
GTSCR1	AY262164 // GTSCR1 // Gilles de la Tourette syndrome chromosome region, candidat	-2,01	LUMINAL A down vs CONTROL
INO80E	NM_173618 // INO80E // INO80 complex subunit E // 16p11.2 // 283899 /// ENST0000	-2,01	LUMINAL A down vs CONTROL
MPST	NR_024038 // MPST // mercaptopyruvate sulfurtransferase // 22q13.1 // 4357 /// N	-2,02	LUMINAL A down vs CONTROL
C14orf139	NR_026779 // C14orf139 // chromosome 14 open reading frame 139 // 14q32.13 // 79	-2,02	LUMINAL A down vs CONTROL
AKR1C3	NM_003739 // AKR1C3 // aldo-keto reductase family 1, member C3 (3-alpha hydroxys	-2,02	LUMINAL A down vs CONTROL
SAMD4A	NM_015589 // SAMD4A // sterile alpha motif domain containing 4A // 14q22.2 // 23	-2,02	LUMINAL A down vs CONTROL
CDK5R1	NM_003885 // CDK5R1 // cyclin-dependent kinase 5, regulatory subunit 1 (p35) //	-2,02	LUMINAL A down vs CONTROL
KPNA2	NM_002266 // KPNA2 // karyopherin alpha 2 (RAG cohort 1, importin alpha 1) // 17	-2,02	LUMINAL A down vs CONTROL
SLC25A18	NM_031481 // SLC25A18 // solute carrier family 25 (mitochondrial carrier), membe	-2,02	LUMINAL A down vs CONTROL
STX1B	NM_052874 // STX1B // syntaxin 1B // 16p11.2 // 112755 /// ENST00000215095 // ST	-2,02	LUMINAL A down vs CONTROL
AK5	NM_174858 // AK5 // adenylate kinase 5 // 1p31 // 26289 /// NM_012093 // AK5 //	-2,02	LUMINAL A down vs CONTROL
KIAA1683	NM_001145304 // KIAA1683 // KIAA1683 // 19p13.1 // 80726 /// NM_025249 // KIAA16	-2,02	LUMINAL A down vs CONTROL
NOTCH3	NM_000435 // NOTCH3 // Notch homolog 3 (Drosophila) // 19p13.2-p13.1 // 4854 ///	-2,02	LUMINAL A down vs CONTROL

CDC37	NM_007065 // CDC37 // cell division cycle 37 homolog (S. cerevisiae) // 19p13.2	-2,02	LUMINAL A down vs CONTROL
CNOT3	NM_014516 // CNOT3 // CCR4-NOT transcription complex, subunit 3 // 19q13.4 // 48	-2,02	LUMINAL A down vs CONTROL
TAF1C	NM_005679 // TAF1C // TATA box binding protein (TBP)-associated factor, RNA poly	-2,02	LUMINAL A down vs CONTROL
PLEKHG2	NM_022835 // PLEKHG2 // pleckstrin homology domain containing, family G (with Rh	-2,02	LUMINAL A down vs CONTROL
MLLT1	NM_005934 // MLLT1 // myeloid/lymphoid or mixed-lineage leukemia (trithorax homo	-2,02	LUMINAL A down vs CONTROL
IRF1	NM_002198 // IRF1 // interferon regulatory factor 1 // 5q31.1 // 3659 /// ENST00	-2,02	LUMINAL A down vs CONTROL
MPPED2	NM_001584 // MPPED2 // metallophosphoesterase domain containing 2 // 11p13 // 74	-2,02	LUMINAL A down vs CONTROL
CDC42EP2	NM_006779 // CDC42EP2 // CDC42 effector protein (Rho GTPase binding) 2 // 11q13	-2,02	LUMINAL A down vs CONTROL
BCL6B	NM_181844 // BCL6B // B-cell CLL/lymphoma 6, member B // 17p13.1 // 255877 /// E	-2,02	LUMINAL A down vs CONTROL
ALPL	NM_000478 // ALPL // alkaline phosphatase, liver/bone/kidney // 1p36.12 // 249 /	-2,02	LUMINAL A down vs CONTROL
04/set	NM_080415 // SEPT4 // septin 4 // 17q22-q23 // 5414 /// NM_004574 // SEPT4 // se	-2,02	LUMINAL A down vs CONTROL
IRX1	NM_024337 // IRX1 // iroquois homeobox 1 // 5p15.3 // 79192 /// NM_024336 // IRX	-2,02	LUMINAL A down vs CONTROL
ITGB4	NM_000213 // ITGB4 // integrin, beta 4 // 17q25 // 3691 /// NM_001005731 // ITGB	-2,02	LUMINAL A down vs CONTROL
05/set	NM_002688 // SEPT5 // septin 5 // 22q11.21 // 5413 /// ENST00000455784 // SEPT5	-2,03	LUMINAL A down vs CONTROL
HOXA5	NM_019102 // HOXA5 // homeobox A5 // 7p15-p14 // 3202 /// ENST00000222726 // HOX	-2,03	LUMINAL A down vs CONTROL
CHST3	NM_004273 // CHST3 // carbohydrate (chondroitin 6) sulfotransferase 3 // 10q22.1	-2,03	LUMINAL A down vs CONTROL
UBL5	NM_024292 // UBL5 // ubiquitin-like 5 // 19p13.3 // 59286 /// NM_001048241 // UB	-2,03	LUMINAL A down vs CONTROL
TLE1	NM_005077 // TLE1 // transducin-like enhancer of split 1 (E(sp1) homolog, Drosop	-2,03	LUMINAL A down vs CONTROL
MXRA7	NM_001008528 // MXRA7 // matrix-remodelling associated 7 // 17q25.1 // 439921 //	-2,03	LUMINAL A down vs CONTROL
LYNX1	NM_177457 // LYNX1 // Ly6/neurotoxin 1 // 8q24.3 // 66004 /// NM_177477 // LYNX1	-2,03	LUMINAL A down vs CONTROL
PDLIM4	NM_003687 // PDLIM4 // PDZ and LIM domain 4 // 5q31.1 // 8572 /// NM_001131027 /	-2,03	LUMINAL A down vs CONTROL
L3MBTL	NM_032107 // L3MBTL // l(3)mbt-like (Drosophila) // 20q13.12 // 26013 /// NM_015	-2,03	LUMINAL A down vs CONTROL
LALBA	NM_002289 // LALBA // lactalbumin, alpha- // 12q13 // 3906 /// ENST00000301046 /	-2,03	LUMINAL A down vs CONTROL
OSBPL5	NM_020896 // OSBPL5 // oxysterol binding protein-like 5 // 11p15.4 // 114879 ///	-2,03	LUMINAL A down vs CONTROL
BHLHA15	NM_177455 // BHLHA15 // basic helix-loop-helix family, member a15 // 7q21.3 // 1	-2,04	LUMINAL A down vs CONTROL
SOX18	NM_018419 // SOX18 // SRY (sex determining region Y)-box 18 // 20q13.33 // 54345	-2,04	LUMINAL A down vs CONTROL
BPIL1	NM_025227 // BPIL1 // bactericidal/permeability-increasing protein-like 1 // 20q	-2,04	LUMINAL A down vs CONTROL
DVL1	NM_004421 // DVL1 // dishevelled, dsh homolog 1 (Drosophila) // 1p36 // 1855 ///	-2,04	LUMINAL A down vs CONTROL
PEG3	NM_006210 // PEG3 // paternally expressed 3 // 19q13.4 // 5178 /// NM_001146184	-2,04	LUMINAL A down vs CONTROL
PLA2G6	NM_003560 // PLA2G6 // phospholipase A2, group VI (cytosolic, calcium-independen	-2,04	LUMINAL A down vs CONTROL
THRA	NM_003250 // THRA // thyroid hormone receptor, alpha (erythroblastic leukemia vi	-2,04	LUMINAL A down vs CONTROL
RPS25	NM_001028 // RPS25 // ribosomal protein S25 // 11q23.3 // 6230 /// ENST000002369	-2,04	LUMINAL A down vs CONTROL
OR10A2	NM_001004460 // OR10A2 // olfactory receptor, family 10, subfamily A, member 2 /	-2,04	LUMINAL A down vs CONTROL

SHB	NM_003028 // SHB // Src homology 2 domain containing adaptor protein B // 9p13.2	-2,05	LUMINAL A down vs CONTROL
TRIOBP	NM_001039141 // TRIOBP // TRIO and F-actin binding protein // 22q13.1 // 11078 /	-2,05	LUMINAL A down vs CONTROL
ZNF771	NM_016643 // ZNF771 // zinc finger protein 771 // 16p11.2 // 51333 /// NM_001142	-2,05	LUMINAL A down vs CONTROL
LRP1	NM_002332 // LRP1 // low density lipoprotein receptor-related protein 1 // 12q13	-2,05	LUMINAL A down vs CONTROL
DUS1L	NM_022156 // DUS1L // dihydrouridine synthase 1-like (S. cerevisiae) // 17q25.3	-2,05	LUMINAL A down vs CONTROL
SLC14A2	NM_007163 // SLC14A2 // solute carrier family 14 (urea transporter), member 2 //	-2,05	LUMINAL A down vs CONTROL
LCAT	NM_000229 // LCAT // lecithin-cholesterol acyltransferase // 16q22.1 // 3931 ///	-2,05	LUMINAL A down vs CONTROL
RNASE9	NM_001110359 // RNASE9 // ribonuclease, RNase A family, 9 (non-active) // 14q11.	-2,05	LUMINAL A down vs CONTROL
GAS6	NM_000820 // GAS6 // growth arrest-specific 6 // 13q34 // 2621 /// NM_001143946	-2,05	LUMINAL A down vs CONTROL
RERE	NM_012102 // RERE // arginine-glutamic acid dipeptide (RE) repeats // 1p36.2-p36	-2,05	LUMINAL A down vs CONTROL
AMOTL1	NM_130847 // AMOTL1 // angiomotin like 1 // 11q14.3 // 154810 /// ENST0000043306	-2,05	LUMINAL A down vs CONTROL
CCDC40	NM_017950 // CCDC40 // coiled-coil domain containing 40 // 17q25.3 // 55036 ///	-2,05	LUMINAL A down vs CONTROL
CCBE1	NM_133459 // CCBE1 // collagen and calcium binding EGF domains 1 // 18q21.32 //	-2,05	LUMINAL A down vs CONTROL
MAP1LC3A	NM_032514 // MAP1LC3A // microtubule-associated protein 1 light chain 3 alpha //	-2,05	LUMINAL A down vs CONTROL
RPSAP15	AF284768 // RPSAP15 // ribosomal protein SA pseudogene 15 // Xq21.31 // 220885 /	-2,05	LUMINAL A down vs CONTROL
USP15	NM_006313 // USP15 // ubiquitin specific peptidase 15 // 12q14 // 9958 /// ENST0	-2,05	LUMINAL A down vs CONTROL
FSTL3	NM_005860 // FSTL3 // follistatin-like 3 (secreted glycoprotein) // 19p13 // 102	-2,05	LUMINAL A down vs CONTROL
CDKN1A	NM_078467 // CDKN1A // cyclin-dependent kinase inhibitor 1A (p21, Cip1) // 6p21.	-2,05	LUMINAL A down vs CONTROL
TFCP2L1	NM_014553 // TFCP2L1 // transcription factor CP2-like 1 // 2q14 // 29842 /// ENS	-2,05	LUMINAL A down vs CONTROL
ZNF331	NM_018555 // ZNF331 // zinc finger protein 331 // 19q13.42 // 55422 /// NM_00107	-2,05	LUMINAL A down vs CONTROL
CSNK1G2	NM_001319 // CSNK1G2 // casein kinase 1, gamma 2 // 19p13.3 // 1455 /// ENST0000	-2,05	LUMINAL A down vs CONTROL
HOXA2	NM_006735 // HOXA2 // homeobox A2 // 7p15-p14 // 3199 /// ENST00000222718 // HOX	-2,06	LUMINAL A down vs CONTROL
AHNAK	NM_001620 // AHNAK // AHNAK nucleoprotein // 11q12.2 // 79026 /// NM_024060 // A	-2,06	LUMINAL A down vs CONTROL
MTRF1L	NM_019041 // MTRF1L // mitochondrial translational release factor 1-like // 6q25	-2,06	LUMINAL A down vs CONTROL
IGFBP6	NM_002178 // IGFBP6 // insulin-like growth factor binding protein 6 // 12q13 //	-2,06	LUMINAL A down vs CONTROL
CHERP	NM_006387 // CHERP // calcium homeostasis endoplasmic reticulum protein // 19p13	-2,06	LUMINAL A down vs CONTROL
LHCGR	NM_000233 // LHCGR // luteinizing hormone/choriogonadotropin receptor // 2p21 //	-2,06	LUMINAL A down vs CONTROL
KLF9	NM_001206 // KLF9 // Kruppel-like factor 9 // 9q13 // 687 /// ENST00000377126 //	-2,06	LUMINAL A down vs CONTROL
C17orf103	NM_152914 // C17orf103 // chromosome 17 open reading frame 103 // 17p11.2 // 256	-2,06	LUMINAL A down vs CONTROL
LGI4	NM_139284 // LGI4 // leucine-rich repeat LGI family, member 4 // 19q13.12|19q13.	-2,06	LUMINAL A down vs CONTROL
FBXO31	NM_024735 // FBXO31 // F-box protein 31 // 16q24.2 // 79791 /// NR_024568 // FBX	-2,06	LUMINAL A down vs CONTROL
COL4A1	NM_001845 // COL4A1 // collagen, type IV, alpha 1 // 13q34 // 1282 /// ENST00000	-2,06	LUMINAL A down vs CONTROL
NOTCH4	NM_004557 // NOTCH4 // Notch homolog 4 (Drosophila) // 6p21.3 // 4855 /// ENST00	-2,07	LUMINAL A down vs CONTROL

CDKN1C	NM_000076 // CDKN1C // cyclin-dependent kinase inhibitor 1C (p57, Kip2) // 11p15	-2,07	LUMINAL A down vs CONTROL
CHMP4B	NM_176812 // CHMP4B // chromatin modifying protein 4B // 20q11.22 // 128866 ///	-2,07	LUMINAL A down vs CONTROL
TMEM100	NM_001099640 // TMEM100 // transmembrane protein 100 // 17q22 // 55273 /// NM_01	-2,07	LUMINAL A down vs CONTROL
ERVWE1	NM_001130925 // ERVWE1 // endogenous retroviral family W, env(C7), member 1 // 7	-2,07	LUMINAL A down vs CONTROL
HRASLS2	NM_017878 // HRASLS2 // HRAS-like suppressor 2 // 11q12.3 // 54979 /// ENST00000	-2,07	LUMINAL A down vs CONTROL
NFASC	NM_001005388 // NFASC // neurofascin homolog (chicken) // 1q32.1 // 23114 /// NM	-2,07	LUMINAL A down vs CONTROL
MYO15B	NR_003587 // MYO15B // myosin XVB pseudogene // 17q25.1 // 80022 /// AF418286 //	-2,07	LUMINAL A down vs CONTROL
IFNA7	NM_021057 // IFNA7 // interferon, alpha 7 // 9p22 // 3444 /// NM_002172 // IFNA1	-2,07	LUMINAL A down vs CONTROL
SOX10	NM_006941 // SOX10 // SRY (sex determining region Y)-box 10 // 22q13.1 // 6663 /	-2,07	LUMINAL A down vs CONTROL
GALNTL6	NM_001034845 // GALNTL6 // UDP-N-acetyl-alpha-D-galactosamine:polypeptide N-acet	-2,07	LUMINAL A down vs CONTROL
CXCL1	NM_001511 // CXCL1 // chemokine (C-X-C motif) ligand 1 (melanoma growth stimulat	-2,07	LUMINAL A down vs CONTROL
TUBB2B	NM_178012 // TUBB2B // tubulin, beta 2B // 6p25 // 347733 /// ENST00000259818 //	-2,08	LUMINAL A down vs CONTROL
GPR109A	NM_177551 // GPR109A // G protein-coupled receptor 109A // 12q24.31 // 338442 //	-2,08	LUMINAL A down vs CONTROL
AVPI1	NM_021732 // AVPI1 // arginine vasopressin-induced 1 // 10q24.2 // 60370 /// ENS	-2,08	LUMINAL A down vs CONTROL
TSSK2	NM_053006 // TSSK2 // testis-specific serine kinase 2 // 22q11.21 // 23617 /// E	-2,08	LUMINAL A down vs CONTROL
CX3CL1	NM_002996 // CX3CL1 // chemokine (C-X3-C motif) ligand 1 // 16q13 // 6376 /// EN	-2,08	LUMINAL A down vs CONTROL
ZC3H18	NM_144604 // ZC3H18 // zinc finger CCCH-type containing 18 // 16q24.2 // 124245	-2,08	LUMINAL A down vs CONTROL
GRASP	NM_181711 // GRASP // GRP1 (general receptor for phosphoinositides 1)-associated	-2,08	LUMINAL A down vs CONTROL
TKT	NM_001135055 // TKT // transketolase // 3p14.3 // 7086 /// NM_001064 // TKT // t	-2,08	LUMINAL A down vs CONTROL
HPD	NM_001171993 // HPD // 4-hydroxyphenylpyruvate dioxygenase // 12q24-qter // 3242	-2,08	LUMINAL A down vs CONTROL
HAAO	NM_012205 // HAAO // 3-hydroxyanthranilate 3,4-dioxygenase // 2p21 // 23498 ///	-2,08	LUMINAL A down vs CONTROL
NFIL3	NM_005384 // NFIL3 // nuclear factor, interleukin 3 regulated // 9q22 // 4783 //	-2,09	LUMINAL A down vs CONTROL
ZNF618	NM_133374 // ZNF618 // zinc finger protein 618 // 9q32 // 114991 /// ENST0000028	-2,09	LUMINAL A down vs CONTROL
ADAM33	NM_025220 // ADAM33 // ADAM metallopeptidase domain 33 // 20p13 // 80332 /// NM_	-2,09	LUMINAL A down vs CONTROL
TPPP	NM_007030 // TPPP // tubulin polymerization promoting protein // 5p15.3 // 11076	-2,09	LUMINAL A down vs CONTROL
ZC3H7B	NM_017590 // ZC3H7B // zinc finger CCCH-type containing 7B // 22q13.2 // 23264 /	-2,09	LUMINAL A down vs CONTROL
PLCD3	NM_133373 // PLCD3 // phospholipase C, delta 3 // 17q21.31 // 113026 /// ENST000	-2,09	LUMINAL A down vs CONTROL
PRDM9	NM_020227 // PRDM9 // PR domain containing 9 // 5p14 // 56979 /// NM_001098173 /	-2,09	LUMINAL A down vs CONTROL
EML3	NM_153265 // EML3 // echinoderm microtubule associated protein like 3 // 11q12.3	-2,09	LUMINAL A down vs CONTROL
KCNE2	NM_172201 // KCNE2 // potassium voltage-gated channel, Isk-related family, membe	-2,09	LUMINAL A down vs CONTROL
GPLD1	NM_001503 // GPLD1 // glycosylphosphatidylinositol specific phospholipase D1 //	-2,09	LUMINAL A down vs CONTROL
SYN2	NM_133625 // SYN2 // synapsin II // 3p25 // 6854 /// NM_003178 // SYN2 // synaps	-2,09	LUMINAL A down vs CONTROL
ATP1A2	NM_000702 // ATP1A2 // ATPase, Na+/K+ transporting, alpha 2 polypeptide // 1q21-	-2,09	LUMINAL A down vs CONTROL

PGM1	NM_002633 // PGM1 // phosphoglucomutase 1 // 1p31 // 5236 /// NM_001172818 // PG	-2,09	LUMINAL A down vs CONTROL
NUDT8	NM_181843 // NUDT8 // nudix (nucleoside diphosphate linked moiety X)-type motif	-2,09	LUMINAL A down vs CONTROL
PDE11A	NM_001077197 // PDE11A // phosphodiesterase 11A // 2q31.2 // 50940 /// NM_016953	-2,09	LUMINAL A down vs CONTROL
OCM2	NM_006188 // OCM2 // oncomodulin 2 // 7q21.2 // 4951 /// NM_001097622 // OCM //	-2,09	LUMINAL A down vs CONTROL
MPV17L2	NM_032683 // MPV17L2 // MPV17 mitochondrial membrane protein-like 2 // 19p13.11	-2,10	LUMINAL A down vs CONTROL
ANAPC2	NM_013366 // ANAPC2 // anaphase promoting complex subunit 2 // 9q34.3 // 29882 /	-2,10	LUMINAL A down vs CONTROL
PER1	NM_002616 // PER1 // period homolog 1 (Drosophila) // 17p13.1-p12 // 5187 /// EN	-2,10	LUMINAL A down vs CONTROL
AFAP1L1	NM_152406 // AFAP1L1 // actin filament associated protein 1-like 1 // 5q32 // 13	-2,10	LUMINAL A down vs CONTROL
NAV2	NM_182964 // NAV2 // neuron navigator 2 // 11p15.1 // 89797 /// NM_145117 // NAV	-2,10	LUMINAL A down vs CONTROL
FGFR1	NM_001174064 // FGFR1 // fibroblast growth factor receptor 1 // 8p11.2-p11.1 //	-2,10	LUMINAL A down vs CONTROL
ULBP1	NM_025218 // ULBP1 // UL16 binding protein 1 // 6q25 // 80329 /// ENST0000022970	-2,10	LUMINAL A down vs CONTROL
LBP	NM_004139 // LBP // lipopolysaccharide binding protein // 20q11.23-q12 // 3929 /	-2,10	LUMINAL A down vs CONTROL
FLJ10357	NM_018071 // FLJ10357 // protein SOLO // 14q11.2 // 55701 /// ENST00000298694 //	-2,10	LUMINAL A down vs CONTROL
CLIC5	NM_001114086 // CLIC5 // chloride intracellular channel 5 // 6p21.1-p12.1 // 534	-2,10	LUMINAL A down vs CONTROL
ARHGDIA	NM_004309 // ARHGDIA // Rho GDP dissociation inhibitor (GDI) alpha // 17q25.3 //	-2,11	LUMINAL A down vs CONTROL
COX7A1	NM_001864 // COX7A1 // cytochrome c oxidase subunit VIIa polypeptide 1 (muscle)	-2,11	LUMINAL A down vs CONTROL
TCF25	NM_014972 // TCF25 // transcription factor 25 (basic helix-loop-helix) // 16q24.	-2,11	LUMINAL A down vs CONTROL
BHMT2	NM_017614 // BHMT2 // betaine--homocysteine S-methyltransferase 2 // 5q13 // 237	-2,11	LUMINAL A down vs CONTROL
FOXO4	NM_005938 // FOXO4 // forkhead box O4 // Xq13.1 // 4303 /// NM_001170931 // FOXO	-2,12	LUMINAL A down vs CONTROL
TUBB6	NM_032525 // TUBB6 // tubulin, beta 6 // 18p11.21 // 84617 /// ENST00000317702 /	-2,12	LUMINAL A down vs CONTROL
CSRNP1	NM_033027 // CSRNP1 // cysteine-serine-rich nuclear protein 1 // 3p22 // 64651 /	-2,12	LUMINAL A down vs CONTROL
REEP2	NM_016606 // REEP2 // receptor accessory protein 2 // 5q31 // 51308 /// ENST0000	-2,12	LUMINAL A down vs CONTROL
IGF2BP2	NM_006548 // IGF2BP2 // insulin-like growth factor 2 mRNA binding protein 2 // 3	-2,12	LUMINAL A down vs CONTROL
SGSM2	NM_014853 // SGSM2 // small G protein signaling modulator 2 // 17p13.3 // 9905 /	-2,12	LUMINAL A down vs CONTROL
AIF1L	NM_031426 // AIF1L // allograft inflammatory factor 1-like // 9q34.13-q34.3 // 8	-2,12	LUMINAL A down vs CONTROL
MAP3K11	NM_002419 // MAP3K11 // mitogen-activated protein kinase kinase kinase 11 // 11q	-2,12	LUMINAL A down vs CONTROL
NMNAT2	NM_015039 // NMNAT2 // nicotinamide nucleotide adenylyltransferase 2 // 1q25 //	-2,12	LUMINAL A down vs CONTROL
LTBP3	NM_001130144 // LTBP3 // latent transforming growth factor beta binding protein	-2,12	LUMINAL A down vs CONTROL
INF2	NM_022489 // INF2 // inverted formin, FH2 and WH2 domain containing // 14q32.33	-2,12	LUMINAL A down vs CONTROL
HEBP2	NM_014320 // HEBP2 // heme binding protein 2 // 6q24 // 23593 /// ENST0000005869	-2,12	LUMINAL A down vs CONTROL
PEBP4	NM_144962 // PEBP4 // phosphatidylethanolamine-binding protein 4 // 8p21.3 // 15	-2,12	LUMINAL A down vs CONTROL
CCDC92	NM_025140 // CCDC92 // coiled-coil domain containing 92 // 12q24.31 // 80212 ///	-2,13	LUMINAL A down vs CONTROL
KLHL21	NM_014851 // KLHL21 // kelch-like 21 (Drosophila) // 1p36.31 // 9903 /// ENST000	-2,13	LUMINAL A down vs CONTROL

TMOD1	NM_003275 // TMOD1 // tropomodulin 1 // 9q22.3 // 7111 /// NM_001166116 // TMOD1	-2,13	LUMINAL A down vs CONTROL
CRABP1	NM_004378 // CRABP1 // cellular retinoic acid binding protein 1 // 15q24 // 1381	-2,13	LUMINAL A down vs CONTROL
FLJ16171	AK131247 // FLJ16171 // FLJ16171 protein // 5q35.2 // 441116	-2,13	LUMINAL A down vs CONTROL
WNK2	NM_006648 // WNK2 // WNK lysine deficient protein kinase 2 // 9q22.3 // 65268 //	-2,13	LUMINAL A down vs CONTROL
DOT1L	NM_032482 // DOT1L // DOT1-like, histone H3 methyltransferase (S. cerevisiae) //	-2,13	LUMINAL A down vs CONTROL
TAC4	NM_170685 // TAC4 // tachykinin 4 (hemokinin) // 17q21.33 // 255061 /// NM_00107	-2,13	LUMINAL A down vs CONTROL
RIC3	NM_024557 // RIC3 // resistance to inhibitors of cholinesterase 3 homolog (C. el	-2,13	LUMINAL A down vs CONTROL
VWA1	NM_022834 // VWA1 // von Willebrand factor A domain containing 1 // 1p36.33 // 6	-2,13	LUMINAL A down vs CONTROL
IER2	NM_004907 // IER2 // immediate early response 2 // 19p13.2 // 9592 /// ENST00000	-2,13	LUMINAL A down vs CONTROL
NOVA2	NM_002516 // NOVA2 // neuro-oncological ventral antigen 2 // 19q13.3 // 4858 ///	-2,13	LUMINAL A down vs CONTROL
GPIHBP1	NM_178172 // GPIHBP1 // glycosylphosphatidylinositol anchored high density lipop	-2,13	LUMINAL A down vs CONTROL
DTX2	NM_020892 // DTX2 // deltex homolog 2 (Drosophila) // 7q11.23 // 113878 /// NM_0	-2,13	LUMINAL A down vs CONTROL
HSPA12A	NM_025015 // HSPA12A // heat shock 70kDa protein 12A // 10q26.12 // 259217 /// E	-2,13	LUMINAL A down vs CONTROL
LYL1	NM_005583 // LYL1 // lymphoblastic leukemia derived sequence 1 // 19p13.2 // 406	-2,13	LUMINAL A down vs CONTROL
KRT14	NM_000526 // KRT14 // keratin 14 // 17q12-q21 // 3861 /// ENST00000167586 // KRT	-2,13	LUMINAL A down vs CONTROL
MMRN2	NM_024756 // MMRN2 // multimerin 2 // 10q23.2 // 79812 /// ENST00000372027 // MM	-2,13	LUMINAL A down vs CONTROL
CCIN	NM_005893 // CCIN // calicin // 9p13.3 // 881 /// ENST00000335119 // CCIN // cal	-2,14	LUMINAL A down vs CONTROL
RPL23AP64	NR_003040 // RPL23AP64 // ribosomal protein L23a pseudogene 64 // 11q23.3 // 649	-2,14	LUMINAL A down vs CONTROL
LARP6	NM_018357 // LARP6 // La ribonucleoprotein domain family, member 6 // 15q23 // 5	-2,14	LUMINAL A down vs CONTROL
KLF2	NM_016270 // KLF2 // Kruppel-like factor 2 (lung) // 19p13.13-p13.11 // 10365 //	-2,14	LUMINAL A down vs CONTROL
MTSS1L	NM_138383 // MTSS1L // metastasis suppressor 1-like // 16q22.1 // 92154 /// ENST	-2,14	LUMINAL A down vs CONTROL
GDPD5	NM_030792 // GDPD5 // glycerophosphodiester phosphodiesterase domain containing	-2,14	LUMINAL A down vs CONTROL
GABRE	NM_004961 // GABRE // gamma-aminobutyric acid (GABA) A receptor, epsilon // Xq28	-2,14	LUMINAL A down vs CONTROL
LY6G5B	NM_021221 // LY6G5B // lymphocyte antigen 6 complex, locus G5B // 6p21.3 // 5849	-2,14	LUMINAL A down vs CONTROL
GAB2	NM_080491 // GAB2 // GRB2-associated binding protein 2 // 11q14.1 // 9846 /// NM	-2,14	LUMINAL A down vs CONTROL
MCC	NM_001085377 // MCC // mutated in colorectal cancers // 5q21 // 4163 /// NM_0023	-2,14	LUMINAL A down vs CONTROL
APLNR	NM_005161 // APLNR // apelin receptor // 11q12 // 187 /// NR_027991 // APLNR //	-2,15	LUMINAL A down vs CONTROL
SERP2	NM_001010897 // SERP2 // stress-associated endoplasmic reticulum protein family	-2,15	LUMINAL A down vs CONTROL
PKN3	NM_013355 // PKN3 // protein kinase N3 // 9q34.11 // 29941 /// ENST00000291906 /	-2,15	LUMINAL A down vs CONTROL
CDK10	NM_052988 // CDK10 // cyclin-dependent kinase 10 // 16q24 // 8558 /// NM_0010985	-2,15	LUMINAL A down vs CONTROL
SLC16A2	NM_006517 // SLC16A2 // solute carrier family 16, member 2 (monocarboxylic acid	-2,15	LUMINAL A down vs CONTROL
MAP3K6	NM_004672 // MAP3K6 // mitogen-activated protein kinase kinase kinase 6 // 1p36.	-2,15	LUMINAL A down vs CONTROL
SLC6A8	NM_005629 // SLC6A8 // solute carrier family 6 (neurotransmitter transporter, cr	-2,15	LUMINAL A down vs CONTROL

MIR1974	NR_031738 // MIR1974 // microRNA 1974 // 5q15 // 100302207 /// NR_031741 // MIR1	-2,15	LUMINAL A down vs CONTROL
GPX3	NM_002084 // GPX3 // glutathione peroxidase 3 (plasma) // 5q23 // 2878 /// ENST0	-2,16	LUMINAL A down vs CONTROL
CALCA	NM_001741 // CALCA // calcitonin-related polypeptide alpha // 11p15.2-p15.1 // 7	-2,16	LUMINAL A down vs CONTROL
HBQ1	NM_005331 // HBQ1 // hemoglobin, theta 1 // 16p13.3 // 3049 /// ENST00000199708	-2,16	LUMINAL A down vs CONTROL
YBX2	NM_015982 // YBX2 // Y box binding protein 2 // 17p11.2-p13.1 // 51087 /// ENST0	-2,16	LUMINAL A down vs CONTROL
POR	NM_000941 // POR // P450 (cytochrome) oxidoreductase // 7q11.2 // 5447 /// ENST0	-2,16	LUMINAL A down vs CONTROL
DNM2	NM_001005360 // DNM2 // dynamin 2 // 19p13.2 // 1785 /// NM_001005361 // DNM2 //	-2,16	LUMINAL A down vs CONTROL
LMOD1	NM_012134 // LMOD1 // leiomodin 1 (smooth muscle) // 1q32 // 25802 /// ENST00000	-2,16	LUMINAL A down vs CONTROL
BIN1	NM_139343 // BIN1 // bridging integrator 1 // 2q14 // 274 /// NM_139344 // BIN1	-2,17	LUMINAL A down vs CONTROL
NPY2R	NM_000910 // NPY2R // neuropeptide Y receptor Y2 // 4q31 // 4887 /// ENST0000032	-2,17	LUMINAL A down vs CONTROL
KRTAP23-1	NM_181624 // KRTAP23-1 // keratin associated protein 23-1 // 21q22.1 // 337963 /	-2,17	LUMINAL A down vs CONTROL
CA4	NM_000717 // CA4 // carbonic anhydrase IV // 17q23 // 762 /// ENST00000300900 //	-2,17	LUMINAL A down vs CONTROL
PRELP	NM_002725 // PRELP // proline/arginine-rich end leucine-rich repeat protein // 1	-2,17	LUMINAL A down vs CONTROL
C16orf7	NM_004913 // C16orf7 // chromosome 16 open reading frame 7 // 16q24 // 9605 ///	-2,17	LUMINAL A down vs CONTROL
MYO1C	NM_001080779 // MYO1C // myosin IC // 17p13 // 4641 /// NM_001080950 // MYO1C //	-2,18	LUMINAL A down vs CONTROL
C1orf113	NM_001162530 // C1orf113 // chromosome 1 open reading frame 113 // 1p34.3 // 797	-2,18	LUMINAL A down vs CONTROL
TSHZ2	NM_173485 // TSHZ2 // teashirt zinc finger homeobox 2 // 20q13.2 // 128553 /// E	-2,18	LUMINAL A down vs CONTROL
RSPO3	NM_032784 // RSPO3 // R-spondin 3 homolog (Xenopus laevis) // 6q22.33 // 84870 /	-2,18	LUMINAL A down vs CONTROL
SAA4	NM_006512 // SAA4 // serum amyloid A4, constitutive // 11p15.1-p14 // 6291 /// E	-2,18	LUMINAL A down vs CONTROL
CABC1	NM_020247 // CABC1 // chaperone, ABC1 activity of bc1 complex homolog (S. pombe)	-2,18	LUMINAL A down vs CONTROL
CCDC124	NM_138442 // CCDC124 // coiled-coil domain containing 124 // 19p13.11 // 115098	-2,18	LUMINAL A down vs CONTROL
EEPD1	NM_030636 // EEPD1 // endonuclease/exonuclease/phosphatase family domain contain	-2,19	LUMINAL A down vs CONTROL
DAAM2	NM_015345 // DAAM2 // dishevelled associated activator of morphogenesis 2 // 6p2	-2,19	LUMINAL A down vs CONTROL
NTF4	NM_006179 // NTF4 // neurotrophin 4 // 19q13.3 // 4909 /// ENST00000301411 // NT	-2,19	LUMINAL A down vs CONTROL
PLXNA4	NM_020911 // PLXNA4 // plexin A4 // 7q32.3 // 91584 /// NM_181775 // PLXNA4 // p	-2,19	LUMINAL A down vs CONTROL
TUBG2	NM_016437 // TUBG2 // tubulin, gamma 2 // 17q21 // 27175 /// ENST00000251412 //	-2,19	LUMINAL A down vs CONTROL
CRIM1	NM_016441 // CRIM1 // cysteine rich transmembrane BMP regulator 1 (chordin-like)	-2,19	LUMINAL A down vs CONTROL
TMTC1	NM_175861 // TMTC1 // transmembrane and tetratricopeptide repeat containing 1 //	-2,19	LUMINAL A down vs CONTROL
MSRA	NM_012331 // MSRA // methionine sulfoxide reductase A // 8p23.1 // 4482 /// NM_0	-2,19	LUMINAL A down vs CONTROL
TMC7	NM_024847 // TMC7 // transmembrane channel-like 7 // 16p12.3 // 79905 /// NM_001	-2,20	LUMINAL A down vs CONTROL
SPRR2A	NM_005988 // SPRR2A // small proline-rich protein 2A // 1q21-q22 // 6700 /// NM_	-2,20	LUMINAL A down vs CONTROL
KIR3DL1	NM_013289 // KIR3DL1 // killer cell immunoglobulin-like receptor, three domains,	-2,20	LUMINAL A down vs CONTROL
C20orf112	NM_080616 // C20orf112 // chromosome 20 open reading frame 112 // 20q11.1-q11.23	-2,20	LUMINAL A down vs CONTROL

KRTAP4-2	NM_033062 // KRTAP4-2 // keratin associated protein 4-2 // 17q12-q21 // 85291 //	-2,21	LUMINAL A down vs CONTROL
MRAP	ENST00000303645 // MRAP // melanocortin 2 receptor accessory protein // 21q22.1	-2,21	LUMINAL A down vs CONTROL
ACADVL	NM_000018 // ACADVL // acyl-CoA dehydrogenase, very long chain // 17p13-p11 // 3	-2,22	LUMINAL A down vs CONTROL
HLF	NM_002126 // HLF // hepatic leukemia factor // 17q22 // 3131 /// ENST00000226067	-2,22	LUMINAL A down vs CONTROL
MT1G	NM_005950 // MT1G // metallothionein 1G // 16q13 // 4495 /// BC020757 // MT1G //	-2,22	LUMINAL A down vs CONTROL
ENSA	NM_207044 // ENSA // endosulfine alpha // 1q21.3 // 2029 /// NM_207047 // ENSA /	-2,22	LUMINAL A down vs CONTROL
C15orf5	NR_026813 // C15orf5 // chromosome 15 open reading frame 5 // 15q23-q24 // 81698	-2,23	LUMINAL A down vs CONTROL
SNRNP70	NM_003089 // SNRNP70 // small nuclear ribonucleoprotein 70kDa (U1) // 19q13.3 //	-2,23	LUMINAL A down vs CONTROL
PSMD4	NM_002810 // PSMD4 // proteasome (prosome, macropain) 26S subunit, non-ATPase, 4	-2,23	LUMINAL A down vs CONTROL
HAND2	NM_021973 // HAND2 // heart and neural crest derivatives expressed 2 // 4q33 //	-2,23	LUMINAL A down vs CONTROL
IRS2	NM_003749 // IRS2 // insulin receptor substrate 2 // 13q34 // 8660 /// ENST00000	-2,23	LUMINAL A down vs CONTROL
CRHBP	NM_001882 // CRHBP // corticotropin releasing hormone binding protein // 5q11.2-	-2,23	LUMINAL A down vs CONTROL
CYB5R3	NM_001129819 // CYB5R3 // cytochrome b5 reductase 3 // 22q13.31-qter|22q13.2-q13	-2,23	LUMINAL A down vs CONTROL
GPC3	NM_001164617 // GPC3 // glypican 3 // Xq26.1 // 2719 /// NM_004484 // GPC3 // gl	-2,23	LUMINAL A down vs CONTROL
PPP2R1B	NM_002716 // PPP2R1B // protein phosphatase 2, regulatory subunit A, beta // 11q	-2,23	LUMINAL A down vs CONTROL
YBX1	NM_004559 // YBX1 // Y box binding protein 1 // 1p34 // 4904 /// ENST00000321358	-2,23	LUMINAL A down vs CONTROL
ANKRD29	NM_173505 // ANKRD29 // ankyrin repeat domain 29 // 18q11.2 // 147463 /// ENST00	-2,23	LUMINAL A down vs CONTROL
LOC147804	CR610971 // LOC147804 // tropomyosin 3 pseudogene // 19q13.42 // 147804	-2,23	LUMINAL A down vs CONTROL
NNAT	NM_005386 // NNAT // neuronatin // 20q11.2-q12 // 4826 /// NM_181689 // NNAT //	-2,23	LUMINAL A down vs CONTROL
ANKRD11	NM_013275 // ANKRD11 // ankyrin repeat domain 11 // 16q24.3 // 29123 /// ENST000	-2,23	LUMINAL A down vs CONTROL
NCOR2	NM_006312 // NCOR2 // nuclear receptor co-repressor 2 // 12q24 // 9612 /// NM_00	-2,24	LUMINAL A down vs CONTROL
GPX4	NM_002085 // GPX4 // glutathione peroxidase 4 (phospholipid hydroperoxidase) //	-2,24	LUMINAL A down vs CONTROL
C17orf88	NR_026770 // C17orf88 // chromosome 17 open reading frame 88 // 17q21.31 // 2359	-2,24	LUMINAL A down vs CONTROL
GSTT1	NM_000853 // GSTT1 // glutathione S-transferase theta 1 // 22q11.23 // 2952 ///	-2,24	LUMINAL A down vs CONTROL
NTRK2	NM_006180 // NTRK2 // neurotrophic tyrosine kinase, receptor, type 2 // 9q22.1 /	-2,24	LUMINAL A down vs CONTROL
HSD17B13	NM_178135 // HSD17B13 // hydroxysteroid (17-beta) dehydrogenase 13 // 4q22.1 //	-2,24	LUMINAL A down vs CONTROL
KIF1C	NM_006612 // KIF1C // kinesin family member 1C // 17p13.2 // 10749 /// ENST00000	-2,24	LUMINAL A down vs CONTROL
LIMS2	NM_001136037 // LIMS2 // LIM and senescent cell antigen-like domains 2 // 2q14.3	-2,25	LUMINAL A down vs CONTROL
DPT	NM_001937 // DPT // dermatopontin // 1q12-q23 // 1805 /// ENST00000367817 // DPT	-2,25	LUMINAL A down vs CONTROL
TRY6	NR_001296 // TRY6 // trypsinogen C // 7q34 // 154754 /// AY052784 // PRSS2 // pr	-2,25	LUMINAL A down vs CONTROL
CKB	NM_001823 // CKB // creatine kinase, brain // 14q32 // 1152 /// ENST00000348956	-2,25	LUMINAL A down vs CONTROL
FXYD1	NM_005031 // FXYD1 // FXYD domain containing ion transport regulator 1 // 19q13.	-2,25	LUMINAL A down vs CONTROL
MKNK2	NM_199054 // MKNK2 // MAP kinase interacting serine/threonine kinase 2 // 19p13.	-2,25	LUMINAL A down vs CONTROL

GPR4	NM_005282 // GPR4 // G protein-coupled receptor 4 // 19q13.3 // 2828 /// ENST000	-2,26	LUMINAL A down vs CONTROL
FAM13A	NM_001015045 // FAM13A // family with sequence similarity 13, member A // 4q22.1	-2,26	LUMINAL A down vs CONTROL
OR1S1	NM_001004458 // OR1S1 // olfactory receptor, family 1, subfamily S, member 1 //	-2,26	LUMINAL A down vs CONTROL
CEND1	NM_016564 // CEND1 // cell cycle exit and neuronal differentiation 1 // 11p15.5	-2,26	LUMINAL A down vs CONTROL
PLEKHG5	NM_198681 // PLEKHG5 // pleckstrin homology domain containing, family G (with Rh	-2,26	LUMINAL A down vs CONTROL
KLF15	NM_014079 // KLF15 // Kruppel-like factor 15 // 3q13-q21 // 28999 /// ENST000002	-2,26	LUMINAL A down vs CONTROL
EFNB1	NM_004429 // EFNB1 // ephrin-B1 // Xq12 // 1947 /// ENST00000204961 // EFNB1 //	-2,26	LUMINAL A down vs CONTROL
HSPG2	NM_005529 // HSPG2 // heparan sulfate proteoglycan 2 // 1p36.1-p34 // 3339 /// N	-2,27	LUMINAL A down vs CONTROL
RPS6KA2	NM_021135 // RPS6KA2 // ribosomal protein S6 kinase, 90kDa, polypeptide 2 // 6q2	-2,27	LUMINAL A down vs CONTROL
MYOM1	NM_003803 // MYOM1 // myomesin 1, 185kDa // 18p11.31 // 8736 /// NM_019856 // MY	-2,27	LUMINAL A down vs CONTROL
C1orf115	NM_024709 // C1orf115 // chromosome 1 open reading frame 115 // 1q41 // 79762 //	-2,27	LUMINAL A down vs CONTROL
REXO1L1	NM_172239 // REXO1L1 // REX1, RNA exonuclease 1 homolog (S. cerevisiae)-like 1 /	-2,27	LUMINAL A down vs CONTROL
HOMER2	NM_199330 // HOMER2 // homer homolog 2 (Drosophila) // 15q24.3 // 9455 /// NM_00	-2,27	LUMINAL A down vs CONTROL
RETSAT	NM_017750 // RETSAT // retinol saturase (all-trans-retinol 13,14-reductase) // 2	-2,27	LUMINAL A down vs CONTROL
ACSS2	NM_001076552 // ACSS2 // acyl-CoA synthetase short-chain family member 2 // 20q1	-2,27	LUMINAL A down vs CONTROL
PER2	NM_022817 // PER2 // period homolog 2 (Drosophila) // 2q37.3 // 8864 /// ENST000	-2,27	LUMINAL A down vs CONTROL
TSPYL2	NM_022117 // TSPYL2 // TSPY-like 2 // Xp11.2 // 64061 /// NM_018969 // GPR173 //	-2,28	LUMINAL A down vs CONTROL
GNAL	NM_182978 // GNAL // guanine nucleotide binding protein (G protein), alpha activ	-2,28	LUMINAL A down vs CONTROL
SPANXE	NM_145665 // SPANXE // SPANX family, member E // Xq27.1 // 171489 /// NM_032417	-2,29	LUMINAL A down vs CONTROL
JUNB	NM_002229 // JUNB // jun B proto-oncogene // 19p13.2 // 3726 /// ENST00000302754	-2,29	LUMINAL A down vs CONTROL
MT1X	NM_005952 // MT1X // metallothionein 1X // 16q13 // 4501 /// ENST00000394485 //	-2,30	LUMINAL A down vs CONTROL
TOMM40	NM_001128917 // TOMM40 // translocase of outer mitochondrial membrane 40 homolog	-2,30	LUMINAL A down vs CONTROL
NMB	NM_021077 // NMB // neuromedin B // 15q22-qter // 4828 /// NM_205858 // NMB // n	-2,30	LUMINAL A down vs CONTROL
STXBP1	NM_003165 // STXBP1 // syntaxin binding protein 1 // 9q34.1 // 6812 /// NM_00103	-2,30	LUMINAL A down vs CONTROL
KANK1	NM_153186 // KANK1 // KN motif and ankyrin repeat domains 1 // 9p24.3 // 23189 /	-2,30	LUMINAL A down vs CONTROL
MAN2A2	NM_006122 // MAN2A2 // mannosidase, alpha, class 2A, member 2 // 15q26.1 // 4122	-2,30	LUMINAL A down vs CONTROL
CD300LG	NM_145273 // CD300LG // CD300 molecule-like family member g // 17q21.31 // 14689	-2,30	LUMINAL A down vs CONTROL
ABCC11	NM_033151 // ABCC11 // ATP-binding cassette, sub-family C (CFTR/MRP), member 11	-2,30	LUMINAL A down vs CONTROL
ATAD3C	NM_001039211 // ATAD3C // ATPase family, AAA domain containing 3C // 1p36.33 //	-2,30	LUMINAL A down vs CONTROL
PGAM2	NM_000290 // PGAM2 // phosphoglycerate mutase 2 (muscle) // 7p13-p12 // 5224 ///	-2,31	LUMINAL A down vs CONTROL
FKSG43	BC128150 // FKSG43 // FKSG43 gene // Xq12 // 83957	-2,31	LUMINAL A down vs CONTROL
DEFB129	NM_080831 // DEFB129 // defensin, beta 129 // 20p13 // 140881 /// ENST0000024610	-2,31	LUMINAL A down vs CONTROL
PPL	NM_002705 // PPL // periplakin // 16p13.3 // 5493 /// ENST00000345988 // PPL //	-2,31	LUMINAL A down vs CONTROL

SFRS16	NM_007056 // SFRS16 // splicing factor, arginine/serine-rich 16 // 19q13.3 // 11	-2,31	LUMINAL A down vs CONTROL
QTRT1	NM_031209 // QTRT1 // queuine tRNA-ribosyltransferase 1 // 19p13.3 // 81890 ///	-2,31	LUMINAL A down vs CONTROL
ACSL1	NM_001995 // ACSL1 // acyl-CoA synthetase long-chain family member 1 // 4q34-q35	-2,31	LUMINAL A down vs CONTROL
C5orf4	NM_032385 // C5orf4 // chromosome 5 open reading frame 4 // 5q31-q32 // 10826 //	-2,31	LUMINAL A down vs CONTROL
LCE2D	NM_178430 // LCE2D // late cornified envelope 2D // 1q21.3 // 353141 /// NM_1784	-2,31	LUMINAL A down vs CONTROL
SUN2	NM_015374 // SUN2 // Sad1 and UNC84 domain containing 2 // 22q13.1 // 25777 ///	-2,32	LUMINAL A down vs CONTROL
MUSTN1	NM_205853 // MUSTN1 // musculoskeletal, embryonic nuclear protein 1 // 3p21.1 //	-2,32	LUMINAL A down vs CONTROL
IFITM3	NM_021034 // IFITM3 // interferon induced transmembrane protein 3 (1-8U) // 11p1	-2,32	LUMINAL A down vs CONTROL
GAS8	NR_023348 // GAS8 // growth arrest-specific 8 // 16q24.3 // 2622 /// NM_001481 /	-2,32	LUMINAL A down vs CONTROL
PEMT	NM_148172 // PEMT // phosphatidylethanolamine N-methyltransferase // 17p11.2 //	-2,32	LUMINAL A down vs CONTROL
HSPA1B	NM_005346 // HSPA1B // heat shock 70kDa protein 1B // 6p21.3 // 3304 /// NM_0053	-2,33	LUMINAL A down vs CONTROL
CEBPD	NM_005195 // CEBPD // CCAAT/enhancer binding protein (C/EBP), delta // 8p11.2-p1	-2,33	LUMINAL A down vs CONTROL
GNA11	NM_002067 // GNA11 // guanine nucleotide binding protein (G protein), alpha 11 (	-2,33	LUMINAL A down vs CONTROL
KLK7	NM_139277 // KLK7 // kallikrein-related peptidase 7 // 19q13.41 // 5650 /// NM_0	-2,34	LUMINAL A down vs CONTROL
LAMB3	NM_001017402 // LAMB3 // laminin, beta 3 // 1q32 // 3914 /// NM_001127641 // LAM	-2,34	LUMINAL A down vs CONTROL
LOC92973	NR_024006 // LOC92973 // hypothetical LOC92973 // 9p13.3 // 92973	-2,34	LUMINAL A down vs CONTROL
LCE3A	NM_178431 // LCE3A // late cornified envelope 3A // 1q21.3 // 353142 /// ENST000	-2,34	LUMINAL A down vs CONTROL
SKI	NM_003036 // SKI // v-ski sarcoma viral oncogene homolog (avian) // 1q22-q24 //	-2,34	LUMINAL A down vs CONTROL
FAM107A	NM_007177 // FAM107A // family with sequence similarity 107, member A // 3p21.1	-2,35	LUMINAL A down vs CONTROL
FNDC4	NM_022823 // FNDC4 // fibronectin type III domain containing 4 // 2p23.3 // 6483	-2,35	LUMINAL A down vs CONTROL
ZNF385B	NM_152520 // ZNF385B // zinc finger protein 385B // 2q31.2-q31.3 // 151126 /// N	-2,35	LUMINAL A down vs CONTROL
ADAMTS15	NM_139055 // ADAMTS15 // ADAM metallopeptidase with thrombospondin type 1 motif,	-2,35	LUMINAL A down vs CONTROL
KLK11	NM_001167605 // KLK11 // kallikrein-related peptidase 11 // 19q13.3-q13.4 // 110	-2,35	LUMINAL A down vs CONTROL
CDH5	NM_001795 // CDH5 // cadherin 5, type 2 (vascular endothelium) // 16q22.1 // 100	-2,36	LUMINAL A down vs CONTROL
ECHDC2	NM_018281 // ECHDC2 // enoyl CoA hydratase domain containing 2 // 1p32.3 // 5526	-2,36	LUMINAL A down vs CONTROL
EHD2	NM_014601 // EHD2 // EH-domain containing 2 // 19q13.3 // 30846 /// ENST00000263	-2,36	LUMINAL A down vs CONTROL
ALOX15B	NM_001141 // ALOX15B // arachidonate 15-lipoxygenase, type B // 17p13.1 // 247 /	-2,36	LUMINAL A down vs CONTROL
PPP1R12C	NM_017607 // PPP1R12C // protein phosphatase 1, regulatory (inhibitor) subunit 1	-2,36	LUMINAL A down vs CONTROL
DDA1	NM_024050 // DDA1 // DET1 and DDB1 associated 1 // 19p13.11 // 79016 /// ENST000	-2,36	LUMINAL A down vs CONTROL
AMOTL2	NM_016201 // AMOTL2 // angiomotin like 2 // 3q21-q22 // 51421 /// ENST0000024988	-2,36	LUMINAL A down vs CONTROL
AK3L1	NM_001005353 // AK3L1 // adenylate kinase 3-like 1 // 1p31.3 // 205 /// NM_01341	-2,37	LUMINAL A down vs CONTROL
SAFB2	NM_014649 // SAFB2 // scaffold attachment factor B2 // 19p13.3 // 9667 /// ENST0	-2,37	LUMINAL A down vs CONTROL
CDK3	NM_001258 // CDK3 // cyclin-dependent kinase 3 // 17q22-qter // 1018 /// ENST000	-2,37	LUMINAL A down vs CONTROL

NSUN5P2	NR_033323 // NSUN5P2 // NOP2/Sun domain family, member 5 pseudogene 2 // 7q11.23	-2,37	LUMINAL A down vs CONTROL
SLC2A4	NM_001042 // SLC2A4 // solute carrier family 2 (facilitated glucose transporter)	-2,37	LUMINAL A down vs CONTROL
MAP7D1	NM_018067 // MAP7D1 // MAP7 domain containing 1 // 1p34.3 // 55700 /// ENST00000	-2,37	LUMINAL A down vs CONTROL
TNFRSF10C	NM_003841 // TNFRSF10C // tumor necrosis factor receptor superfamily, member 10c	-2,37	LUMINAL A down vs CONTROL
TAS2R41	NM_176883 // TAS2R41 // taste receptor, type 2, member 41 // 7q35 // 259287 ///	-2,37	LUMINAL A down vs CONTROL
FAM89A	NM_198552 // FAM89A // family with sequence similarity 89, member A // 1q42.2 //	-2,37	LUMINAL A down vs CONTROL
ATN1	NM_001007026 // ATN1 // atrophin 1 // 12p13.31 // 1822 /// NM_001940 // ATN1 //	-2,37	LUMINAL A down vs CONTROL
PRM1	NM_002761 // PRM1 // protamine 1 // 16p13.2 // 5619 /// ENST00000312511 // PRM1	-2,38	LUMINAL A down vs CONTROL
PPP1R12B	NM_032105 // PPP1R12B // protein phosphatase 1, regulatory (inhibitor) subunit 1	-2,38	LUMINAL A down vs CONTROL
SSX8	NR_027250 // SSX8 // synovial sarcoma, X breakpoint 8 // Xp11.23 // 280659 /// A	-2,38	LUMINAL A down vs CONTROL
NOTCH1	NM_017617 // NOTCH1 // Notch homolog 1, translocation-associated (Drosophila) //	-2,38	LUMINAL A down vs CONTROL
FAM167B	NM_032648 // FAM167B // family with sequence similarity 167, member B // 1p35.1	-2,38	LUMINAL A down vs CONTROL
TCF7L1	NM_031283 // TCF7L1 // transcription factor 7-like 1 (T-cell specific, HMG-box)	-2,38	LUMINAL A down vs CONTROL
PPP1R15A	NM_014330 // PPP1R15A // protein phosphatase 1, regulatory (inhibitor) subunit 1	-2,38	LUMINAL A down vs CONTROL
SNCG	NM_003087 // SNCG // synuclein, gamma (breast cancer-specific protein 1) // 10q2	-2,38	LUMINAL A down vs CONTROL
ADAMTS5	NM_007038 // ADAMTS5 // ADAM metallopeptidase with thrombospondin type 1 motif,	-2,39	LUMINAL A down vs CONTROL
PYGM	NM_005609 // PYGM // phosphorylase, glycogen, muscle // 11q12-q13.2 // 5837 ///	-2,39	LUMINAL A down vs CONTROL
CHRM3	NM_000740 // CHRM3 // cholinergic receptor, muscarinic 3 // 1q43 // 1131 /// ENS	-2,39	LUMINAL A down vs CONTROL
RYR3	NM_001036 // RYR3 // ryanodine receptor 3 // 15q14-q15 // 6263 /// ENST000003892	-2,39	LUMINAL A down vs CONTROL
VEGFB	NM_003377 // VEGFB // vascular endothelial growth factor B // 11q13 // 7423 ///	-2,39	LUMINAL A down vs CONTROL
MTA1	NM_004689 // MTA1 // metastasis associated 1 // 14q32.3 // 9112 /// ENST00000331	-2,40	LUMINAL A down vs CONTROL
APCDD1	NM_153000 // APCDD1 // adenomatosis polyposis coli down-regulated 1 // 18p11.22	-2,40	LUMINAL A down vs CONTROL
LDLR	NM_000527 // LDLR // low density lipoprotein receptor // 19p13.3 // 3949 /// ENS	-2,40	LUMINAL A down vs CONTROL
JDP2	NM_001135049 // JDP2 // Jun dimerization protein 2 // 14q24.3 // 122953 /// NM_1	-2,40	LUMINAL A down vs CONTROL
TLE2	NM_003260 // TLE2 // transducin-like enhancer of split 2 (E(sp1) homolog, Drosop	-2,40	LUMINAL A down vs CONTROL
PLEKHM1	NM_014798 // PLEKHM1 // pleckstrin homology domain containing, family M (with RU	-2,41	LUMINAL A down vs CONTROL
VPS4A	NM_013245 // VPS4A // vacuolar protein sorting 4 homolog A (S. cerevisiae) // 16	-2,41	LUMINAL A down vs CONTROL
CASZ1	NM_001079843 // CASZ1 // castor zinc finger 1 // 1p36.22 // 54897 /// NM_017766	-2,41	LUMINAL A down vs CONTROL
TCEAL3	NM_001006933 // TCEAL3 // transcription elongation factor A (SII)-like 3 // Xq22	-2,41	LUMINAL A down vs CONTROL
TYRO3	NM_006293 // TYRO3 // TYRO3 protein tyrosine kinase // 15q15 // 7301 /// ENST000	-2,41	LUMINAL A down vs CONTROL
KIRREL	NM_018240 // KIRREL // kin of IRRE like (Drosophila) // 1q21-q25 // 55243 /// EN	-2,41	LUMINAL A down vs CONTROL
CCDC84	NM_198489 // CCDC84 // coiled-coil domain containing 84 // 11q23.3 // 338657 ///	-2,41	LUMINAL A down vs CONTROL
KALRN	NM_003947 // KALRN // kalirin, RhoGEF kinase // 3q21.2 // 8997 /// NM_001024660	-2,42	LUMINAL A down vs CONTROL

DAB2IP	NM_032552 // DAB2IP // DAB2 interacting protein // 9q33.1-q33.3 // 153090 /// NM	-2,42	LUMINAL A down vs CONTROL
ZNF219	NM_016423 // ZNF219 // zinc finger protein 219 // 14q11 // 51222 /// NM_00110167	-2,42	LUMINAL A down vs CONTROL
TTC38	NM_017931 // TTC38 // tetratricopeptide repeat domain 38 // 22q13 // 55020 /// E	-2,42	LUMINAL A down vs CONTROL
NAT8L	NM_178557 // NAT8L // N-acetyltransferase 8-like (GCN5-related, putative) // 4p1	-2,42	LUMINAL A down vs CONTROL
SIN3B	NM_015260 // SIN3B // SIN3 homolog B, transcription regulator (yeast) // 19p13.1	-2,42	LUMINAL A down vs CONTROL
ALDH1L1	NM_012190 // ALDH1L1 // aldehyde dehydrogenase 1 family, member L1 // 3q21.3 //	-2,42	LUMINAL A down vs CONTROL
TNIP1	NM_006058 // TNIP1 // TNFAIP3 interacting protein 1 // 5q32-q33.1 // 10318 /// E	-2,43	LUMINAL A down vs CONTROL
GNG11	NM_004126 // GNG11 // guanine nucleotide binding protein (G protein), gamma 11 /	-2,44	LUMINAL A down vs CONTROL
CCDC9	NM_015603 // CCDC9 // coiled-coil domain containing 9 // 19q13.32 // 26093 /// E	-2,44	LUMINAL A down vs CONTROL
MATN2	NM_002380 // MATN2 // matrilin 2 // 8q22 // 4147 /// NM_030583 // MATN2 // matri	-2,44	LUMINAL A down vs CONTROL
IGHMBP2	NM_002180 // IGHMBP2 // immunoglobulin mu binding protein 2 // 11q13.3 // 3508 /	-2,44	LUMINAL A down vs CONTROL
LCE1D	NM_178352 // LCE1D // late cornified envelope 1D // 1q21.3 // 353134 /// NM_1783	-2,44	LUMINAL A down vs CONTROL
TRIM8	NM_030912 // TRIM8 // tripartite motif-containing 8 // 10q24.3 // 81603 /// ENST	-2,44	LUMINAL A down vs CONTROL
AIFM2	NM_032797 // AIFM2 // apoptosis-inducing factor, mitochondrion-associated, 2 //	-2,44	LUMINAL A down vs CONTROL
SMTN	NM_134269 // SMTN // smoothelin // 22q12.2 // 6525 /// NM_134270 // SMTN // smoo	-2,45	LUMINAL A down vs CONTROL
EPB49	NM_001978 // EPB49 // erythrocyte membrane protein band 4.9 (dematin) // 8p21.1	-2,45	LUMINAL A down vs CONTROL
GSTA2	NM_000846 // GSTA2 // glutathione S-transferase alpha 2 // 6p12.1 // 2939 /// EN	-2,45	LUMINAL A down vs CONTROL
POLR2E	NM_002695 // POLR2E // polymerase (RNA) II (DNA directed) polypeptide E, 25kDa /	-2,45	LUMINAL A down vs CONTROL
LOC80054	NR_026887 // LOC80054 // hypothetical LOC80054 // 19q13.11 // 80054	-2,45	LUMINAL A down vs CONTROL
AHDC1	NM_001029882 // AHDC1 // AT hook, DNA binding motif, containing 1 // 1p36.13 //	-2,46	LUMINAL A down vs CONTROL
RASD1	NM_016084 // RASD1 // RAS, dexamethasone-induced 1 // 17p11.2 // 51655 /// ENST0	-2,46	LUMINAL A down vs CONTROL
GNG13	NM_016541 // GNG13 // guanine nucleotide binding protein (G protein), gamma 13 /	-2,46	LUMINAL A down vs CONTROL
KRT15	NM_002275 // KRT15 // keratin 15 // 17q21.2 // 3866 /// ENST00000254043 // KRT15	-2,46	LUMINAL A down vs CONTROL
ADCY4	NM_139247 // ADCY4 // adenylate cyclase 4 // 14q12 // 196883 /// ENST00000310677	-2,46	LUMINAL A down vs CONTROL
IL28A	NM_172138 // IL28A // interleukin 28A (interferon, lambda 2) // 19q13.13 // 2826	-2,46	LUMINAL A down vs CONTROL
TUBB2A	NM_001069 // TUBB2A // tubulin, beta 2A // 6p25 // 7280 /// ENST00000333628 // T	-2,47	LUMINAL A down vs CONTROL
CAMK1	NM_003656 // CAMK1 // calcium/calmodulin-dependent protein kinase I // 3p25.3 //	-2,47	LUMINAL A down vs CONTROL
TLN2	NM_015059 // TLN2 // talin 2 // 15q15-q21 // 83660 /// NR_029709 // MIR190 // mi	-2,47	LUMINAL A down vs CONTROL
SPTBN1	NM_003128 // SPTBN1 // spectrin, beta, non-erythrocytic 1 // 2p21 // 6711 /// NM	-2,47	LUMINAL A down vs CONTROL
MT1E	NM_175617 // MT1E // metallothionein 1E // 16q13 // 4493 /// NM_176870 // MT1M /	-2,47	LUMINAL A down vs CONTROL
MAPK10	NM_138982 // MAPK10 // mitogen-activated protein kinase 10 // 4q22.1-q23 // 5602	-2,48	LUMINAL A down vs CONTROL
C17orf91	NR_028502 // C17orf91 // chromosome 17 open reading frame 91 // 17p13.3 // 84981	-2,48	LUMINAL A down vs CONTROL
UBE2E3	NM_006357 // UBE2E3 // ubiquitin-conjugating enzyme E2E 3 (UBC4/5 homolog, yeast	-2,48	LUMINAL A down vs CONTROL

SRP14	NM_003134 // SRP14 // signal recognition particle 14kDa (homologous Alu RNA bind	-2,49	LUMINAL A down vs CONTROL
COL15A1	NM_001855 // COL15A1 // collagen, type XV, alpha 1 // 9q21-q22 // 1306 /// ENST0	-2,50	LUMINAL A down vs CONTROL
BLOC1S1	NM_001487 // BLOC1S1 // biogenesis of lysosomal organelles complex-1, subunit 1	-2,50	LUMINAL A down vs CONTROL
PDE2A	NR_026572 // PDE2A // phosphodiesterase 2A, cGMP-stimulated // 11q13.4 // 5138 /	-2,50	LUMINAL A down vs CONTROL
RAPGEF3	NM_001098532 // RAPGEF3 // Rap guanine nucleotide exchange factor (GEF) 3 // 12q	-2,51	LUMINAL A down vs CONTROL
GLTSCR2	NM_015710 // GLTSCR2 // glioma tumor suppressor candidate region gene 2 // 19q13	-2,51	LUMINAL A down vs CONTROL
AGPAT2	NM_006412 // AGPAT2 // 1-acylglycerol-3-phosphate O-acyltransferase 2 (lysophosp	-2,51	LUMINAL A down vs CONTROL
MAFF	NM_012323 // MAFF // v-maf musculoaponeurotic fibrosarcoma oncogene homolog F (a	-2,51	LUMINAL A down vs CONTROL
FOS	NM_005252 // FOS // FBJ murine osteosarcoma viral oncogene homolog // 14q24.3 //	-2,52	LUMINAL A down vs CONTROL
ECE1	NM_001113349 // ECE1 // endothelin converting enzyme 1 // 1p36.1 // 1889 /// NM_	-2,52	LUMINAL A down vs CONTROL
ANO2	NM_020373 // ANO2 // anoctamin 2 // 12p13.3 // 57101 /// ENST00000327087 // ANO2	-2,53	LUMINAL A down vs CONTROL
DIRAS3	NM_004675 // DIRAS3 // DIRAS family, GTP-binding RAS-like 3 // 1p31 // 9077 ///	-2,53	LUMINAL A down vs CONTROL
NFIX	NM_002501 // NFIX // nuclear factor I/X (CCAAT-binding transcription factor) //	-2,53	LUMINAL A down vs CONTROL
PRLH	NM_015893 // PRLH // prolactin releasing hormone // 2q37.3 // 51052 /// ENST0000	-2,53	LUMINAL A down vs CONTROL
FAM18B	AF151906 // FAM18B // family with sequence similarity 18, member B // 17p11.2 //	-2,53	LUMINAL A down vs CONTROL
LENG8	NM_052925 // LENG8 // leukocyte receptor cluster (LRC) member 8 // 19q13.42 // 1	-2,53	LUMINAL A down vs CONTROL
FGF2	NM_002006 // FGF2 // fibroblast growth factor 2 (basic) // 4q26-q27 // 2247 ///	-2,54	LUMINAL A down vs CONTROL
PCOLCE2	NM_013363 // PCOLCE2 // procollagen C-endopeptidase enhancer 2 // 3q21-q24 // 26	-2,54	LUMINAL A down vs CONTROL
CAV1	NM_001172895 // CAV1 // caveolin 1, caveolae protein, 22kDa // 7q31.1 // 857 ///	-2,54	LUMINAL A down vs CONTROL
LRP5	NM_002335 // LRP5 // low density lipoprotein receptor-related protein 5 // 11q13	-2,54	LUMINAL A down vs CONTROL
PHYHD1	NM_001100876 // PHYHD1 // phytanoyl-CoA dioxygenase domain containing 1 // 9q34.	-2,54	LUMINAL A down vs CONTROL
KRTAP9-4	NM_033191 // KRTAP9-4 // keratin associated protein 9-4 // 17q12-q21 // 85280 //	-2,55	LUMINAL A down vs CONTROL
CDH13	NM_001257 // CDH13 // cadherin 13, H-cadherin (heart) // 16q24.2-q24.3 // 1012 /	-2,55	LUMINAL A down vs CONTROL
CLEC3B	NM_003278 // CLEC3B // C-type lectin domain family 3, member B // 3p22-p21.3 //	-2,55	LUMINAL A down vs CONTROL
OBP2B	NM_014581 // OBP2B // odorant binding protein 2B // 9q34 // 29989 /// NM_014582	-2,55	LUMINAL A down vs CONTROL
C3orf19	NM_016474 // C3orf19 // chromosome 3 open reading frame 19 // 3p25.1 // 51244 //	-2,55	LUMINAL A down vs CONTROL
OXTR	NM_000916 // OXTR // oxytocin receptor // 3p25 // 5021 /// BC137443 // OXTR // o	-2,55	LUMINAL A down vs CONTROL
ADRA2A	NM_000681 // ADRA2A // adrenergic, alpha-2A-, receptor // 10q24-q26 // 150 /// A	-2,56	LUMINAL A down vs CONTROL
CHRM2	NM_001006626 // CHRM2 // cholinergic receptor, muscarinic 2 // 7q31-q35 // 1129	-2,56	LUMINAL A down vs CONTROL
LAMA3	NM_198129 // LAMA3 // laminin, alpha 3 // 18q11.2 // 3909 /// NM_001127717 // LA	-2,57	LUMINAL A down vs CONTROL
PRKAR2B	NM_002736 // PRKAR2B // protein kinase, cAMP-dependent, regulatory, type II, bet	-2,57	LUMINAL A down vs CONTROL
KRT17	NM_000422 // KRT17 // keratin 17 // 17q12-q21 // 3872 /// ENST00000311208 // KRT	-2,57	LUMINAL A down vs CONTROL
KIAA0664	NM_015229 // KIAA0664 // KIAA0664 // 17p13.3 // 23277 /// ENST00000435359 // KIA	-2,57	LUMINAL A down vs CONTROL

PTX3	NM_002852 // PTX3 // pentraxin 3, long // 3q25 // 5806 /// ENST00000295927 // PT	-2,57	LUMINAL A down vs CONTROL
C10orf10	NM_007021 // C10orf10 // chromosome 10 open reading frame 10 // 10q11.21 // 1106	-2,58	LUMINAL A down vs CONTROL
VWF	NM_000552 // VWF // von Willebrand factor // 12p13.3 // 7450 /// ENST00000261405	-2,58	LUMINAL A down vs CONTROL
SSX6	NR_028366 // SSX6 // synovial sarcoma, X breakpoint 6 (pseudogene) // Xp11.2 //	-2,58	LUMINAL A down vs CONTROL
COL4A2	NM_001846 // COL4A2 // collagen, type IV, alpha 2 // 13q34 // 1284 /// ENST00000	-2,58	LUMINAL A down vs CONTROL
GNAO1	NM_020988 // GNAO1 // guanine nucleotide binding protein (G protein), alpha acti	-2,58	LUMINAL A down vs CONTROL
DMD	NM_000109 // DMD // dystrophin // Xp21.2 // 1756 /// NM_004010 // DMD // dystrop	-2,58	LUMINAL A down vs CONTROL
AES	NM_198969 // AES // amino-terminal enhancer of split // 19p13.3 // 166 /// NM_00	-2,59	LUMINAL A down vs CONTROL
VLDLR	NM_003383 // VLDLR // very low density lipoprotein receptor // 9p24 // 7436 ///	-2,59	LUMINAL A down vs CONTROL
CLK3	NM_001130028 // CLK3 // CDC-like kinase 3 // 15q24 // 1198 /// NM_003992 // CLK3	-2,61	LUMINAL A down vs CONTROL
PTPRS	NM_002850 // PTPRS // protein tyrosine phosphatase, receptor type, S // 19p13.3	-2,61	LUMINAL A down vs CONTROL
F3	NM_001993 // F3 // coagulation factor III (thromboplastin, tissue factor) // 1p2	-2,61	LUMINAL A down vs CONTROL
SHISA6	NM_207386 // SHISA6 // shisa homolog 6 (Xenopus laevis) // 17p12 // 388336 /// N	-2,62	LUMINAL A down vs CONTROL
C19orf59	NM_174918 // C19orf59 // chromosome 19 open reading frame 59 // 19p13.2 // 19967	-2,62	LUMINAL A down vs CONTROL
KCNB1	NM_004975 // KCNB1 // potassium voltage-gated channel, Shab-related subfamily, m	-2,62	LUMINAL A down vs CONTROL
ECHDC3	NM_024693 // ECHDC3 // enoyl CoA hydratase domain containing 3 // 10p14 // 79746	-2,64	LUMINAL A down vs CONTROL
NFIC	NM_205843 // NFIC // nuclear factor I/C (CCAAT-binding transcription factor) //	-2,64	LUMINAL A down vs CONTROL
GSN	NM_001127663 // GSN // gelsolin // 9q33 // 2934 /// NM_000177 // GSN // gelsolin	-2,65	LUMINAL A down vs CONTROL
SLIT3	NM_003062 // SLIT3 // slit homolog 3 (Drosophila) // 5q35 // 6586 /// NR_029632	-2,65	LUMINAL A down vs CONTROL
SLC4A2	NM_003040 // SLC4A2 // solute carrier family 4, anion exchanger, member 2 (eryth	-2,65	LUMINAL A down vs CONTROL
TESC	NR_031766 // TESC // tescalcin // 12q24.22 // 54997 /// NM_017899 // TESC // tes	-2,65	LUMINAL A down vs CONTROL
MCF2L	NM_001112732 // MCF2L // MCF.2 cell line derived transforming sequence-like // 1	-2,67	LUMINAL A down vs CONTROL
MAOA	NM_000240 // MAOA // monoamine oxidase A // Xp11.3 // 4128 /// ENST00000338702 /	-2,67	LUMINAL A down vs CONTROL
NTRK3	NM_001007156 // NTRK3 // neurotrophic tyrosine kinase, receptor, type 3 // 15q25	-2,68	LUMINAL A down vs CONTROL
SDPR	NM_004657 // SDPR // serum deprivation response // 2q32-q33 // 8436 /// ENST0000	-2,68	LUMINAL A down vs CONTROL
OR10H4	NM_001004465 // OR10H4 // olfactory receptor, family 10, subfamily H, member 4 /	-2,68	LUMINAL A down vs CONTROL
SIK2	NM_015191 // SIK2 // salt-inducible kinase 2 // 11q23.1 // 23235 /// ENST0000030	-2,69	LUMINAL A down vs CONTROL
SLC29A4	NM_001040661 // SLC29A4 // solute carrier family 29 (nucleoside transporters), m	-2,69	LUMINAL A down vs CONTROL
KRT16	NM_005557 // KRT16 // keratin 16 // 17q12-q21 // 3868 /// NR_029393 // KRT16P3 /	-2,69	LUMINAL A down vs CONTROL
NPR3	NM_000908 // NPR3 // natriuretic peptide receptor C/guanylate cyclase C (atriona	-2,70	LUMINAL A down vs CONTROL
RPL19	NM_000981 // RPL19 // ribosomal protein L19 // 17q11.2-q12 // 6143 /// AB018295	-2,70	LUMINAL A down vs CONTROL
KIAA0284	NM_001112726 // KIAA0284 // KIAA0284 // 14q32.33 // 283638 /// NM_015005 // KIAA	-2,72	LUMINAL A down vs CONTROL
IGF2	NM_000612 // IGF2 // insulin-like growth factor 2 (somatomedin A) // 11p15.5 //	-2,72	LUMINAL A down vs CONTROL

SIRT7	NM_016538 // SIRT7 // sirtuin (silent mating type information regulation 2 homol	-2,72	LUMINAL A down vs CONTROL
SEMA3G	NM_020163 // SEMA3G // sema domain, immunoglobulin domain (Ig), short basic doma	-2,72	LUMINAL A down vs CONTROL
PYGL	NM_002863 // PYGL // phosphorylase, glycogen, liver // 14q21-q22 // 5836 /// NM_	-2,73	LUMINAL A down vs CONTROL
CHRFAM7A	NM_139320 // CHRFAM7A // CHRNA7 (cholinergic receptor, nicotinic, alpha 7, exons	-2,73	LUMINAL A down vs CONTROL
PIP	NM_002652 // PIP // prolactin-induced protein // 7q34 // 5304 /// ENST0000029100	-2,73	LUMINAL A down vs CONTROL
SOCS3	NM_003955 // SOCS3 // suppressor of cytokine signaling 3 // 17q25.3 // 9021 ///	-2,73	LUMINAL A down vs CONTROL
HBCBP	AF529371 // HBCBP // HBcAg-binding protein // 12q13.3 // 100130083 /// ENST00000	-2,74	LUMINAL A down vs CONTROL
DOCK6	NM_020812 // DOCK6 // dedicator of cytokinesis 6 // 19p13.2 // 57572 /// ENST000	-2,74	LUMINAL A down vs CONTROL
EPAS1	NM_001430 // EPAS1 // endothelial PAS domain protein 1 // 2p21-p16 // 2034 /// E	-2,75	LUMINAL A down vs CONTROL
TNMD	NM_022144 // TNMD // tenomodulin // Xq21.33-q23 // 64102 /// ENST00000373031 //	-2,77	LUMINAL A down vs CONTROL
ASAM	NM_024769 // ASAM // adipocyte-specific adhesion molecule // 11q24.1 // 79827 //	-2,78	LUMINAL A down vs CONTROL
C10orf116	NM_006829 // C10orf116 // chromosome 10 open reading frame 116 // 10q23.2 // 109	-2,79	LUMINAL A down vs CONTROL
DEFB125	NM_153325 // DEFB125 // defensin, beta 125 // 20p13 // 245938 /// ENST0000038241	-2,79	LUMINAL A down vs CONTROL
COL17A1	NM_000494 // COL17A1 // collagen, type XVII, alpha 1 // 10q24.3 // 1308 /// NR_0	-2,80	LUMINAL A down vs CONTROL
LCN12	NM_178536 // LCN12 // lipocalin 12 // 9q34.3 // 286256 /// ENST00000371633 // LC	-2,81	LUMINAL A down vs CONTROL
STX11	NM_003764 // STX11 // syntaxin 11 // 6q24.2 // 8676 /// ENST00000367568 // STX11	-2,81	LUMINAL A down vs CONTROL
ACTG2	NM_001615 // ACTG2 // actin, gamma 2, smooth muscle, enteric // 2p13.1 // 72 ///	-2,81	LUMINAL A down vs CONTROL
CBX7	NM_175709 // CBX7 // chromobox homolog 7 // 22q13.1 // 23492 /// ENST00000216133	-2,82	LUMINAL A down vs CONTROL
SAA1	NM_000331 // SAA1 // serum amyloid A1 // 11p15.1 // 6288 /// NM_030754 // SAA2 /	-2,82	LUMINAL A down vs CONTROL
TF	NM_001063 // TF // transferrin // 3q22.1 // 7018 /// NM_021203 // SRPRB // signa	-2,83	LUMINAL A down vs CONTROL
LOC150381	NR_027034 // LOC150381 // hypothetical LOC150381 // 22q13.31 // 150381 /// NR_02	-2,84	LUMINAL A down vs CONTROL
PHLDB1	NM_015157 // PHLDB1 // pleckstrin homology-like domain, family B, member 1 // 11	-2,84	LUMINAL A down vs CONTROL
MT1E	NM_175617 // MT1E // metallothionein 1E // 16q13 // 4493 /// NM_176870 // MT1M /	-2,84	LUMINAL A down vs CONTROL
ALDH2	NM_000690 // ALDH2 // aldehyde dehydrogenase 2 family (mitochondrial) // 12q24.2	-2,84	LUMINAL A down vs CONTROL
TFAP2B	NM_003221 // TFAP2B // transcription factor AP-2 beta (activating enhancer bindi	-2,85	LUMINAL A down vs CONTROL
EGR1	NM_001964 // EGR1 // early growth response 1 // 5q31.1 // 1958 /// ENST000002399	-2,85	LUMINAL A down vs CONTROL
ADM	NM_001124 // ADM // adrenomedullin // 11p15.4 // 133 /// ENST00000278175 // ADM	-2,85	LUMINAL A down vs CONTROL
PC	NM_001040716 // PC // pyruvate carboxylase // 11q13.4-q13.5 // 5091 /// NM_00092	-2,86	LUMINAL A down vs CONTROL
SYNPO	NM_007286 // SYNPO // synaptopodin // 5q33.1 // 11346 /// NM_001166209 // SYNPO	-2,86	LUMINAL A down vs CONTROL
KIAA0427	NM_001142397 // KIAA0427 // KIAA0427 // 18q21.1 // 9811 /// NM_014772 // KIAA042	-2,86	LUMINAL A down vs CONTROL
FASN	NM_004104 // FASN // fatty acid synthase // 17q25 // 2194 /// ENST00000306749 //	-2,86	LUMINAL A down vs CONTROL
RBPMS2	NM_194272 // RBPMS2 // RNA binding protein with multiple splicing 2 // 15q22.31	-2,86	LUMINAL A down vs CONTROL
HYI	NM_031207 // HYI // hydroxypyruvate isomerase homolog (E. coli) // 1p34.2 // 818	-2,87	LUMINAL A down vs CONTROL

MYBPC1	NM_002465 // MYBPC1 // myosin binding protein C, slow type // 12q23.2 // 4604 //	-2,87	LUMINAL A down vs CONTROL
PTRF	NM_012232 // PTRF // polymerase I and transcript release factor // 17q21.2 // 28	-2,89	LUMINAL A down vs CONTROL
FAH	NM_000137 // FAH // fumarylacetoacetate hydrolase (fumarylacetoacetase) // 15q23	-2,89	LUMINAL A down vs CONTROL
KCNE1	NM_000219 // KCNE1 // potassium voltage-gated channel, Isk-related family, membe	-2,90	LUMINAL A down vs CONTROL
ZNF99	NM_001080409 // ZNF99 // zinc finger protein 99 // 19p12 // 7652 /// NM_00100141	-2,91	LUMINAL A down vs CONTROL
C14orf49	NM_152592 // C14orf49 // chromosome 14 open reading frame 49 // 14q32.13 // 1611	-2,91	LUMINAL A down vs CONTROL
HBB	NM_000518 // HBB // hemoglobin, beta // 11p15.5 // 3043 /// ENST00000335295 // H	-2,92	LUMINAL A down vs CONTROL
SOD3	NM_003102 // SOD3 // superoxide dismutase 3, extracellular // 4p15.3-p15.1 // 66	-2,92	LUMINAL A down vs CONTROL
IGF2	NM_000612 // IGF2 // insulin-like growth factor 2 (somatomedin A) // 11p15.5 //	-2,93	LUMINAL A down vs CONTROL
NRBP2	NM_178564 // NRBP2 // nuclear receptor binding protein 2 // 8q24.3 // 340371 ///	-2,94	LUMINAL A down vs CONTROL
PLA2G16	NM_007069 // PLA2G16 // phospholipase A2, group XVI // 11q12.3 // 11145 /// NM_0	-2,95	LUMINAL A down vs CONTROL
TNNT3	NM_006757 // TNNT3 // troponin T type 3 (skeletal, fast) // 11p15.5 // 7140 ///	-2,95	LUMINAL A down vs CONTROL
EBF1	NM_024007 // EBF1 // early B-cell factor 1 // 5q34 // 1879 /// ENST00000313708 /	-2,95	LUMINAL A down vs CONTROL
FAM45B	NR_027141 // FAM45B // family with sequence similarity 45, member A pseudogene /	-2,95	LUMINAL A down vs CONTROL
APOD	NM_001647 // APOD // apolipoprotein D // 3q26.2-qter // 347 /// ENST00000343267	-2,96	LUMINAL A down vs CONTROL
ACSM1	NM_052956 // ACSM1 // acyl-CoA synthetase medium-chain family member 1 // 16p12.	-2,96	LUMINAL A down vs CONTROL
NECAB1	NM_022351 // NECAB1 // N-terminal EF-hand calcium binding protein 1 // 8q21.3 //	-2,97	LUMINAL A down vs CONTROL
TMEM37	NM_183240 // TMEM37 // transmembrane protein 37 // 2q14.2 // 140738 /// ENST0000	-2,97	LUMINAL A down vs CONTROL
CNTFR	NM_147164 // CNTFR // ciliary neurotrophic factor receptor // 9p13 // 1271 /// N	-2,98	LUMINAL A down vs CONTROL
SYNM	NM_145728 // SYNM // synemin, intermediate filament protein // 15q26.3 // 23336	-2,98	LUMINAL A down vs CONTROL
EGFR	NM_005228 // EGFR // epidermal growth factor receptor (erythroblastic leukemia v	-2,98	LUMINAL A down vs CONTROL
TCF7L2	NM_001146274 // TCF7L2 // transcription factor 7-like 2 (T-cell specific, HMG-bo	-2,99	LUMINAL A down vs CONTROL
UGT2B28	NM_053039 // UGT2B28 // UDP glucuronosyltransferase 2 family, polypeptide B28 //	-2,99	LUMINAL A down vs CONTROL
HOOK2	NM_013312 // HOOK2 // hook homolog 2 (Drosophila) // 19p13.2 // 29911 /// NM_001	-3,00	LUMINAL A down vs CONTROL
PPARG	NM_138712 // PPARG // peroxisome proliferator-activated receptor gamma // 3p25 /	-3,03	LUMINAL A down vs CONTROL
CEBPA	NM_004364 // CEBPA // CCAAT/enhancer binding protein (C/EBP), alpha // 19q13.1 /	-3,05	LUMINAL A down vs CONTROL
SLC7A10	NM_019849 // SLC7A10 // solute carrier family 7, (neutral amino acid transporter	-3,06	LUMINAL A down vs CONTROL
TRIM29	NM_012101 // TRIM29 // tripartite motif-containing 29 // 11q22-q23 // 23650 ///	-3,06	LUMINAL A down vs CONTROL
FADS3	NM_021727 // FADS3 // fatty acid desaturase 3 // 11q12-q13.1 // 3995 /// ENST000	-3,08	LUMINAL A down vs CONTROL
NPR1	NM_000906 // NPR1 // natriuretic peptide receptor A/guanylate cyclase A (atriona	-3,08	LUMINAL A down vs CONTROL
PDZD2	NM_178140 // PDZD2 // PDZ domain containing 2 // 5p13.3 // 23037 /// ENST0000043	-3,09	LUMINAL A down vs CONTROL
CCDC69	NM_015621 // CCDC69 // coiled-coil domain containing 69 // 5q33.1 // 26112 /// E	-3,09	LUMINAL A down vs CONTROL
HEPACAM	NM_152722 // HEPACAM // hepatocyte cell adhesion molecule // 11q24.2 // 220296 /	-3,10	LUMINAL A down vs CONTROL

HRASLS5	NM_054108 // HRASLS5 // HRAS-like suppressor family, member 5 // 11q13.2 // 1172	-3,12	LUMINAL A down vs CONTROL
SLC19A3	NM_025243 // SLC19A3 // solute carrier family 19, member 3 // 2q37 // 80704 ///	-3,12	LUMINAL A down vs CONTROL
KDM6B	NM_001080424 // KDM6B // lysine (K)-specific demethylase 6B // 17p13.1 // 23135	-3,12	LUMINAL A down vs CONTROL
NMT2	NM_004808 // NMT2 // N-myristoyltransferase 2 // 10p13 // 9397 /// ENST000003781	-3,13	LUMINAL A down vs CONTROL
LHX5	NM_022363 // LHX5 // LIM homeobox 5 // 12q24 // 64211 /// ENST00000261731 // LHX	-3,13	LUMINAL A down vs CONTROL
MOSC1	NM_022746 // MOSC1 // MOCO sulphurase C-terminal domain containing 1 // 1q41 //	-3,14	LUMINAL A down vs CONTROL
GCOM1	NM_001018090 // GCOM1 // GRINL1A complex locus // 15q21.3 // 145781 /// NM_00101	-3,14	LUMINAL A down vs CONTROL
POU5F1P4	GU480875 // POU5F1P4 // POU class 5 homeobox 1 pseudogene 4 // 1q22 // 645682 //	-3,15	LUMINAL A down vs CONTROL
GSTM2	NM_000848 // GSTM2 // glutathione S-transferase mu 2 (muscle) // 1p13.3 // 2946	-3,15	LUMINAL A down vs CONTROL
DTX1	NM_004416 // DTX1 // deltex homolog 1 (Drosophila) // 12q24.13 // 1840 /// ENST0	-3,18	LUMINAL A down vs CONTROL
CIDEA	NM_001279 // CIDEA // cell death-inducing DFFA-like effector a // 18p11.21|18 //	-3,19	LUMINAL A down vs CONTROL
COMMD9	NM_014186 // COMMD9 // COMM domain containing 9 // 11p13 // 29099 /// NM_0011016	-3,20	LUMINAL A down vs CONTROL
ME3	NM_001161586 // ME3 // malic enzyme 3, NADP(+)-dependent, mitochondrial // 11cen	-3,22	LUMINAL A down vs CONTROL
TAAR1	NM_138327 // TAAR1 // trace amine associated receptor 1 // 6q23.2 // 134864 ///	-3,27	LUMINAL A down vs CONTROL
MRAS	NM_012219 // MRAS // muscle RAS oncogene homolog // 3q22.3 // 22808 /// NM_00108	-3,28	LUMINAL A down vs CONTROL
TNS4	NM_032865 // TNS4 // tensin 4 // 17q21.2 // 84951 /// ENST00000254051 // TNS4 //	-3,29	LUMINAL A down vs CONTROL
TENC1	NM_170754 // TENC1 // tensin like C1 domain containing phosphatase (tensin 2) //	-3,29	LUMINAL A down vs CONTROL
PALM	NM_002579 // PALM // paralemmin // 19p13.3 // 5064 /// NM_001040134 // PALM // p	-3,29	LUMINAL A down vs CONTROL
POM121	NM_172020 // POM121 // POM121 membrane glycoprotein (rat) // 7q11.23 // 9883 ///	-3,30	LUMINAL A down vs CONTROL
ALDOC	NM_005165 // ALDOC // aldolase C, fructose-bisphosphate // 17cen-q12 // 230 ///	-3,30	LUMINAL A down vs CONTROL
RBMY1F	NM_152585 // RBMY1F // RNA binding motif protein, Y-linked, family 1, member F /	-3,31	LUMINAL A down vs CONTROL
C12orf39	NM_030572 // C12orf39 // chromosome 12 open reading frame 39 // 12p12.1 // 80763	-3,32	LUMINAL A down vs CONTROL
TINAGL1	NM_022164 // TINAGL1 // tubulointerstitial nephritis antigen-like 1 // 1p35.2 //	-3,33	LUMINAL A down vs CONTROL
IL6	NM_000600 // IL6 // interleukin 6 (interferon, beta 2) // 7p21 // 3569 /// ENST0	-3,35	LUMINAL A down vs CONTROL
CDO1	NM_001801 // CDO1 // cysteine dioxygenase, type I // 5q22-q23 // 1036 /// ENST00	-3,36	LUMINAL A down vs CONTROL
CCDC3	NM_031455 // CCDC3 // coiled-coil domain containing 3 // 10p13 // 83643 /// ENST	-3,37	LUMINAL A down vs CONTROL
C2orf40	NM_032411 // C2orf40 // chromosome 2 open reading frame 40 // 2q12.2 // 84417 //	-3,38	LUMINAL A down vs CONTROL
SORBS1	NM_001034954 // SORBS1 // sorbin and SH3 domain containing 1 // 10q23.3-q24.1 //	-3,38	LUMINAL A down vs CONTROL
EGFL6	NM_015507 // EGFL6 // EGF-like-domain, multiple 6 // Xp22 // 25975 /// NM_001167	-3,39	LUMINAL A down vs CONTROL
MLXIPL	NM_032951 // MLXIPL // MLX interacting protein-like // 7q11.23 // 51085 /// NM_0	-3,39	LUMINAL A down vs CONTROL
FIGF	NM_004469 // FIGF // c-fos induced growth factor (vascular endothelial growth fa	-3,41	LUMINAL A down vs CONTROL
PALM2-AKAP2	NM_007203 // PALM2-AKAP2 // PALM2-AKAP2 readthrough // 9q31-q33 // 445815 /// NM	-3,41	LUMINAL A down vs CONTROL
WASF3	NM_006646 // WASF3 // WAS protein family, member 3 // 13q12 // 10810 /// NM_0010	-3,41	LUMINAL A down vs CONTROL

MGLL	NM_007283 // MGLL // monoglyceride lipase // 3q21.3 // 11343 /// NM_001003794 //	-3,42	LUMINAL A down vs CONTROL
ABLIM3	NM_014945 // ABLIM3 // actin binding LIM protein family, member 3 // 5q32 // 228	-3,42	LUMINAL A down vs CONTROL
STAC2	NM_198993 // STAC2 // SH3 and cysteine rich domain 2 // 17q12 // 342667 /// ENST	-3,42	LUMINAL A down vs CONTROL
CNN1	NM_001299 // CNN1 // calponin 1, basic, smooth muscle // 19p13.2-p13.1 // 1264 /	-3,43	LUMINAL A down vs CONTROL
PNPLA2	NM_020376 // PNPLA2 // patatin-like phospholipase domain containing 2 // 11p15.5	-3,44	LUMINAL A down vs CONTROL
CHRDL1	NM_145234 // CHRDL1 // chordin-like 1 // Xq23 // 91851 /// NM_001143981 // CHRDL	-3,44	LUMINAL A down vs CONTROL
AQP7	NM_001170 // AQP7 // aquaporin 7 // 9p13 // 364 /// NR_002817 // AQP7P1 // aquap	-3,45	LUMINAL A down vs CONTROL
TRIP10	NM_004240 // TRIP10 // thyroid hormone receptor interactor 10 // 19p13.3 // 9322	-3,49	LUMINAL A down vs CONTROL
PFKFB1	NM_002625 // PFKFB1 // 6-phosphofructo-2-kinase/fructose-2,6-biphosphatase 1 //	-3,51	LUMINAL A down vs CONTROL
KLB	NM_175737 // KLB // klotho beta // 4p14 // 152831 /// ENST00000257408 // KLB //	-3,51	LUMINAL A down vs CONTROL
PALMD	NM_017734 // PALMD // palmdelphin // 1p22-p21 // 54873 /// ENST00000263174 // PA	-3,52	LUMINAL A down vs CONTROL
BTNL9	NM_152547 // BTNL9 // butyrophilin-like 9 // 5q35.3 // 153579 /// ENST0000032770	-3,55	LUMINAL A down vs CONTROL
PFKFB3	NM_004566 // PFKFB3 // 6-phosphofructo-2-kinase/fructose-2,6-biphosphatase 3 //	-3,55	LUMINAL A down vs CONTROL
WIF1	NM_007191 // WIF1 // WNT inhibitory factor 1 // 12q14.3 // 11197 /// ENST0000028	-3,56	LUMINAL A down vs CONTROL
JUND	NM_005354 // JUND // jun D proto-oncogene // 19p13.2 // 3727 /// ENST00000252818	-3,57	LUMINAL A down vs CONTROL
PCK1	NM_002591 // PCK1 // phosphoenolpyruvate carboxykinase 1 (soluble) // 20q13.31 /	-3,63	LUMINAL A down vs CONTROL
CAV2	NM_001233 // CAV2 // caveolin 2 // 7q31.1 // 858 /// NM_198212 // CAV2 // caveol	-3,68	LUMINAL A down vs CONTROL
S100B	NM_006272 // S100B // S100 calcium binding protein B // 21q22.3 // 6285 /// ENST	-3,68	LUMINAL A down vs CONTROL
SGK2	NM_016276 // SGK2 // serum/glucocorticoid regulated kinase 2 // 20q13.2 // 10110	-3,83	LUMINAL A down vs CONTROL
CD36	NM_001001548 // CD36 // CD36 molecule (thrombospondin receptor) // 7q11.2 // 948	-3,84	LUMINAL A down vs CONTROL
AKR1C2	NM_205845 // AKR1C2 // aldo-keto reductase family 1, member C2 (dihydrodiol dehy	-3,90	LUMINAL A down vs CONTROL
TCEAL6	NM_001006938 // TCEAL6 // transcription elongation factor A (SII)-like 6 // Xq22	-3,95	LUMINAL A down vs CONTROL
KRT5	NM_000424 // KRT5 // keratin 5 // 12q12-q13 // 3852 /// ENST00000252242 // KRT5	-3,98	LUMINAL A down vs CONTROL
TFPI2	NM_006528 // TFPI2 // tissue factor pathway inhibitor 2 // 7q22 // 7980 /// ENST	-3,98	LUMINAL A down vs CONTROL
TIMP4	NM_003256 // TIMP4 // TIMP metallopeptidase inhibitor 4 // 3p25 // 7079 /// ENST	-3,99	LUMINAL A down vs CONTROL
TPM2	NM_003289 // TPM2 // tropomyosin 2 (beta) // 9p13.2-p13.1 // 7169 /// NM_213674	-4,00	LUMINAL A down vs CONTROL
RBP7	NM_052960 // RBP7 // retinol binding protein 7, cellular // 1p36.22 // 116362 //	-4,03	LUMINAL A down vs CONTROL
MYL9	NM_006097 // MYL9 // myosin, light chain 9, regulatory // 20q11.23 // 10398 ///	-4,04	LUMINAL A down vs CONTROL
CFD	NM_001928 // CFD // complement factor D (adipsin) // 19p13.3 // 1675 /// ENST000	-4,06	LUMINAL A down vs CONTROL
SAA1	NM_000331 // SAA1 // serum amyloid A1 // 11p15.1 // 6288 /// NM_001178006 // SAA	-4,10	LUMINAL A down vs CONTROL
GPAM	NM_020918 // GPAM // glycerol-3-phosphate acyltransferase, mitochondrial // 10q2	-4,12	LUMINAL A down vs CONTROL
PDZK1	NM_002614 // PDZK1 // PDZ domain containing 1 // 1q21 // 5174 /// NR_003377 // P	-4,24	LUMINAL A down vs CONTROL
FOSB	NM_006732 // FOSB // FBJ murine osteosarcoma viral oncogene homolog B // 19q13.3	-4,24	LUMINAL A down vs CONTROL

TUSC5	NM_172367 // TUSC5 // tumor suppressor candidate 5 // 17p13.3 // 286753 /// ENST	-4,26	LUMINAL A down vs CONTROL
GSTM5	NM_000851 // GSTM5 // glutathione S-transferase mu 5 // 1p13.3 // 2949 /// ENST0	-4,34	LUMINAL A down vs CONTROL
OR1E1	NM_003553 // OR1E1 // olfactory receptor, family 1, subfamily E, member 1 // 17p	-4,38	LUMINAL A down vs CONTROL
ITGA7	NM_001144996 // ITGA7 // integrin, alpha 7 // 12q13 // 3679 /// NM_002206 // ITG	-4,43	LUMINAL A down vs CONTROL
ANKRD30A	NM_052997 // ANKRD30A // ankyrin repeat domain 30A // 10p11.21 // 91074 /// NM_0	-4,47	LUMINAL A down vs CONTROL
MT1M	NM_176870 // MT1M // metallothionein 1M // 16q13 // 4499 /// ENST00000379818 //	-4,53	LUMINAL A down vs CONTROL
HRCT1	NM_001039792 // HRCT1 // histidine rich carboxyl terminus 1 // 9p13.3 // 646962	-4,53	LUMINAL A down vs CONTROL
ZBTB16	NM_006006 // ZBTB16 // zinc finger and BTB domain containing 16 // 11q23.1 // 77	-4,63	LUMINAL A down vs CONTROL
ANGPTL4	NM_139314 // ANGPTL4 // angiopoietin-like 4 // 19p13.3 // 51129 /// NM_001039667	-4,64	LUMINAL A down vs CONTROL
RDH5	NM_002905 // RDH5 // retinol dehydrogenase 5 (11-cis/9-cis) // 12q13-q14 // 5959	-4,78	LUMINAL A down vs CONTROL
LGALS12	NM_001142535 // LGALS12 // lectin, galactoside-binding, soluble, 12 // 11q13 //	-4,81	LUMINAL A down vs CONTROL
GLYAT	NM_201648 // GLYAT // glycine-N-acyltransferase // 11q12.1 // 10249 /// NM_00583	-4,82	LUMINAL A down vs CONTROL
PRSS3	NM_007343 // PRSS3 // protease, serine, 3 // 9p11.2 // 5646 /// NM_002771 // PRS	-4,89	LUMINAL A down vs CONTROL
KCNIP2	NM_014591 // KCNIP2 // Kv channel interacting protein 2 // 10q24 // 30819 /// NM	-4,92	LUMINAL A down vs CONTROL
TNS1	NM_022648 // TNS1 // tensin 1 // 2q35-q36 // 7145 /// ENST00000171887 // TNS1 //	-4,94	LUMINAL A down vs CONTROL
ITIH5	NM_030569 // ITIH5 // inter-alpha (globulin) inhibitor H5 // 10p14 // 80760 ///	-4,96	LUMINAL A down vs CONTROL
AKR1C1	NM_001353 // AKR1C1 // aldo-keto reductase family 1, member C1 (dihydrodiol dehy	-5,17	LUMINAL A down vs CONTROL
LIPE	NM_005357 // LIPE // lipase, hormone-sensitive // 19q13.2 // 3991 /// ENST000002	-5,17	LUMINAL A down vs CONTROL
SFRP1	NM_003012 // SFRP1 // secreted frizzled-related protein 1 // 8p12-p11.1 // 6422	-5,35	LUMINAL A down vs CONTROL
GYG2	NM_003918 // GYG2 // glycogenin 2 // Xp22.3 // 8908 /// NM_001079855 // GYG2 //	-5,37	LUMINAL A down vs CONTROL
FHL1	NM_001159702 // FHL1 // four and a half LIM domains 1 // Xq26 // 2273 /// NM_001	-5,44	LUMINAL A down vs CONTROL
MYH11	NM_022844 // MYH11 // myosin, heavy chain 11, smooth muscle // 16p13.11 // 4629	-5,51	LUMINAL A down vs CONTROL
HSPB7	NM_014424 // HSPB7 // heat shock 27kDa protein family, member 7 (cardiovascular)	-5,76	LUMINAL A down vs CONTROL
LOC388152	BC054509 // LOC388152 // hypothetical LOC388152 // 15q25.2 // 388152	-5,77	LUMINAL A down vs CONTROL
MCAM	NM_006500 // MCAM // melanoma cell adhesion molecule // 11q23.3 // 4162 /// ENST	-5,81	LUMINAL A down vs CONTROL
DEFB132	NM_207469 // DEFB132 // defensin, beta 132 // 20p13 // 400830 /// ENST0000038237	-5,86	LUMINAL A down vs CONTROL
HSPB6	NM_144617 // HSPB6 // heat shock protein, alpha-crystallin-related, B6 // 19q13.	-5,97	LUMINAL A down vs CONTROL
ACACB	NM_001093 // ACACB // acetyl-CoA carboxylase beta // 12q24.11 // 32 /// ENST0000	-6,40	LUMINAL A down vs CONTROL
CALB2	NM_001740 // CALB2 // calbindin 2 // 16q22.2 // 794 /// NR_027910 // CALB2 // ca	-6,44	LUMINAL A down vs CONTROL
DGAT2	NM_032564 // DGAT2 // diacylglycerol O-acyltransferase homolog 2 (mouse) // 11q1	-6,63	LUMINAL A down vs CONTROL
LPL	NM_000237 // LPL // lipoprotein lipase // 8p22 // 4023 /// ENST00000311322 // LP	-6,79	LUMINAL A down vs CONTROL
CIDEC	NM_022094 // CIDEC // cell death-inducing DFFA-like effector c // 3p25.3 // 6392	-7,12	LUMINAL A down vs CONTROL
AOC3	NM_003734 // AOC3 // amine oxidase, copper containing 3 (vascular adhesion prote	-7,17	LUMINAL A down vs CONTROL

TAT	NM_000353 // TAT // tyrosine aminotransferase // 16q22.1 // 6898 /// ENST0000035	-7,59	LUMINAL A down vs CONTROL
PLIN1	NM_002666 // PLIN1 // perilipin 1 // 15q26 // 5346 /// NM_001145311 // PLIN1 //	-7,96	LUMINAL A down vs CONTROL
ADIPOQ	NM_001177800 // ADIPOQ // adiponectin, C1Q and collagen domain containing // 3q2	-8,05	LUMINAL A down vs CONTROL
RBP4	NM_006744 // RBP4 // retinol binding protein 4, plasma // 10q23-q24 // 5950 ///	-8,44	LUMINAL A down vs CONTROL
G0S2	NM_015714 // G0S2 // G0/G1switch 2 // 1q32.2 // 50486 /// ENST00000367029 // G0S	-8,63	LUMINAL A down vs CONTROL
LEP	NM_000230 // LEP // leptin // 7q31.3 // 3952 /// NR_029596 // MIR129-1 // microR	-9,35	LUMINAL A down vs CONTROL
FDXACB1	NM_138378 // FDXACB1 // ferredoxin-fold anticodon binding domain containing 1 //	-10,16	LUMINAL A down vs CONTROL
PPP1R1A	NM_006741 // PPP1R1A // protein phosphatase 1, regulatory (inhibitor) subunit 1A	-10,46	LUMINAL A down vs CONTROL
THRSP	NM_003251 // THRSP // thyroid hormone responsive (SPOT14 homolog, rat) // 11q13.	-11,95	LUMINAL A down vs CONTROL
MUCL1	NM_058173 // MUCL1 // mucin-like 1 // 12q // 118430 /// ENST00000308796 // MUCL1	-12,49	LUMINAL A down vs CONTROL
GPD1	NM_005276 // GPD1 // glycerol-3-phosphate dehydrogenase 1 (soluble) // 12q12-q13	-13,31	LUMINAL A down vs CONTROL
FABP4	NM_001442 // FABP4 // fatty acid binding protein 4, adipocyte // 8q21 // 2167 //	-14,46	LUMINAL A down vs CONTROL
