# Supplementary material for: NRIP1 is activated by C-JUN/C-FOS and activates the expression of PGR, ESR1 and CCND1 in luminal A breast cancer
Source: Sci Rep. 2021 Oct 27;11:21159. doi: 10.1038/s41598-021-00291-w (PMC8551324; doi:10.1038/s41598-021-00291-w)
Supplement: Supplementary file 11 — Supplementary Table S3. [file 41598_2021_291_MOESM11_ESM.pdf]

Supplementary Table 3: List of the differentially expressed genes that were identified by chip array assay and potentially regulated by C-JUN and C-FOS.

Gene Symbol	gene_assignment	Fold-Change	Fold-Change(LUMINAL A vs. CONTROL)
ABLIM3	NM_014945 // ABLIM3 // actin binding LIM protein family, member 3 // 5q32 // 228	-3,42	LUMINAL A down vs CONTROL
ALG8	NM_024079 // ALG8 // asparagine-linked glycosylation 8, alpha-1,3-glucosyltransf	3,56	LUMINAL A up vs CONTROL
AQR	NM_014691 // AQR // aquarius homolog (mouse) // 15q14 // 9716 /// ENST0000015647	2,12	LUMINAL A up vs CONTROL
ATP2C1	NM_014382 // ATP2C1 // ATPase, Ca++ transporting, type 2C, member 1 // 3q22.1 //	2,07	LUMINAL A up vs CONTROL
C12orf51	NM_001109662 // C12orf51 // chromosome 12 open reading frame 51 // 12q24.13 // 2	2,10	LUMINAL A up vs CONTROL
CHML	NM_001821 // CHML // choroideremia-like (Rab escort protein 2) // 1q42-qter // 1	2,40	LUMINAL A up vs CONTROL
CMTM6	NM_017801 // CMTM6 // CKLF-like MARVEL transmembrane domain containing 6 // 3p22	3,10	LUMINAL A up vs CONTROL
DCTN5	NM_032486 // DCTN5 // dynactin 5 (p25) // 16p12.2 // 84516 /// ENST00000300087 /	3,53	LUMINAL A up vs CONTROL
DDX46	NM_014829 // DDX46 // DEAD (Asp-Glu-Ala-Asp) box polypeptide 46 // 5q31.1 // 987	2,04	LUMINAL A up vs CONTROL
**DNAJC10**	**NM_018981 // DNAJC10 // DnaJ (Hsp40) homolog, subfamily C, member 10 // 2q32.1 /**	**4,79**	**LUMINAL A up vs CONTROL**
DYNLT1	NM_006519 // DYNLT1 // dynein, light chain, Tctex-type 1 // 6q25.2-q25.3 // 6993	2,88	LUMINAL A up vs CONTROL
GLYAT	NM_201648 // GLYAT // glycine-N-acyltransferase // 11q12.1 // 10249 /// NM_00583	-4,82	LUMINAL A down vs CONTROL
HIF1A	NM_001530 // HIF1A // hypoxia inducible factor 1, alpha subunit (basic helix-loo	3,18	LUMINAL A up vs CONTROL
IARS2	NM_018060 // IARS2 // isoleucyl-tRNA synthetase 2, mitochondrial // 1q41 // 5569	2,74	LUMINAL A up vs CONTROL
**INHBA**	**NM_002192 // INHBA // inhibin, beta A // 7p15-p13 // 3624 /// ENST00000242208 //**	**2,68**	**LUMINAL A up vs CONTROL**
ITK	NM_005546 // ITK // IL2-inducible T-cell kinase // 5q31-q32 // 3702 /// ENST0000	2,24	LUMINAL A up vs CONTROL
JAK2	NM_004972 // JAK2 // Janus kinase 2 // 9p24 // 3717 /// ENST00000381652 // JAK2	2,14	LUMINAL A up vs CONTROL
LNPEP	NM_005575 // LNPEP // leucyl/cystinyl aminopeptidase // 5q15 // 4012 /// NM_1759	2,75	LUMINAL A up vs CONTROL
MAPRE1	NM_012325 // MAPRE1 // microtubule-associated protein, RP/EB family, member 1 //	2,06	LUMINAL A up vs CONTROL
MUC15	NM_001135091 // MUC15 // mucin 15, cell surface associated // 11p14.3 // 143662	2,03	LUMINAL A up vs CONTROL
NFIL3	NM_005384 // NFIL3 // nuclear factor, interleukin 3 regulated // 9q22 // 4783 //	-2,09	LUMINAL A down vs CONTROL
**NRIP1**	**NM_003489 // NRIP1 // nuclear receptor interacting protein 1 // 21q11.2 // 8204**	**2,83**	**LUMINAL A up vs CONTROL**
PCOLCE2	NM_013363 // PCOLCE2 // procollagen C-endopeptidase enhancer 2 // 3q21-q24 // 26	-2,54	LUMINAL A down vs CONTROL
PHIP	NM_017934 // PHIP // pleckstrin homology domain interacting protein // 6q14 // 5	2,22	LUMINAL A up vs CONTROL
POC5	NM_001099271 // POC5 // POC5 centriolar protein homolog (Chlamydomonas) // 5q13.	2,15	LUMINAL A up vs CONTROL
PRKACB	NM_182948 // PRKACB // protein kinase, cAMP-dependent, catalytic, beta // 1p36.1	2,37	LUMINAL A up vs CONTROL
PRRC1	NM_130809 // PRRC1 // proline-rich coiled-coil 1 // 5q23.2 // 133619 /// ENST000	3,53	LUMINAL A up vs CONTROL
PTK2	NM_153831 // PTK2 // PTK2 protein tyrosine kinase 2 // 8q24-qter // 5747 /// NM_	2,20	LUMINAL A up vs CONTROL
RAB10	NM_016131 // RAB10 // RAB10, member RAS oncogene family // 2p23.3 // 10890 /// E	2,47	LUMINAL A up vs CONTROL
RAB30	NM_014488 // RAB30 // RAB30, member RAS oncogene family // 11q12-q14 // 27314 //	3,16	LUMINAL A up vs CONTROL
RNF103	NM_005667 // RNF103 // ring finger protein 103 // 2p11.2 // 7844 /// ENST0000023	3,52	LUMINAL A up vs CONTROL

RPF1	NM_025065 // RPF1 // ribosome production factor 1 homolog (S. cerevisiae) // 1p2	2,24	LUMINAL A up vs CONTROL
RPS25	NM_001028 // RPS25 // ribosomal protein S25 // 11q23.3 // 6230 /// ENST000002369	-2,04	LUMINAL A down vs CONTROL
SBDS	NM_016038 // SBDS // Shwachman-Bodian-Diamond syndrome // 7q11.21 // 51119 /// N	2,30	LUMINAL A up vs CONTROL
SHOC2	NM_007373 // SHOC2 // soc-2 suppressor of clear homolog (C. elegans) // 10q25 //	3,20	LUMINAL A up vs CONTROL
SRP54	NM_003136 // SRP54 // signal recognition particle 54kDa // 14q13.2 // 6729 /// N	3,51	LUMINAL A up vs CONTROL
SSB	NM_003142 // SSB // Sjogren syndrome antigen B (autoantigen La) // 2q31.1 // 674	2,38	LUMINAL A up vs CONTROL
SYCP2	NM_014258 // SYCP2 // synaptonemal complex protein 2 // 20q13.33 // 10388 /// EN	5,49	LUMINAL A up vs CONTROL
TARBP1	NM_005646 // TARBP1 // TAR (HIV-1) RNA binding protein 1 // 1q42.3 // 6894 /// E	2,15	LUMINAL A up vs CONTROL
TMEM30A	NM_018247 // TMEM30A // transmembrane protein 30A // 6q14.1 // 55754 /// NM_0011	2,84	LUMINAL A up vs CONTROL
TNPO1	NM_002270 // TNPO1 // transportin 1 // 5q13.2 // 3842 /// NM_153188 // TNPO1 //	2,29	LUMINAL A up vs CONTROL
UBA5	NM_024818 // UBA5 // ubiquitin-like modifier activating enzyme 5 // 3q22.1 // 79	2,45	LUMINAL A up vs CONTROL
VPS26A	NM_004896 // VPS26A // vacuolar protein sorting 26 homolog A (S. pombe) // 10q21	2,03	LUMINAL A up vs CONTROL
**YTHDF3**	**NM_152758 // YTHDF3 // YTH domain family, member 3 // 8q12.3 // 253943 /// ENST0**	**2,85**	**LUMINAL A up vs CONTROL**
**ZBTB6**	**NM_006626 // ZBTB6 // zinc finger and BTB domain containing 6 // 9q33.2 // 10773**	**2,74**	**LUMINAL A up vs CONTROL**
