# Supplementary material for: NRIP1 is activated by C-JUN/C-FOS and activates the expression of PGR, ESR1 and CCND1 in luminal A breast cancer
Source: Sci Rep. 2021 Oct 27;11:21159. doi: 10.1038/s41598-021-00291-w (PMC8551324; doi:10.1038/s41598-021-00291-w)
Supplement: Supplementary file 12 — Supplementary Table S4. [file 41598_2021_291_MOESM12_ESM.pdf]

Supplementary Table 4: List of 762 differentially expressed genes identified by the chip array assay related to NRIP1 silencing.

GeneSymbol	FC ([SI] vs [SC])	Description	RefSeqAccession
ABCC6P1	3,61	Homo sapiens ATP-binding cassette, sub-family C, member 6 pseudogene 1 (functional) (ABCC6P1), non-coding RNA [NR_003569]	NR_003569
ABO	1,77	Homo sapiens ABO blood group (transferase A, alpha 1-3-N-acetylgalactosaminyltransferase; transferase B, alpha 1-3-galactosyltransferase) (ABO), mRNA [NM_020469]	NM_020469
ACSM5	-7,26	Homo sapiens acyl-CoA synthetase medium-chain family member 5 (ACSM5), mRNA [NM_017888]	NM_017888
AES	1,82	Homo sapiens amino-terminal enhancer of split (AES), transcript variant 1, mRNA [NM_198969]	NM_198969
AGBL2	1,79	Homo sapiens ATP/GTP binding protein-like 2 (AGBL2), mRNA [NM_024783]	NM_024783
AGER	1,83	Homo sapiens advanced glycosylation end product-specific receptor (AGER), transcript variant 9, mRNA [NM_001206966]	NM_001206966
ALX4	2,09	ALX homeobox 4 [Source:HGNC Symbol;Acc:HGNC:450] [ENST00000329255]	
AMIGO3	1,59	Homo sapiens adhesion molecule with Ig-like domain 3 (AMIGO3), mRNA [NM_198722]	NM_198722
ANKRD11	1,75	Homo sapiens ankyrin repeat domain 11 (ANKRD11), transcript variant 1, mRNA [NM_001256182]	NM_001256182
ANKRD18A	1,57	Homo sapiens ankyrin repeat domain 18A (ANKRD18A), mRNA [NM_147195]	NM_147195
ANKRD2	-1,58	Homo sapiens ankyrin repeat domain 2 (stretch responsive muscle) (ANKRD2), transcript variant 1, mRNA [NM_001291218]	NM_001291218
ANKRD29	-5,10	Homo sapiens ankyrin repeat domain 29 (ANKRD29), mRNA [NM_173505]	NM_173505
ANKRD30A	-2,59	Homo sapiens ankyrin repeat domain 30A (ANKRD30A), mRNA [NM_052997]	NM_052997
ANKRD30BL	1,56	Homo sapiens ankyrin repeat domain 30B-like (ANKRD30BL), transcript variant 1, non-coding RNA [NR_027019]	NR_027019
ANKRD46	-1,89	Homo sapiens ankyrin repeat domain 46 (ANKRD46), transcript variant 2, mRNA [NM_198401]	NM_198401
ANKRD53	1,58	Homo sapiens ankyrin repeat domain 53 (ANKRD53), transcript variant 2, mRNA [NM_024933]	NM_024933
ANXA3	-1,50	Homo sapiens annexin A3 (ANXA3), mRNA [NM_005139]	NM_005139
AP1S2	-13,45	adaptor-related protein complex 1, sigma 2 subunit [Source:HGNC Symbol;Acc:HGNC:560] [ENST00000380291]	XM_005274612
APCDD1	-1,70	Homo sapiens adenomatosis polyposis coli down-regulated 1 (APCDD1), mRNA [NM_153000]	NM_153000
APOBEC3F	-2,01	Homo sapiens apolipoprotein B mRNA editing enzyme, catalytic polypeptide-like 3F (APOBEC3F), transcript variant 1, mRNA [NM_145298]	NM_145298
APOD	-1,55	Homo sapiens apolipoprotein D (APOD), mRNA [NM_001647]	NM_001647
AREL1	-1,52	Homo sapiens apoptosis resistant E3 ubiquitin protein ligase 1 (AREL1), mRNA [NM_001039479]	NM_001039479
ARGFXP2	-2,91	Homo sapiens arginine-fifty homeobox pseudogene 2 (ARGFXP2), non-coding RNA [NR_002222]	NR_002222
ARGLU1	1,96	PREDICTED: Homo sapiens arginine and glutamate rich 1 (ARGLU1), transcript variant X1, misc_RNA [XR_243043]	XR_243043
ARHGAP17	1,56	Rho GTPase activating protein 17 [Source:HGNC Symbol;Acc:HGNC:18239] [ENST00000573703]	
ARHGAP21	1,88	Homo sapiens Rho GTPase activating protein 21 (ARHGAP21), mRNA [NM_020824]	NM_020824
ARHGAP6	1,79	Homo sapiens Rho GTPase activating protein 6 (ARHGAP6), transcript variant 1, mRNA [NM_013427]	NM_013427
ARHGEF9	2,04	Homo sapiens Cdc42 guanine nucleotide exchange factor (GEF) 9 (ARHGEF9), transcript variant 1, mRNA [NM_015185]	NM_015185

ARID3B	-1,55	Homo sapiens AT rich interactive domain 3B (BRIGHT-like) (ARID3B), mRNA [NM_006465]	NM_006465
ASCL2	1,61	Homo sapiens achaete-scute family bHLH transcription factor 2 (ASCL2), mRNA [NM_005170]	NM_005170
ASIP	1,87	Homo sapiens agouti signaling protein (ASIP), mRNA [NM_001672]	NM_001672
ASPRV1	1,57	Homo sapiens aspartic peptidase, retroviral-like 1 (ASPRV1), mRNA [NM_152792]	NM_152792
ATF1	1,60	Homo sapiens activating transcription factor 1 (ATF1), mRNA [NM_005171]	NM_005171
ATG5	-1,51	Homo sapiens autophagy related 5 (ATG5), transcript variant 1, mRNA [NM_004849]	NM_004849
ATP1A1-AS1	1,63	Homo sapiens ATP1A1 antisense RNA 1 (ATP1A1-AS1), transcript variant 3, long non-coding RNA [NR_024126]	NR_024126
ATP6V0A4	1,89	Homo sapiens ATPase, H+ transporting, lysosomal V0 subunit a4 (ATP6V0A4), transcript variant 1, mRNA [NM_020632]	NM_020632
ATXN1	1,73	Homo sapiens ataxin 1 (ATXN1), transcript variant 1, mRNA [NM_000332]	NM_000332
ATXN2	-2,14	ataxin 2 [Source:HGNC Symbol;Acc:HGNC:10555] [ENST00000392645]	
BACH1	-1,88	Homo sapiens BTB and CNC homology 1, basic leucine zipper transcription factor 1 (BACH1), transcript variant t, non-coding RNA [NR_027655]	NR_027655
BBS1	1,62	Bardet-Biedl syndrome 1 [Source:HGNC Symbol;Acc:HGNC:966] [ENST00000529955]	
BICC1	1,60	BicC family RNA binding protein 1 [Source:HGNC Symbol;Acc:HGNC:19351] [ENST00000263103]	
BICD2	2,26	Homo sapiens bicaudal D homolog 2 (Drosophila) (BICD2), transcript variant 2, mRNA [NM_015250]	NM_015250
BMP1	1,56	bone morphogenetic protein 1 [Source:HGNC Symbol;Acc:HGNC:1067] [ENST00000471755]	
BTBD17	-2,32	Homo sapiens BTB (POZ) domain containing 17 (BTBD17), mRNA [NM_001080466]	NM_001080466
BTN3A1	-1,65	Homo sapiens butyrophilin, subfamily 3, member A1 (BTN3A1), transcript variant 1, mRNA [NM_007048]	NM_007048
C11orf63	1,94	Homo sapiens chromosome 11 open reading frame 63 (C11orf63), transcript variant 2, mRNA [NM_199124]	NM_199124
C16orf62	1,50	Homo sapiens chromosome 16 open reading frame 62 (C16orf62), transcript variant 1, mRNA [NM_020314]	NM_020314
C17orf97	1,51	Homo sapiens chromosome 17 open reading frame 97 (C17orf97), mRNA [NM_001013672]	NM_001013672
C19orf68	1,74	Homo sapiens chromosome 19 open reading frame 68 (C19orf68), mRNA [NM_199341]	NM_199341
C1orf220	1,83	Homo sapiens chromosome 1 open reading frame 220 (C1orf220), long non-coding RNA [NR_033186]	NR_033186
C1orf229	-1,74	Homo sapiens chromosome 1 open reading frame 229 (C1orf229), mRNA [NM_207401]	NM_207401
C1QTNF1	-1,85	Homo sapiens C1q and tumor necrosis factor related protein 1 (C1QTNF1), transcript variant 4, mRNA [NM_198594]	NM_198594
C22orf46	1,55	Homo sapiens chromosome 22 open reading frame 46 (C22orf46), mRNA [NM_001142964]	NM_001142964
C2orf68	1,53	Homo sapiens chromosome 2 open reading frame 68 (C2orf68), mRNA [NM_001013649]	NM_001013649
C6orf164	1,62	Homo sapiens chromosome 6 open reading frame 164 (C6orf164), long non-coding RNA [NR_026784]	NR_026784
C7orf55	1,72	Homo sapiens chromosome 7 open reading frame 55 (C7orf55), transcript variant 1, mRNA [NM_197964]	NM_197964
C8orf31	-6,05	Homo sapiens chromosome 8 open reading frame 31 (C8orf31), transcript variant 1, mRNA [NM_173687]	NM_173687
C8orf48	1,57	Homo sapiens chromosome 8 open reading frame 48 (C8orf48), mRNA [NM_001007090]	NM_001007090
C9orf78	1,53	Homo sapiens chromosome 9 open reading frame 78 (C9orf78), mRNA [NM_016520]	NM_016520
CA2	1,53	Homo sapiens carbonic anhydrase II (CA2), transcript variant 1, mRNA [NM_000067]	NM_000067

CA9	-5,63	Homo sapiens carbonic anhydrase IX (CA9), mRNA [NM_001216]	NM_001216
CACNA1B	1,64	Homo sapiens calcium channel, voltage-dependent, N type, alpha 1B subunit (CACNA1B), transcript variant 2, mRNA [NM_001243812]	NM_001243812
CACNA2D1	1,58	Homo sapiens calcium channel, voltage-dependent, alpha 2/delta subunit 1 (CACNA2D1), transcript variant 1, mRNA [NM_000722]	NM_000722
CALML3-AS1	1,80	Homo sapiens CALML3 antisense RNA 1 (CALML3-AS1), transcript variant 1, long non-coding RNA [NR_120496]	NR_120496
CARD11	1,53	Homo sapiens cDNA FLJ39820 fis, clone SPLEN2010625. [AK097139]	
CARD9	1,52	Homo sapiens caspase recruitment domain family, member 9 (CARD9), transcript variant 2, mRNA [NM_052814]	NM_052814
CASC2	2,43	Homo sapiens cancer susceptibility candidate 2 (non-protein coding) (CASC2), transcript variant 1, long non-coding RNA [NR_026939]	NR_026939
CASP7	2,23	Homo sapiens caspase 7, apoptosis-related cysteine peptidase (CASP7), transcript variant d, mRNA [NM_033338]	NM_033338
CBLN3	1,60	Homo sapiens cerebellin 3 precursor (CBLN3), mRNA [NM_001039771]	NM_001039771
CCDC103	2,17	Homo sapiens coiled-coil domain containing 103 (CCDC103), transcript variant 1, mRNA [NM_213607]	NM_213607
CCDC114	1,64	Homo sapiens coiled-coil domain containing 114 (CCDC114), mRNA [NM_144577]	NM_144577
CCDC149	1,58	Homo sapiens coiled-coil domain containing 149 (CCDC149), transcript variant 1, mRNA [NM_173463]	NM_173463
CCDC181	1,65	Homo sapiens coiled-coil domain containing 181 (CCDC181), transcript variant 2, mRNA [NM_021179]	NM_021179
CCDC7	-2,20	Homo sapiens coiled-coil domain containing 7 (CCDC7), transcript variant 1, mRNA [NM_145023]	NM_145023
CCDC71	-2,01	Homo sapiens coiled-coil domain containing 71 (CCDC71), mRNA [NM_022903]	NM_022903
CCDC87	1,82	Homo sapiens coiled-coil domain containing 87 (CCDC87), mRNA [NM_018219]	NM_018219
CCL1	-1,63	Homo sapiens chemokine (C-C motif) ligand 1 (CCL1), mRNA [NM_002981]	NM_002981
CCNA1	-1,65	Homo sapiens cyclin A1 (CCNA1), transcript variant 1, mRNA [NM_003914]	NM_003914
CCND1	-1,73	Homo sapiens cyclin D1 (CCND1), mRNA [NM_053056]	NM_053056
CD164	1,67	Homo sapiens CD164 molecule, sialomucin (CD164), transcript variant 1, mRNA [NM_006016]	NM_006016
CD2	-2,14	Homo sapiens CD2 molecule (CD2), mRNA [NM_001767]	NM_001767
CDIPT-AS1	-1,55	Homo sapiens CDIPT antisense RNA 1 (head to head) (CDIPT-AS1), transcript variant 1, long non-coding RNA [NR_015396]	NR_015396
CDK14	1,52	Homo sapiens cyclin-dependent kinase 14 (CDK14), transcript variant 2, mRNA [NM_012395]	NM_012395
CDK16	1,81	Homo sapiens cyclin-dependent kinase 16 (CDK16), transcript variant 1, mRNA [NM_006201]	NM_006201
CDR2L	1,58	Homo sapiens cerebellar degeneration-related protein 2-like (CDR2L), mRNA [NM_014603]	NM_014603
CDRT1	1,70	Homo sapiens CMT1A duplicated region transcript 1 (CDRT1), transcript variant 1, mRNA [NM_006382]	NM_006382
CEBPA-AS1	1,84	Homo sapiens CEBPA antisense RNA 1 (head to head) (CEBPA-AS1), long non-coding RNA [NR_026887]	NR_026887
CENPBD1	1,60	Homo sapiens CENPB DNA-binding domains containing 1 (CENPBD1), mRNA [NM_145039]	NM_145039
CENPBD1P1	1,55	Homo sapiens CENPBD1 pseudogene 1 (CENPBD1P1), non-coding RNA [NR_026052]	NR_026052
CFAP36	1,57	Homo sapiens cilia and flagella associated protein 36 (CFAP36), transcript variant 2, mRNA [NM_080667]	NM_080667
CFAP53	2,34	Homo sapiens cilia and flagella associated protein 53 (CFAP53), mRNA [NM_145020]	NM_145020

CHMP4B	-1,81	Homo sapiens charged multivesicular body protein 4B (CHMP4B), mRNA [NM_176812]	NM_176812
CHP2	1,51	Homo sapiens calcineurin-like EF-hand protein 2 (CHP2), mRNA [NM_022097]	NM_022097
CHST14	2,18	Homo sapiens carbohydrate (N-acetylgalactosamine 4-0) sulfotransferase 14 (CHST14), mRNA [NM_130468]	NM_130468
CHSY3	1,62	Homo sapiens chondroitin sulfate synthase 3 (CHSY3), mRNA [NM_175856]	NM_175856
CHTOP	1,79	chromatin target of PRMT1 [Source:HGNC Symbol;Acc:HGNC:24511] [ENST00000368686]	
CIDECP	1,74	cell death-inducing DFFA-like effector c pseudogene [Source:HGNC Symbol;Acc:HGNC:24230] [ENST00000335507]	
CLCC1	-1,52	Homo sapiens chloride channel CLIC-like 1 (CLCC1), transcript variant 1, mRNA [NM_001048210]	NM_001048210
CLMN	1,74	Homo sapiens calmin (calponin-like, transmembrane) (CLMN), mRNA [NM_024734]	NM_024734
CNNM3	-1,52	Homo sapiens cyclin and CBS domain divalent metal cation transport mediator 3 (CNNM3), transcript variant 1, mRNA [NM_017623]	NM_017623
CNOT4	-1,62	Homo sapiens CCR4-NOT transcription complex, subunit 4 (CNOT4), transcript variant 4, mRNA [NM_001190848]	NM_001190848
CNTLN	1,96	Homo sapiens centlein, centrosomal protein (CNTLN), transcript variant 1, mRNA [NM_017738]	NM_017738
CNTNAP3B	-2,17	Homo sapiens contactin associated protein-like 3B (CNTNAP3B), mRNA [NM_001201380]	NM_001201380
COL21A1	-1,73	Homo sapiens collagen, type XXI, alpha 1 (COL21A1), mRNA [NM_030820]	NM_030820
COL5A2	1,69	Homo sapiens collagen, type V, alpha 2 (COL5A2), mRNA [NM_000393]	NM_000393
COLEC11	1,73	Homo sapiens collectin sub-family member 11 (COLEC11), transcript variant 2, mRNA [NM_199235]	NM_199235
CP	1,64	Homo sapiens ceruloplasmin (ferroxidase) (CP), transcript variant 1, mRNA [NM_000096]	NM_000096
CPEB2	1,64	Homo sapiens cytoplasmic polyadenylation element binding protein 2 (CPEB2), transcript variant B, mRNA [NM_182485]	NM_182485
CPS1-IT1	-1,51	Homo sapiens CPS1 intronic transcript 1 (non-protein coding) (CPS1-IT1), long non-coding RNA [NR_002763]	NR_002763
CSK	1,75	Homo sapiens c-src tyrosine kinase (CSK), transcript variant 1, mRNA [NM_004383]	NM_004383
CXCL1	1,65	Homo sapiens chemokine (C-X-C motif) ligand 1 (melanoma growth stimulating activity, alpha) (CXCL1), transcript variant 1, mRNA [NM_001511]	NM_001511
CXCL12	-1,79	Homo sapiens chemokine (C-X-C motif) ligand 12 (CXCL12), transcript variant 2, mRNA [NM_000609]	NM_000609
CYFIP2	-1,52	Homo sapiens cytoplasmic FMR1 interacting protein 2 (CYFIP2), transcript variant 3, mRNA [NM_014376]	NM_014376
CYP2B6	1,85	Homo sapiens cytochrome P450, family 2, subfamily B, polypeptide 6 (CYP2B6), mRNA [NM_000767]	NM_000767
DAND5	1,87	Homo sapiens DAN domain family member 5, BMP antagonist (DAND5), mRNA [NM_152654]	NM_152654
DBF4	-1,67	Homo sapiens DBF4 zinc finger (DBF4), mRNA [NM_006716]	NM_006716
DCBLD1	2,03	Homo sapiens discoidin, CUB and LCCL domain containing 1 (DCBLD1), mRNA [NM_173674]	NM_173674
DCBLD2	1,94	Homo sapiens discoidin, CUB and LCCL domain containing 2 (DCBLD2), mRNA [NM_080927]	NM_080927
DCLK1	-1,95	Homo sapiens doublecortin-like kinase 1 (DCLK1), transcript variant 1, mRNA [NM_004734]	NM_004734
DCP2	1,59	Homo sapiens decapping mRNA 2 (DCP2), transcript variant 1, mRNA [NM_152624]	NM_152624
DDHD1	1,57	Homo sapiens DDHD domain containing 1 (DDHD1), transcript variant 1, mRNA [NM_030637]	NM_030637
DDIT4L	-2,09	Homo sapiens DNA-damage-inducible transcript 4-like (DDIT4L), mRNA [NM_145244]	NM_145244

DDX60	1,54	Homo sapiens DEAD (Asp-Glu-Ala-Asp) box polypeptide 60 (DDX60), mRNA [NM_017631]	NM_017631
DENND4A	1,73	Homo sapiens DENN/MADD domain containing 4A (DENND4A), transcript variant 1, mRNA [NM_001144823]	NM_001144823
DENND5A	1,69	Homo sapiens DENN/MADD domain containing 5A (DENND5A), transcript variant 1, mRNA [NM_015213]	NM_015213
DESI2	-1,71	Homo sapiens desumoylating isopeptidase 2 (DESI2), transcript variant 1, mRNA [NM_016076]	NM_016076
DGCR5	1,69	Homo sapiens DiGeorge syndrome critical region gene 5 (non-protein coding) (DGCR5), transcript variant 1, non-coding RNA [NR_002733]	NR_002733
DHRS3	-1,53	Homo sapiens dehydrogenase/reductase (SDR family) member 3 (DHRS3), mRNA [NM_004753]	NM_004753
DHX57	-1,73	Homo sapiens DEAH (Asp-Glu-Ala-Asp/His) box polypeptide 57 (DHX57), mRNA [NM_198963]	NM_198963
DIO3	1,52	Homo sapiens deiodinase, iodothyronine, type III (DIO3), mRNA [NM_001362]	NM_001362
DIRC3	2,81	Homo sapiens disrupted in renal carcinoma 3 (DIRC3), long non-coding RNA [NR_026597]	NR_026597
DKFZP434I0714	1,79	Homo sapiens uncharacterized protein DKFZP434I0714 (DKFZP434I0714), long non-coding RNA [NR_033797]	NR_033797
DKFZp434J0226	-9,72	Homo sapiens uncharacterized LOC93429 (DKFZp434J0226), long non-coding RNA [NR_027003]	NR_027003
DLST	-2,17	Homo sapiens dihydrolipoamide S-succinyltransferase (E2 component of 2-oxo-glutarate complex) (DLST), transcript variant 1, mRNA [NM_001933]	NM_001933
DNAJC7	-1,90	Homo sapiens DnaJ (Hsp40) homolog, subfamily C, member 7 (DNAJC7), transcript variant 1, mRNA [NM_003315]	NM_003315
DNHD1	1,52	Homo sapiens dynein heavy chain domain 1 (DNHD1), transcript variant 2, mRNA [NM_173589]	NM_173589
DOCK10	1,59	Homo sapiens dedicator of cytokinesis 10 (DOCK10), transcript variant DOCK10.1, mRNA [NM_014689]	NM_014689
DOK5	1,90	Homo sapiens docking protein 5 (DOK5), transcript variant 1, mRNA [NM_018431]	NM_018431
DOK7	1,59	Homo sapiens docking protein 7 (DOK7), transcript variant 4, mRNA [NM_001301071]	NM_001301071
DOT1L	1,52	Homo sapiens DOT1-like histone H3K79 methyltransferase (DOT1L), mRNA [NM_032482]	NM_032482
DRAXIN	-2,15	Homo sapiens dorsal inhibitory axon guidance protein (DRAXIN), mRNA [NM_198545]	NM_198545
DSCR8	1,50	Homo sapiens Down syndrome critical region 8 (DSCR8), transcript variant 4, long non-coding RNA [NR_026838]	NR_026838
DUSP19	2,40	Homo sapiens dual specificity phosphatase 19 (DUSP19), transcript variant 1, mRNA [NM_080876]	NM_080876
DUSP26	1,68	Homo sapiens dual specificity phosphatase 26 (putative) (DUSP26), mRNA [NM_024025]	NM_024025
DUSP5	1,66	Homo sapiens dual specificity phosphatase 5 (DUSP5), mRNA [NM_004419]	NM_004419
DUX4	1,58	Homo sapiens double homeobox 4 (DUX4), mRNA [NM_001293798]	NM_001293798
DYNC1LI2	-1,63	Homo sapiens dynein, cytoplasmic 1, light intermediate chain 2 (DYNC1LI2), transcript variant 1, mRNA [NM_006141]	NM_006141
E2F5	-1,52	Homo sapiens E2F transcription factor 5, p130-binding (E2F5), transcript variant 1, mRNA [NM_001951]	NM_001951
EBF1	-2,01	Homo sapiens early B-cell factor 1 (EBF1), transcript variant 1, mRNA [NM_001290360]	NM_001290360
ECEL1P2	-1,52	Homo sapiens endothelin converting enzyme-like 1, pseudogene 2 (ECEL1P2), non-coding RNA [NR_028501]	NR_028501
EFR3B	1,51	Homo sapiens EFR3 homolog B (S. cerevisiae) (EFR3B), mRNA [NM_014971]	NM_014971
EFTUD1	1,77	Homo sapiens elongation factor Tu GTP binding domain containing 1 (EFTUD1), transcript variant 1, mRNA [NM_024580]	NM_024580
EGR1	1,80	Homo sapiens early growth response 1 (EGR1), mRNA [NM_001964]	NM_001964

EGR4	2,18	Homo sapiens early growth response 4 (EGR4), mRNA [NM_001965]	NM_001965
EHHADH	-1,56	Homo sapiens enoyl-CoA, hydratase/3-hydroxyacyl CoA dehydrogenase (EHHADH), transcript variant 1, mRNA [NM_001966]	NM_001966
ELFN2	1,58	Homo sapiens extracellular leucine-rich repeat and fibronectin type III domain containing 2 (ELFN2), transcript variant 1, mRNA [NM_052906]	NM_052906
ELK1	2,15	Homo sapiens ELK1, member of ETS oncogene family (ELK1), transcript variant 3, mRNA [NM_001257168]	NM_001257168
EMC6	2,33	Homo sapiens ER membrane protein complex subunit 6 (EMC6), transcript variant 1, mRNA [NM_001014764]	NM_001014764
EMD	-1,92	Homo sapiens emerin (EMD), mRNA [NM_000117]	NM_000117
ENAH	1,56	Homo sapiens enabled homolog (Drosophila) (ENAH), transcript variant 1, mRNA [NM_001008493]	NM_001008493
ENKUR	1,62	Homo sapiens enkurin, TRPC channel interacting protein (ENKUR), transcript variant 1, mRNA [NM_145010]	NM_145010
EOGT	-2,00	Homo sapiens EGF domain-specific O-linked N-acetylglucosamine (GlcNAc) transferase (EOGT), transcript variant 2, mRNA [NM_173654]	NM_173654
EPHA1	1,62	Homo sapiens EPH receptor A1 (EPHA1), mRNA [NM_005232]	NM_005232
ERC2	1,52	Homo sapiens ELKS/RAB6-interacting/CAST family member 2 (ERC2), mRNA [NM_015576]	NM_015576
ERICH5	1,88	Homo sapiens glutamate-rich 5 (ERICH5), transcript variant 1, mRNA [NM_173549]	NM_173549
ESPNL	-1,52	Homo sapiens espin-like (ESPNL), mRNA [NM_194312]	NM_194312
ESR1	-1,52	Homo sapiens estrogen receptor 1 (ESR1), transcript variant 1, mRNA [NM_000125]	NM_000125
ETV3	2,03	Homo sapiens ets variant 3 (ETV3), transcript variant 1, mRNA [NM_001145312]	NM_001145312
ETV5	1,80	Homo sapiens ets variant 5 (ETV5), mRNA [NM_004454]	NM_004454
EVI2A	-4,17	Homo sapiens ecotropic viral integration site 2A (EVI2A), transcript variant 1, mRNA [NM_001003927]	NM_001003927
EXOC3-AS1	1,55	Homo sapiens EXOC3 antisense RNA 1 (EXOC3-AS1), long non-coding RNA [NR_126522]	NR_126522
FABP7	-2,97	Homo sapiens fatty acid binding protein 7, brain (FABP7), mRNA [NM_001446]	NM_001446
FAM110B	1,67	Homo sapiens family with sequence similarity 110, member B (FAM110B), mRNA [NM_147189]	NM_147189
FAM134C	-1,76	Homo sapiens family with sequence similarity 134, member C (FAM134C), transcript variant 1, mRNA [NM_178126]	NM_178126
FAM173B	1,67	Homo sapiens family with sequence similarity 173, member B (FAM173B), transcript variant 1, mRNA [NM_199133]	NM_199133
FAM19A4	1,95	Homo sapiens family with sequence similarity 19 (chemokine (C-C motif)-like), member A4 (FAM19A4), transcript variant 1, mRNA [NM_182522]	NM_182522
FAM201A	1,69	Homo sapiens family with sequence similarity 201, member A (FAM201A), long non-coding RNA [NR_027294]	NR_027294
FAM208A	1,64	Homo sapiens family with sequence similarity 208, member A (FAM208A), transcript variant 2, mRNA [NM_015224]	NM_015224
FAM217B	1,52	Homo sapiens family with sequence similarity 217, member B (FAM217B), transcript variant 2, mRNA [NM_001190826]	NM_001190826
FAM226A	1,88	Homo sapiens family with sequence similarity 226, member A (non-protein coding) (FAM226A), long non-coding RNA [NR_026595]	NR_026595
FAM230A	1,87	PREDICTED: Homo sapiens family with sequence similarity 230, member A (FAM230A), mRNA [XM_006726832]	XM_006726832
FAM35A	1,80	Homo sapiens family with sequence similarity 35, member A (FAM35A), mRNA [NM_019054]	NM_019054

FAM83A	1,51	Homo sapiens family with sequence similarity 83, member A (FAM83A), transcript variant 1, mRNA [NM_032899]	NM_032899
FAM95B1	-3,42	Homo sapiens family with sequence similarity 95, member B1 (FAM95B1), long non-coding RNA [NR_026759]	NR_026759
FBF1	1,61	Homo sapiens Fas (TNFRSF6) binding factor 1 (FBF1), mRNA [NM_001080542]	NM_001080542
FCHSD2	1,58	Homo sapiens FCH and double SH3 domains 2 (FCHSD2), mRNA [NM_014824]	NM_014824
FER1L5	1,66	Homo sapiens fer-1-like family member 5 (FER1L5), mRNA [NM_001293083]	NM_001293083
FGD2	-1,81	Homo sapiens FYVE, RhoGEF and PH domain containing 2 (FGD2), mRNA [NM_173558]	NM_173558
FGD5	1,56	Homo sapiens FYVE, RhoGEF and PH domain containing 5 (FGD5), mRNA [NM_152536]	NM_152536
FGFR3	2,04	Homo sapiens fibroblast growth factor receptor 3 (FGFR3), transcript variant 1, mRNA [NM_000142]	NM_000142
FHDC1	1,54	Homo sapiens FH2 domain containing 1 (FHDC1), mRNA [NM_033393]	NM_033393
FLJ16171	1,58	Homo sapiens FLJ16171 protein (FLJ16171), long non-coding RNA [NR_046113]	NR_046113
FLJ22447	1,68	Homo sapiens uncharacterized LOC400221 (FLJ22447), long non-coding RNA [NR_039985]	NR_039985
FLJ44511	1,53	Homo sapiens uncharacterized LOC441307 (FLJ44511), long non-coding RNA [NR_033963]	NR_033963
FLJ45950	1,78	Homo sapiens cDNA FLJ45950 fis, clone PLACE7008136. [AK127847]	
FNBP1	1,89	PREDICTED: Homo sapiens formin binding protein 1 (FNBP1), transcript variant X1, mRNA [XM_005251815]	XM_005251815
FNDC4	-1,80	Homo sapiens fibronectin type III domain containing 4 (FNDC4), mRNA [NM_022823]	NM_022823
FOPNL	1,77	Homo sapiens FGFR1OP N-terminal like (FOPNL), mRNA [NM_144600]	NM_144600
FOXK2	1,63	Homo sapiens forkhead box K2 (FOXK2), mRNA [NM_004514]	NM_004514
FOXN2	-1,57	Homo sapiens forkhead box N2 (FOXN2), mRNA [NM_002158]	NM_002158
FOXN3	1,51	Homo sapiens forkhead box N3 (FOXN3), transcript variant 2, mRNA [NM_005197]	NM_005197
FOXP2	1,91	Homo sapiens forkhead box P2 (FOXP2), transcript variant 1, mRNA [NM_014491]	NM_014491
FOXP4	1,86	Homo sapiens forkhead box P4 (FOXP4), transcript variant 1, mRNA [NM_001012426]	NM_001012426
FPR1	-1,51	Homo sapiens formyl peptide receptor 1 (FPR1), transcript variant 2, mRNA [NM_002029]	NM_002029
FPR3	-1,58	Homo sapiens formyl peptide receptor 3 (FPR3), mRNA [NM_002030]	NM_002030
FRAT2	1,56	Homo sapiens frequently rearranged in advanced T-cell lymphomas 2 (FRAT2), mRNA [NM_012083]	NM_012083
FSD1L	2,39	Homo sapiens fibronectin type III and SPRY domain containing 1-like (FSD1L), transcript variant 3, mRNA [NM_001145313]	NM_001145313
FUT11	-1,55	Homo sapiens fucosyltransferase 11 (alpha (1,3) fucosyltransferase) (FUT11), transcript variant 2, mRNA [NM_001284194]	NM_001284194
FUT5	-1,90	Homo sapiens fucosyltransferase 5 (alpha (1,3) fucosyltransferase) (FUT5), mRNA [NM_002034]	NM_002034
FXYD2	1,77	Homo sapiens FXYD domain containing ion transport regulator 2 (FXYD2), transcript variant a, mRNA [NM_001680]	NM_001680
FZD10	-23,81	Homo sapiens frizzled class receptor 10 (FZD10), mRNA [NM_007197]	NM_007197
G3BP2	2,57	Homo sapiens GTPase activating protein (SH3 domain) binding protein 2 (G3BP2), transcript variant 1, mRNA [NM_203505]	NM_203505
GAB1	1,54	Homo sapiens GRB2-associated binding protein 1 (GAB1), transcript variant 1, mRNA [NM_207123]	NM_207123
GAD1	2,28	Homo sapiens glutamate decarboxylase 1 (brain, 67kDa) (GAD1), transcript variant GAD67, mRNA [NM_000817]	NM_000817

GANAB	1,83	Homo sapiens glucosidase, alpha; neutral AB (GANAB), transcript variant 3, mRNA [NM_198335]	NM_198335
GATA3-AS1	1,55	Homo sapiens GATA3 antisense RNA 1 (GATA3-AS1), transcript variant 2, long non-coding RNA [NR_024256]	NR_024256
GATA4	-1,79	Homo sapiens GATA binding protein 4 (GATA4), mRNA [NM_002052]	NM_002052
GBA3	-2,20	Homo sapiens glucosidase, beta, acid 3 (gene/pseudogene) (GBA3), transcript variant 1, mRNA [NM_020973]	NM_020973
GJD3	2,51	Homo sapiens gap junction protein, delta 3, 31.9kDa (GJD3), mRNA [NM_152219]	NM_152219
GK5	1,63	Homo sapiens glycerol kinase 5 (putative) (GK5), transcript variant 1, mRNA [NM_001039547]	NM_001039547
GLP2R	-1,55	Homo sapiens glucagon-like peptide 2 receptor (GLP2R), mRNA [NM_004246]	NM_004246
GLTSCR1	1,98	Homo sapiens glioma tumor suppressor candidate region gene 1 (GLTSCR1), mRNA [NM_015711]	NM_015711
GLYCTK	1,64	Homo sapiens glycerate kinase (GLYCTK), transcript variant 1, mRNA [NM_145262]	NM_145262
GML	-1,54	Homo sapiens glycosylphosphatidylinositol anchored molecule like (GML), mRNA [NM_002066]	NM_002066
GNA11	1,53	Homo sapiens guanine nucleotide binding protein (G protein), alpha 11 (Gq class) (GNA11), mRNA [NM_002067]	NM_002067
GNAT2	1,77	Homo sapiens guanine nucleotide binding protein (G protein), alpha transducing activity polypeptide 2 (GNAT2), mRNA [NM_005272]	NM_005272
GOLGA2	1,56	Homo sapiens golgin A2 (GOLGA2), mRNA [NM_004486]	NM_004486
GOLGA6L7P	-1,68	golgin A6 family-like 7, pseudogene [Source:HGNC Symbol;Acc:HGNC:37442] [ENST00000567390]	XM_006726562
GOLGA8A	1,67	Homo sapiens golgin A8 family, member A (GOLGA8A), transcript variant 1, mRNA [NM_181077]	NM_181077
GP6	-1,69	Homo sapiens glycoprotein VI (platelet) (GP6), transcript variant 1, mRNA [NM_001083899]	NM_001083899
GPHA2	-1,78	Homo sapiens glycoprotein hormone alpha 2 (GPHA2), mRNA [NM_130769]	NM_130769
GPR113	-1,78	Homo sapiens G protein-coupled receptor 113 (GPR113), transcript variant 3, mRNA [NM_153835]	NM_153835
GPR137C	-1,54	Homo sapiens G protein-coupled receptor 137C (GPR137C), mRNA [NM_001099652]	NM_001099652
GREB1	-1,54	Homo sapiens GREB1 protein, mRNA (cDNA clone IMAGE:6729261), partial cds. [BC071853]	
GXYLT1	1,55	Homo sapiens glucoside xylosyltransferase 1 (GXYLT1), transcript variant 1, mRNA [NM_173601]	NM_173601
HABP4	2,25	Homo sapiens hyaluronan binding protein 4 (HABP4), mRNA [NM_014282]	NM_014282
HDHD1	-2,06	Homo sapiens haloacid dehalogenase-like hydrolase domain containing 1 (HDHD1), transcript variant 2, mRNA [NM_012080]	NM_012080
HEG1	-1,56	Homo sapiens heart development protein with EGF-like domains 1 (HEG1), mRNA [NM_020733]	NM_020733
HERC2	1,61	Homo sapiens HECT and RLD domain containing E3 ubiquitin protein ligase 2 (HERC2), mRNA [NM_004667]	NM_004667
HEXIM1	1,58	Homo sapiens hexamethylene bis-acetamide inducible 1 (HEXIM1), mRNA [NM_006460]	NM_006460
HHLA1	-1,93	Homo sapiens HERV-H LTR-associating 1 (HHLA1), mRNA [NM_001145095]	NM_001145095
HIST4H4	2,63	Homo sapiens histone cluster 4, H4 (HIST4H4), mRNA [NM_175054]	NM_175054
HMBOX1	1,55	Homo sapiens homeobox containing 1 (HMBOX1), transcript variant 1, mRNA [NM_024567]	NM_024567
HMCN1	1,51	Homo sapiens hemicentin 1 (HMCN1), mRNA [NM_031935]	NM_031935
HNRNPC	-1,67	Homo sapiens heterogeneous nuclear ribonucleoprotein C (C1/C2) (HNRNPC), transcript variant 1, mRNA [NM_031314]	NM_031314

HORMAD1	-2,49	Homo sapiens HORMA domain containing 1 (HORMAD1), transcript variant 1, mRNA [NM_032132]	NM_032132
HOTAIR	1,55	Homo sapiens HOX transcript antisense RNA (HOTAIR), transcript variant 3, long non-coding RNA [NR_047518]	NR_047518
HOXB2	1,55	Homo sapiens homeobox B2 (HOXB2), mRNA [NM_002145]	NM_002145
HRASLS2	2,31	Homo sapiens HRAS-like suppressor 2 (HRASLS2), mRNA [NM_017878]	NM_017878
HRG	-1,63	Homo sapiens histidine-rich glycoprotein (HRG), mRNA [NM_000412]	NM_000412
HS2ST1	1,54	Homo sapiens heparan sulfate 2-O-sulfotransferase 1 (HS2ST1), transcript variant 2, mRNA [NM_001134492]	NM_001134492
HS3ST3B1	1,62	Homo sapiens heparan sulfate (glucosamine) 3-O-sulfotransferase 3B1 (HS3ST3B1), transcript variant 1, mRNA [NM_006041]	NM_006041
HSD3B2	1,77	Homo sapiens hydroxy-delta-5-steroid dehydrogenase, 3 beta- and steroid delta-isomerase 2 (HSD3B2), transcript variant 1, mRNA [NM_000198]	NM_000198
HSDL2	1,95	Homo sapiens hydroxysteroid dehydrogenase like 2 (HSDL2), transcript variant 1, mRNA [NM_032303]	NM_032303
HSPA6	-1,80	Homo sapiens heat shock 70kDa protein 6 (HSP70B') (HSPA6), mRNA [NM_002155]	NM_002155
HYMAI	1,52	Homo sapiens hydatidiform mole associated and imprinted (non-protein coding) (HYMAI), long non-coding RNA [NR_002768]	NR_002768
ICAM4	-1,65	Homo sapiens intercellular adhesion molecule 4 (Landsteiner-Wiener blood group) (ICAM4), transcript variant 2, mRNA [NM_022377]	NM_022377
IER2	1,79	Homo sapiens immediate early response 2 (IER2), mRNA [NM_004907]	NM_004907
IFIT1	1,73	Homo sapiens interferon-induced protein with tetratricopeptide repeats 1 (IFIT1), transcript variant 1, mRNA [NM_001548]	NM_001548
IFIT2	1,68	Homo sapiens interferon-induced protein with tetratricopeptide repeats 2 (IFIT2), mRNA [NM_001547]	NM_001547
IFT27	1,89	Homo sapiens intraflagellar transport 27 (IFT27), transcript variant 1, mRNA [NM_001177701]	NM_001177701
IGDCC4	1,96	Homo sapiens immunoglobulin superfamily, DCC subclass, member 4 (IGDCC4), mRNA [NM_020962]	NM_020962
IGLL1	1,57	Homo sapiens immunoglobulin lambda-like polypeptide 1 (IGLL1), transcript variant 1, mRNA [NM_020070]	NM_020070
IKZF4	1,51	Homo sapiens IKAROS family zinc finger 4 (Eos) (IKZF4), mRNA [NM_022465]	NM_022465
IL1RL2	1,63	Homo sapiens interleukin 1 receptor-like 2 (IL1RL2), mRNA [NM_003854]	NM_003854
IL24	-1,92	Homo sapiens interleukin 24 (IL24), transcript variant 3, mRNA [NM_001185156]	NM_001185156
INPP5A	1,82	Homo sapiens inositol polyphosphate-5-phosphatase, 40kDa (INPP5A), mRNA [NM_005539]	NM_005539
INTS5	1,51	Homo sapiens integrator complex subunit 5 (INTS5), mRNA [NM_030628]	NM_030628
IPO7	-1,51	Homo sapiens importin 7 (IPO7), mRNA [NM_006391]	NM_006391
IQCD	1,92	Homo sapiens cDNA FLJ45209 fis, clone BRCAN2015757. [AK127152]	
ITPK1	-1,88	Homo sapiens inositol-tetrakisphosphate 1-kinase (ITPK1), transcript variant 1, mRNA [NM_014216]	NM_014216
ITPR2	1,53	Homo sapiens inositol 1,4,5-trisphosphate receptor, type 2 (ITPR2), mRNA [NM_002223]	NM_002223
ITSN2	1,63	Homo sapiens intersectin 2 (ITSN2), transcript variant 2, mRNA [NM_147152]	NM_147152
JAG1	-3,09	Homo sapiens jagged 1 (JAG1), mRNA [NM_000214]	NM_000214

KALRN	-1,57	Homo sapiens kalirin, RhoGEF kinase (KALRN), transcript variant 1, mRNA [NM_001024660]	NM_001024660
KALRN	1,90	Homo sapiens kalirin, RhoGEF kinase (KALRN), transcript variant 2, mRNA [NM_003947]	NM_003947
KANK4	1,76	Homo sapiens KN motif and ankyrin repeat domains 4 (KANK4), mRNA [NM_181712]	NM_181712
KAZN	2,08	PREDICTED: Homo sapiens kazrin, periplakin interacting protein (KAZN), transcript variant X2, mRNA [XM_005245796]	XM_005245796
KCNB1	2,30	Homo sapiens potassium channel, voltage gated Shab related subfamily B, member 1 (KCNB1), mRNA [NM_004975]	NM_004975
KCNC1	-2,06	Homo sapiens potassium channel, voltage gated Shaw related subfamily C, member 1 (KCNC1), transcript variant 2, mRNA [NM_004976]	NM_004976
KCNC4	1,50	Homo sapiens potassium channel, voltage gated Shaw related subfamily C, member 4 (KCNC4), transcript variant 3, mRNA [NM_001039574]	NM_001039574
KCNG3	-2,57	Homo sapiens potassium channel, voltage gated modifier subfamily G, member 3 (KCNG3), transcript variant 1, mRNA [NM_133329]	NM_133329
KCNH2	1,54	Homo sapiens potassium voltage-gated channel, subfamily H (eag-related), member 2 (KCNH2), transcript variant 2, mRNA [NM_172056]	NM_172056
KCNIP2-AS1	1,52	Homo sapiens KCNIP2 antisense RNA 1 (KCNIP2-AS1), long non-coding RNA [NR_045118]	NR_045118
KCNJ13	-1,76	Homo sapiens potassium channel, inwardly rectifying subfamily J, member 13 (KCNJ13), transcript variant 1, mRNA [NM_002242]	NM_002242
KCNJ2	1,53	Homo sapiens potassium channel, inwardly rectifying subfamily J, member 2 (KCNJ2), mRNA [NM_000891]	NM_000891
KCNJ3	1,54	Homo sapiens potassium channel, inwardly rectifying subfamily J, member 3 (KCNJ3), transcript variant 1, mRNA [NM_002239]	NM_002239
KCNK3	-1,53	Homo sapiens potassium channel, two pore domain subfamily K, member 3 (KCNK3), mRNA [NM_002246]	NM_002246
KCNK4	1,77	Homo sapiens potassium channel, two pore domain subfamily K, member 4 (KCNK4), mRNA [NM_033310]	NM_033310
KCNK5	-1,58	Homo sapiens potassium channel, two pore domain subfamily K, member 5 (KCNK5), mRNA [NM_003740]	NM_003740
KCNQ1	2,07	Homo sapiens potassium voltage-gated channel, KQT-like subfamily, member 1 (KCNQ1), transcript variant 1, mRNA [NM_000218]	NM_000218
KCTD13	1,50	Homo sapiens potassium channel tetramerization domain containing 13 (KCTD13), transcript variant 1, mRNA [NM_178863]	NM_178863
KDELC2	1,60	Homo sapiens KDEL (Lys-Asp-Glu-Leu) containing 2 (KDELC2), mRNA [NM_153705]	NM_153705
KDM2B	2,20	Homo sapiens lysine (K)-specific demethylase 2B (KDM2B), transcript variant 1, mRNA [NM_032590]	NM_032590
KDM4B	1,84	Homo sapiens lysine (K)-specific demethylase 4B (KDM4B), mRNA [NM_015015]	NM_015015
KIAA0556	1,51	Homo sapiens KIAA0556 (KIAA0556), mRNA [NM_015202]	NM_015202
KIAA1217	1,62	Homo sapiens KIAA1217 (KIAA1217), transcript variant 1, mRNA [NM_019590]	NM_019590
KIAA1522	1,60	Homo sapiens KIAA1522 (KIAA1522), transcript variant 1, mRNA [NM_020888]	NM_020888
KIAA1614	-1,70	Homo sapiens KIAA1614 (KIAA1614), mRNA [NM_020950]	NM_020950
KIF13B	-1,52	Homo sapiens kinesin family member 13B (KIF13B), mRNA [NM_015254]	NM_015254
KIF1B	1,52	Homo sapiens kinesin family member 1B (KIF1B), transcript variant 2, mRNA [NM_183416]	NM_183416

KIF1C	1,50	Homo sapiens kinesin family member 1C (KIF1C), mRNA [NM_006612]	NM_006612
KIF3C	1,89	Homo sapiens kinesin family member 3C (KIF3C), mRNA [NM_002254]	NM_002254
KLHDC10	2,32	Homo sapiens kelch domain containing 10 (KLHDC10), mRNA [NM_014997]	NM_014997
KLHL2	-1,63	Homo sapiens kelch-like family member 2 (KLHL2), transcript variant 1, mRNA [NM_007246]	NM_007246
KLHL38	1,95	Homo sapiens kelch-like family member 38 (KLHL38), mRNA [NM_001081675]	NM_001081675
KLHL42	-1,52	Homo sapiens kelch-like family member 42 (KLHL42), mRNA [NM_020782]	NM_020782
KLHL6	-1,63	Homo sapiens kelch-like family member 6 (KLHL6), mRNA [NM_130446]	NM_130446
KLK10	1,74	Homo sapiens kallikrein-related peptidase 10 (KLK10), transcript variant 1, mRNA [NM_002776]	NM_002776
L1CAM	1,87	Homo sapiens L1 cell adhesion molecule (L1CAM), transcript variant 1, mRNA [NM_000425]	NM_000425
LAMA4	-1,63	Homo sapiens laminin, alpha 4 (LAMA4), transcript variant 1, mRNA [NM_001105206]	NM_001105206
LAMC1	-1,59	Homo sapiens laminin, gamma 1 (formerly LAMB2) (LAMC1), mRNA [NM_002293]	NM_002293
LATS2	1,56	Homo sapiens large tumor suppressor kinase 2 (LATS2), mRNA [NM_014572]	NM_014572
LCE3E	-1,59	Homo sapiens late cornified envelope 3E (LCE3E), mRNA [NM_178435]	NM_178435
LCN12	-1,50	Homo sapiens lipocalin 12 (LCN12), mRNA [NM_178536]	NM_178536
LCOR	1,82	Homo sapiens ligand dependent nuclear receptor corepressor (LCOR), transcript variant 1, mRNA [NM_032440]	NM_032440
LDB3	-4,82	Homo sapiens LIM domain binding 3 (LDB3), transcript variant 5, mRNA [NM_001171610]	NM_001171610
LEKR1	1,51	Homo sapiens cDNA FLJ37161 fis, clone BRACE2026725. [AK094480]	
LEPR	1,65	Homo sapiens leptin receptor (LEPR), transcript variant 3, mRNA [NM_001003679]	NM_001003679
LHFPL3-AS2	2,04	Homo sapiens LHFPL3 antisense RNA 2 (LHFPL3-AS2), long non-coding RNA [NR_027374]	NR_027374
LINC00115	1,52	Homo sapiens long intergenic non-protein coding RNA 115 (LINC00115), long non-coding RNA [NR_024321]	NR_024321
LINC00173	1,55	Homo sapiens long intergenic non-protein coding RNA 173 (LINC00173), transcript variant 1, long non-coding RNA [NR_027345]	NR_027345
LINC00239	-2,20	Homo sapiens long intergenic non-protein coding RNA 239 (LINC00239), long non-coding RNA [NR_026774]	NR_026774
LINC00269	-1,52	Homo sapiens long intergenic non-protein coding RNA 269 (LINC00269), long non-coding RNA [NR_103715]	NR_103715
LINC00273	1,52	Homo sapiens long intergenic non-protein coding RNA 273 (LINC00273), long non-coding RNA [NR_038368]	NR_038368
LINC00337	1,63	Homo sapiens long intergenic non-protein coding RNA 337 (LINC00337), long non-coding RNA [NR_103534]	NR_103534
LINC00476	2,05	Homo sapiens long intergenic non-protein coding RNA 476 (LINC00476), transcript variant 1, long non-coding RNA [NR_023390]	NR_023390
LINC00597	-3,03	Homo sapiens long intergenic non-protein coding RNA 597 (LINC00597), long non-coding RNA [NR_026813]	NR_026813
LINC00663	1,67	Homo sapiens long intergenic non-protein coding RNA 663 (LINC00663), long non-coding RNA [NR_026956]	NR_026956
LINC00685	1,55	Homo sapiens long intergenic non-protein coding RNA 685 (LINC00685), transcript variant 1, long non-coding RNA [NR_027232]	NR_027232
LINC00965	1,73	Homo sapiens long intergenic non-protein coding RNA 965 (LINC00965), long non-coding RNA [NR_027000]	NR_027000

LINC01011	2,00	Homo sapiens long intergenic non-protein coding RNA 1011 (LINC01011), transcript variant 1, long non-coding RNA [NR_026855]	NR_026855
LINC01105	1,68	Homo sapiens long intergenic non-protein coding RNA 1105 (LINC01105), long non-coding RNA [NR_026832]	NR_026832
LINC01165	1,52	PREDICTED: Homo sapiens long intergenic non-protein coding RNA 1165 (LINC01165), misc_RNA [XR_242765]	XR_242765
LINC01183	1,63	long intergenic non-protein coding RNA 1183 [Source:HGNC Symbol;Acc:HGNC:49566] [ENST00000513708]	XR_159059
LINC01343	-4,34	Homo sapiens long intergenic non-protein coding RNA 1343 (LINC01343), long non-coding RNA [NR_038928]	NR_038928
LINC01431	1,61	Homo sapiens long intergenic non-protein coding RNA 1431 (LINC01431), long non-coding RNA [NR_109884]	NR_109884
LINC01560	1,74	Homo sapiens long intergenic non-protein coding RNA 1560 (LINC01560), long non-coding RNA [NR_126059]	NR_126059
LMNB1	-1,56	Homo sapiens lamin B1 (LMNB1), transcript variant 1, mRNA [NM_005573]	NM_005573
lnc-ABCC11-1	-2,75	zt99e08.r1 Soares_testis_NHT Homo sapiens cDNA clone IMAGE:730502 5', mRNA sequence [AA412392]	
lnc-ANP32A-3	1,59	Homo sapiens cDNA FLJ32231 fis, clone PLACE6004491. [AK056793]	
lnc-B4GALT3-1	1,54	LNCipedia lincRNA (lnc-B4GALT3-1), lincRNA [lnc-B4GALT3-1:1]	
lnc-BAI1-1	2,26	Homo sapiens hypothetical protein LOC286121, mRNA (cDNA clone IMAGE:4837614). [BC039302]	
lnc-C10orf71-1	1,96	17000600021474 GRN_PRENEU Homo sapiens cDNA 5', mRNA sequence [CN291113]	
lnc-EYS-2	1,53	Homo sapiens cDNA clone IMAGE:4830172. [BC040303]	
lnc-GOLGA8J-3	1,58	Homo sapiens, clone IMAGE:5221398, mRNA. [BC043570]	
lnc-ITGA2-1	1,85	LNCipedia lincRNA (lnc-ITGA2-1), lincRNA [lnc-ITGA2-1:5]	
lnc-MRPL14-1	1,62	Homo sapiens alpha-actinin-like mRNA, partial sequence. [DQ272581]	
lnc-RP11-712L6.5.1-2	1,64	Homo sapiens cDNA FLJ39051 fis, clone NT2RP7011452. [AK096370]	
LOC100049716	2,01	Homo sapiens uncharacterized LOC100049716 (LOC100049716), long non-coding RNA [NR_122124]	NR_122124
LOC100130152	1,64	Homo sapiens cDNA FLJ45869 fis, clone OCBBF3004908. [AK127768]	
LOC100130417	1,77	Homo sapiens uncharacterized LOC100130417 (LOC100130417), transcript variant 1, long non-coding RNA [NR_026874]	NR_026874
LOC100130587	-1,54	Homo sapiens uncharacterized LOC100130587 (LOC100130587), long non-coding RNA [NR_110634]	NR_110634
LOC100130865	1,56	Homo sapiens cDNA FLJ41135 fis, clone BRACE2028970. [AK123130]	
LOC100131000	1,50	Homo sapiens cDNA FLJ42370 fis, clone UTERU2030280. [AK124361]	
LOC100131195	-1,62	Homo sapiens cDNA FLJ40424 fis, clone TESTI2039026. [AK097743]	
LOC100131347	1,79	Homo sapiens RAD52 motif containing 1 pseudogene (LOC100131347), non-coding RNA [NR_036551]	NR_036551
LOC100132249	1,75	PREDICTED: Homo sapiens family with sequence similarity 201, member B (FAM201B), misc_RNA [XR_158980]	XR_158980
LOC100270804	1,59	Homo sapiens uncharacterized LOC100270804 (LOC100270804), long non-coding RNA [NR_026885]	NR_026885
LOC100288619	1,59	Homo sapiens cDNA FLJ27363 fis, clone UBA02185. [AK130873]	
LOC100289580	1,85	Homo sapiens uncharacterized LOC100289580 (LOC100289580), long non-coding RNA [NR_103774]	NR_103774
LOC101927202	1,52	PREDICTED: Homo sapiens uncharacterized LOC101927202 (LOC101927202), ncRNA [XR_244435]	XR_244435
LOC101927645	1,78	PREDICTED: Homo sapiens putative uncharacterized protein FLJ44967-like (LOC101927645), mRNA [XM_005275697]	XM_005275697

LOC101929023	-1,65	Homo sapiens uncharacterized LOC101929023 (LOC101929023), transcript variant 1, long non-coding RNA [NR_125972]	NR_125972
LOC102723652	1,81	long intergenic non-protein coding RNA 863 [Source:HGNC Symbol;Acc:HGNC:45162] [ENST00000439559]	XR_432607
LOC102725453	-1,68	PREDICTED: Homo sapiens uncharacterized LOC102725453 (LOC102725453), mRNA [XM_006710128]	XM_006710128
LOC143666	1,61	Homo sapiens uncharacterized LOC143666 (LOC143666), long non-coding RNA [NR_026967]	NR_026967
LOC257396	1,55	Homo sapiens uncharacterized LOC257396 (LOC257396), transcript variant 1, long non-coding RNA [NR_034107]	NR_034107
LOC285762	1,62	Homo sapiens uncharacterized LOC285762 (LOC285762), long non-coding RNA [NR_046100]	NR_046100
LOC286071	1,98	Homo sapiens cDNA FLJ34440 fis, clone HLUNG2001214. [AK091759]	
LOC339988	1,62	PREDICTED: Homo sapiens uncharacterized LOC339988 (RP11-539L10.2), misc_RNA [XR_249565]	XR_249565
LOC440896	1,79	Homo sapiens uncharacterized LOC440896 (LOC440896), long non-coding RNA [NR_015361]	NR_015361
LOC441666	1,68	Homo sapiens zinc finger protein 91 pseudogene (LOC441666), non-coding RNA [NR_024380]	NR_024380
LOC642236	-1,51	Homo sapiens FSHD region gene 1 pseudogene (LOC642236), non-coding RNA [NR_033907]	NR_033907
LOC643936	1,57	Homo sapiens cDNA FLJ45840 fis, clone NTONG2004829. [AK127740]	
LOC653602	-1,65	Homo sapiens uncharacterized LOC653602 (LOC653602), mRNA [NM_001291410]	NM_001291410
LOC728093	1,68	PREDICTED: Homo sapiens putative POM121-like protein 1-like (LOC728093), misc_RNA [XR_425912]	XR_425912
LOC728613	1,77	Homo sapiens programmed cell death 6 pseudogene (LOC728613), non-coding RNA [NR_003713]	NR_003713
LOC728903	1,53	Homo sapiens cDNA FLJ36403 fis, clone THYMU2009948. [AK093722]	
LOC729040	-1,61	PREDICTED: Homo sapiens uncharacterized LOC729040 (LOC729040), misc_RNA [XR_241838]	XR_241838
LOC729083	-1,53	Homo sapiens uncharacterized LOC729083 (LOC729083), long non-coding RNA [NR_122070]	NR_122070
LOC729970	-1,68	Homo sapiens hCG2028352-like (LOC729970), long non-coding RNA [NR_033998]	NR_033998
LOC91548	1,56	PREDICTED: Homo sapiens uncharacterized LOC91548 (RP11-122M14.1), misc_RNA [XR_247053]	XR_247053
LOH12CR2	1,66	Homo sapiens loss of heterozygosity, 12, chromosomal region 2 (non-protein coding) (LOH12CR2), long non-coding RNA [NR_024061]	NR_024061
LPAR6	-1,51	Homo sapiens lysophosphatidic acid receptor 6 (LPAR6), transcript variant 1, mRNA [NM_005767]	NM_005767
LPCAT2	-1,69	Homo sapiens lysophosphatidylcholine acyltransferase 2 (LPCAT2), mRNA [NM_017839]	NM_017839
LPP	1,55	Homo sapiens LIM domain containing preferred translocation partner in lipoma (LPP), transcript variant 1, mRNA [NM_005578]	NM_005578
LRAT	-2,49	Homo sapiens lecithin retinol acyltransferase (phosphatidylcholine--retinol O-acyltransferase) (LRAT), transcript variant 1, mRNA [NM_004744]	NM_004744
LRFN2	1,54	leucine rich repeat and fibronectin type III domain containing 2 [Source:HGNC Symbol;Acc:HGNC:21226] [ENST00000338305]	
LRP1B	1,58	Homo sapiens low density lipoprotein receptor-related protein 1B (LRP1B), mRNA [NM_018557]	NM_018557
LRRC10B	2,32	Homo sapiens leucine rich repeat containing 10B (LRRC10B), mRNA [NM_001145077]	NM_001145077
LRRC37A2	1,52	Homo sapiens leucine rich repeat containing 37, member A2 (LRRC37A2), mRNA [NM_001006607]	NM_001006607
LRRC8B	-1,60	Homo sapiens leucine rich repeat containing 8 family, member B (LRRC8B), transcript variant 1, mRNA [NM_015350]	NM_015350

LRRIQ1	1,89	Homo sapiens leucine-rich repeats and IQ motif containing 1 (LRRIQ1), mRNA [NM_001079910]	NM_001079910
LTBP1	1,54	Homo sapiens latent transforming growth factor beta binding protein 1 (LTBP1), transcript variant 1, mRNA [NM_206943]	NM_206943
LYNX1	-1,50	Homo sapiens Ly6/neurotoxin 1 (LYNX1), transcript variant SLURP2, mRNA [NM_177458]	NM_177458
LYPD6	1,63	Homo sapiens LY6/PLAUR domain containing 6 (LYPD6), transcript variant 2, mRNA [NM_194317]	NM_194317
LYRM5	1,54	Homo sapiens LYR motif containing 5 (LYRM5), mRNA [NM_001001660]	NM_001001660
MAGI2	1,50	Homo sapiens membrane associated guanylate kinase, WW and PDZ domain containing 2 (MAGI2), transcript variant 1, mRNA [NM_012301]	NM_012301
MAP3K4	1,79	Homo sapiens mitogen-activated protein kinase kinase kinase 4 (MAP3K4), transcript variant 2, mRNA [NM_006724]	NM_006724
MAPT-AS1	-3,38	Homo sapiens MAPT antisense RNA 1 (MAPT-AS1), long non-coding RNA [NR_024559]	NR_024559
MARVELD1	1,57	Homo sapiens MARVEL domain containing 1 (MARVELD1), mRNA [NM_031484]	NM_031484
MCTS2P	1,51	Homo sapiens malignant T cell amplified sequence 2, pseudogene (MCTS2P), non-coding RNA [NR_003677]	NR_003677
ME1	1,69	Homo sapiens malic enzyme 1, NADP(+)-dependent, cytosolic (ME1), mRNA [NM_002395]	NM_002395
MED11	1,85	Homo sapiens mediator complex subunit 11 (MED11), mRNA [NM_001001683]	NM_001001683
MED29	1,52	Homo sapiens mediator complex subunit 29 (MED29), mRNA [NM_017592]	NM_017592
MEG3	1,70	Homo sapiens maternally expressed 3 (non-protein coding) (MEG3), transcript variant 1, long non-coding RNA [NR_002766]	NR_002766
MEIG1	-1,59	Homo sapiens meiosis/spermiogenesis associated 1 (MEIG1), mRNA [NM_001080836]	NM_001080836
MFI2	1,72	Homo sapiens antigen p97 (melanoma associated) identified by monoclonal antibodies 133.2 and 96.5 (MFI2), transcript variant 1, mRNA [NM_005929]	NM_005929
MGAT3	-1,53	Homo sapiens mannosyl (beta-1,4-)-glycoprotein beta-1,4-N-acetylglucosaminyltransferase (MGAT3), transcript variant 1, mRNA [NM_002409]	NM_002409
MGAT5	1,54	Homo sapiens mannosyl (alpha-1,6-)-glycoprotein beta-1,6-N-acetyl-glucosaminyltransferase (MGAT5), mRNA [NM_002410]	NM_002410
MIDN	1,58	Homo sapiens midnolin (MIDN), mRNA [NM_177401]	NM_177401
MIER3	-1,86	Homo sapiens mesoderm induction early response 1, family member 3 (MIER3), transcript variant 3, mRNA [NM_152622]	NM_152622
MINPP1	-1,57	Homo sapiens multiple inositol-polyphosphate phosphatase 1 (MINPP1), transcript variant 2, mRNA [NM_001178117]	NM_001178117
MIR99AHG	1,60	Homo sapiens mir-99a-let-7c cluster host gene (non-protein coding) (MIR99AHG), transcript variant 1, long non-coding RNA [NR_027790]	NR_027790
MKL2	-1,96	Homo sapiens cDNA FLJ36258 fis, clone THYMU2002450. [AK093577]	
MKNK2	1,53	Homo sapiens MAP kinase interacting serine/threonine kinase 2 (MKNK2), transcript variant 1, mRNA [NM_017572]	NM_017572
MMP23B	1,87	Homo sapiens matrix metallopeptidase 23B (MMP23B), mRNA [NM_006983]	NM_006983
MORC4	-1,52	Homo sapiens MORC family CW-type zinc finger 4 (MORC4), transcript variant 1, mRNA [NM_024657]	NM_024657
MORN1	1,60	Homo sapiens MORN repeat containing 1 (MORN1), transcript variant 3, non-coding RNA [NR_125361]	NR_125361
MRGPRF	2,49	Homo sapiens MAS-related GPR, member F (MRGPRF), transcript variant 2, mRNA [NM_145015]	NM_145015
MS4A7	-3,89	Homo sapiens membrane-spanning 4-domains, subfamily A, member 7 (MS4A7), transcript variant 1, mRNA [NM_021201]	NM_021201

MSRB3	1,65	Homo sapiens methionine sulfoxide reductase B3 (MSRB3), transcript variant 3, mRNA [NM_001193460]	NM_001193460
MSX2	-1,63	Homo sapiens msh homeobox 2 (MSX2), mRNA [NM_002449]	NM_002449
MT1G	1,94	Homo sapiens metallothionein 1G (MT1G), transcript variant 2, mRNA [NM_001301267]	NM_001301267
MTCL1	1,69	Homo sapiens microtubule crosslinking factor 1 (MTCL1), mRNA [NM_015210]	NM_015210
MTM1	1,50	Homo sapiens myotubularin 1 (MTM1), mRNA [NM_000252]	NM_000252
MUC3A	1,84	Homo sapiens mucin 3A, cell surface associated (MUC3A), mRNA [NM_005960]	NM_005960
MXI1	1,54	Homo sapiens MAX interactor 1, dimerization protein (MXI1), transcript variant 1, mRNA [NM_005962]	NM_005962
MYBL1	-1,54	Homo sapiens v-myb avian myeloblastosis viral oncogene homolog-like 1 (MYBL1), transcript variant 3, mRNA [NM_001294282]	NM_001294282
MYCL	1,70	Homo sapiens v-myc avian myelocytomatosis viral oncogene lung carcinoma derived homolog (MYCL), transcript variant 1, mRNA [NM_001033081]	NM_001033081
MYOM1	1,58	Homo sapiens myomesin 1 (MYOM1), transcript variant 1, mRNA [NM_003803]	NM_003803
MYPOP	1,88	Myb-related transcription factor, partner of profilin [Source:HGNC Symbol;Acc:HGNC:20178] [ENST00000322217]	
NALCN	1,51	sodium leak channel, non-selective [Source:HGNC Symbol;Acc:HGNC:19082] [ENST00000470333]	
NBR2	-1,53	Homo sapiens neighbor of BRCA1 gene 2 (non-protein coding) (NBR2), long non-coding RNA [NR_003108]	NR_003108
NCKAP5L	1,74	NCK-associated protein 5-like [Source:HGNC Symbol;Acc:HGNC:29321] [ENST00000491441]	
NCMAP	-1,94	Homo sapiens noncompact myelin associated protein (NCMAP), mRNA [NM_001010980]	NM_001010980
NDST4	-1,61	Homo sapiens N-deacetylase/N-sulfotransferase (heparan glucosaminyl) 4 (NDST4), mRNA [NM_022569]	NM_022569
NDUFA4	-2,17	Homo sapiens NDUFA4, mitochondrial complex associated (NDUFA4), mRNA [NM_002489]	NM_002489
NEBL	1,95	Homo sapiens nebulette (NEBL), transcript variant 1, mRNA [NM_006393]	NM_006393
NECAP1	-1,77	Homo sapiens NECAP endocytosis associated 1 (NECAP1), transcript variant 1, mRNA [NM_015509]	NM_015509
NEK6	-1,80	NIMA-related kinase 6 [Source:HGNC Symbol;Acc:HGNC:7749] [ENST00000373596]	XM_005251664
NF1	1,56	Homo sapiens neurofibromin 1 (NF1), transcript variant 2, mRNA [NM_000267]	NM_000267
NHLRC1	1,83	Homo sapiens NHL repeat containing E3 ubiquitin protein ligase 1 (NHLRC1), mRNA [NM_198586]	NM_198586
NHS	1,74	Homo sapiens Nance-Horan syndrome (congenital cataracts and dental anomalies) (NHS), transcript variant 1, mRNA [NM_198270]	NM_198270
NID1	-1,54	Homo sapiens nidogen 1 (NID1), mRNA [NM_002508]	NM_002508
NINL	1,58	Homo sapiens ninein-like (NINL), mRNA [NM_025176]	NM_025176
NKX2-2	1,60	Homo sapiens NK2 homeobox 2 (NKX2-2), mRNA [NM_002509]	NM_002509
NLRP1	-1,76	Homo sapiens NLR family, pyrin domain containing 1 (NLRP1), transcript variant 5, mRNA [NM_001033053]	NM_001033053
NOLC1	2,18	Homo sapiens nucleolar and coiled-body phosphoprotein 1 (NOLC1), transcript variant 1, mRNA [NM_001284388]	NM_001284388
NPAS3	2,15	Homo sapiens neuronal PAS domain protein 3 (NPAS3), transcript variant 2, mRNA [NM_022123]	NM_022123
NPR3	1,91	Homo sapiens natriuretic peptide receptor 3 (NPR3), transcript variant 1, mRNA [NM_001204375]	NM_001204375
NPSR1	-1,95	Homo sapiens neuropeptide S receptor 1 (NPSR1), transcript variant 4, mRNA [NM_001300934]	NM_001300934

NRIP1	-1,81	Homo sapiens nuclear receptor interacting protein 1 (NRIP1), mRNA [NM_003489]	NM_003489
NRK	1,86	Homo sapiens Nik related kinase (NRK), mRNA [NM_198465]	NM_198465
NRSN1	1,65	neurensin 1 [Source:HGNC Symbol;Acc:HGNC:17881] [ENST00000378475]	
NTN4	1,60	Homo sapiens netrin 4 (NTN4), mRNA [NM_021229]	NM_021229
NUTM2B	-4,26	Homo sapiens NUT family member 2B (NUTM2B), mRNA [NM_001278495]	NM_001278495
NXPH3	-1,63	Homo sapiens neurexophilin 3 (NXPH3), mRNA [NM_007225]	NM_007225
OGFR	2,22	Homo sapiens opioid growth factor receptor (OGFR), mRNA [NM_007346]	NM_007346
OPHN1	-1,51	Homo sapiens oligophrenin 1 (OPHN1), mRNA [NM_002547]	NM_002547
OR2T27	1,58	Homo sapiens olfactory receptor, family 2, subfamily T, member 27 (OR2T27), mRNA [NM_001001824]	NM_001001824
OR8H1	1,58	Homo sapiens olfactory receptor, family 8, subfamily H, member 1 (OR8H1), mRNA [NM_001005199]	NM_001005199
OSBPL5	1,92	Homo sapiens oxysterol binding protein-like 5 (OSBPL5), transcript variant 1, mRNA [NM_020896]	NM_020896
OTOG	1,55	Homo sapiens otogelin (OTOG), transcript variant 1, mRNA [NM_001277269]	NM_001277269
OTUB2	1,53	Homo sapiens OTU deubiquitinase, ubiquitin aldehyde binding 2 (OTUB2), mRNA [NM_023112]	NM_023112
P2RY10	1,83	Homo sapiens purinergic receptor P2Y, G-protein coupled, 10 (P2RY10), transcript variant 1, mRNA [NM_014499]	NM_014499
PAGE1	-59,50	Homo sapiens P antigen family, member 1 (prostate associated) (PAGE1), mRNA [NM_003785]	NM_003785
PALM3	1,65	Homo sapiens paralemmin 3 (PALM3), mRNA [NM_001145028]	NM_001145028
PALM3	1,53	Homo sapiens paralemmin 3 (PALM3), mRNA [NM_001145028]	NM_001145028
PAPD7	1,50	Homo sapiens PAP associated domain containing 7 (PAPD7), transcript variant 1, mRNA [NM_006999]	NM_006999
PAPLN	1,65	Homo sapiens papilin, proteoglycan-like sulfated glycoprotein (PAPLN), mRNA [NM_173462]	NM_173462
PASD1	-2,47	Homo sapiens PAS domain containing 1 (PASD1), mRNA [NM_173493]	NM_173493
PAXIP1-AS2	1,92	Homo sapiens, clone IMAGE:5745756, mRNA. [BC050402]	
PCBD1	-2,18	Homo sapiens pterin-4 alpha-carbinolamine dehydratase/dimerization cofactor of hepatocyte nuclear factor 1 alpha (PCBD1), transcript variant 1, mRNA [NM_000281]	NM_000281
PCDHGB4	-1,64	Homo sapiens protocadherin gamma subfamily B, 4 (PCDHGB4), transcript variant 2, mRNA [NM_032098]	NM_032098
PCNXL3	2,14	Homo sapiens pecanex-like 3 (Drosophila) (PCNXL3), mRNA [NM_032223]	NM_032223
PDE6A	1,54	Homo sapiens phosphodiesterase 6A, cGMP-specific, rod, alpha (PDE6A), mRNA [NM_000440]	NM_000440
PDE8A	1,61	Homo sapiens phosphodiesterase 8A (PDE8A), transcript variant 3, mRNA [NM_001243137]	NM_001243137
PDZK1	-2,25	Homo sapiens PDZ domain containing 1 (PDZK1), transcript variant 1, mRNA [NM_002614]	NM_002614
PERP	1,86	Homo sapiens PERP, TP53 apoptosis effector (PERP), mRNA [NM_022121]	NM_022121
PFDN6	-1,52	Homo sapiens prefoldin subunit 6 (PFDN6), transcript variant 2, mRNA [NM_014260]	NM_014260
PGBD1	1,53	Homo sapiens piggyBac transposable element derived 1 (PGBD1), transcript variant 2, mRNA [NM_032507]	NM_032507
PGBD5	2,29	Homo sapiens piggyBac transposable element derived 5 (PGBD5), mRNA [NM_001258311]	NM_001258311
PGLYRP2	-1,57	Homo sapiens peptidoglycan recognition protein 2 (PGLYRP2), mRNA [NM_052890]	NM_052890

PGR	-2,07	Homo sapiens progesterone receptor (PGR), transcript variant 2, mRNA [NM_000926]	NM_000926
PHACTR2	1,88	Homo sapiens phosphatase and actin regulator 2 (PHACTR2), transcript variant 1, mRNA [NM_001100164]	NM_001100164
PHLDB1	1,51	Homo sapiens pleckstrin homology-like domain, family B, member 1 (PHLDB1), transcript variant 1, mRNA [NM_015157]	NM_015157
PHLPP2	-1,70	Homo sapiens PH domain and leucine rich repeat protein phosphatase 2 (PHLPP2), transcript variant 2, mRNA [NM_001289003]	NM_001289003
PI15	2,00	Homo sapiens peptidase inhibitor 15 (PI15), mRNA [NM_015886]	NM_015886
PKD1L2	1,66	Homo sapiens polycystic kidney disease 1-like 2 (gene/pseudogene) (PKD1L2), transcript variant 1, mRNA [NM_052892]	NM_052892
PKIA	-1,53	Homo sapiens protein kinase (cAMP-dependent, catalytic) inhibitor alpha (PKIA), transcript variant 1, mRNA [NM_006823]	NM_006823
PLA2G12A	1,82	Homo sapiens phospholipase A2, group XIIA (PLA2G12A), mRNA [NM_030821]	NM_030821
PLA2G4F	1,59	Homo sapiens phospholipase A2, group IVF (PLA2G4F), transcript variant 1, mRNA [NM_213600]	NM_213600
PLAG1	-1,66	Homo sapiens pleiomorphic adenoma gene 1 (PLAG1), transcript variant 1, mRNA [NM_002655]	NM_002655
PLEC	1,88	Homo sapiens plectin (PLEC), transcript variant 6, mRNA [NM_201380]	NM_201380
POLR3G	-1,53	Homo sapiens polymerase (RNA) III (DNA directed) polypeptide G (32kD) (POLR3G), mRNA [NM_006467]	NM_006467
PPARG	2,20	Homo sapiens peroxisome proliferator-activated receptor gamma (PPARG), transcript variant 3, mRNA [NM_138711]	NM_138711
PPIL6	1,71	Homo sapiens peptidylprolyl isomerase (cyclophilin)-like 6 (PPIL6), transcript variant 1, mRNA [NM_173672]	NM_173672
PPIP5K2	1,61	Homo sapiens diphosphoinositol pentakisphosphate kinase 2 (PPIP5K2), transcript variant 2, mRNA [NM_015216]	NM_015216
PPM1A	1,51	Homo sapiens protein phosphatase, Mg2+/Mn2+ dependent, 1A (PPM1A), transcript variant 2, mRNA [NM_177951]	NM_177951
PPP1R14C	-1,92	Homo sapiens protein phosphatase 1, regulatory (inhibitor) subunit 14C (PPP1R14C), mRNA [NM_030949]	NM_030949
PPP1R9B	1,54	Homo sapiens protein phosphatase 1, regulatory subunit 9B (PPP1R9B), mRNA [NM_032595]	NM_032595
PPP6C	-1,55	Homo sapiens protein phosphatase 6, catalytic subunit (PPP6C), transcript variant 1, mRNA [NM_001123355]	NM_001123355
PRDX4	1,58	Homo sapiens peroxiredoxin 4 (PRDX4), mRNA [NM_006406]	NM_006406
PRKCQ-AS1	-1,80	Homo sapiens PRKCQ antisense RNA 1 (PRKCQ-AS1), transcript variant 1, long non-coding RNA [NR_036502]	NR_036502
PRMT6	2,13	Homo sapiens protein arginine methyltransferase 6 (PRMT6), mRNA [NM_018137]	NM_018137
PRPS1	-1,76	Homo sapiens phosphoribosyl pyrophosphate synthetase 1 (PRPS1), transcript variant 1, mRNA [NM_002764]	NM_002764
PSD3	1,61	Homo sapiens pleckstrin and Sec7 domain containing 3 (PSD3), transcript variant 1, mRNA [NM_015310]	NM_015310
PSKH1	1,57	Homo sapiens protein serine kinase H1 (PSKH1), mRNA [NM_006742]	NM_006742
PSTPIP2	-1,55	Homo sapiens proline-serine-threonine phosphatase interacting protein 2 (PSTPIP2), mRNA [NM_024430]	NM_024430
PTGES	-1,80	Homo sapiens prostaglandin E synthase (PTGES), mRNA [NM_004878]	NM_004878
PTH1R	-2,01	Homo sapiens parathyroid hormone 1 receptor (PTH1R), transcript variant 1, mRNA [NM_000316]	NM_000316
PTPRJ	1,55	Homo sapiens protein tyrosine phosphatase, receptor type, J (PTPRJ), transcript variant 1, mRNA [NM_002843]	NM_002843
PYGM	1,58	Homo sapiens phosphorylase, glycogen, muscle (PYGM), transcript variant 1, mRNA [NM_005609]	NM_005609
PYGO1	1,55	Homo sapiens pygopus family PHD finger 1 (PYGO1), mRNA [NM_015617]	NM_015617
RAB22A	2,13	Homo sapiens RAB22A, member RAS oncogene family (RAB22A), mRNA [NM_020673]	NM_020673

RAB28	-1,51	Homo sapiens RAB28, member RAS oncogene family (RAB28), transcript variant 2, mRNA [NM_004249]	NM_004249
RAB9B	1,58	Homo sapiens RAB9B, member RAS oncogene family (RAB9B), mRNA [NM_016370]	NM_016370
RAC1	1,58	Homo sapiens ras-related C3 botulinum toxin substrate 1 (rho family, small GTP binding protein Rac1) (RAC1), transcript variant Rac1b, mRNA [NM_018890]	NM_018890
RAD23A	1,79	Homo sapiens RAD23 homolog A (S. cerevisiae) (RAD23A), transcript variant 1, mRNA [NM_005053]	NM_005053
RALGAPA2	1,64	Homo sapiens Ral GTPase activating protein, alpha subunit 2 (catalytic) (RALGAPA2), mRNA [NM_020343]	NM_020343
RAP2C-AS1	1,62	Homo sapiens RAP2C antisense RNA 1 (RAP2C-AS1), long non-coding RNA [NR_110410]	NR_110410
RBAK	1,60	Homo sapiens RB-associated KRAB zinc finger (RBAK), transcript variant 1, mRNA [NM_021163]	NM_021163
RBM12	1,92	Homo sapiens RB-associated KRAB zinc finger (RBAK), transcript variant 1, mRNA [NM_021163]	NM_021163
RBM12	-1,60	Homo sapiens RNA binding motif protein 12 (RBM12), transcript variant 1, mRNA [NM_006047]	NM_006047
RBM15B	1,79	Homo sapiens RNA binding motif protein 15B (RBM15B), mRNA [NM_013286]	NM_013286
RBM24	-1,71	Homo sapiens RNA binding motif protein 24 (RBM24), transcript variant 2, mRNA [NM_153020]	NM_153020
RCHY1	-1,52	Homo sapiens ring finger and CHY zinc finger domain containing 1, E3 ubiquitin protein ligase (RCHY1), transcript variant 9, mRNA [NM_001278539]	NM_001278539
REEP3	-2,04	Homo sapiens receptor accessory protein 3 (REEP3), mRNA [NM_001001330]	NM_001001330
REEP5	2,03	Homo sapiens receptor accessory protein 5 (REEP5), mRNA [NM_005669]	NM_005669
RELL1	1,56	Homo sapiens RELT-like 1 (RELL1), transcript variant 1, mRNA [NM_001085400]	NM_001085400
RGS18	-2,90	Homo sapiens regulator of G-protein signaling 18 (RGS18), mRNA [NM_130782]	NM_130782
RGS19	2,00	Homo sapiens regulator of G-protein signaling 19 (RGS19), transcript variant 1, mRNA [NM_005873]	NM_005873
RIMS3	1,77	Homo sapiens regulating synaptic membrane exocytosis 3 (RIMS3), mRNA [NM_014747]	NM_014747
RIT1	1,93	Homo sapiens Ras-like without CAAX 1 (RIT1), transcript variant 2, mRNA [NM_006912]	NM_006912
RLBP1	1,53	Homo sapiens retinaldehyde binding protein 1 (RLBP1), mRNA [NM_000326]	NM_000326
RNF207	2,03	ring finger protein 207 [Source:HGNC Symbol;Acc:HGNC:32947] [ENST00000466994]	
RNF216P1	-1,67	Homo sapiens ring finger protein 216 pseudogene 1 (RNF216P1), transcript variant 1, non-coding RNA [NR_023384]	NR_023384
RPL23AP64	1,56	Homo sapiens ribosomal protein L23a pseudogene 64 (RPL23AP64), non-coding RNA [NR_003040]	NR_003040
RPL26L1	1,69	Homo sapiens ribosomal protein L26-like 1 (RPL26L1), mRNA [NM_016093]	NM_016093
RPP14	-1,87	Homo sapiens ribonuclease P/MRP 14kDa subunit (RPP14), transcript variant 1, mRNA [NM_001098783]	NM_001098783
RPP30	1,59	Homo sapiens ribonuclease P/MRP 30kDa subunit (RPP30), transcript variant 2, mRNA [NM_006413]	NM_006413
RPS6KA2	-1,51	Homo sapiens ribosomal protein S6 kinase, 90kDa, polypeptide 2 (RPS6KA2), transcript variant 1, mRNA [NM_021135]	NM_021135
RQCD1	-1,84	Homo sapiens RCD1 required for cell differentiation1 homolog (S. pombe) (RQCD1), transcript variant 2, mRNA [NM_005444]	NM_005444
RRP15	-1,70	Homo sapiens ribosomal RNA processing 15 homolog (S. cerevisiae) (RRP15), mRNA [NM_016052]	NM_016052
RSG1	1,69	Homo sapiens REM2 and RAB-like small GTPase 1 (RSG1), mRNA [NM_030907]	NM_030907
RSPH1	1,89	Homo sapiens radial spoke head 1 homolog (Chlamydomonas) (RSPH1), transcript variant 1, mRNA [NM_080860]	NM_080860

RSPO3	1,76	Homo sapiens R-spondin 3 (RSPO3), mRNA [NM_032784]	NM_032784
RUNDC3A	1,53	Homo sapiens RUN domain containing 3A (RUNDC3A), transcript variant 2, mRNA [NM_006695]	NM_006695
SC5D	1,57	Homo sapiens sterol-C5-desaturase (SC5D), transcript variant 2, mRNA [NM_001024956]	NM_001024956
SCARA5	1,54	scavenger receptor class A, member 5 [Source:HGNC Symbol;Acc:HGNC:28701] [ENST00000518030]	
SCIN	-1,50	Homo sapiens scinderin (SCIN), transcript variant 2, mRNA [NM_033128]	NM_033128
SCN1B	1,51	Homo sapiens sodium channel, voltage gated, type I beta subunit (SCN1B), transcript variant b, mRNA [NM_199037]	NM_199037
SCN8A	1,60	Homo sapiens sodium channel, voltage gated, type VIII alpha subunit (SCN8A), transcript variant 1, mRNA [NM_014191]	NM_014191
SECISBP2	2,22	Homo sapiens cDNA FLJ45753 fis, clone LYMPB2002478. [AK127655]	
SELV	-4,29	Homo sapiens selenoprotein V (SELV), mRNA [NM_182704]	NM_182704
SEMA4D	1,54	Homo sapiens sema domain, immunoglobulin domain (Ig), transmembrane domain (TM) and short cytoplasmic domain, (semaphorin) 4D (SEMA4D), transcript variant 1, mRNA [NM_006378]	NM_006378
SEMA6B	1,67	Homo sapiens sema domain, transmembrane domain (TM), and cytoplasmic domain, (semaphorin) 6B (SEMA6B), mRNA [NM_032108]	NM_032108
SERPINB9	-1,56	Homo sapiens serpin peptidase inhibitor, clade B (ovalbumin), member 9 (SERPINB9), mRNA [NM_004155]	NM_004155
SETD7	2,06	Homo sapiens SET domain containing (lysine methyltransferase) 7 (SETD7), mRNA [NM_030648]	NM_030648
SGK494	1,66	Homo sapiens uncharacterized serine/threonine-protein kinase SgK494 (SGK494), mRNA [NM_001174103]	NM_001174103
SGPP1	-1,53	Homo sapiens sphingosine-1-phosphate phosphatase 1 (SGPP1), mRNA [NM_030791]	NM_030791
SH3GL3	1,68	Homo sapiens SH3-domain GRB2-like 3 (SH3GL3), transcript variant EEN-B2-L1, mRNA [NM_003027]	NM_003027
SHC3	1,75	Homo sapiens SHC (Src homology 2 domain containing) transforming protein 3 (SHC3), mRNA [NM_016848]	NM_016848
SIRPG	1,65	Homo sapiens signal-regulatory protein gamma (SIRPG), transcript variant 1, mRNA [NM_018556]	NM_018556
SIRT5	1,61	Homo sapiens sirtuin 5 (SIRT5), transcript variant 2, mRNA [NM_031244]	NM_031244
SIX2	1,77	Homo sapiens SIX homeobox 2 (SIX2), mRNA [NM_016932]	NM_016932
SKIDA1	1,76	Homo sapiens SKI/DACH domain containing 1 (SKIDA1), mRNA [NM_207371]	NM_207371
SLA2	1,60	Homo sapiens Src-like-adaptor 2 (SLA2), transcript variant 1, mRNA [NM_032214]	NM_032214
SLC14A2	1,53	Homo sapiens solute carrier family 14 (urea transporter), member 2 (SLC14A2), transcript variant 1, mRNA [NM_007163]	NM_007163
SLC22A7	-1,64	Homo sapiens solute carrier family 22 (organic anion transporter), member 7 (SLC22A7), transcript variant 2, mRNA [NM_153320]	NM_153320
SLC26A11	1,70	Homo sapiens solute carrier family 26 (anion exchanger), member 11 (SLC26A11), transcript variant 1, mRNA [NM_001166347]	NM_001166347
SLC2A3	-2,99	Homo sapiens solute carrier family 2 (facilitated glucose transporter), member 3 (SLC2A3), mRNA [NM_006931]	NM_006931
SLC39A2	-1,60	Homo sapiens solute carrier family 39 (zinc transporter), member 2 (SLC39A2), transcript variant 1, mRNA [NM_014579]	NM_014579
SLC46A3	1,94	Homo sapiens solute carrier family 46, member 3 (SLC46A3), transcript variant 1, mRNA [NM_181785]	NM_181785
SLC52A3	1,51	Homo sapiens solute carrier family 52 (riboflavin transporter), member 3 (SLC52A3), mRNA [NM_033409]	NM_033409
SLC5A5	1,92	Homo sapiens solute carrier family 5 (sodium/iodide cotransporter), member 5 (SLC5A5), mRNA [NM_000453]	NM_000453

SLC6A8	1,51	Homo sapiens solute carrier family 6 (neurotransmitter transporter), member 8 (SLC6A8), transcript variant 1, mRNA [NM_005629]	NM_005629
SLC8A1	1,67	Homo sapiens solute carrier family 8 (sodium/calcium exchanger), member 1 (SLC8A1), transcript variant A, mRNA [NM_021097]	NM_021097
SMCO1	1,51	Homo sapiens single-pass membrane protein with coiled-coil domains 1 (SMCO1), mRNA [NM_001077657]	NM_001077657
SNHG4	-1,66	Homo sapiens small nucleolar RNA host gene 4 (non-protein coding) (SNHG4), transcript variant 1, long non-coding RNA [NR_003141]	NR_003141
SNRPC	-1,73	Homo sapiens small nuclear ribonucleoprotein polypeptide C (SNRPC), transcript variant 1, mRNA [NM_003093]	NM_003093
SOX9	1,79	Homo sapiens SRY (sex determining region Y)-box 9 (SOX9), mRNA [NM_000346]	NM_000346
SPACA3	-1,91	Homo sapiens sperm acrosome associated 3 (SPACA3), mRNA [NM_173847]	NM_173847
SPACA6P	1,66	Homo sapiens sperm acrosome associated 6, pseudogene (SPACA6P), long non-coding RNA [NR_024330]	NR_024330
SPANXA1	1,56	Homo sapiens sperm protein associated with the nucleus, X-linked, family member A1 (SPANXA1), mRNA [NM_013453]	NM_013453
SPATA6L	-1,68	Homo sapiens spermatogenesis associated 6-like (SPATA6L), mRNA [NM_001039395]	NM_001039395
SPINK4	-1,57	Homo sapiens serine peptidase inhibitor, Kazal type 4 (SPINK4), mRNA [NM_014471]	NM_014471
SPRR2D	1,67	Homo sapiens small proline-rich protein 2D (SPRR2D), mRNA [NM_006945]	NM_006945
SPRR2G	-1,71	Homo sapiens small proline-rich protein 2G (SPRR2G), mRNA [NM_001014291]	NM_001014291
SRGAP2	1,85	Homo sapiens SLIT-ROBO Rho GTPase activating protein 2 (SRGAP2), transcript variant 4, mRNA [NM_001300952]	NM_001300952
SRSF8	1,77	Homo sapiens serine/arginine-rich splicing factor 8 (SRSF8), transcript variant 1, mRNA [NM_032102]	NM_032102
ST20-AS1	1,60	Homo sapiens ST20 antisense RNA 1 (ST20-AS1), long non-coding RNA [NR_028330]	NR_028330
ST3GAL4	1,54	Homo sapiens cDNA FLJ11867 fis, clone HEMBA1006976, weakly similar to H.sapiens mRNA for Gal-beta(1-3/1-4)GlcNAc alpha-2.3-sialyltransferase. [AK021929]	
ST3GAL5	1,55	Homo sapiens ST3 beta-galactoside alpha-2,3-sialyltransferase 5 (ST3GAL5), transcript variant 1, mRNA [NM_003896]	NM_003896
STX12	1,94	Homo sapiens syntaxin 12 (STX12), mRNA [NM_177424]	NM_177424
SULF1	1,53	Homo sapiens sulfatase 1 (SULF1), transcript variant 3, mRNA [NM_015170]	NM_015170
SULT6B1	-1,93	Homo sapiens sulfotransferase family, cytosolic, 6B, member 1 (SULT6B1), mRNA [NM_001032377]	NM_001032377
SUV39H2	1,79	Homo sapiens suppressor of variegation 3-9 homolog 2 (Drosophila) (SUV39H2), transcript variant 3, mRNA [NM_024670]	NM_024670
SYNE3	1,65	Homo sapiens spectrin repeat containing, nuclear envelope family member 3 (SYNE3), mRNA [NM_152592]	NM_152592
SYTL3	1,56	Homo sapiens synaptotagmin-like 3 (SYTL3), transcript variant 3, mRNA [NM_001009991]	NM_001009991
TACSTD2	-1,52	Homo sapiens tumor-associated calcium signal transducer 2 (TACSTD2), mRNA [NM_002353]	NM_002353
TAF1B	1,79	TATA box binding protein (TBP)-associated factor, RNA polymerase I, B, 63kDa [Source:HGNC Symbol;Acc:HGNC:11533] [ENST00000402170]	
TBC1D10B	1,97	Homo sapiens TBC1 domain family, member 10B (TBC1D10B), mRNA [NM_015527]	NM_015527
TBC1D22A	-1,62	Homo sapiens TBC1 domain family, member 22A (TBC1D22A), transcript variant 5, non-coding RNA [NR_104292]	NR_104292
TBC1D9	2,15	Homo sapiens TBC1 domain family, member 9 (with GRAM domain) (TBC1D9), mRNA [NM_015130]	NM_015130

TBC1D9B	1,60	Homo sapiens TBC1 domain family, member 9B (with GRAM domain) (TBC1D9B), transcript variant 1, mRNA [NM_198868]	NM_198868
TCF3	1,81	Homo sapiens transcription factor 3 (TCF3), transcript variant 1, mRNA [NM_003200]	NM_003200
TCF7L1	1,64	Homo sapiens transcription factor 7-like 1 (T-cell specific, HMG-box) (TCF7L1), mRNA [NM_031283]	NM_031283
TCOF1	1,54	Homo sapiens Treacher Collins-Franceschetti syndrome 1 (TCOF1), transcript variant 4, mRNA [NM_001135243]	NM_001135243
TCTN2	1,65	Homo sapiens tectonic family member 2 (TCTN2), transcript variant 1, mRNA [NM_024809]	NM_024809
TDRD12	2,75	Homo sapiens tudor domain containing 12 (TDRD12), mRNA [NM_001110822]	NM_001110822
TFF1	-1,77	Homo sapiens trefoil factor 1 (TFF1), mRNA [NM_003225]	NM_003225
THAP7-AS1	2,18	Homo sapiens THAP7 antisense RNA 1 (THAP7-AS1), transcript variant 2, long non-coding RNA [NR_027052]	NR_027052
THBS4	1,71	Homo sapiens thrombospondin 4 (THBS4), mRNA [NM_003248]	NM_003248
THSD4	-1,52	Homo sapiens thrombospondin, type I, domain containing 4 (THSD4), transcript variant 1, mRNA [NM_024817]	NM_024817
TIFA	1,50	Homo sapiens TRAF-interacting protein with forkhead-associated domain (TIFA), mRNA [NM_052864]	NM_052864
TM7SF3	1,54	Homo sapiens transmembrane 7 superfamily member 3 (TM7SF3), mRNA [NM_016551]	NM_016551
TMC8	-1,72	Homo sapiens transmembrane channel-like 8 (TMC8), mRNA [NM_152468]	NM_152468
TMCC2	-1,56	Homo sapiens transmembrane and coiled-coil domain family 2 (TMCC2), transcript variant 1, mRNA [NM_014858]	NM_014858
TMEM106A	1,72	Homo sapiens transmembrane protein 106A (TMEM106A), transcript variant 1, mRNA [NM_001291586]	NM_001291586
TMEM154	-1,54	Homo sapiens transmembrane protein 154 (TMEM154), mRNA [NM_152680]	NM_152680
TMEM204	-1,69	Homo sapiens transmembrane protein 204 (TMEM204), transcript variant 1, mRNA [NM_024600]	NM_024600
TMEM53	1,86	transmembrane protein 53 [Source:HGNC Symbol;Acc:HGNC:26186] [ENST00000372244]	XR_425151
TMPRSS9	1,64	transmembrane protease, serine 9 [Source:HGNC Symbol;Acc:HGNC:30079] [ENST00000395264]	
TMTC2	1,51	Homo sapiens transmembrane and tetratricopeptide repeat containing 2 (TMTC2), mRNA [NM_152588]	NM_152588
TNNT2	-1,78	Homo sapiens troponin T type 2 (cardiac) (TNNT2), transcript variant 1, mRNA [NM_000364]	NM_000364
TNRC6C	1,86	Homo sapiens trinucleotide repeat containing 6C (TNRC6C), transcript variant 1, mRNA [NM_001142640]	NM_001142640
TOMM22	1,64	Homo sapiens translocase of outer mitochondrial membrane 22 homolog (yeast) (TOMM22), mRNA [NM_020243]	NM_020243
TOX3	1,58	Homo sapiens TOX high mobility group box family member 3 (TOX3), transcript variant 2, mRNA [NM_001146188]	NM_001146188
TP73	1,51	Homo sapiens tumor protein p73 (TP73), transcript variant 1, mRNA [NM_005427]	NM_005427
TPRXL	-1,86	Homo sapiens tetra-peptide repeat homeobox-like (TPRXL), non-coding RNA [NR_002223]	NR_002223
TRAF3	1,61	Homo sapiens TNF receptor-associated factor 3 (TRAF3), transcript variant 1, mRNA [NM_145725]	NM_145725
TRAF3IP3	-2,76	Homo sapiens TRAF3 interacting protein 3 (TRAF3IP3), transcript variant 2, mRNA [NM_001287754]	NM_001287754
TRAK1	1,52	Homo sapiens trafficking protein, kinesin binding 1 (TRAK1), transcript variant 3, mRNA [NM_001265608]	NM_001265608
TRIM36	1,59	Homo sapiens tripartite motif-containing 36, mRNA (cDNA clone IMAGE:5015503). [BC017346]	
TRIM41	-1,58	Homo sapiens tripartite motif containing 41 (TRIM41), transcript variant 2, mRNA [NM_201627]	NM_201627
TRIOBP	2,11	Homo sapiens TRIO and F-actin binding protein (TRIOBP), transcript variant 6, mRNA [NM_001039141]	NM_001039141
TSC22D1	1,90	Homo sapiens TSC22 domain family, member 1 (TSC22D1), transcript variant 1, mRNA [NM_183422]	NM_183422

TSEN15	-1,89	Homo sapiens TSEN15 tRNA splicing endonuclease subunit (TSEN15), transcript variant 3, non-coding RNA [NR_023349]	NR_023349
TSHZ3	2,02	Homo sapiens teashirt zinc finger homeobox 3 (TSHZ3), mRNA [NM_020856]	NM_020856
TSPAN17	1,74	Homo sapiens tetraspanin 17 (TSPAN17), transcript variant 1, mRNA [NM_012171]	NM_012171
TSPY10	-2,35	Homo sapiens testis specific protein, Y-linked 10 (TSPY10), mRNA [NM_001282469]	NM_001282469
TUBA3D	-1,59	Homo sapiens tubulin, alpha 3d (TUBA3D), mRNA [NM_080386]	NM_080386
TULP1	1,66	Homo sapiens tubby like protein 1 (TULP1), transcript variant 1, mRNA [NM_003322]	NM_003322
TUSC8	-1,51	tumor suppressor candidate 8 (non-protein coding) [Source:HGNC Symbol;Acc:HGNC:49111] [ENST00000607312]	
UBA7	-1,56	Homo sapiens ubiquitin-like modifier activating enzyme 7 (UBA7), mRNA [NM_003335]	NM_003335
UBE2E1	1,94	Homo sapiens ubiquitin-conjugating enzyme E2E 1 (UBE2E1), transcript variant 1, mRNA [NM_003341]	NM_003341
UBE2Q2P2	-1,56	Homo sapiens ubiquitin-conjugating enzyme E2Q family member 2 pseudogene 2 (UBE2Q2P2), non-coding RNA [NR_004847]	NR_004847
UBE3B	1,85	Homo sapiens ubiquitin protein ligase E3B (UBE3B), transcript variant 1, mRNA [NM_130466]	NM_130466
UNC13B	1,51	Homo sapiens unc-13 homolog B (C. elegans) (UNC13B), mRNA [NM_006377]	NM_006377
UQCRB	1,65	Homo sapiens ubiquinol-cytochrome c reductase binding protein (UQCRB), transcript variant 3, mRNA [NM_001254752]	NM_001254752
USP17L7	1,74	Homo sapiens ubiquitin specific peptidase 17-like family member 7 (USP17L7), mRNA [NM_001256869]	NM_001256869
USP21	1,54	Homo sapiens ubiquitin specific peptidase 21 (USP21), transcript variant 3, mRNA [NM_001014443]	NM_001014443
USP27X-AS1	1,54	Homo sapiens USP27X antisense RNA 1 (head to head) (USP27X-AS1), long non-coding RNA [NR_026742]	NR_026742
UXT	-2,38	ubiquitously-expressed, prefoldin-like chaperone [Source:HGNC Symbol;Acc:HGNC:12641] [ENST00000376964]	
VAC14	2,14	Vac14 homolog (S. cerevisiae) [Source:HGNC Symbol;Acc:HGNC:25507] [ENST00000571759]	XM_005256038
VAT1L	-3,40	Homo sapiens vesicle amine transport 1-like (VAT1L), mRNA [NM_020927]	NM_020927
VLDLR-AS1	-1,61	Homo sapiens VLDLR antisense RNA 1 (VLDLR-AS1), long non-coding RNA [NR_015375]	NR_015375
VOPP1	1,63	Homo sapiens vesicular, overexpressed in cancer, prosurvival protein 1 (VOPP1), transcript variant 1, mRNA [NM_030796]	NM_030796
WBP4	2,12	Homo sapiens WW domain binding protein 4 (WBP4), mRNA [NM_007187]	NM_007187
WDR13	-1,93	Homo sapiens WD repeat domain 13 (WDR13), transcript variant 1, mRNA [NM_017883]	NM_017883
WDR5	-1,63	WD repeat domain 5 [Source:HGNC Symbol;Acc:HGNC:12757] [ENST00000608937]	XM_005272163
WDR55	-1,53	Homo sapiens WD repeat domain 55 (WDR55), mRNA [NM_017706]	NM_017706
WDR5B	-1,55	Homo sapiens WD repeat domain 5B (WDR5B), mRNA [NM_019069]	NM_019069
WDR74	1,58	WD repeat domain 74 [Source:HGNC Symbol;Acc:HGNC:25529] [ENST00000538098]	XM_005274055
WNT1	-1,58	Homo sapiens wingless-type MMTV integration site family, member 1 (WNT1), mRNA [NM_005430]	NM_005430
WNT3A	1,75	Homo sapiens wingless-type MMTV integration site family, member 3A (WNT3A), mRNA [NM_033131]	NM_033131
WTAP	1,64	Homo sapiens Wilms tumor 1 associated protein, mRNA (cDNA clone IMAGE:5399821), partial cds. [BC028180]	
XKR8	-1,65	Homo sapiens XK, Kell blood group complex subunit-related family, member 8 (XKR8), mRNA [NM_018053]	NM_018053
ZADH2	1,52	Homo sapiens zinc binding alcohol dehydrogenase domain containing 2 (ZADH2), mRNA [NM_175907]	NM_175907

ZBTB7C	-1,67	Homo sapiens zinc finger and BTB domain containing 7C (ZBTB7C), mRNA [NM_001039360]	NM_001039360
ZDHHC16	1,62	Homo sapiens zinc finger, DHHC-type containing 16 (ZDHHC16), transcript variant 5, mRNA [NM_198046]	NM_198046
ZDHHC8P1	2,02	Homo sapiens zinc finger, DHHC-type containing 8 pseudogene 1 (ZDHHC8P1), non-coding RNA [NR_003950]	NR_003950
ZEB2	-1,67	Homo sapiens zinc finger E-box binding homeobox 2 (ZEB2), transcript variant 1, mRNA [NM_014795]	NM_014795
ZFP2	1,55	Homo sapiens ZFP2 zinc finger protein (ZFP2), mRNA [NM_030613]	NM_030613
ZFP37	1,56	Homo sapiens ZFP37 zinc finger protein (ZFP37), transcript variant 3, mRNA [NM_003408]	NM_003408
ZKSCAN1	1,58	Homo sapiens zinc finger with KRAB and SCAN domains 1 (ZKSCAN1), transcript variant 2, mRNA [NM_001287054]	NM_001287054
ZMAT1	1,84	Homo sapiens zinc finger, matrin-type 1 (ZMAT1), transcript variant 2, mRNA [NM_001282400]	NM_001282400
ZMYM6	1,63	zinc finger, MYM-type 6 [Source:HGNC Symbol;Acc:HGNC:13050] [ENST00000460607]	
ZNF100	2,10	zinc finger protein 100 [Source:HGNC Symbol;Acc:HGNC:12880] [ENST00000358296]	XM_005259783
ZNF107	1,82	Homo sapiens zinc finger protein 107 (ZNF107), transcript variant 1, mRNA [NM_016220]	NM_016220
ZNF117	2,37	Homo sapiens zinc finger protein 117 (ZNF117), mRNA [NM_015852]	NM_015852
ZNF137P	1,69	Homo sapiens zinc finger protein 137, pseudogene (ZNF137P), non-coding RNA [NR_023311]	NR_023311
ZNF138	2,20	Homo sapiens zinc finger protein 138 (ZNF138), transcript variant 1, mRNA [NM_006524]	NM_006524
ZNF141	1,53	Homo sapiens zinc finger protein 141 (ZNF141), mRNA [NM_003441]	NM_003441
ZNF160	1,77	Homo sapiens zinc finger protein 160 (ZNF160), transcript variant 2, mRNA [NM_198893]	NM_198893
ZNF17	1,68	Homo sapiens zinc finger protein 17 (ZNF17), mRNA [NM_006959]	NM_006959
ZNF184	1,52	Homo sapiens zinc finger protein 184 (ZNF184), mRNA [NM_007149]	NM_007149
ZNF197	1,88	Homo sapiens zinc finger protein 197 (ZNF197), transcript variant 1, mRNA [NM_006991]	NM_006991
ZNF248	1,65	Homo sapiens zinc finger protein 248 (ZNF248), transcript variant 1, mRNA [NM_021045]	NM_021045
ZNF252P	1,77	Homo sapiens zinc finger protein 252, pseudogene (ZNF252P), non-coding RNA [NR_023392]	NR_023392
ZNF253	1,50	Homo sapiens zinc finger protein 253 (ZNF253), mRNA [NM_021047]	NM_021047
ZNF254	1,59	Homo sapiens zinc finger protein 254 (ZNF254), transcript variant 1, mRNA [NM_001278677]	NM_001278677
ZNF267	2,48	Homo sapiens zinc finger protein 267 (ZNF267), transcript variant 1, mRNA [NM_003414]	NM_003414
ZNF292	-1,70	zinc finger protein 292 [Source:HGNC Symbol;Acc:HGNC:18410] [ENST00000369578]	
ZNF320	1,76	Homo sapiens zinc finger protein 320 (ZNF320), mRNA [NM_207333]	NM_207333
ZNF32-AS3	1,54	Homo sapiens ZNF32 antisense RNA 3 (ZNF32-AS3), long non-coding RNA [NR_038867]	NR_038867
ZNF347	1,66	Homo sapiens zinc finger protein 347 (ZNF347), transcript variant 1, mRNA [NM_001172674]	NM_001172674
ZNF354A	2,03	Homo sapiens zinc finger protein 354A (ZNF354A), mRNA [NM_005649]	NM_005649
ZNF365	-2,62	Homo sapiens zinc finger protein 365 (ZNF365), transcript variant A, mRNA [NM_014951]	NM_014951
ZNF385C	-1,87	zinc finger protein 385C [Source:HGNC Symbol;Acc:HGNC:33722] [ENST00000453355]	
ZNF415	1,92	Homo sapiens zinc finger protein 415 (ZNF415), transcript variant 1, mRNA [NM_001136038]	NM_001136038
ZNF429	1,71	Homo sapiens zinc finger protein 429 (ZNF429), mRNA [NM_001001415]	NM_001001415

ZNF43	1,73	Homo sapiens zinc finger protein 43 (ZNF43), transcript variant 1, mRNA [NM_001256648]	NM_001256648
ZNF430	1,52	Homo sapiens zinc finger protein 430 (ZNF430), transcript variant 1, mRNA [NM_025189]	NM_025189
ZNF468	1,58	Homo sapiens zinc finger protein 468 (ZNF468), transcript variant 1, mRNA [NM_001277120]	NM_001277120
ZNF471	-1,57	Homo sapiens zinc finger protein 471 (ZNF471), mRNA [NM_020813]	NM_020813
ZNF479	1,63	Homo sapiens zinc finger protein 479 (ZNF479), mRNA [NM_033273]	NM_033273
ZNF492	1,78	Homo sapiens zinc finger protein 492 (ZNF492), mRNA [NM_020855]	NM_020855
ZNF493	1,77	Homo sapiens zinc finger protein 493 (ZNF493), transcript variant 3, mRNA [NM_001076678]	NM_001076678
ZNF503-AS2	2,43	Homo sapiens ZNF503 antisense RNA 2 (ZNF503-AS2), transcript variant 5, long non-coding RNA [NR_110301]	NR_110301
ZNF532	1,53	Homo sapiens zinc finger protein 532 (ZNF532), mRNA [NM_018181]	NM_018181
ZNF542P	1,82	Homo sapiens zinc finger protein 542, pseudogene (ZNF542P), transcript variant 4, non-coding RNA [NR_003127]	NR_003127
ZNF585A	1,75	Homo sapiens zinc finger protein 585A (ZNF585A), transcript variant 1, mRNA [NM_152655]	NM_152655
ZNF585B	1,57	Homo sapiens zinc finger protein 585B (ZNF585B), mRNA [NM_152279]	NM_152279
ZNF594	1,60	Homo sapiens zinc finger protein 594 (ZNF594), mRNA [NM_032530]	NM_032530
ZNF600	1,55	Homo sapiens zinc finger protein 600 (ZNF600), mRNA [NM_198457]	NM_198457
ZNF606	1,67	Homo sapiens zinc finger protein 606 (ZNF606), mRNA [NM_025027]	NM_025027
ZNF615	1,51	Homo sapiens zinc finger protein 615 (ZNF615), transcript variant 1, mRNA [NM_001199324]	NM_001199324
ZNF616	2,56	Homo sapiens zinc finger protein 616 (ZNF616), mRNA [NM_178523]	NM_178523
ZNF618	1,60	Homo sapiens zinc finger protein 618 (ZNF618), mRNA [NM_133374]	NM_133374
ZNF624	1,88	Homo sapiens zinc finger protein 624 (ZNF624), mRNA [NM_020787]	NM_020787
ZNF629	1,66	Homo sapiens zinc finger protein 629 (ZNF629), mRNA [NM_001080417]	NM_001080417
ZNF658	2,00	Homo sapiens zinc finger protein 658 (ZNF658), mRNA [NM_033160]	NM_033160
ZNF664	1,66	Homo sapiens zinc finger protein 664 (ZNF664), transcript variant 1, mRNA [NM_152437]	NM_152437
ZNF676	1,51	Homo sapiens zinc finger protein 676 (ZNF676), mRNA [NM_001001411]	NM_001001411
ZNF681	1,59	Homo sapiens zinc finger protein 681 (ZNF681), mRNA [NM_138286]	NM_138286
ZNF682	1,61	Homo sapiens zinc finger protein 682 (ZNF682), transcript variant 1, mRNA [NM_033196]	NM_033196
ZNF705G	1,63	Homo sapiens zinc finger protein 705G (ZNF705G), mRNA [NM_001164457]	NM_001164457
ZNF714	1,56	Homo sapiens zinc finger protein 714 (ZNF714), transcript variant 1, mRNA [NM_182515]	NM_182515
ZNF718	1,70	Homo sapiens zinc finger protein 718 (ZNF718), transcript variant 1, mRNA [NM_001039127]	NM_001039127
ZNF721	2,96	Homo sapiens zinc finger protein 721 (ZNF721), mRNA [NM_133474]	NM_133474
ZNF727	1,50	Homo sapiens zinc finger protein 727 (ZNF727), mRNA [NM_001159522]	NM_001159522
ZNF730	2,03	PREDICTED: Homo sapiens zinc finger protein 730 (ZNF730), transcript variant X1, mRNA [XM_006722596]	XM_006722596
ZNF738	1,52	Homo sapiens zinc finger protein 738 (ZNF738), non-coding RNA [NR_027130]	NR_027130
ZNF761	1,85	Homo sapiens zinc finger protein 761 (ZNF761), transcript variant 1, mRNA [NM_001008401]	NM_001008401

ZNF765	2,44	Homo sapiens zinc finger protein 765 (ZNF765), mRNA [NM_001040185]	NM_001040185
ZNF765	2,45	Homo sapiens zinc finger protein 765 (ZNF765), mRNA [NM_001040185]	NM_001040185
ZNF77	1,51	Homo sapiens zinc finger protein 77 (ZNF77), mRNA [NM_021217]	NM_021217
ZNF774	1,53	Homo sapiens zinc finger protein 774 (ZNF774), mRNA [NM_001004309]	NM_001004309
ZNF788	-2,67	Homo sapiens zinc finger family member 788 (ZNF788), non-coding RNA [NR_027049]	NR_027049
ZNF791	1,63	Homo sapiens zinc finger protein 791 (ZNF791), mRNA [NM_153358]	NM_153358
ZNF793	1,96	Homo sapiens zinc finger protein 793 (ZNF793), mRNA [NM_001013659]	NM_001013659
ZNF805	1,57	Homo sapiens zinc finger protein 805 (ZNF805), transcript variant 1, mRNA [NM_001023563]	NM_001023563
ZNF813	1,79	Homo sapiens zinc finger protein 813 (ZNF813), mRNA [NM_001004301]	NM_001004301
ZNF813	1,65	Homo sapiens zinc finger protein 813 (ZNF813), mRNA [NM_001004301]	NM_001004301
ZNF818P	1,59	Homo sapiens zinc finger protein 818, pseudogene (ZNF818P), non-coding RNA [NR_073396]	NR_073396
ZNF827	1,96	Homo sapiens zinc finger protein 827 (ZNF827), mRNA [NM_178835]	NM_178835
ZNF83	1,74	Homo sapiens zinc finger protein 83 (ZNF83), transcript variant 10, mRNA [NM_001277945]	NM_001277945
ZNF836	1,94	Homo sapiens zinc finger protein 836 (ZNF836), mRNA [NM_001102657]	NM_001102657
ZNF84	1,53	Homo sapiens zinc finger protein 84 (ZNF84), transcript variant 1, mRNA [NM_003428]	NM_003428
ZNF841	1,54	Homo sapiens zinc finger protein 841 (ZNF841), mRNA [NM_001136499]	NM_001136499
ZNF845	1,90	Homo sapiens zinc finger protein 845 (ZNF845), mRNA [NM_138374]	NM_138374
ZNF85	1,58	Homo sapiens zinc finger protein 85 (ZNF85), transcript variant 1, mRNA [NM_003429]	NM_003429
ZNF91	1,66	Homo sapiens zinc finger protein 91 (ZNF91), transcript variant 1, mRNA [NM_003430]	NM_003430
ZNF99	1,51	Homo sapiens zinc finger protein 99 (ZNF99), mRNA [NM_001080409]	NM_001080409
ZSCAN29	1,71	Homo sapiens zinc finger and SCAN domain containing 29 (ZSCAN29), mRNA [NM_152455]	NM_152455
ZSCAN30	2,08	Homo sapiens zinc finger and SCAN domain containing 30 (ZSCAN30), transcript variant 1, mRNA [NM_001166012]	NM_001166012
ZSCAN5B	2,04	Homo sapiens zinc finger and SCAN domain containing 5B (ZSCAN5B), mRNA [NM_001080456]	NM_001080456
ZW10	1,82	Homo sapiens zw10 kinetochore protein (ZW10), mRNA [NM_004724]	NM_004724
